# Flavonoid-based inhibitors of the Phi-class glutathione transferase from black-grass to combat multiple herbicide resistance†‡

**DOI:** 10.1039/d1ob01802g

**Published:** 2021-10-13

**Authors:** Maria Schwarz, Rebecca F. M. Eno, Stefanie Freitag-Pohl, Christopher R. Coxon, Hannah E. Straker, David J. Wortley, David J. Hughes, Glynn Mitchell, Jenny Moore, Ian Cummins, Nawaporn Onkokesung, Melissa Brazier-Hicks, Robert Edwards, Ehmke Pohl, Patrick G. Steel

**Affiliations:** Department of Chemistry, University of Durham, Science Laboratories South Road Durham DH1 3LE UK p.g.steel@durham.ac.uk ehmke.pohl@durham.ac.uk; Centre for Novel Agricultural Products, Department of Biology, University of York York YO10 5DD UK; Syngenta, Jealott's Hill International Research Station Bracknell Berks RG42 6EY UK; Department of Biosciences, University of Durham, Science Laboratories South Road Durham DH1 3LE UK; Agriculture, School of Natural and Environmental Sciences, Newcastle University Newcastle-upon-Tyne NE1 7RU UK

## Abstract

The evolution and growth of multiple-herbicide resistance (MHR) in grass weeds continues to threaten global cereal production. While various processes can contribute to resistance, earlier work has identified the phi class glutathione-*S*-transferase (*Am*GSTF1) as a functional biomarker of MHR in black-grass (*Alopecurus myosuroides*). This study provides further insights into the role of *Am*GSTF1 in MHR using a combination of chemical and structural biology. Crystal structures of wild-type *Am*GSTF1, together with two specifically designed variants that allowed the co-crystal structure determination with glutathione and a glutathione adduct of the *Am*GSTF1 inhibitor 4-chloro-7-nitro-benzofurazan (NBD-Cl) were obtained. These studies demonstrated that the inhibitory activity of NBD-Cl was associated with the occlusion of the active site and the impediment of substrate binding. A search for other selective inhibitors of *Am*GSTF1, using ligand-fishing experiments, identified a number of flavonoids as potential ligands. Subsequent experiments using black-grass extracts discovered a specific flavonoid as a natural ligand of the recombinant enzyme. A series of related synthetic flavonoids was prepared and their binding to *Am*GSTF1 was investigated showing a high affinity for derivatives bearing a *O*-5-decyl-α-carboxylate. Molecular modelling based on high-resolution crystal structures allowed a binding pose to be defined which explained flavonoid binding specificity. Crucially, high binding affinity was linked to a reversal of the herbicide resistance phenotype in MHR black-grass. Collectively, these results present a nature-inspired new lead for the development of herbicide synergists to counteract MHR in weeds.

## Abstract 2

Nature inspired flavonoid derivatives bind to AmGSTF1 and overcome herbicide resistance in multiple herbicide resistant (MHR) Black Grass.

## Graphical abstract

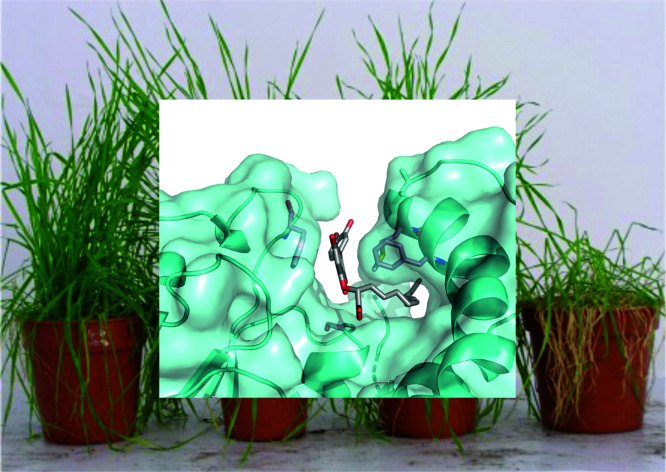

## Introduction

A growing global population, and an increasing demand for meat products, that require a vast amount of feedstock, coupled with a fixed availability of arable land necessitates ever increasing crop yields.^1^ Selective weed control using herbicides is a key contributor to arable productivity, with these crop protection agents representing >44% of total agrochemical inputs.^2^ However, in the case of cereals such as wheat and barley, herbicide resistance in weeds, notably wild grasses, has become a major threat to intensive production.^3,4^ Herbicide resistance can arise from two distinct molecular mechanisms; target site resistance (TSR) and non-target-site resistance (NTSR). TSR results from mutation(s) of the target protein(s) leading to decreased herbicide binding and/or sensitivity, or can arise from the over-production of target protein(s) as a result of gene over-expression.^5^ In contrast, NTSR, which is independent of herbicide mode of action, can occur *via* a number of different protective mechanisms.^6^ These range from decreased cellular uptake of herbicides, to their enhanced detoxification *via* various enzyme families including glutathione-*S*-transferases (GSTs) and cytochromes P450 (CYPs). NTSR based mechanisms are particularly damaging as they are effective against diverse classes of compounds leading to enhanced tolerance to multiple herbicides. In Northern Europe including the United Kingdom (UK), multiple herbicide resistance (MHR) caused by NTSR is best exemplified in black-grass (*Alopecurus myosuroides*), where MHR is now well established and is a major threat to winter wheat production.^4^

Despite its importance in weed control, the specific molecular mechanisms of NTSR are poorly understood and this hampers the development of effective countermeasures.^3^ In recent years, there is growing evidence implicating GSTs as key regulators of NTSR in weeds.^7,8^ Members of the functionally diverse GST super-family were initially implicated in resistance through their ability to conjugate herbicides with glutathione, thereby leading to their inactivation through detoxification.^9^ However, more recently, it has become clear that this is only one of the possible routes that GSTs contribute to MHR. While GSTs are primarily associated with their capability to catalyse the nucleophilic conjugation of glutathione to electrophilic compounds, members of the superfamily also deliver alternative protective functions including acting as glutathione peroxidases (GPOXs), isomerases and as carrier proteins of biologically active secondary metabolites.^7,10^ In black-grass, upregulation of a phi (F)-class GST1 (*Am*GSTF1) has been shown to be a functional biomarker of MHR.^8,11,12^ Thus, *Am*GSTF1 was consistently overexpressed in populations exhibiting resistance to aryloxyphenoxypropionate, phenylurea and sulfonylurea post-emergence herbicides compared to susceptible and TSR populations. Importantly, *Am*GSTF1 orthologues in wild oat (*Avena fatua*) and rigid ryegrass (*Lolium rigidum*) have also been linked to MHR suggesting a universal resistance mechanism in wild grasses.^13^ However, the specific molecular mechanisms of how *Am*GSTF1 leads to MHR remains elusive. Recombinant *Am*GSTF1 has been shown to exhibit both glutathione-*S*-transferase activity toward xenobiotics, as well as acting as a GPOX, reducing fatty-acid hydroperoxides to less reactive monohydroxy derivatives. While both cytoprotective activities may contribute to MHR mechanisms, recent studies indicate that this enzyme is likely to have additional regulatory functions. Previous research showed that transgenic *Arabidopsis thaliana* plants over-expressing *Am*GSTF1 were more resilient to a range of herbicide chemistries and that this was linked to an up-regulation of enzymes involved in oxidative stress responses, an increase in glutathione content and the accumulation of specific flavonoids and anthocyanins.^9^ Related studies also identified intriguing similarities between the functioning of *Am*GSTF1 in MHR in weeds and the distantly related Pi (P) class *Hs*GSTP1 in multiple drug resistance in human cancer cells.^14^ For example, *Hs*GSTP1 is highly expressed in drug-resistant cancer cells and exhibits cytoprotective activities against a range of xenobiotic compounds. Further parallels between *Am*GSTF1 and *Hs*GSTP1 can be made, notably the resistance phenotype linked to the respective proteins could be reversed in both cases by the alkylation of the respective enzymes with 4-chloro-7-nitrobenzofurazan (NBD-Cl).^15^

In this study, using a combination of chemical and structural biology, we demonstrate how NBD-Cl disrupts *Am*GSTF1 function and exerts its role in MHR in plants. Expanding on the use of selective chemical intervention to disrupt MHR, we have then screened for further inhibitors of *Am*GSTF1 and demonstrated that flavonoids are effective ligands that disrupt the functions of the protein in conferring resistance. Using this understanding we have designed a new series of potential herbicide synergists inspired by natural flavonoid chemistry that can reverse the MHR phenotype in black-grass.

## Results

### Crystal structure of wild-type AmGSTF1

**Fig. 1:**
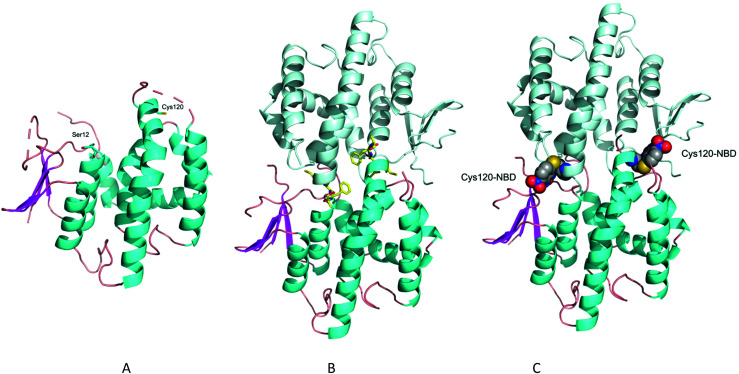
Crystal structure of *Am*GSTF1. (A) Ribbon diagram of the *Am*GSTF1 monomer in the hexagonal crystal form. The N-terminal domain with its β-sheets is shown in magenta on the left-hand side and the C-terminal domain with its α-helices in cyan on the right. Less structured loops are shown in grey with the active site located at the top of the structure. The active site serine is depicted in ball-and-stick representation (B) ribbon representation of the crystal packing of *Am*GSTF1 with the last three residues of the loop (Cys120, Leu121 and Phe122, respectively) shown in a yellow stick-representation. The symmetry-mate in light cyan shows how the loop region of the mate interacts with the active site and hence blocks the access. (C) Ribbon representation of *Am*GSTF1 with the covalently modified Cys120-NBD shown in a CPK representation.

To explore how *Am*GSTF1 can be regulated by chemical inhibitors, a series of crystal structures of the native protein was determined. The first two crystal modifications obtained, hexagonal and rhombohedral, diffracted to 1.53 Å and 1.95 Å resolution, respectively (Table S1a‡). The hexagonal modification contained one protein chain in the asymmetric unit, whereas the rhombohedral form had six independent molecules with a very similar packing arrangement of trimers composed of the functional GST dimer. All polypeptide chains adopted virtually identical overall structures, with rmsds ranging from 0.2–0.4 Å on Cα-atoms. The following description is therefore based on the highest resolution structure in the hexagonal crystal form. *Am*GSTF1 adopts the classical glutathione-*S*-transferase fold,^16^ with the N-terminal domain containing the putative catalytic residue (Ser12) and being composed of four-stranded antiparallel β-sheets, with two α-helices (Fig. 1A). The loop region (residues 38–48), which is associated with glutathione-binding (G-site), showed no electron density, is presumably disordered and as a result, was not included in this model. The C-terminal domain responsible for providing the bulk of the hydrophobic substrate-binding (H-site), consists of six α-helices and a disordered peptide loop (residues 125–137). In both crystal modifications, the four residues preceding this loop, (Cys120-Leu121-Phe122-Asn123), were found to project into the monomer subunit of a symmetry-related chain, effectively blocking the active site of the adjacent subunit (Fig. 1B). This arrangement explains why no ligands were found to be bound in either the G- or the H-site of this open-loop apo-structure, with both loops being flexible and presumably closing upon ligand binding. The closed loop conformation was observed in the recent independent crystal structure determination of *Am*GSTF1 in a different crystal form.^17^ As described in more detail below, a similar closed conformation was subsequently confirmed in the *Am*GSTF1 variant structures and with native *Am*GSTF1 bound to *S*-glutathionylated-NBD in a third crystal form. The cores of all of these structures superimpose very well, with an rmsd of approximately 0.4 Å (Fig. S1A‡). These results confirm a highly conserved core and exemplify the flexibility of the two binding loops including their role in closure upon substrate binding.

### Alkylation with 4-chloro-7-nitrobenzofurazan leads to inhibition of *Am*GSTF1 function

Due to the phenotypic parallels of reversing drug/herbicide resistance through the inhibition of *Am*GSTF1 and *Hs*GSTP1 by 4-chloro-7-nitrobenzofurazan (NBD-Cl), the two protein structures were compared noting several important similarities. Despite showing only 27% sequence identity (Fig. S2‡), the overall architecture of the human and plant GSTs is surprisingly similar. Thus, the two structures could be superimposed with an rmsd of 3.5 Å, with the largest differences resulting from the motion of the two domains with respect to each other and the flexibility of the two binding loops (Fig. S1b‡). These structural parallels are of potential significance, as both proteins were selectively inhibited following treatment with NBD-Cl. Importantly, just as treatment of MHR black-grass plants with NBD-Cl reversed resistance toward three herbicide chemistries,^9^ this alkylating agent also disrupts the drug resistance function of *Hs*GSTP1 in human tumour cells.^14^ In both cases, the inhibitory activity of NBD-Cl was linked to the alkylation of specific cysteine residues, namely Cys47 in *Hs*GSTP1 and Cys120 in *Am*GSTF1, which are respectively located on opposite sides of the binding cleft in different loop regions of each protein (Fig. S1b‡). To determine the structural basis of this covalent modification, *Am*GSTF1 was quantitatively alkylated with NBD-Cl as confirmed by mass spectrometric analysis prior to crystallisation (data not shown). The modified protein crystallised in the same hexagonal space group as the native form, diffracting to 2.8 Å, revealing a conformation that is almost identical in structure to the apo-protein, with rmsd of 0.2 Å for 188 Cα-atoms. The Cys120-NBD adduct was clearly visible in the unbiased electron density (Fig. S3‡), even though the density and the refinement indicated only partial occupancy in the crystal. Although not directly associated with the active site, Cys120 is one of the last residues visible prior to the disordered loop projecting into the active site of the neighbouring polypeptide subunit. The alkylated residue points into the H-site of the symmetry-related protein (Fig. 1C).

To unravel the roles of Cys120 and the catalytic residue Ser12 in controlling *Am*GSTF1 activity, the two amino acids were substituted by site directed mutations, namely Cys120Val and Ser12Ala. The respective recombinant variant proteins were expressed in *E. coli* and assayed for activity against a series of classical GST substrates. In comparison to native *Am*GSTF1, the Ser12Ala (S12A) mutant enzyme showed greatly reduced activity towards all substrates, confirming its key-role as a catalytic residue. The Cys120Val (C120V) variant on the other hand was much less affected, with the exception of its enhanced GPOX activity toward cumene hydroperoxide (Table S2‡). The Cys120Val variant was far more resistant to inhibition by NBD-Cl relative to the native *Am*GSTF1, with the inhibition being independent of exposure time. However, with native *Am*GSTF1, a secondary time dependent loss of activity was observed in the presence of NBD-Cl and glutathione (Fig. S4‡), suggesting there was a secondary mechanism of inhibition at work. It was concluded that this was most likely due to competitive inhibition at the active-site by NBD-Cl, or more likely, the respective NBD-glutathione (GS-NBD) conjugate formed non-enzymatically (Table S3‡). To verify this hypothesis, GS-NBD was synthesized, binding to *Am*GSTF1 confirmed by isothermal titration calorimetry (Fig. S5‡), and shown to afford similar levels of inhibition as observed with NBD-Cl alone (Table S3‡). Collectively, these results supported the observations from the crystal structure determination that Ser12 is the catalytically critical residue and that Cys120 influences ligand binding and catalytic activity even though not located at the active site.

### Screening for *Am*GSTF1 inhibitors identifies flavonoids as binding partners

Having identified the mechanism of NBD-Cl as an inhibitor of *Am*GSTF1, it was then of interest to screen for other less toxic compounds that could serve as leads as herbicide synergists in MHR wild grasses. Drawing parallels with reported roles for members of the GST superfamily involved in drug resistance in animals,^18^ we hypothesised that the regulatory role of *Am*GSTF1 in MHR could be linked to the protein's ability to bind and regulate the biological availability of herbicides.^19^ To test this hypothesis, a range of selective herbicides used to control black-grass were tested for their ability to bind to the protein as determined by thermal shift assay (Fig. S6‡).^20^ When assessed at a final concentration of 100 μM, none of the herbicides tested were found to bind tightly suggesting that it was extremely unlikely that *Am*GSTF1 acts as a herbicide-binding protein.

**Fig. 2:**
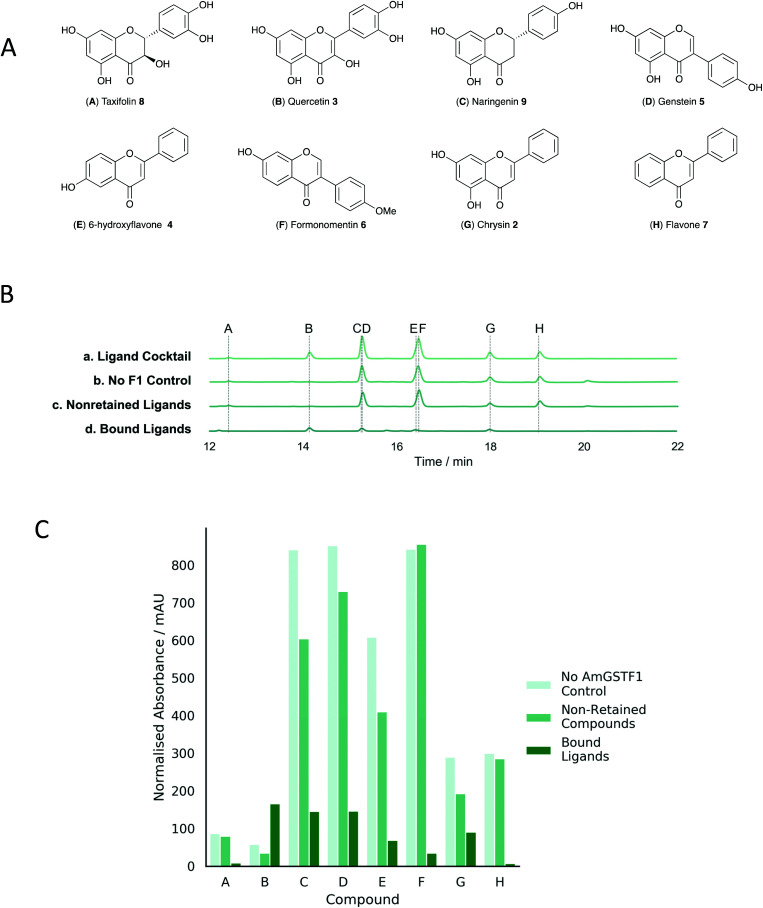
Ligand fishing experiment with representative flavonoids using strep-tagged *Am*GSTF1 immobilized on a streptactin column. (A). Molecular structures of flavonoids used in the ligand cocktail. (B) HPLC trace showing results from the ligand-fishing experiment: a. ligand cocktail – each flavonoid at a concentration of 100 μM; b. no F1 control – ligands retained by blank streptactin column lacking *Am*GSTF1; c. nonretained ligands – non-binding ligand mixture isolated following equilibration and washing; d. bound ligands – mixture isolated following equilibration, washing to remove non-binding ligands and denaturing of protein to elute most strongly retained compounds. (C) Integrated normalized areas of each peak in experiments b to d.

To look for other potential binding ligands, *Am*GSTF1 was recombinantly expressed and purified as an N-terminal streptactin-tag fusion protein and then immobilised on a StrepTactin affinity column. The column was incubated with a cocktail of small molecules including a series of benzofurazans and purines that had previously been shown to be inhibitors of the enzyme^21,22^ (Fig. S7A‡). As part of this cocktail, the model flavonoid apigenin **1** was included, as elevated levels of derivatives of this flavone had been consistently associated with the elevated expression of *Am*GSTF1 in MHR black-grass as compared with herbicide sensitive plants.^44^ After recovery of the protein from the column, the bound ligands were identified by mass spectrometry and the inhibitory activity of each ligand tested using authentic standards by monitoring the *Am*GSTF1-catalysed glutathionylation of chlorodinitrobenzene (CDNB). These ligand-fishing results showed that *Am*GSTF1 selectively retained and bound the flavonoid in favour of the other heterocyclic ligands in the cocktail (Fig. S7B‡). The biological relevance of this observation was then confirmed by undertaking a second ligand-fishing experiment, in which the immobilised protein was exposed to crude extracts from young black-grass plants. These studies revealed the selective binding of a single flavonoid metabolite, tentatively identified by MS-MS analysis as a glucosylated derivative of apigenin (Fig. S8‡).^23^ To explore this link between *Am*GSTF1 and flavonoids, a series of related derivatives was assayed in a similar fashion, with all compounds tested showing evidence of binding. Ligand binding was seen to be independent of the presence of GSH as co-substrate pointing to the H-site as the likely primary binding site (Fig. 2). To further examine the mechanistic basis of these interactions, the degree of inhibition of *Am*GSTF1-mediated CDNB conjugation to glutathione was determined with a range of flavonoid chemistries. These experiments revealed that flavones afforded higher levels of *Am*GSTF1 inhibition than isoflavones (Table S4,‡ entries 3, 5 and 6), with the presence of the 2,3-double bond and C-4 carbonyl group both contributing to binding activity. This result was further confirmed when catechin **10**, lacking both elements, was used and showed no inhibition of CDNB conjugation by *Am*GSTF1 (Table S4,‡ entry 9). Importantly, preliminary spray trial experiments with apigenin **1** showed a clear synergistic effect of flavonoids counteracting MHR in black-grass plants exposed to the herbicide pinoxaden, with no adverse phytotoxic effect determined in wheat as the companion crop plant (Fig. S9‡).

### Enhanced inhibition of *Am*GSTF1 by synthetic flavonoids

**Fig. 3:**
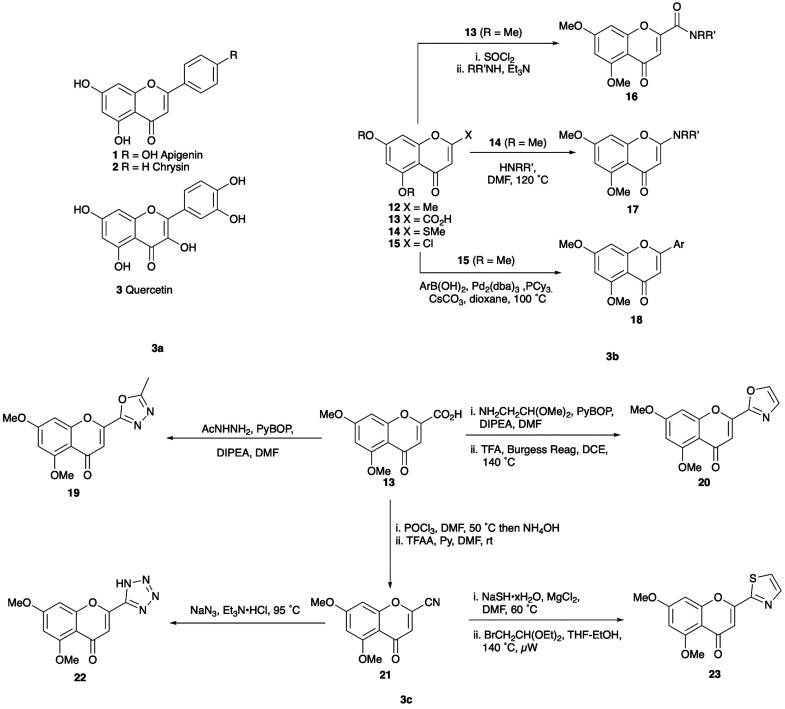
Chemical structures (**3a**) molecular structures of apigenin **1**, chrysin **2** and quercetin **3**; (**3b**) synthetic routes to 2-substituted flavonoid derivatives **16–18**; (**3c**) synthetic routes to 2-heterocyclic substituted flavonoid derivatives **19–23**.

Having established flavonoids as *Am*GSTF1 ligands with the potential to reverse the MHR phenotype, the scope for optimising their activity, selectivity, and physicochemical profile using synthetic modifications was explored. Using 5,7-dihydroxyflavone (chrysin, **2**) as precursor (Fig. 3a), the synthesis of flavonoid derivatives with simple A-ring modifications were undertaken. Each compound was then assessed for its binding to the protein target using both thermal shift assays and by the inhibition of enzyme activity toward CDNB (Table S4‡). The 2-methylchromen-4-one derivative **12** (Table S4;‡ entry 11) was synthesised to assess the effect of replacement of the aryl ring at the 2-position (B-ring) with a smaller group. However, this compound showed reduced inhibitory activity toward *Am*GSTF1, even at relatively high concentrations, suggesting some bulk in this position was essential. Alternative 2-substituents, including amides **16**, cyclic amines **17** and (hetero)arenes **18**, each introduced from the corresponding carboxylic acid **13**, thioether **14** and chloride **15** using standard acylation, S_N_Ar and Pd-catalysed cross-coupling reactions (Fig. 3b). The binding and enzyme inhibition data of these compounds revealed that whilst a broad range of substituents could be tolerated at the C-2 position, higher inhibitory activity was linked to aromatic substituents. However, for these compounds, the assessment of activity was difficult due to their low solubility in aqueous solutions. To increase solubility, a set of heterocyclic derivatives were prepared using standard protocols from the corresponding carboxylic acid **13** (Fig. 3c). While the majority of the 2-heteroaryl derivatives explored showed improved solubility, the enhancement was modest with the exception of the thiazole **23**. Although it is not obvious why this should be the case, this increased solubility correlated with the much lower melting point of the thiazole when compared with most of the other heterocyclic derivatives. Variations explored at C-7 with amines, ethers and esters (Table S4;‡ entries 40–52) revealed little in the way of enhanced inhibitory activity, although the introduction of a PEG unit at this location was beneficial in terms of solubility. Whilst most substituents introduced at O-5 lead to a loss of activity, this was regained in the presence of an α-carboxylate group. Somewhat surprisingly, activity increased with increasing chain length of this substituent, plateauing beyond C-10. Attempts to introduce additional bulk, or conformation restriction, into this element through the introduction of alternative components (*eg* amides, ethers, ring systems) were detrimental to enzyme inhibition. To confirm if the binding observed with these synthetic analogues was specific to *Am*GSTF1, as determined by the inhibition of CDNB activity, the effect of this flavonoid series on an alternative well described phi class GST from *A. thaliana* (*At*GSTF8), was investigated. Importantly, *At*GSTF8 which shares a sequence identity of 42% with *Am*GSTF1 shows no link to herbicide resistance.^24^ Most of the flavonoid analogues prepared showed similar levels of inhibitory activity toward the two enzymes, but again the incorporation of the α-alkoxycarboxylate unit at C-5 led to a selective increased affinity for *Am*GSTF1, with the selectivity toward the black-grass, relative to the *A. thaliana* enzyme further enhanced with increasing chain length (Fig. S10‡).

**Fig. 4:**
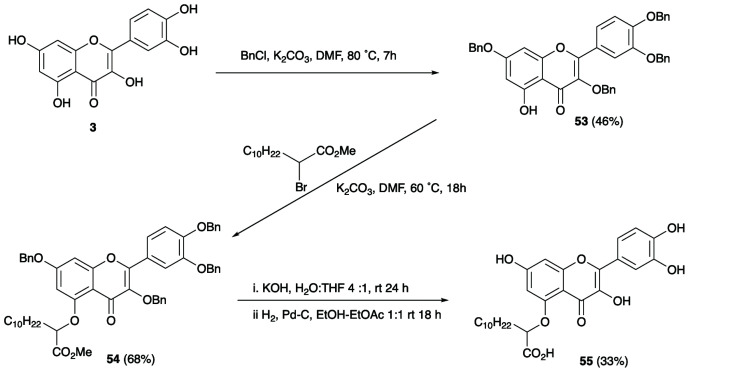
Synthetic route to optimised quercetin derivative **55**.

However, one undesired consequence of these longer chain derivatives was their lower aqueous solubility even for the thiazole derivative. This was partly overcome by using more polar flavonoids as scaffolds. In this respect, the polyhydroxylated flavonol quercetin **3**, which showed good binding activity and high solubility, proved to be a promising lead compound. Combining the quercetin nucleus with a C-5 long chain α-hydroxycarboxylate provided compound **55** (Fig. 4), which demonstrated excellent inhibitory activity in the CDNB assay, good selectivity toward *Am*GSTF1 as compared to *At*GSTF8 and was sufficient soluble to be explored in small scale plant spray trials.

**Fig. 5:**
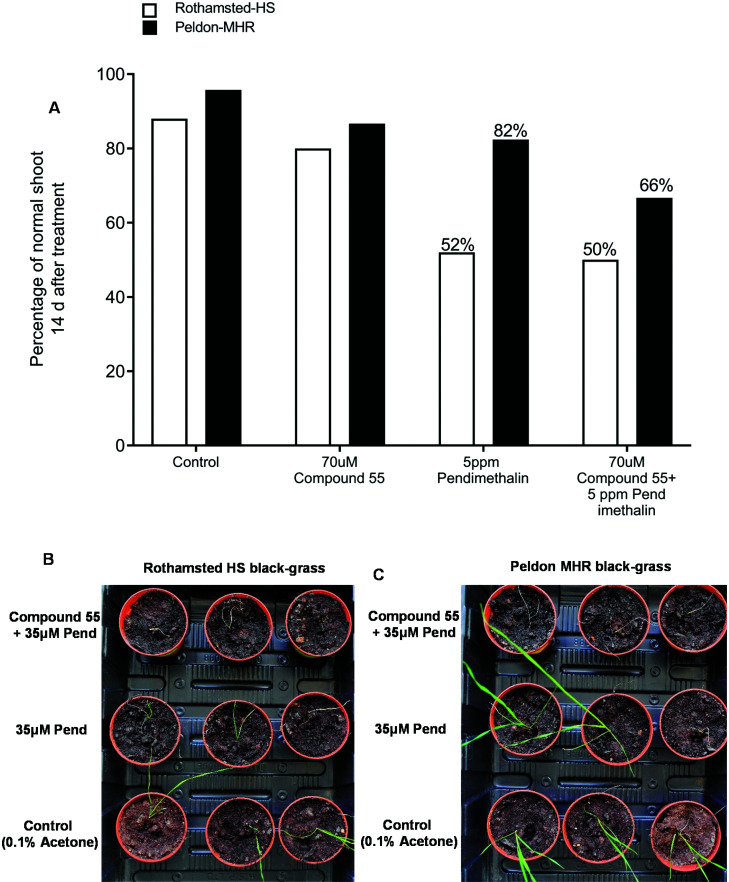
The application of the synthetic flavonoid **55** reverses the resistance to pendimethalin in MHR black-grass. (A) The percentage of normal shoot growth in HS (Rothamsted) and MHR black-grass (Peldon) at 14 d after pendimethalin treatment in petri-dish assay. A pre-treatment with compound **55** for 24 h prior to an application of pendimethalin reduced the percentage of normal shoot growth in MHR black-grass (Peldon). (B–C) A pre-application of compound **55** for 24 h prior to spray treatment of pendimethalin effectively inhibited the growth of MHR and HS black-grass. Plant images were taken at 14 d after pendimethalin application. Plants treated with 0.1% acetone were used as control in petri-dish and spray assay.

To verify flavonoid compound **55** as a herbicide synergist, herbicide sensitive (HS) and MHR black-grass seedlings were treated in petri-dish assays with 70 μM of compound **55**, 24 h prior to an application of 5 ppm pendimethalin, a tubulin assembly inhibitor which is utilised as a classic test herbicide for NTSR.^25^ Resistance to pendimethalin in MHR seedlings pre-treated with **55** was reduced compared to untreated seedlings (Fig. 5).^8^ The impact of compound **55** on resistance to pendimethalin in established plants was further confirmed by treating three-leaf stage (BBCH 13) MHR black-grass plants with 70 μM compound **55**, 24 h prior to exposure to a spray treatment with 35 μM pendimethalin. As with the seedlings, prior treatment with this compound reversed pendimethalin resistance in the MHR black-grass plants (Fig. 5). Collectively, these results demonstrated that compound **55** can partially reverse MHR in black-grass.

### Crystal structures of Tyr118Ser and Phe122Thr variants of *Am*GSTF1

**Fig. 6:**
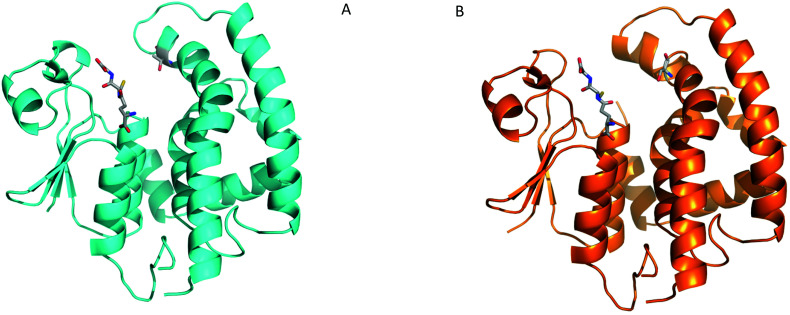
Crystal structures of *Am*GSTF1 variants (A) Ribbon representation of the Phe122Thr *Am*GSTF1 variant (cyan) with the GSH ligand and the mutated residue Thr122 shown in stick representation (B) Tyr118Ser variant (orange) with the GSH ligand and the mutated residue Ser118 shown in stick representation.

To further elucidate the molecular mechanisms of *Am*GSTF1 activities and ligand binding, we then attempted to crystallize the *Am*GSTF1 enzyme in the presence of substrates and ligands. In spite of extensive crystallisation and soaking efforts, no crystal with any substrate bound or any alternative crystal packing forms of the wild-type protein were obtained. Based on a careful analysis of the crystal packing, the key hydrophobic contact stabilising a crystallographic dimer interface shown in Fig. 1b was identified and three protein variants were designed to facilitate alternative packing. The three variants, Tyr118Ser, Phe122Thr, and the double mutation Tyr118Ser/Phe122Thr were expressed in *E. coli* and purified in the similar manner as the recombinant native enzyme. Circular dichroism studies showed that all variants were properly folded, with the same solution structure as the native enzyme albeit with the mutants showing considerably diminished enzyme activity (Fig. S11‡). Crystals of the Phe122Thr variant of *Am*GSTF1 mutant were subsequently obtained in a new, tetragonal modification, and the structure was determined to 2.8 Å resolution (Table S1b‡). The core structure was found to be virtually identical to that of the native protein (Fig. 6a). However, the previously disordered loops in wild-type *Am*GSTF1 were now well-defined in the Phe112Thr variant and, for the first time, a glutathione molecule was determined as being bound in the G-site, confirming the validity of the design strategy. The Phe122Tyr variant therefore adopted the closed conformation for the two loops over the binding cleft with glutathione bound in the G-site. Having identified new crystallisation conditions for the Phe122Thr variant, a similar approach was used in screening for the Tyr118Ser *Am*GSTF1 variant, with the structure determined to a resolution of 2.6 Å (Table S1b‡). The protein was found to adopt a typical GST fold (Fig. 6b), with density again observed for the loop over the G site the resulting structure being identical to that of the native enzyme and the Phe122Thr variant presented here, respectively (Fig. S12‡). Density was observed again associated with a glutathione molecule in the binding site, although this was refined with a reduced occupancy of 0.7. Interestingly, the Tyr118Ser mutation resulted in the top of the α4 helix becoming disordered, with the weak density observed for residues from Phe122 to Thr131 consistent with considerable flexibility. This suggests that interactions formed by this Tyr118 residue are essential to the structure of this helix and may explain the reduced enzymatic activity associated with the mutation of this residue.

### *Am*GSTF1 binding to GSH-NBD

**Fig. 7:**
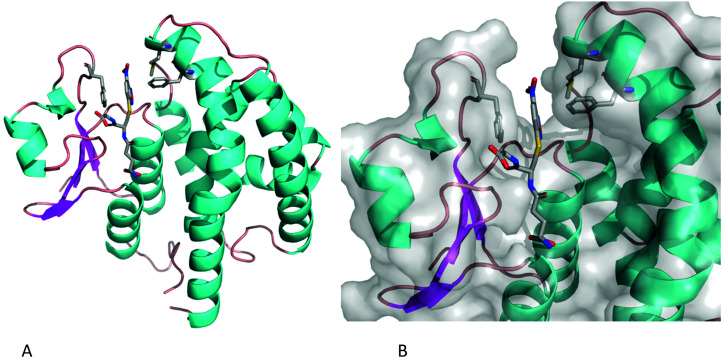
(A) Ribbon representation of wild-type *Am*GSTF1 in the tetragonal crystal form with the ligand GS-NBD and key residues forming the hydrophobic channel depicted in a stick representation (B) Surface representation of the close-up of the GS-NBD binding site in the crystal structure of wild-type *Am*GSTF1.

To investigate the native *Am*GSTF1 active site in more detail, a cross-seeding approach was utilised whereby microseeds of the Phe122Thr variant crystals were used to obtain well-diffracting wild-type crystals in the tetragonal modification suitable for ligand soaking experiments.^26^ Following soaking with the glutathione-NBD conjugate, the crystals were found to diffract to 2.3 Å (Table S1b‡). As with the Ph122Thr variant GSH-bound structure, crystals were in a tetragonal modification, in space group *I*4_1_22. The overall structure was virtually identical to that of the independently solved structure,^17^ as well as to that of the Phe122Tyr mutant (Fig. 7a). The NBD-GS molecule was seen to bind across both the G and H sites (Fig. 7b), with the GSH portion of the molecule adopting the same conformation as the unconjugated GSH molecule seen in previous structures and the NBD moiety extending into the upper portion of the H-site. The aromatic portion of the molecule is seen to form a strong π-stacking interaction with Phe36, with its side-chain clearly rotated from its location in the apo-structure. The hydrophobic side chains of Phe122 and Met126 complete a narrow hydrophobic channel perfectly suited to bind the NBD moiety. These hydrophobic interactions help explain how the GS-NBD adduct acts as a strong competitive inhibitor following the conjugation of NBD-Cl.

### Flavonoid binding by molecular modelling

**Fig. 8:**
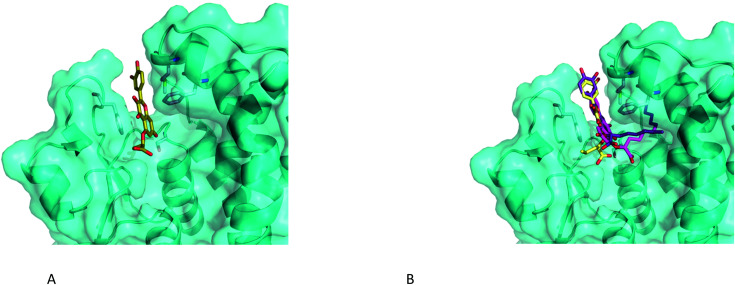
Close-up of the binding poses of 5-alkoxycarboxylate analogues of **55** with increasing chain length docked into the binding site of *Am*GSTF using GOLD (A) *n* = 1, (B) *n* = 3 (yellow), *n* = 6 (violet) and *n* = 10 (**55**) shown in magenta.

Due to their low solubility, the flavonoid derivatives proved to be unsuitable for soaking experiments. Therefore, we used the now well-defined active site structure of *Am*GSTF1 as a starting point for molecular docking using GOLD.^27^ Using analogues of **55** with a range of aliphatic chain lengths, compounds were docked into the binding site. In all cases, the respective aromatic ring system occupied the same space between the two closing loops as the ring system of GS-NBF (Fig. 8a). This hydrophobic cleft is defined by the side chains of residues Phe36 on one side, and Met126, Tyr178 and most notably Phe122 on the other side. This binding pose further supports our observation that the Phe122Thr variant exhibits reduced binding. Notably, the C-7 oxygen substituent is in a perfect position to form a hydrogen bond of 2.9 Å to the OH-group of the catalytic Ser12 at the bottom of the active site. The carboxylate group points towards the canonical G-site, which also provides space for an increasing aliphatic chain (Fig. 8b). Further increases in the chain length (*n* > 6) lead to a different binding pose, where the aliphatic chain occupies a narrow channel further increasing contacts and hence binding affinity up to *n* = 10 (Fig. 7b). Overall the docking poses are consistent with the observation of enhanced inhibitory activity with increased chain length.

## Discussion

Plant GSTs are a superfamily of enzymes of great interest in agriculture due to their roles in herbicide selectivity and tolerance to biotic and abiotic stress.^9,28^ Protective activities toward chemically-imposed stress demonstrated by GSTs in crops and weeds include their ability to use the tripeptide glutathione (GSH) to both conjugate and detoxify xenobiotics and to act as a GPOX, reducing toxic organic hydroperoxides to the corresponding alcohols. GSTs have also been linked to a diverse range of other functions, including signalling, counteracting oxidative stress, and detoxifying and transporting secondary metabolites. In this last role, a range of biologically-active plant secondary metabolites including fatty acids, auxins and phenolics are known to be GST ligands, with binding occurring either in the hydrophobic domain of the active site, or at distinct ligandin binding sites. The Phi (F) class GSTs, of which *Am*GSTF1 is an example, are found in all higher plants, being homodimeric enzymes in which each protomer of approximately 23–30 kDa with the catalytic serine residues located at the interface. Each protomer has two distinct domains; the N-terminal domain adopts the typical thioredoxin-fold, with the C-terminal domain being a bundle of at least five helices as demonstrated by the crystal structures of *Am*GSTF1 presented here. To gain additional insight into the potential functions of *Am*GSTF1, it is interesting to compare its structure to that of other plant GSTs involved in resistance to chemicals. The most closely related structure to *Am*GSTF1 is that of the phi class maize *Zm*GSTF1 (PDB code 1AXD),^29,30^ a protein known to be involved in herbicide tolerance in this major crop.^19^ While the two orthologues share a sequence identity of only 63%, the two structures were found to superimpose very well, with an rmsd of 1.3 Å for 182 equivalent Cα-atoms. In addition, the residues lining both the GSH and hydrophobic binding domains of the active sites are highly conserved. Superimposing the crystal structures of the maize and black-grass proteins showed that the greatest differences in the two GSTFs are the two disordered loops in the *Am*GSTF1 structure, and the areas immediately surrounding them (residues 38–48 and residues 124–138) (Fig. S1C‡). In the *Zm*GSTF1 structure, the loop over the G site (residues 38–48) is known to change structure upon substrate binding, resulting in an induced fit to the substrate as the loop closes over the binding site.^30^

Significant similarity between *Am*GSTF1 and the human pi class GST, *Hs*GSTP1 linked to multiple drug resistance was also investigated. Whilst differing greatly in primary sequence, the tertiary structure of the two GSTs was surprisingly similar (Fig. S1B‡). Both enzymes possess a solvent-exposed cysteine residue located close to, but on opposite sides of the substrate binding pocket, which on alkylation with NBD-Cl leads to a corresponding inhibition of GST function. The covalent modification of cysteines located in the binding loop clearly affect enzymatic activity presumably due to changes in dynamic behaviour. Interestingly, the GS-NBD conjugate in *Am*GSTF1 is seen to occupy a similar position to that observed for the related inhibitor 6-(7-nitro-2,1,3-benzoxadiazol-4-ylthio)hexanol (NBDHEX) and its respective GSH conjugate, as determined in the active site of *Hs*GSTP1.^14,21,31^ In both cases, the inhibitor ligands are seen to form interactions both with residues in the α4 helix and the loop over the G-site. In *Hs*GSTP1, these ligands are thought to stabilise GSH binding in the active site by preventing its conjugation with natural substrate molecules and subsequent dissociation. It is likely that the NBD derivatives play similar roles in *Am*GSTF1. Collectively, a combination of structural biology and kinetic analyses of the native and mutant enzymes suggests that the protein exerts its activity through binding regulatory ligands within, or close to, the active site. However, while the black-grass and human GSTs share the same overall structure, the residues lining the hydrophobic pocket in the active site are not conserved, suggesting the two proteins bind different ligands. For example, the hydrophobic binding pocket residues Tyr7, Phe8, Pro9 and Val10, in the *Hs*GSTP1 structure, were substituted with the residues Gly-Pro-Ala-Met in the *Am*GSTF1 sequence. In contrast, the residues responsible for inhibitor recognition in the *Hs*GSTP1 structure (Ile104 and Tyr108), were semi-conserved with the equivalent residues (Leu, Phe) in *Am*GSTF1.

A range of biologically active secondary metabolites are known to be selectively bound by plant GSTs, with disruption in the expression of specific phi and tau family members altering the accumulation of anthocyanin derivatives in a range of plant species.^28^ Previous studies have also shown that *Am*GSTF1 selectively binds flavonoids, such as hydroxylated isorhamnetin.^8^ Consistent with these observations, the ligand fishing experiments presented here revealed a variety of flavonoid structures to be potent binders to *Am*GSTF1. In many hyphenated techniques, including HPLC-ESI-MS^32–34^ and kinetic capillary electrophoresis MS,^35^ the detection of ligand binding is sensitive to the concentration of the analytes, with more abundant compounds, but not necessarily more active binding agents dominating selection. In contrast, the ligand fishing technology employed here is more suitable for the identification of relatively low abundant ligands. Although most commonly undertaken with proteins immobilised on magnetic nanoparticles,^36^ in this study we opted to use *Am*GSTF1 bound to conventional columns as this enabled easier integration with LC-MS analysis. As such, this approach is a variation of the previously used frontal affinity chromatographic methods^37^ with the advantage that smaller quantities of analytes are required which is particularly critical for the identification of lower-affinity ligands. The use of ligand concentrations far in excess of the protein and low flow rates ensures equilibrium between free and bound ligands, and therefore, that compounds of greater affinity should outcompete weaker binders.

The identification of flavonoids as potent binders to *Am*GSTF1 is not without precedent.^38^ As ubiquitous natural products in plants, flavonoids are known to exhibit a wide range of protective biological activities, functioning as antioxidants, UV-protectants and antimicrobial agents.^39^ Within the pharmaceutical domain, flavonoids have been shown to modulate key metabolic enzymes and drug transporters. For example, in humans, flavonoids inhibit the CYP enzymes responsible for the bioactivation of xenobiotics and upregulate the enzymes involved in subsequent phase II metabolism detoxification such as the UDP-glucuronosyltransferases and GSTs.^40^ Moreover, drawing further parallels between MHR and other MDR phenomena, flavonoids have been reported to inhibit various enzymes involved in drug detoxification notably ATP-binding cassette (ABC) transporters including P-glycoprotein-1 (PLP), Breast Cancer Resistance Protein (BCRP) and Multi-drug resistance protein (MRP) in various cancer cell lines, with specific MRP isoforms in turn known to be involved in transporting flavonoids and glutathione conjugates.^41,42^ It is also well established that certain plant GSTs bind flavonoids as part of a ligandin function, suggesting that GSTs can play a role in flavonoid transport and bioavailability under stress conditions. It is therefore a reasonable assumption that proteins that can selectively perturb flavonoid composition in plants can also influence their protective activities. Our results support this hypothesis showing an unexpected connection between herbicide tolerance and flavonoids, suggesting that the functions of *Am*GSTF1 in MHR can be modulated through flavonoid binding. The natural relevance of this remains to be fully established although there is a growing body of evidence linking flavonoid content to stress tolerance and developmental signalling in plants.^43^ Further studies that shed light on the relationships of *Am*GSTF1, and related proteins with flavonoid levels in MHR in blackgrass and other weeds are currently in progress.

## Conclusions

In conclusion, the roles for *Am*GSTF1 and *Hs*GSTP1 functioning in multiple resistance mechanisms to herbicides in plants and drugs in man are classic examples of parallel evolution drawing on an existing tractable protein scaffold to perform new cellular functions. Our results further demonstrate the multiple roles for GSTs in herbicide tolerance and the potential for developing new chemical strategies for counteracting resistance based on a fundamental understanding of their natural functions. In the case of *Am*GSTF1, structural studies have shown that flavonoid binding occurs within the active site and the models established here represent a starting point for structure-guided inhibitor design. Whilst derivatives of flavonoids that show selectivity for binding to *Am*GSTF1 and can reverse the MHR phenotype can be chemically prepared, this class of synergist is challenged in its adoption for roles in agriculture due to low aqueous solubility. Biosteric structures that mimic this activity with better physicochemical profiles are currently under investigation and will be reported in due course.

## Author contributions

R. F. M. E., M. S., contributed equally to this paper. N. O., R. F. M. E., M. S., S. F. P., D. J. W., H. E. S., C. R. C., M. B. H., I. C. designed and performed experiments, analysed data and drafted reports; D. H., J. M., G. M. gave technical support and conceptual advice; and R. E., P. G. S., and E. P. conceived the study, designed experiments, analysed data and wrote the paper.

## Conflicts of interest

The authors declare no competing interests.

## Supplementary Material

OB-019-D1OB01802G-s001
