# Supplementary material for: Flavonoid-based inhibitors of the Phi-class glutathione transferase from black-grass to combat multiple herbicide resistance
Source: Org Biomol Chem. 2021 Oct 13;19(42):9211–22. doi: 10.1039/d1ob01802g (PMC8564858; doi:10.1039/d1ob01802g)
Supplement: OB-019-D1OB01802G-s001 [file OB-019-D1OB01802G-s001.pdf]

Electronic Supplementary Material (ESI) for Organic & Biomolecular Chemistry.
This journal is © The Royal Society of Chemistry 2021

Flavonoid-based inhibitors of the Phi-class glutathione transferase from black grass to combat multi-herbicide resistance

Maria Schwarz[$1], Rebecca F. M. Eno[$1], Stefanie Freitag-Pohl[1], Christopher R. Coxon[1], Hannah E. Straker[1], David J. Wortley[2], David J. Hughes[3], Glynn Mitchell[3], Jenny Moore[3], Ian Cummins[4&], Nawaporn Onkokesung[5], Melissa Brazier-Hicks[5#], Robert Edwards[5], Ehmke Pohl[1,4*], Patrick G. Steel[1*]

[1]Department of Chemistry, University of Durham, Science Laboratories, South Road, Durham, DH1 3LE, United Kingdom

[2]Centre for Novel Agricultural Products, Department of Biology, University of York, York YO10 5DD, United Kingdom

[3]Syngenta, Jealott's Hill International Research Station, Bracknell, Berks RG42 6EY, United Kingdom

[4]Department of Biosciences, University of Durham, Science Laboratories, South Road, Durham, DH1 3LE, United Kingdom

[5]Agriculture, School of Natural and Environmental Sciences, Newcastle University, Newcastle-upon-Tyne, NE1 7RU, United Kingdom

$ joint first authors

& deceased on June 4th 2019

Materials and Methods

Cloning, expression and purification of wildtype AmGSTF1

Expression of *Am*GSTF1. Binding studies, enzymatic assay and ligand assays were performed using the strep-tagged *Am*GSTF1 described previously [1]. Briefly, the protein was over-expressed in *Escherichia coli* and the over-expression induced with Isopropyl β-D-1-thiogalactopyranoside (IPTG at 100 µM). Purification was carried out in a single step using the Strep-tactin sepharose high-perfomance colum (GE Healthcare). For crystallographic studies *Am*GSTF1 was expressed as the native protein without any affinity tags in *E. coli* (Rosetta) cells. Bacteria transformed with the plasmid were cultured in LB medium (1 L) until the OD_{600} reaches 0.7. The cultures were then induced with 1 mM IPTG, grown overnight and harvested by centrifugation. Bacteria were resuspended in 20 mM HEPES pH 7.6 containing 150 mM NaCl, cOmplete™ (Roche) mini protease inhibitor cocktail, lysed by ultrasonication and pelleted by centrifugation. Based on the original purification protocol [2] the protein was purified in a two-step process. Lysate was incubated with glutathione agarose (Sigma Aldrich) for 1 h at 4 °C, the column washed with 20 mM Tris pH 7.5 containing 200 mM KCl and 1 mM DTT and eluted using 20 mM Tris pH 7.5 containing 5 mM glutathione, 1 mM DTT. Protein was dialysed overnight into 20 mM Tris pH 7.5 followed by purification by anion exchange chromatography with a Pharmacia MonoQ column using 20 mM Tris pH 7.5 and 20 mM Tris pH 7.5 containing 500 mM NaCl. All protein samples were characterised by SDS Page and LC-ESI mass spectrometric analysis.

Cloning, expression and purification of the Cys120Val and Ser12Aala *Am*GSTF1.

The coding sequence of *Am*GSTF1, sub-cloned in the pET 24a vector was used to generate the C120V and S12A mutants by PCR: C120V forward primer 5'-ccgatcgtgtatcaggttctgtttaacccg-3', C120V reverse primer 5'-cgggttaaacagaacctgatacacgatcgg-3', S12A forward primer 5'-ggcccggccatggcaaccaacgttgcacg-3' and S12A reverse primer 5'-cgtgcaacgttggttgccatggccgggcc-3'. Following sequence validation, the amplified products were sub-cloned into the pET-STRP3 vector along with native *Am*GSTF1, *Lr*GSTF1 and *Zm*GSTF1 available from previous studies [2]. After expression in *E. coli* Tuner (DE3) cells using the pRARE plasmid, the tagged recombinant enzymes were purified using *Strep*-tactin affinity chromatography prior to enzyme assay [1]. The recombinant wildtype and mutant enzymes proved to be equally stable with all retaining activity over a 30 day storage at 4 ˚C

Cloning, expression and purification of the Tyr118Ser Phe122Thr *Am*GSTF1.

The Tyr118Ser variant was constructed from the wildtype using standard PCR with the following primers: 5'-CCGATCGTGTCTCAGTGTCTG-3' (forward) and 5'-GCTCAGTGCCGGATTATAG -3 (reverse). The Phe122Thr variant was created from the wild type untagged construct using PCR: forward primer:

5' TCAGTGTCTGACCAACCCGATGA 3', reverse primer 5' TACACGATCGGGCTCAGT 3'. Protein was expressed in *E. coli* as described for the wild type protein. The protein was purified using affinity chromatography with glutathione agarose as for the wild type protein. No additional purification steps were required prior to crystallisation.

Expression and purification of *At*GSTF8

The expression and purification of AtGSTF8 was based on a strep-tagged protein construct in the same plasmids as the *Am*GSTF1 using the same protocols as described before [1].

Crystallisation and structure determination of wild type *Am*GSTF1

Untagged wild type *Am*GSTF1 was used for crystallographic studies. For the NBD-Cl structure *Am*GSTF1 (37 µM) was labelled in the dark for 1 h with NBD-CL (100 µM) and dialysed for 16 h against 20mM Tris pH 7.5. Crystals were obtained using the sitting drop method. For the rhomboedric form crystals were obtained in a condition containing HEPES pH 7.5, ammonium sulfate and PEG 400. For the hexagonal and NBD-Cl crystals were obtained in a condition containing sodium citrate pH 5.6, Na/K tartrate and ammonium sulfate. Data were collected on beamline SLS-X06D[3] for the rhombohedral modification using a Mar Research CCD detector and processed with Denzo/Scalepack [4]. The hexagonal diffraction data were collected on DLS i04-1 using a pixel detector and processed using Xia2 and scaled using AIMLESS/POINTLESS. The NBD-Cl diffraction data set was collected using a Bruker Microstar rotating anode and processed with SAINT. The structure was solved by molecular replacement using Phaser [5] and *Zm*GSTF1 (1AXD) for the rhombohedral crystal form, and against the lower resolution *Am*GSTF1 structures for the hexagonal and NBD-Cl labelled forms. Models were built in CCP4 using Coot [6], and refined with Refmac [7] using local NCS restraints for the rhombohedral modification [8]. Ligand restraints were generated using JLigand [9]. Further experimental detail is summarised in Table S1

Crystallisation and structure determination of Tyr118Ser and Phe122Thr *Am*GSTF1

The Phe122Thr *Am*GSTF1 variant, which was produced was produced in much higher yield, was crystallised first using the sitting drop method in the Morpheus screen, with crystals produced in a number of wells. The same crystallisation conditions were used for the Phe122Thr variant. All diffraction data were collected at the Diamond Light Source. All data was processed using the Xia2 pipeline [10] and the structures were solved using molecular replacement using the wild type structure and Phaser [5].The previously disordered loops were built using Buccaneer [11] and interactive model building with coot. All final models were completed by interactive cycles of model adjustment using

Coot and reciprocal space refinement using Refmac. Further experimental detail is summarised in Table S1

Molecular Modelling

Molecular docking was performed using the GOLD package with CHEMPLP as the target fitness function [12]. The structure of the AmGSTF1 in the tetragonal unit cell with both loops in their closed conformation was used as a starting model for docking. The ligand (GS-NBF) was removed from the binding site. The search space was defined by the position of the ligand in the cavity with a margin of 10 and 15 Å, respectively. The ligands were built with Avogadro [XX]. In each case, the starting geometry minimized using the MMFF94 forcefield [xx]. Each ligand was docked with 30 independent genetic algorithm using the diverse solution settings in order to explore a maximum parameter space.

Isothermal Titration Calorimetry

*Am*GSTF1 was dialysed into HEPES buffer (pH 7.6) overnight at 3 °C and the concentration adjusted to 10 µM in a volume of 3 mL. The activity of the enzyme was calculated prior to use according to the standard CDNB biochemical assay. A solution of the desired ligand (≥ 10 x protein concentration, 200 µL) was prepared in the same HEPES buffer as above. All samples were degassed for 5 minutes prior to use using a thermovac at 24 °C. *Am*GSTF1 was incubated in the cell and the ligand in the syringe, ensuring all air bubbles were excluded from both. The parameters were set as indicated below and the experiment run to completion or until no more titrant was available. The data obtained was analysed using Origin™ software.

Parameter	Value
No. of injections	200
Temperature (°C)	25
Ref Power	10
Initial delay (sec)	60
Stirring Speed	329
Volume/ Duration of 1st Inj	2 µL /4 sec
Volume/ Duration of Inj	8 µL/ 16 sec
Spacing (sec)	150 sec
Filter Period	5

Thermal shift assays

To protein (0.5 mg/mL) was added 20 × SYPRO orange (4 µL/ mL protein). 10 µL of protein (in 20mM HEPES, 150 mM NaCl pH 7.6) was added to each well of a 96 well PCR plate. 10 µL of ligand at varying concentrations (in 20 mM HEPES, 150 mM NaCl, pH 7.6 and 2% DMSO) was also added to the well. The plate was sealed with a PCR seal and centrifuged (1000 rpm, 2 min). Experiments were performed using the Applied Biosystems 7500 Fast Real-Time PCR (RT-PCR) system using Filter C with an excitation of 470 nm and an emission of 569 nm. Samples were run from 24-96 °C with a temperature increase of 1 °C/ min. Once samples had been run results were analysed using NAMI software [13,14].

Biochemical analysis

GST and GPOX assays for activity towards 1-chloro-2,4-dinitrobenzene (CDNB), cumene hydroperoxide, 2-hydroxyethyl disulfide, ethacrynic acid, crotonaldehyde and benzyl isothiocyanate were carried out as described previously [15,16]. For inhibitor treatment studies, enzyme preparation (37 µM) were incubated with 100 µM solutions of inhibitor treatments NBD-CL in the dark for 0-60 min before diluting 1:100 (v/v) and assaying for activity toward CDNB. Enzymes were incubated with an equivalent volume of DMSO as negative controls. Phenolics were analysed and quantified by HPLC linked to mass spectrometry as described [15]

Ligand fishing experiments

Strep tagged protein was expressed and purified as describe before [17] and immobilised on a 1 mL StrepTactin column. Unbound protein was eluted using 20 mM HEPES pH 7.6 and a ligand cocktail containing up to 10 compounds at 100 µM was injected onto the column. Non-retained ligands were eluted with HEPES buffer (flow rate 0.5 ml /min). Protein was eluted using Desthiobiotin (2.5 mM) in HEPES. Recovered protein was concentrated and precipitated by addition of 2 volumes of methanol and cooling on ice for 30 min. Following centrifugation, the supernatant was concentrated in vacuo, re-dissolved in MeOH and analysed by HPLC or Qtof ESI-MS. Bound ligands were compared with the original ligand cocktail, non-binding ligands solution and with a negative-control sample collected by performing the assay using a column containing no protein. Compounds displaying the highest affinity were identified from known HPLC standards and could be quantified from standard curves enabling a ranking of the most potent inhibitors.

Supplementary Figures

Figure S1: Ribbon representations of least-squares superpositions of wildtype *Am*GSTF1 structures (A) *Am*GSTF1 in the hexagonal crystal form in its open conformation reported here (cyan helix, magenta sheets) with *Am*GSTF1 in its closed conformation (PDB:6riv) in yellow (B) *Am*GSTF1 (hexagonal crystal modification) reported here superimposed on *Hs*GSTP1 (PDB:3GSS) in red. The two cysteine residues (Cys120 in *Am*GSTF1 and Cys47 in *Hs*GSTP1, respectively) that are alkylated by NBD-Cl are highlighted in CPK representation (C) Superposition of *Am*GSTF1 in its hexagonal crystal form with *Zm*GSTF1 from Maize (green) (PBV:1AXD).

A

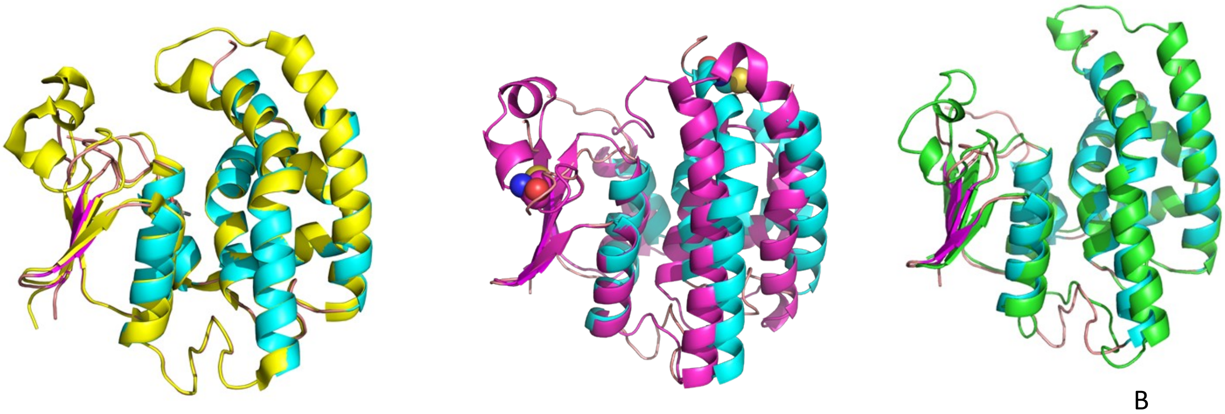

B

C

Figure S2: Sequence alignment of *Am*GSTF1 with orthologues from maize (*Zm*GSTF1) and human (*Hs*GSTF1-1)

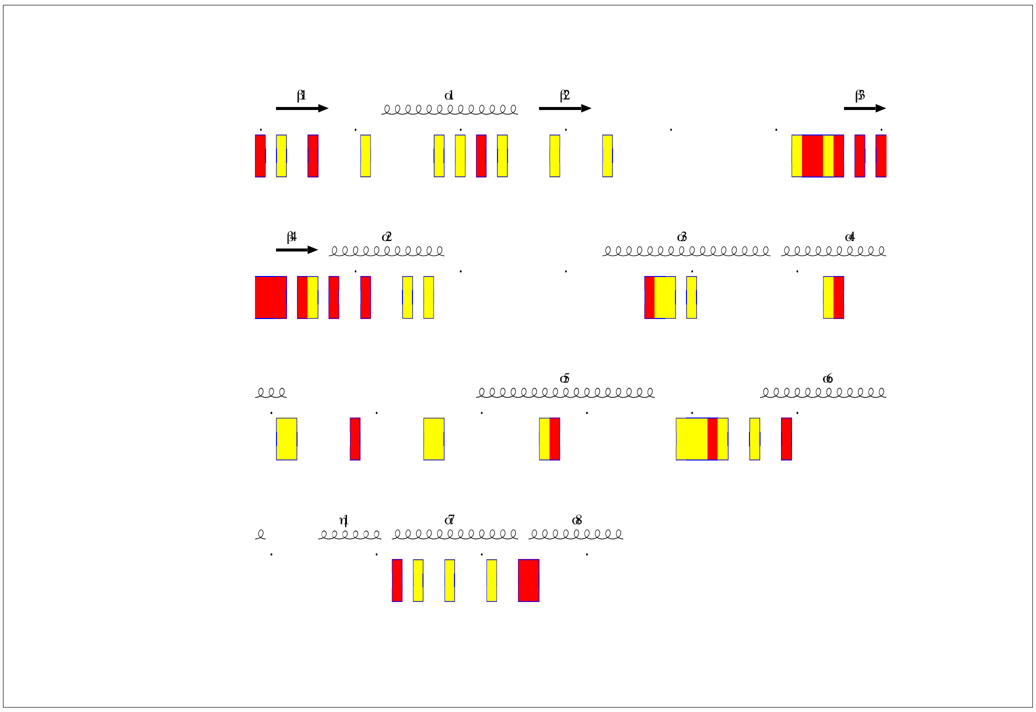

Figure S3: Close-up of active site of apo *Am*GSTF1 covalently modified with CNBD with its open loop conformation and its disordered residues 37-49 and 135-137, respectively. The catalytic residues Ser12 is depicted in stick representation. The symmetry loop residues Ala119'-Cys120'-NBD-Leu121 are shown in stick representation superimposed with the 2Fo-Fc electron density map contoured at 0.7 sigma of the residues. The covalent modification on Cys120' is clearly visible and the symmetry-related loop blocks access to the active site.

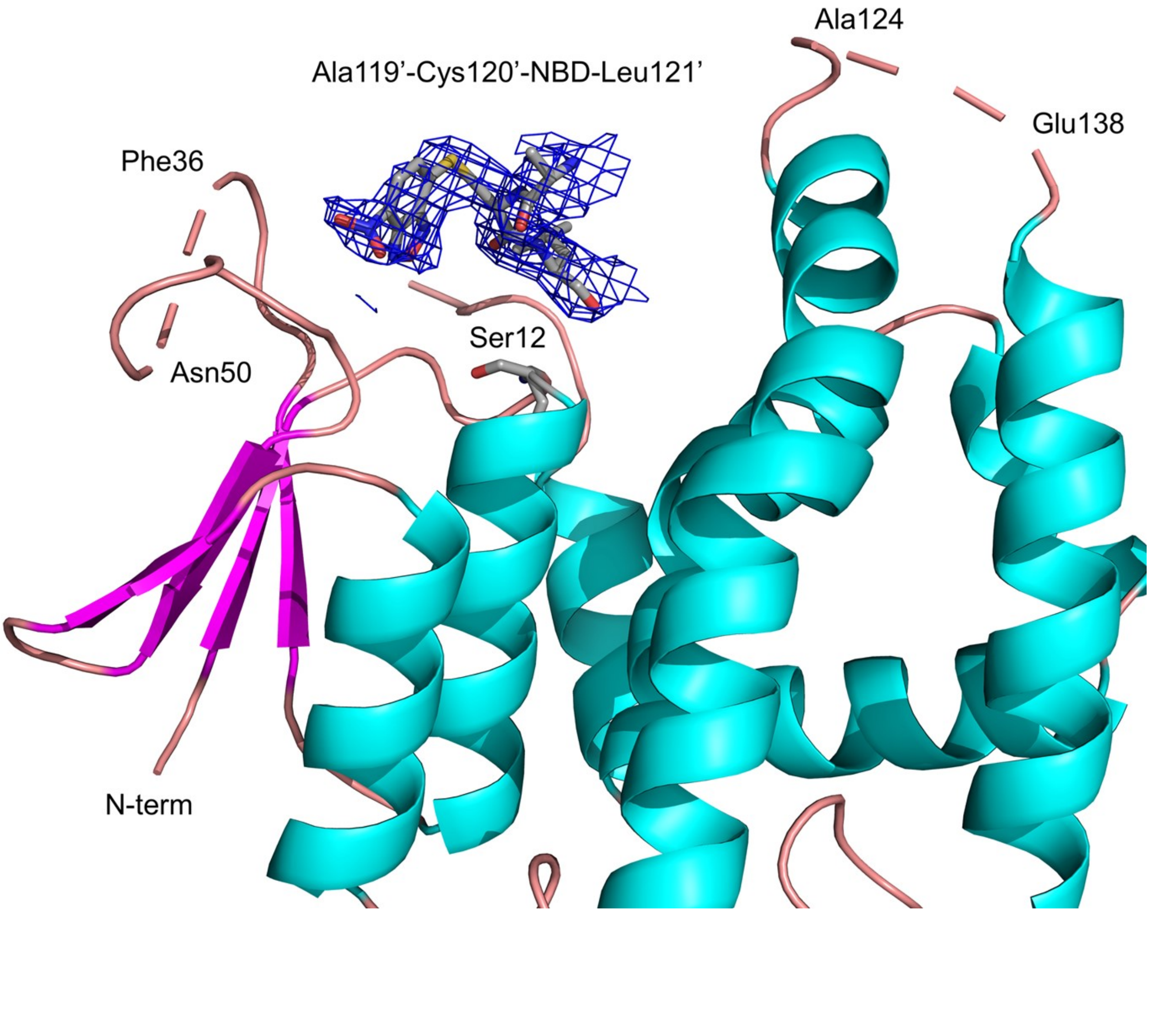

Figure S4. Time-dependent inhibition of *Am*GSTF1 by CNBF. Enzymes were incubated with 100 µM CNBF, or an equivalent volume of DMSO; control, over a period of 60 min before diluting 1:100 (v/v) and assaying for GST activity toward CDNB at time-points. Measurements were performed in triplicate with data points showing means and error bars SDs.

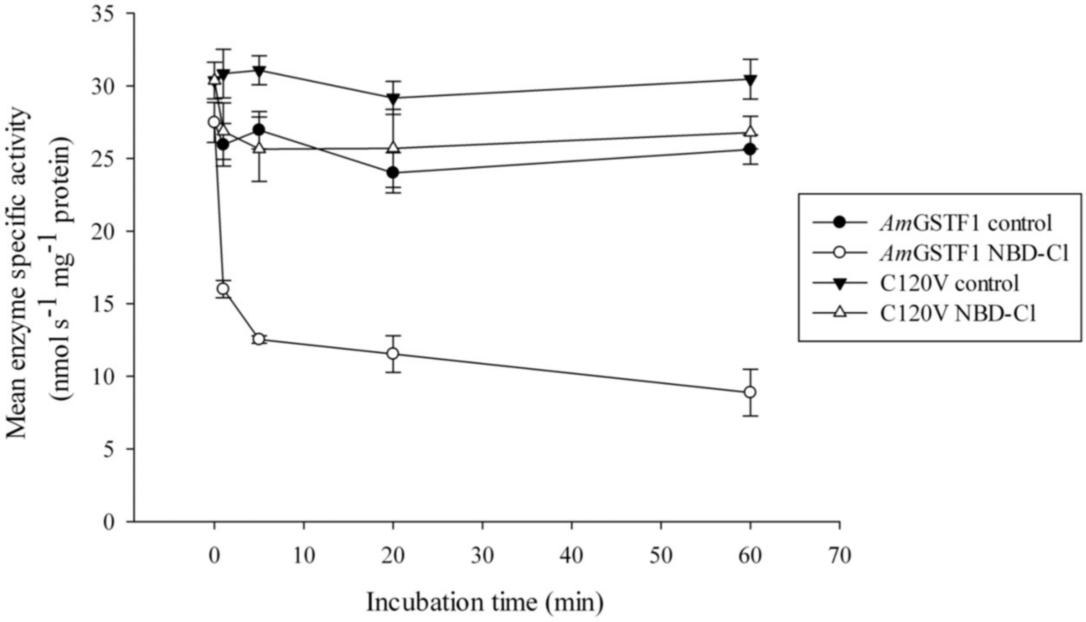

Figure S5: Isothermal titration calorimetry data of *Am*GSTF1 with the GS-NBD adduct showing sub-millimolar binding.

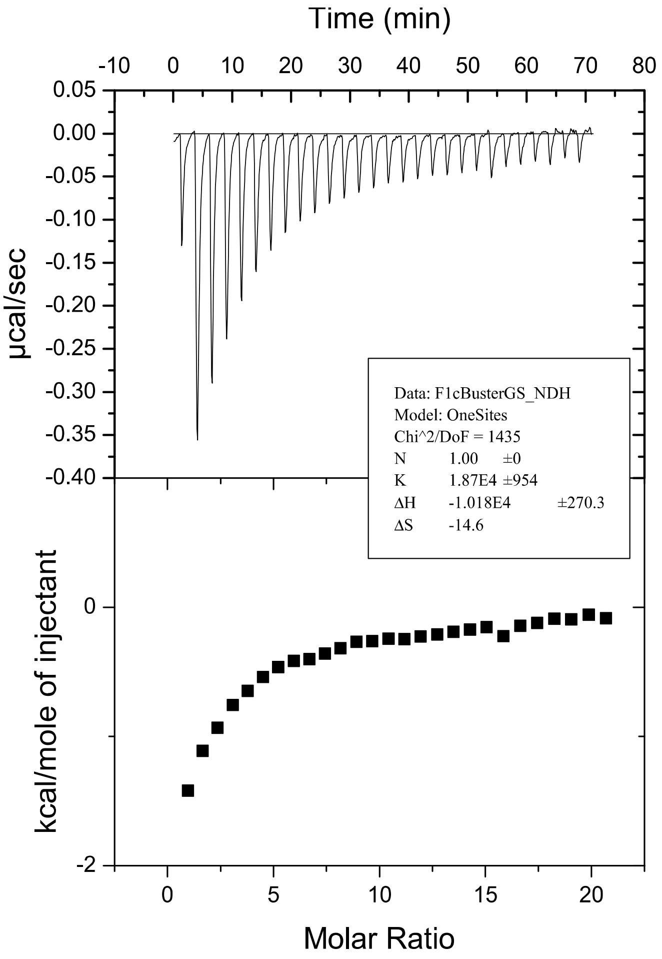

Figure S6: Thermal shift data for herbicide binding to recombinant wild-type *Am*GSTF1. None of the herbicides tested led to a significant change of thermal stability indicating that none of the compounds binds *Am*GSTF1.

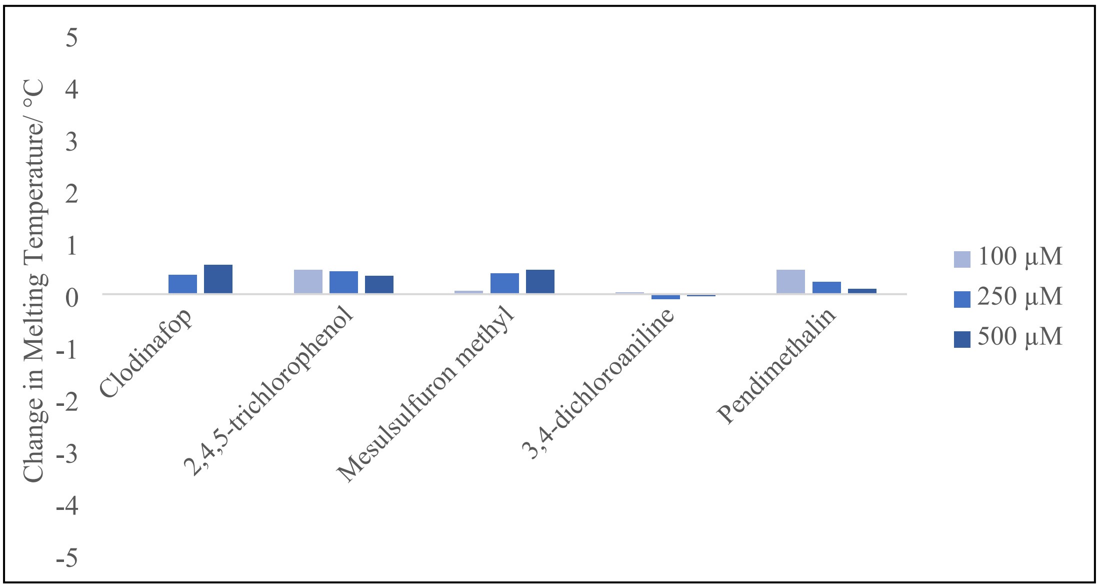

Figure S7: *Am*GSTF1 selectively binds apigenin (flavonoids) from a cocktail of putative inhibitors. **a:** Ligand fishing set of apigenin **1**, purines and related heterocycles previously reported to inhibit glutathionylation of *Am*GSTF1. **b**. Representative HPLC trace from ligand fishing experiment showing selective retention of apigenin **1** by column bound *Am*GSTF1 when compared with NBD-SG.

(a)

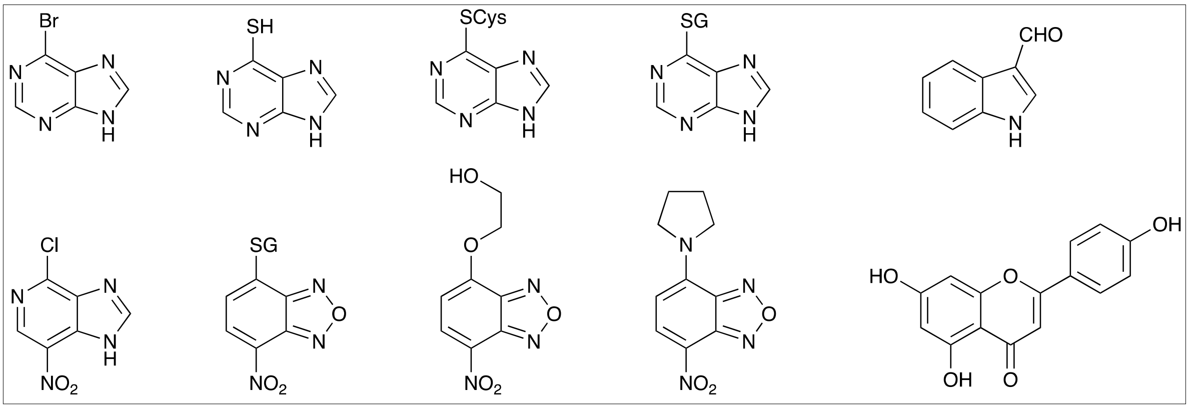

(b)

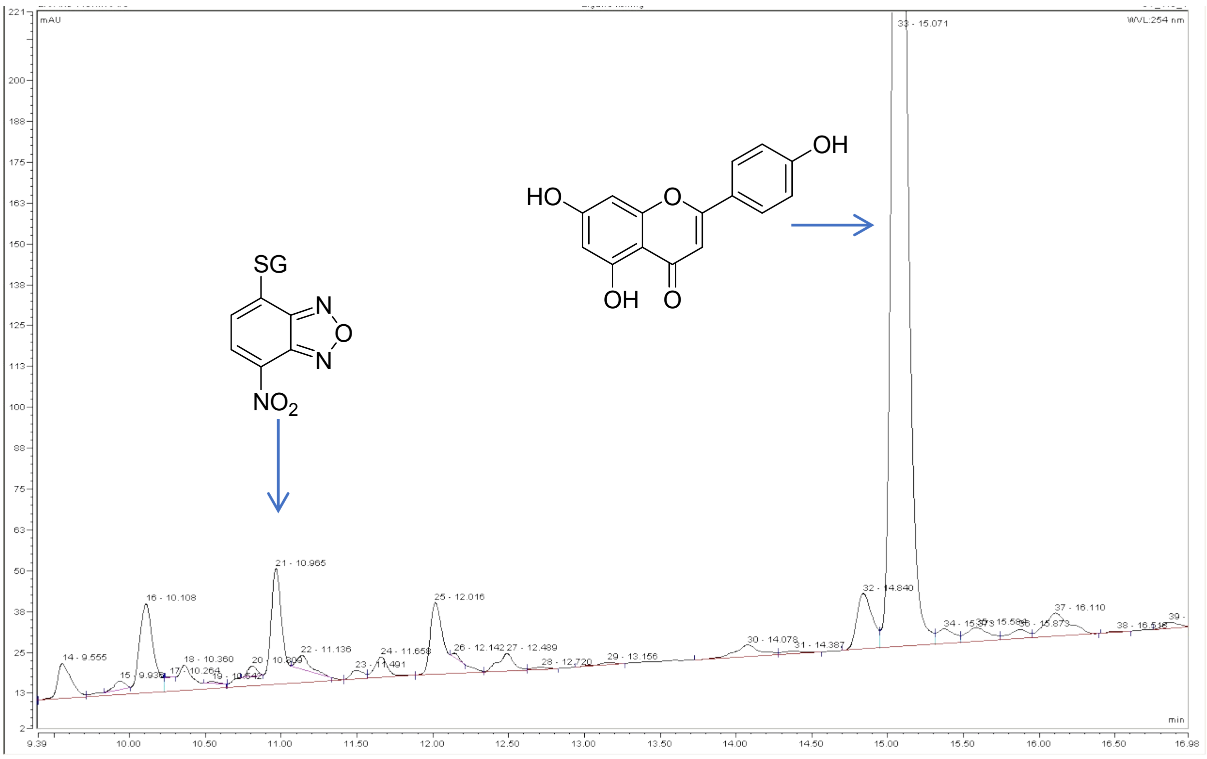

Figure S8: Strep-tagged recombinant protein was immobilized on a streptactin column and exposed to crude extracts from black-grass plants at the tillering stage (BBCH35). After washing, the retained *Am*GSTF1-bound ligands were analysed by UPLC-MS. A) The chromatogram shows that a single major UV-absorbing compound (RT = 2.40, $[M-H]^-$ = 1139.3) was bound by *Am*GSTF1. B) The MS-MS analysis shows that these results were consistent with this compound being a derivative of an arabinosylated C-glucoside of the flavone apigenin ($[M-H]^-$ = 563.). C) In support of this proposal similar MS analysis of MeOH extracts of MHR black grass showed signals and fragmentation pattern expected for apigenin diglycosides ($[M+H]^+$ = 565.2, 415.1, 313.1, 283.1) published previously [18].

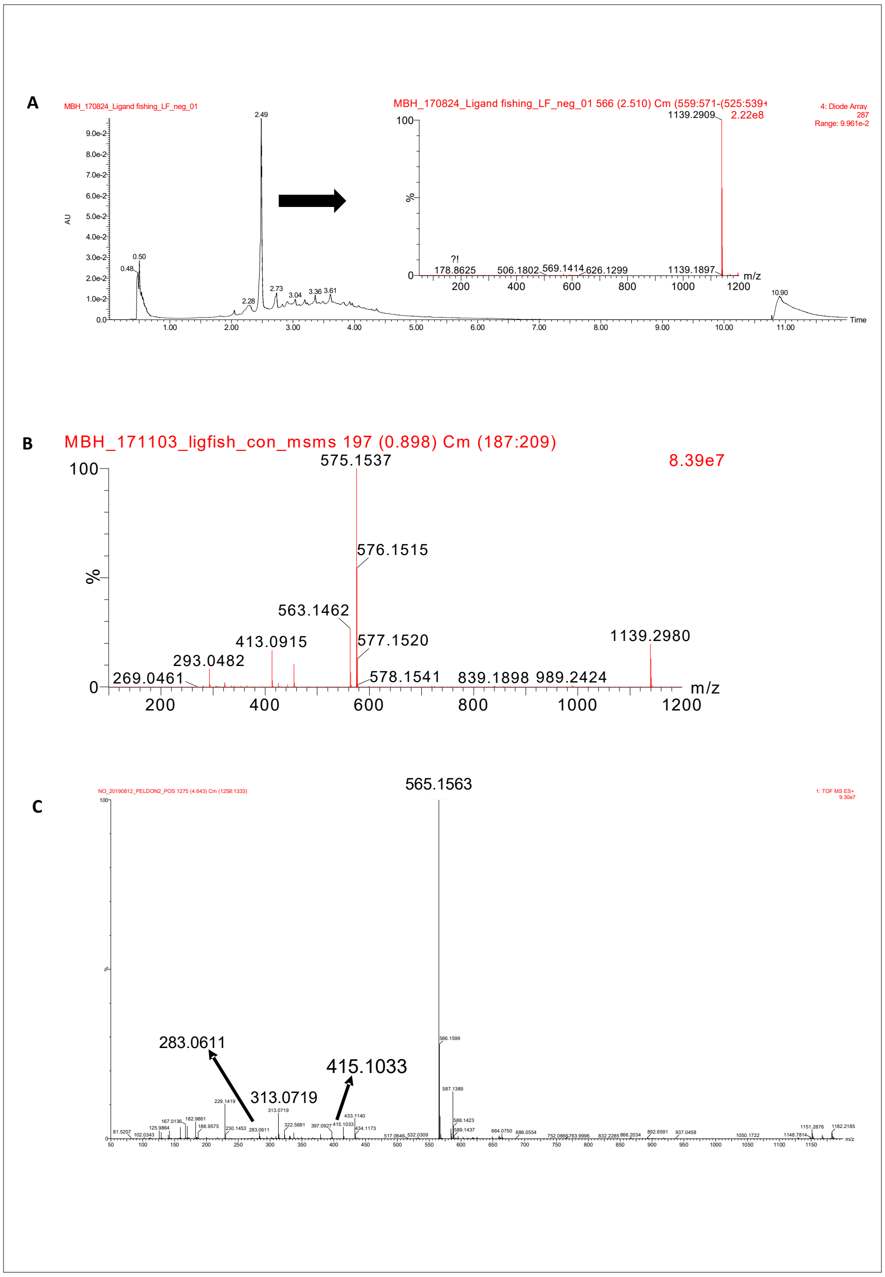

Figure S9: Wheat (top) and black grass (bottom - WT= wild type sensitive, MHR = multiple herbicide resistant) sprayed with apigenin and pinoxaden (PXD): U = untreated, V = vehicle (5% acetone, 0.5% Adigor), apigenin (20 mL, 2 mM, 5% acetone, 0.5% Adigor), PXD = pinoxaden (30 mL, 20 uM, 5% acetone, 0.5% Adigor). Total 50 mL spray/pot.

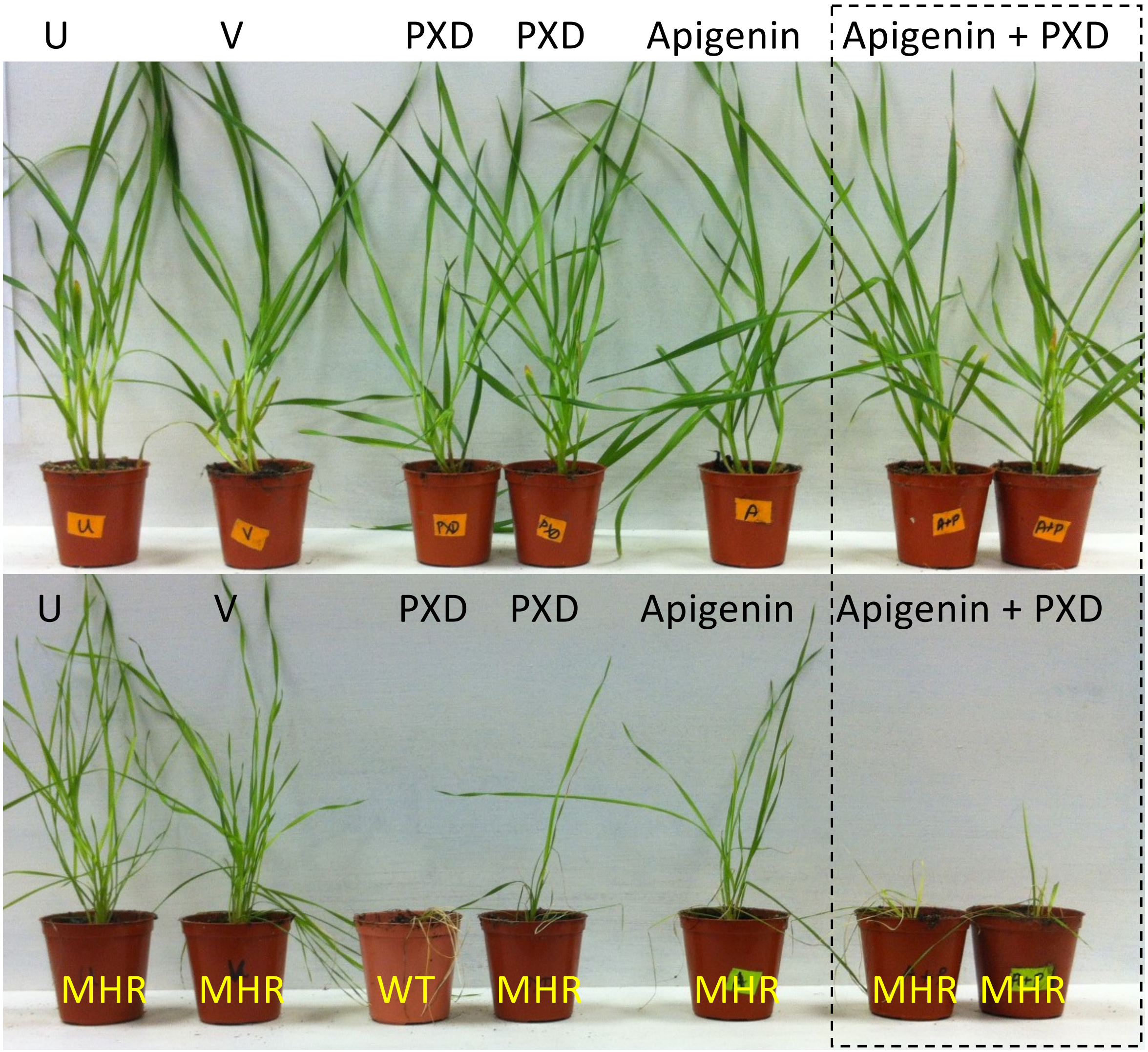

Figure S10: Comparison of inhibition of *Am*GSTF1 (dark blue) compared to *At*GSTF8 (light blue) by synthetic flavonoids with differing alkyl chain length using the CDNB assay.

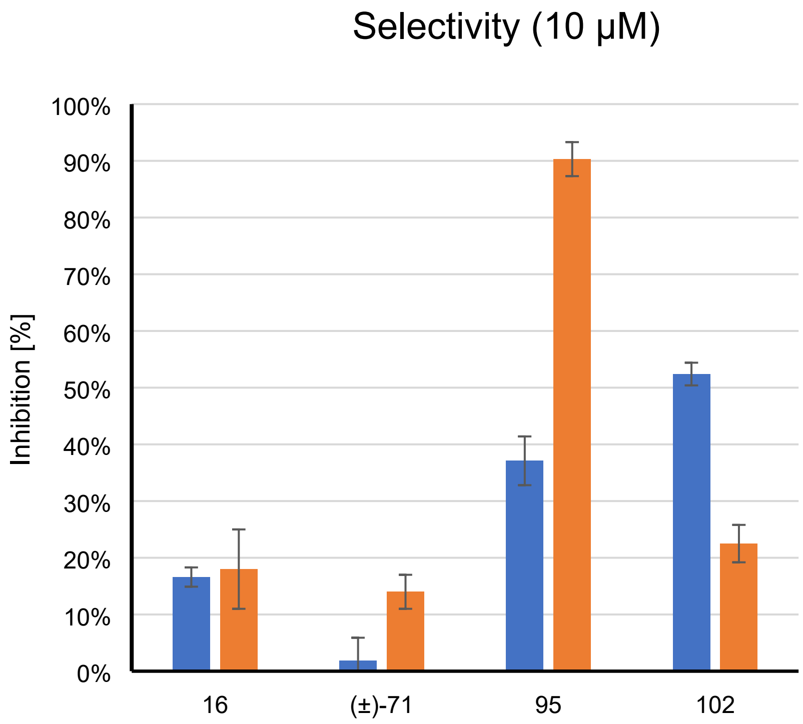

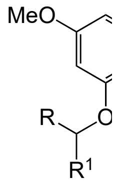

Figure S11: Enzymatic activity of *Am*GSTF1 variants used for crystallisation experiments in the CDNB assay.

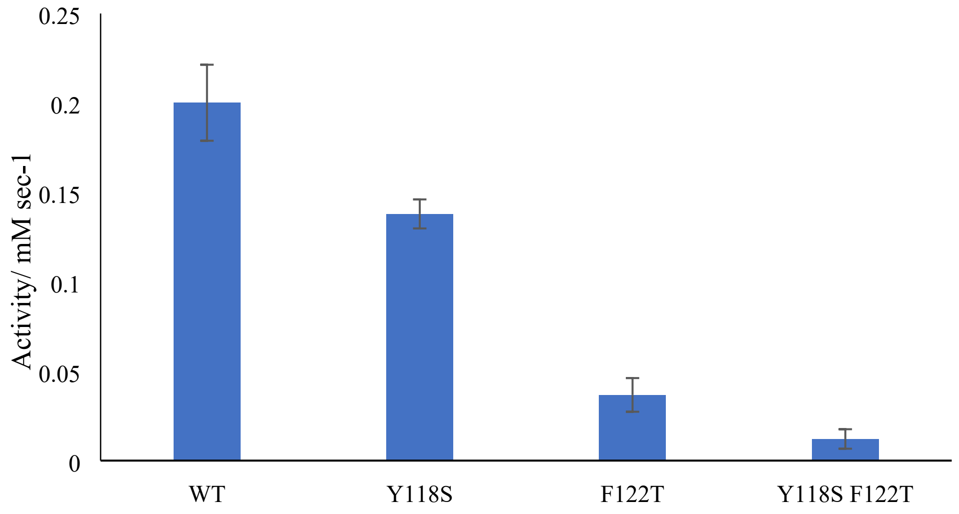

Figure S12: Least squares superposition of the Tyr118Ser (orange), Phe122Thr (cyan) with wild-type *Am*GSTF1 (magenta) with the GSH-NBD ligand shown in a stick representation occupying the G-site.

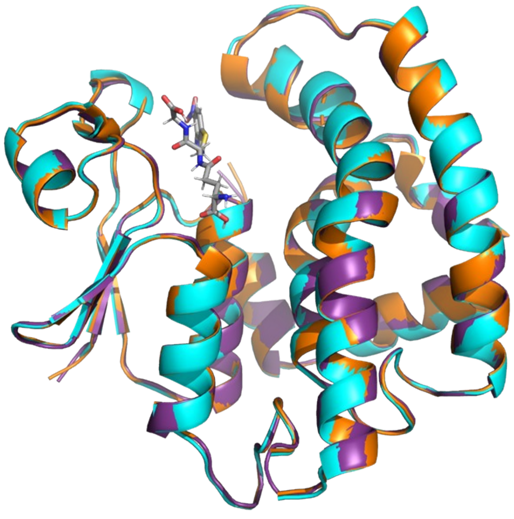

Supplementary Tables

Table S1a: Crystallographic data for the hexagonal and rhombohedral crystal forms of *Am*GSTF1.

	***Am*GSTF1 WT apo**	***Am*GSTF1 WT (rhombohedral)**	***Am*GSTF1 WT covalent CNBF**
Space Group	$P\ 6_3\ 2\ 2$	R32 (H)	$P\ 6_3\ 2\ 2$
Unit cell dimensions [Å], [˚]	a=b=103.69, c=78.77 $\alpha=\beta=90$, $\gamma=120$	a=b=180.78, c=237.89 $\alpha=\beta=90$, $\gamma=120$	a=b=104.08, c=79.84 $\alpha=\beta=90$, $\gamma=120$
Resolution (Å)[a]	51-1.53 (1.56-1.53)	41.9-1.95	59.4 -2.80 (2.90-2.80))
Beamline	DLS i04-1	SLS-PXIII	Bruker Microstar
R_{merge}[a]	0.034 (0.79)	0.046(045)	0.083 (0.411)
rMeas[a]	0.036 (0.887)	n.d.	n.d.
I / *I*[a]	27.3 (2.0)	26.5 (3.7)	43.0 (6.0)
Completeness (%)[a]	100.0 (99.5)	99.1 (99.9)	99.9 (100)
No. reflections (all/free)	38090/1777	101894 (5352)	6674/316
R_{work} / R_{free}	0.19/0.22	0.23/026	0.21/0.28
No. of non H atoms<br>Protein<br>Water<br>Ligand	 1540<br>112<br>-	 8995<br>587<br>57	 1500<br>8<br>-
B-factors<br>Protein<br>Water<br>Ligand	 32.1<br>39.2<br>-	 38.4<br>42.9<br>56.6	 39<br>36<br>-
R.m.s. deviations<br>Bond lengths (Å)<br>Bond angles (°)	 0.019<br>1.94	 0.016<br>1.88	 0.009<br>1.72
PDB code	6TJS	6TNL	6TO3

[a]The values in parentheses correspond to the outermost resolution shell

Table S1b: Crystallographic data for the tetragonal crystal forms of wild-type and variants of *Am*GSTF1

	***Am*GSTF1 F122T**	***Am*GSTF1 Y118S**	***Am*GSTF1 WT GS-NBF**
Space Group	I 4_1 2 2	I 4_1 2 2	I 4_1 2 2
Unit cell dimensions [Å], [˚]	a = b= 112.6 c = 104.3 α=β=γ=90	a = b = 112.6, c = 104.6 α=β=γ=90	A = b = 112.9, c = 101.7 α=β=γ=90
Resolution (Å)[a]	43.6-2.78 (2.93-2.70)	45.30-2.60 (2.72-2.60)	35.70-2.30 (2.38-2.30)
Beamline	DLS I04-1	DLS I04-1	DLS I04-1
R_{merge}[a]	0.040 (0.456)	0.093 (1.02)	0.069 (0.971)
rMeas[a]	0.057 (0.645|)	0.096 (1.06)	0.019 (0.186)
I / sigma(I)[a]	10.7 (1.3)	22.7 (3.7)	26.5 (3.7)
Completeness (%)[a]	99.8 (100)	100 (99.5)	100 (98.6)
No. reflections (all/free)	8717/478	10565/549	14901/715
R_{work} / R_{free}	0.18/0.25	0.19/0.26	0.19/0.24
No. of non H atoms<br>Protein<br>Water<br>Ligand	 1649<br>5<br>29	 1697<br>26<br>20	 1711<br>51<br>39
B-factors<br>Protein<br>Water<br>Ligand	 86<br>76<br>791	 82<br>65<br>69	 73<br>64<br>78
R.m.s. deviations<br>Bond lengths (Å)<br>Bond angles (°)	 0.008<br>1.68	 0.014<br>2.09	 0.14<br>1.97
PDB code	6TK8	7ODM	7OBO

[a]The values in parentheses correspond to the outermost resolution shell

Table S2: The activities of recombinant native and mutant forms of *Am*GSTF1 enzymes toward different class of substrates. The GST activities assessed include the *S*-glutathionylation of acceptors by substitution (1-chloro-2,4-dinitrobenzene, CDNB), by addition reactions (benzylisothiocyanate, BITC), the reduction of organic hydroperoxides (cumene hydroperoxide, CuOOH), ester hydrolysis (*p*-nitrophenyl acetate, NPA) and thiol exchange (hydroxyethyl disufide, HED). An average enzyme activity of each substrate class was reported (mean value ±SE, n = 3).

**Substrate**	**Enzyme and activity (nmol s^{-1} mg^{-1} protein)**		
	***Am*GSTF1** (Native)	***Am*GSTF1** (S12A)	***Am*GSTF1** (C120V)
**CDNB**	25.4±0.8	5.5±0.5	27.8±1.6
**CuOOH**	19.6±1.4	5.1±0.1	24.1±0.8
**HED**	ND	ND	ND
**NPA**	1.1±0.1	0.8±0.0	1.1±0.0
**EA**	16.4±0.8	3.0±0.5	15.7±0.3
**BITC**	34.2±0.7	13.0±0.8	34.8±1.0

Table S3: Inhibition of *Am*GSTF1 and C120V following treatment with the NBD-glutathione conjugate. Enzymes were incubated with 100 µM NBD-Cl, 100 µM NBD-SG or solvent control (DMSO) for 60 min at 20 °C. Following incubation, enzymes were diluted 1:100 (v/v) and assayed for enzyme activity towards 1-chloro-2,4-dinitrobenzene. Mean specific activities are shown ± SD, n = 3. NBD-Cl: 4-chloro-7-nitro-benzoxadiazole. NBD-SG: NBD-glutathione conjugate.

**Enzyme**	**Chemical treatment**	**Mean specific activity (nmol s^{-1} mg^{-1} protein)**	**Inhibition vs. DMSO control (%)**
wildtype *Am*GSTF1	DMSO	19.6 ± 1.2	-
	NBD-Cl	7.8 ± 1.1	60
	NBD-SG	11.2 ± 1.1	43
Cys120Val *Am*GSTF1	DMSO	25.6 ± 1.0	-
	NBD-Cl	21.1 ± 1.1	18
	NBD-SG	22.4 ± 1.6	13

Table S4: Inhibition of *Am*GSTF1 in the CDNB assay. Values are reported as % inhibition of the specific enzyme activity. Each value is the result of three replicate measurements. TSA results are results as difference in melting temperature compared to no ligand.

Entry	Compound		CDNB Assay<br>% Inhibition			Thermal Shift Assay<br>ΔT_m		
			100 µM	10 µM	1 µM	100 µM	10 µM	1 µM
1	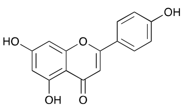	**1**	87	71				
2	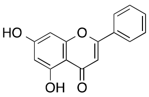	**2**	96	47	-	0.2	0.1	0.1
3	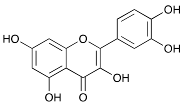	**3**	94	44	-			
4	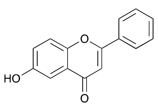	**4**	61	-	-	-0.2	0.0	0.4
5	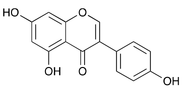	**5**	47					
6	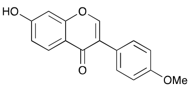	**6**	41					
7	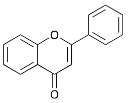	**7**	14	-	-	-0.2	0.0	0.3
8	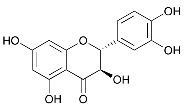	**8**	18%					
9	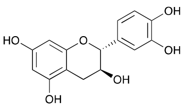	**10**	0%					

10	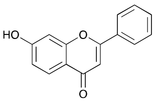	**11**	69	-	-	0.5	0.5	0.2
11	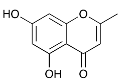	**12**	63	-	-			
12	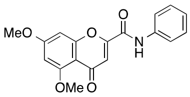	**16a**	49	26	n.d.	0.03	0.03	0.17
13	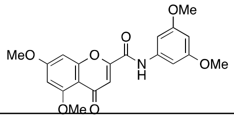	**16b**	56	19	n.d.	0.13	0.50	0.20
14	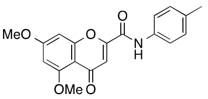	**16c**	64	33	n.d.	0.33	0.10	0.20
15	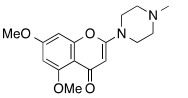	**17a**	16	n.d.	n.d.	0.20	-0.03	0
16	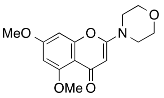	**17b**	37	0	n.d.	0.20	0.23	0.06
17	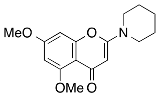	**17c**	67	12	n.d.	0.30	0.13	0.23
18	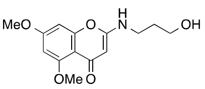	**17d**	12	n.d.	n.d.	0.07	0.17	0.23
19	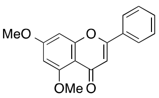	**18a**	70	20	n.d.	0.3	0.33	0.23
20	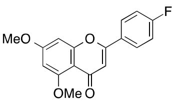	**18b**	78	23	n.d.	0.27	0.33	0.17

21	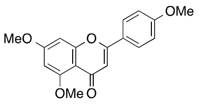	**18c**	100	15	n.d.	0.70	0.17	0.23
22	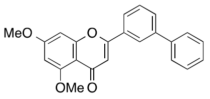	**18d**	87	12	n.d.	-0.1	0.13	0.13
23	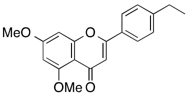	**18e**	80	11	n.d.	0	0.10	30
24	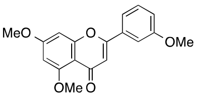	**18f**	79	15	n.d.	-0.17	0.2	0.33
25	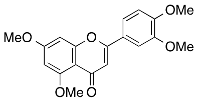	**18g**	100	11	n.d.	0.17	-0.03	0.30
26	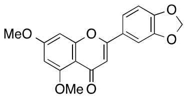	**18h**	33	12	n.d.	0.33	0.17	0.27
27	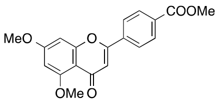	**18i**	58	20	n.d.	0.27	0.30	0.27
28	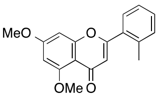	**18j**	36	9	n.d.	-0.03	-0.03	-0.13
29	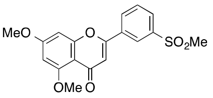	**18k**	60	11	n.d.	0.30	0.10	0.20
30	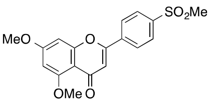	**18l**	35	14	n.d.	0.30	0.20	0.13
31	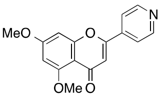	**18m**	50	10	n.d.	0.23	0.27	0.23
32	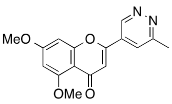	**18n**	64	8	n.d.	-0.33	-0.13	-0.07
33	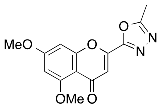	**19**	53	8	n.d.	-0.27	-0.17	-0.10

34	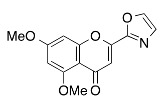	**20**	64	11	n.d.	-0.23	-0.17	-0.10
35	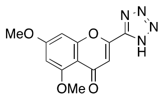	**22**	53	13	n.d.	-0.43	-0.27	-0.23
36	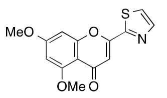	**23**	83	23	n.d.	-0.50	-0.47	-0.40
37	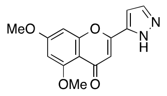	**24**	59	6	n.d.	-0.03	-0.07	-0.30
38	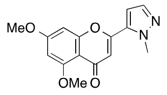	**25**	58	9	n.d.	-0.10	-0.03	0
39	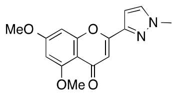	**26**	64	19	n.d.	-0.17	-0.03	0
40	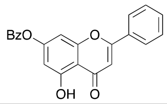	**27**	4	n.d.	n.d.	n.d.	n.d.	n.d.
41	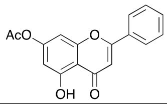	**28**	10	n.d.	n.d.	n.d.	n.d.	n.d.
42	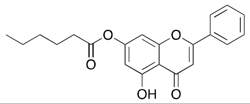	**29**	6	n.d.	n.d.	n.d.	n.d.	n.d.
43	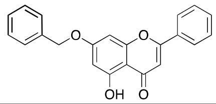	**30**	53	n.d.	n.d.	n.d.	n.d.	n.d.
44	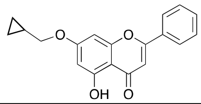	**31**	76	n.d.	n.d.	n.d.	n.d.	n.d.
45	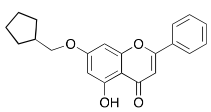	**32**	80	n.d.	n.d.	n.d.	n.d.	n.d.
46	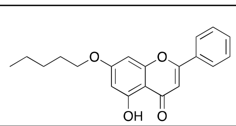	**33**	87	n.d.	n.d.	n.d.	n.d.	n.d.
47	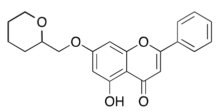	**34**	28	n.d	n.d.	n.d.	n.d.	n.d.

48	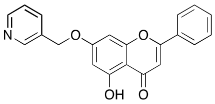	**35**	87	n.d.	n.d.	n.d.	n.d.	n.d.
49	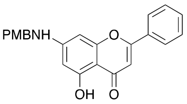	**36**	45	n.d.	n.d.	n.d.	n.d.	n.d.
50	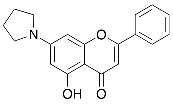	**37**	46	n.d.	n.d.	n.d.	n.d.	n.d.
51	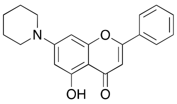	**38**	88	n.d.	n.d.	n.d.	n.d.	n.d.
52	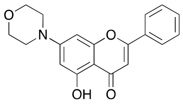	**39**	100	56	n.d.	n.d.	n.d.	n.d.
53	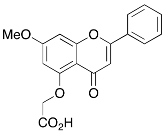	**40**	n.d.	n.d.	23	n.d.	n.d.	n.d.
54	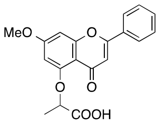	**41**	n.d.	14	n.d.	1.10	0.20	0.20
55	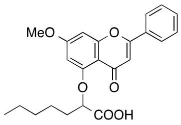	**42**	n.d.	69	4	2.33	0.43	0.17
56	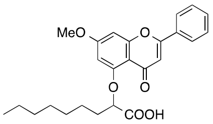	**43**	n.d.	86	40	2.93	0.70	0.27
57	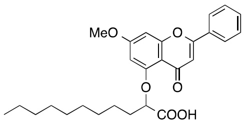	**44**	n.d.	97	58	0.90	0.70	0.40
58	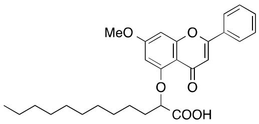	**45**	100	99	71	1.17	0.23	0.30
59	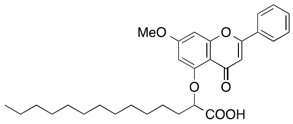	**46**	100	90	75	1.43	0.73	0.20

60	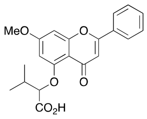	**47**	n.d.	n.d.	7	n.d.	n.d.	n.d.
61	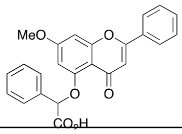	**48**	n.d.	n.d.	16	n.d.	n.d.	n.d.
62	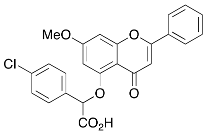	**49**	n.d.	n.d.	27	n.d.	n.d.	n.d.
63	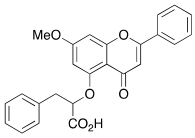	**50**	n.d.	n.d.	8	n.d.	n.d.	n.d.
64	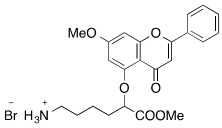	**51**	n.d.	25	n.d.	0.57	0.43	0.20
65	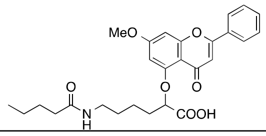	**52**	n.d.	39	6	1.10	0.30	0.13
66	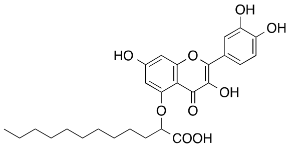	**55**	100	96	70	1.10	2.17	0.20

Chemical Synthesis

General Notes

Chemicals: All chemicals were purchased from commercial suppliers and were used without further purification unless otherwise stated.

Dry Solvents: 1,2-Dichloroethane and methyl-*tert*-butyl-ether (MTBE) were purchased anhydrous from Sigma Aldrich or Acros respectively. All other dry reaction solvents were dried using an Innovative Technology Solvent Purification System and stored under argon.

Column chromatography: Flash column chromatography was performed on a CombiFlash® System from Teledyne Isco equipped with an UV-light detector using prepacked silica RediSep Rf cartridges with the stated solvent gradient. Crude mixtures to be purified were dry loaded onto silica prior to loading on the column.

LC-MS: LC-MS analyses were conducted on a TQD mass spectrometer (Waters Ltd, UK), which was equipped with an Acquity UPLC, using an Acquity UPLC BEH C18 (2.1 mm × 50 mm, 1.7 μm) column, and an electrospray ion source. Absorbance data were acquired from 210 to 400 nm using an Acquity photodiode array detector.

GC-MS: GC-MS analyses were performed on a Shimadzu QP2010-Ultra using electron ionization (EI). The mass spectrometer was equipped with either an Rxi-5Sil MS column (0.15 μm × 10 m × o.15 mm) for non polar compounds or an Rxi-17Sil MS column (0.15 μm × 10 m × o.15 mm) for polar compounds.

ASAP: ASAP measurements were performed on a LCT Premier XE mass spectrometer and ASAP ion source.

HRMS: HRMS measurements were carried out on a QToF Premier mass spectrometer (Waters Ltd, UK) with an electrospray ion source or a LCT Premier XE mass spectrometer with an ASAP ion source.

IR spectroscopy: Infrared (IR) spectra were recorded on a Perkin-Elmer RX I FT-IR spectrometer via use of a Pike ATR accessory in the range of 3500 – 600 cm^{-1}. Assigned peaks are reported in wavenumbers (cm^{-1}).

Melting points: Melting points were measured in open capillary tubes using a Thermo Scientific™ Melting Point Apparatus and are uncorrected.

Microwave: Microwave reactions were performed in septum-containing, crimp capped, sealed vials in a Emrys™ Optimizer microwave unit from Personal Chemistry. The reported times are hold times.

NMR-spectroscopy: NMR-spectra were recorded in $CDCl_3$, d^6-DMSO, CD_3OD or D_2O solutions on a Bruker Advance-400 (^1H, ^{13}C, ^{19}F, DEPT, COSY), Varian Inova-600 (^1H, ^{13}C, ^{19}F, HSQC, HMBC, COSY, NOESY) or Varian VNMRS-700 (^1H, ^{13}C, ^{19}F, HSQC, HMBC, COSY, NOESY) spectrometer, using the solvent peak ($CDCl_3$: δ = 7.26 ppm (^1H), δ = 77.16 ppm (^{13}C), d^6-DMSO: δ = 2.50 ppm (^1H), δ = 39.52 ppm

(^{13}C) CD_3OD: δ = 3.31 ppm (^1H), δ = 49.00 ppm (^{13}C), D_2O: δ = 4.79 ppm (^1H)) as reference. For some spectra tetramethylsilane (TMS) was used as an internal standard. ^{13}C spectra were run in proton-decoupled mode. Chemical shift values (δ) are reported in parts per million (ppm) and coupling constants (J) are given in Hertz (Hz). The multiplicity is indicated by singlet (s), doublet (d), triplet (t), quartet (q), multiplet (m), broad (br) or a combination thereof. Assignment of spectra was carried out using 2D HSQC, HMBC, COSY and NOESY techniques.

TLC: TLC analyses were performed on pre-coated aluminium-backed plates (Silica gel 60 F254, Merck). Signals were visualized with UV-light (254 nm and 365 nm) or by staining with potassium permanganate in water where necessary.

5,7-Dihydroxy-2-methylchromen-4-one [19] **(12)**

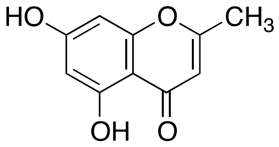

A suspension of 2,4,6-trihydroxyacetophenone (2.0 g, 11.9 mmol) and sodium acetate (0.88 g, 11.9 mmol) in glacial acetic acid (6 mL) was heated at 180 ºC for 40 min by microwave irradiation. The brown gelatinous mixture was added to H_2O (50 mL) and stirred for 30 min affording. The supernatant aqueous phase was decanted and the beige sticky solid was suspended in a solution of 0.1 M K_2CO_3 (15 mL) and subsequently heated at 100 ºC for 2 h using conventional heating. The resulting cooled mixture was acidified by addition of 5 M HCl, affording a beige precipitate, which was isolated by filtration and washing with H_2O (30 mL) followed by Et_2O (30 mL) (700 mg, 31% over two steps). Mp = 278-280 ºC; ^1H NMR (400 MHz, DMSO-d_6) ppm 2.12 - 2.40 (s, 3H, CH_3), 6.13 (m, 2H, ArH), 6.24 - 6.43 (s, 1H, H-3 alkene), 10.78 (s, 1H, OH), 12.80 (br s, 1H, OH); ^{13}C NMR (101 MHz, DMSO-d_6) ppm 19.84, 93.63, 98.67, 103.35, 107.87, 157.71, 161.43, 164.00, 167.56, 181.67. LCMS (ES^+) *m/z* = 193.04 $[M+H]^+$; λ_{max} (MeOH) = 255.8, 296.8. All data in agreement with literature.

5,7-Dimethoxy-4-oxo-4H-chromene-2-carboxylic acid ***13***

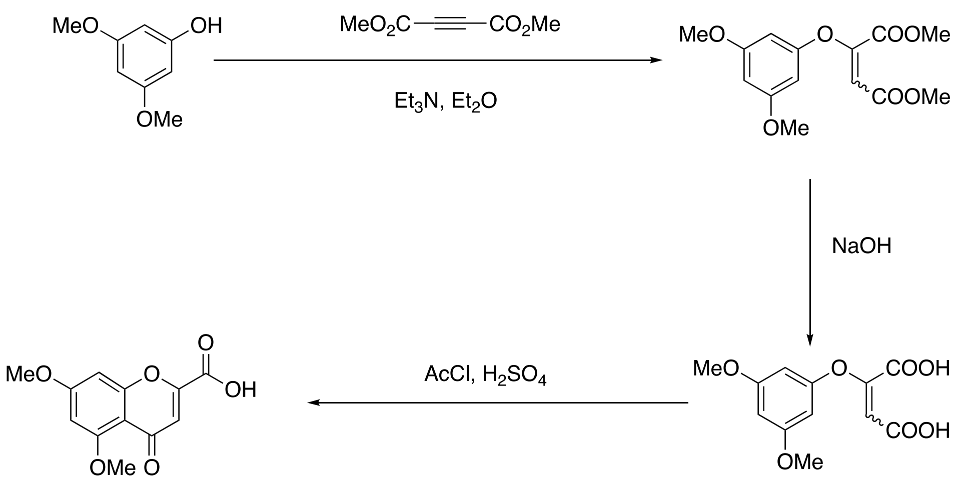

13

3,5-Dimethoxyphenol (920 mg, 6 mmol, 1 eq) was dissolved in dry diethylether (9 ml) under an argon atmosphere. First Et_3N (1.17 ml, 8.4 mmol, 1.4 eq) and then dimethylacetylenedicarboxylate (0.96 ml, 7.8 mmol, 1.3 eq) were added and the solution was stirred at rt for 5.5 h. The reaction mixture was washed with 1 N HCl (3 ml), H_2O (3 ml) and brine (3 ml), dried over Na_2SO_4 and concentrated. Flash column chromatography (40 g silica gel, hexane:EtOAc 9:1 – 4:1) afforded *dimethyl (E,Z)-2-(3′,5′-dimethoxyphenoxy)ethene-1-2-dicarboxylate* (1:0.9 mixture of E:Z isomers, 1.44 g, 81 %) as a colourless solid. M.p.: 56 – 58 °C; δ_H ($CDCl_3$, 400 MHz): 6.57 (s, 1H, 1-H (E)), 6.33 (t, J = 2.2 Hz, 1H, 4′-H (Z)), 6.26 (d, J = 2.2 Hz, 2H, 2′-H, 6′-H (Z)), 6.17 (t, J = 2.2 Hz, 1H, 4′-H (E)), 6.12 (d, J = 2.2 Hz, 2H, 2′-H, 6′-H (E)), 5.24 (s, 1H, 1-H (Z)), 3.92 (s, 3H, COOC**H**$_3$ (Z)), 3.77 – 3.74 (m, 15H, 5 × OC**H**$_3$), 3.71 (s, 3H, COOC**H**$_3$ (E)), 3.67 (s, 3H, COOC**H**$_3$ (Z)); δ_C ($CDCl_3$, 100 MHz): 166.0 (C-2 (E/Z)), 163.9 (C-2 (E/Z)), 163.4 (**C**OOCH$_3$ (Z)), 162.7 (**C**OOCH$_3$ (E)), 161.8 (C-3′, C-5′ (Z)), 161.6 (C-3′, C-5′ (E)), 160.5 (**C**OOCH$_3$ (Z)), 158.3 (C-1′ (E)), 154.6 (C-1′ (Z)), 149.6 (**C**OOCH$_3$ (E)), 115.5 (C-1 (E)), 99.3 (C-1 (Z)), 99.2 (C-2′, C-6′ (Z)), 98.7 (C-4′ (Z)), 95.7(C-4′ (E)), 95.0 (C-2′, C-6′ (E)), 55.7 (2 × O**C**H$_3$ (E/Z)), 55.5 (2 × O**C**H$_3$ (E/Z)), 53.2 (COO**C**H$_3$ (E)), 53.2 (COO**C**H$_3$ (Z)), 52.1 (COO**C**H$_3$ (E)), 51.89(COO**C**H$_3$ (Z)); HRMS (ES^+) found $[M+H]^+$ 297.0995, $C_{14}H_{16}O_7$ requires *M* 297.0974. A 1:0.9 mixture of the E and Z isomer of *dimethyl 2-(3′,5′-dimethoxyphenoxy)ethene-1-2-dicarboxylate* (1.25 g, 4.2 mmol, 1 eq) was heated under reflux with NaOH (680 mg, 16.9 mmol, 4 eq) in H_2O (6.3 ml) for 3 h. The reaction was allowed to cool to rt, washed with Et_2O (3 ml) and acidified to pH 1 with concentrated HCl. Et_2O (7 ml) was added to dissolve the precipitated product and the aqueous layer was extracted with Et_2O (3 × 5 ml). The combined organic layers were dried over Na_2SO_4 and concentrated, which afforded *(E,Z)-2-(3′,5′-dimethoxyphenoxy)ethene-1-2-dicarboxylic acid* (1:0.8 mixture of E:Z isomers, 1.08 g, 95 %) as a yellow solid. M.p.: 147 – 148 °C; δ_H (d^6-DMSO, 400 MHz): 6.51 (s, 1H, 1-H (E)), 6.44 (t, J = 2.2 Hz, 1H, 4′-H (Z)), 6.28 (d, J = 2.2 Hz, 2H, 2′-H, 6′-H (Z)), 6.122 (t, J = 2.2 Hz, 1H, 4′-H (E)), 6.05 (d, J = 2.2 Hz, 2H, 2′-H, 6′-H

(E)), 5.23 (s, 1H, 1-H (Z)), 3.75 (s, 6H, O**C**H_3 (Z)), 3.71 (s, 6H, O**C**H_3 (E)); δ_C (d^6-DMSO, 100 MHz): 165.9 (C-2 (E/Z)), 164.5 (C-2 (E/Z)), 163.3 (**C**OOCH_3 (Z)), 163.0 (**C**OOCH_3 (E)), 161.4 (C-3', C-5' (Z)), 161.1 (C-3', C-5' (E)), 159.0 (**C**OOCH_3 (Z)), 158.2 (C-1' (E)), 155.0 (C-1' (Z)), 148.3 (**C**OOCH_3 (E)), 116.8 (C-1 (E)), 101.9 (C-1 (Z)), 98.4 (C-2', C-6' (Z)), 97.7 (C-4' (Z)), 94.7 (C-4' (E)), 94.5 (C-2', C-6' (E)), 55.7 (2 × O**C**H_3 (Z)), 55.4 (2 × O**C**H_3 (E)),; HRMS (ES^+) found $[M+H]^+$ 269.0691, $C_{12}H_{12}O_7$ requires *M* 269.0661. (*E,Z)-2-(3',5'-dimethoxyphenoxy)ethene-1-2-dicarboxylic acid* (1:0.8 mixture of E:Z isomers, 1.04 g, 3.9 mmol, 1 eq) was dissolved in acetyl chloride (22 ml) and concentrated H_2SO_4 (0.88 ml) slowly added. The reaction mixture was heated to 60 °C and stirred for 10 min. The reaction was allowed to cool to rt and the acetyl chloride removed under reduced pressure. The reaction mixture was cooled with an ice bath and H_2O (XXXpprox.. 65 ml) slowly added. The precipitate was collected by filtration and dried over Na_2SO_4 to afford the title acid **13** (390 mg, 40 %) as a colourless solid. m.p.: 248 - 250 °C; ν_{max} (ATR): 2934 (COO-H), 1748 (C=O), 1640 (C=O), 1594, 1219, 1139, 811, 672 cm^{-1}; δ_H (d^6-DMSO, 400 MHz): 6.72 (d, J = 2.3 Hz, 1H, 8-H), 6.64 (s, 1H, 3-H), 6.54 (d, J = 2.3 Hz, 1H, 6-H), 3.89 (s, 3H, OCH_3), 3.83 (s, 3H, OCH_3); δ_C (d^6-DMSO, 100 MHz): 175.5 (C-4), 164.4 (C-7), 161.5 (COOH), 160.4 (C-5), 159.1 (C-8a), 150.6 (C-2), 115.4 (C-3), 109.1 (C-4a), 96.7 (C-6), 93.4 (C-8), 56.2 (OCH_3), 56.1 (OCH_3); HRMS (ES^+) found $[M+H]^+$ 251.0587, $C_{12}H_{10}O_6$ requires *M* 251.0556.

5,7-Dimethoxy-2-methylthio-4H-chromen-4-one **14**

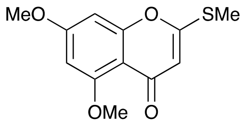

14

n-BuLi (15.1 ml of a 2.5 M solution in hexane, 37.8 mmol, 3.15 eq) was added dropwise over 30 min to a solution of 1,1,1,3,3,3-hexamethyldisilazane (8.25 ml, 39.6 mmol, 3.3 eq) in dry THF (30 ml) under an argon atmosphere at -78 °C. After 10 min the mixture was allowed to warm to 0 °C and stirred for another 10 min. The reaction mixture was recooled to -78°C and a solution of 2-hydroxy-4,6-dimethoxyacetophenone (2.36 g, 12 mmol, 1 eq) in dry THF (9 ml) added dropwise. After stirring for 30 min, CS_2 (1.08 ml, 18 mmol, 1.5 eq) was added in one portion and the reaction mixture allowed to warm to 0°C. After 1 h MeI (1.65 ml, 26.4 mmol, 2.2 eq) was added dropwise over 10 min and stirring continued for another 1 h at rt. 10 N KOH (6 ml) was added and the reaction mixture heated under reflux for 1 h. Then H_2O (20 ml) was added and THF removed under reduced pressure. DCM (20 ml) was added to dissolve the precipitated product and the reaction mixture extracted with DCM (3 × 10 ml). The combined organic layers were dried over Mg_2SO_4 and concentrated. Recrystallization from THF then afforded the title chromenone (2.11 g, 70%) as a pale orange solid.

m.p.: 189 - 191 °C; ν_{max} (ATR): 1621 (C=O), 1585, 1427, 1319, 1304, 1154, 1126, 1106, 1064, 917, 831, 821 cm^{-1}; δ_H (d^6-DMSO, 400 MHz): 6.65 (d, J = 2.3 Hz, 1H, 8-H), 6.48 (d, J = 2.3 Hz, 1H, 6-H), 6.00 (s, 1H, 3-H), 3.85 (s, 3H, OCH_3), 3.80 (s, 3H, OCH_3), 2.53 (s, 3H, SCH_3); δ_C (d^6-DMSO, 100 MHz): 173.1 (C-4), 166.3 (C-2), 163.5 (C-7), 160.3 (C-5), 159.5 (C-8a), 108.1 (C-3), 107.6 (C-4a), 96.3 (C-6), 92.9 (C-8), 56.1 (OCH_3), 56.0 (OCH_3), 13.3 (SCH_3); HRMS (ES$^+$) found $[M+H]^+$ 253.0537, $C_{12}H_{12}O_4S$ requires M 253.0535.

2-Chloro-5,7-dimethoxy-4H-chromen-4-one [20] **15**

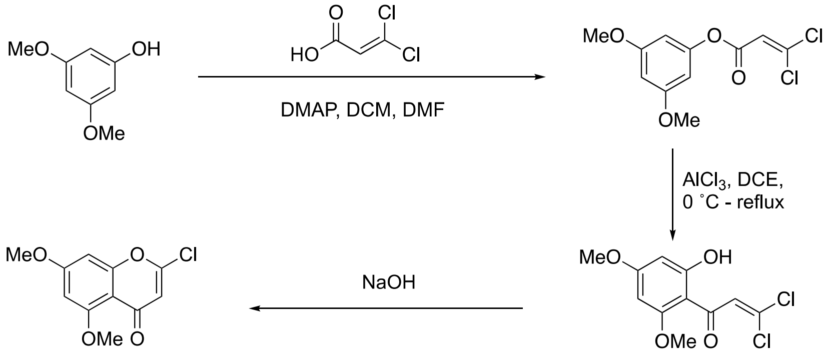

15

A round bottom flask was charged with 3,5-dimethoxyphenol (3.31 g, 21.5 mmol, 1 eq), 3,3-dichloroacrylic acid[21] (3.33 g, 23.6 mmol, 1.1 eq) and 4-(dimethylamino)-pyridine (DMAP) (260 mg, 2.15 mmol, 0.1 eq) and evacuated and flushed with argon three times. DCM (18 ml) and DMF (3.6 ml) were added and the solution was cooled to 0 °C. Diisopropylcarbodiimide (3.33 ml, 21.5 mmol, 1 eq) was added dropwise and after 10 min, the reaction mixture was allowed to warm to rt and stirred for another 2 h. The mixture was filtered through Celite® and washed with 1 M HCl (2 × 10 ml), sat. aqueous $NaHCO_3$ (2 × 10 ml) and with brine (10 ml). The organic layer was dried over Na_2SO_4 and concentrated. The title product was afforded by flash column chromatography (40 g silica gel, hexane:EtOAc 93:7 - 4:1) afforded 3′,5′-*dimethoxyphenyl 3,3-dichloroacrylate*[20] as a colourless solid(5.09 g, 86%). m.p.: 50 - 52 °C; ν_{max} (ATR): 1745 (C=O), 1591, 1456, 1135, 1046, 963, 825 cm^{-1}; δ_H ($CDCl_3$, 400 MHz): 6.58 (s, 1H, 2-H), 6.36 (t, J = 2.2 Hz, 1H, 4′-H), 6.30 (d, J = 2.2 Hz, 2H, 2′-H, 6′-H), 3.77 (s, 6H, 2 × OCH_3); δ_C ($CDCl_3$, 100 MHz): 161.3 (C-3′, C-5′), 160.6 (C-1), 151.7 (C-1′), 140.1 (C-3), 119.3 (C-2), 100.1 (C-2′, C-6′), 98.7 (C-4′), 55.7 (2 × OCH_3); m/z LC-MS (ES$^+$) 155 $[M\text{-}Cl_2CCO+H]^+$, 277 ($[M+H]^+$, Cl^{35}), 279 ($[M+H]^+$, Cl^{35}/Cl^{37}), 281 ($[M+H]^+$, Cl^{37}).

3′,5′-Dimethoxyphenyl 3,3-dichloroacrylate (5.01 g, 18.1 mmol, 1 eq) was dissolved in dry 1,2-dichloroethane (300 ml) and added to a slurry of $AlCl_3$ (3.61, 27.1 mmol, 1.5 eq) in dry 1,2-dichloroethane (100 ml) under an argon atmosphere at 0 °C. Then the reaction mixture was heated under reflux and stirred for 1 h. After cooling down, the mixture was poured over a 1:1 mixture of ice and 1 M HCl (approx. 500 ml) and stirred for 30 min. The organic layer was separated and the aqueous

layer was extracted with DCM (3 × 250 ml). The combined organic layers were washed with H_2O (200 ml) and brine (200 ml), dried over Na_2SO_4 and concentrated. Flash column chromatography (40 g silica gel, hexane:EtOAc 20:1 - 10:1) afforded *3,3-dichloro-1-(2′-hydroxy-4′,6′-dimethoxyphenyl)prop-2-en-1-one*[20] as a yellow solid (2.76 g, 55%). m.p.: 85 - 87 °C; ν_{max} (ATR): 3090 (OH), 3010 (OH), 2951 (OH), 1621 (C=O), 1588, 1557, 1216, 1164, 1110, 948, 820, 736, 753 cm^{-1}; δ_H ($CDCl_3$, 400 MHz): 13.46 (s, 1H, OH), 7.36 (s, 1H, 2-H), 6.08 (d, *J* = 2.3 Hz, 1H, Ar-H), 5.92 (d, *J* = 2.3 Hz, 1H, Ar-H), 3.87 (s, 3H, OCH_3), 3.83 (s, 3H, OCH_3); δ_C ($CDCl_3$, 100 MHz): 189.3 (C-1), 168.3 (C-4′), 167.2 (C-6′), 162.3 (C-2′), 131.5 (C-3), 129.2 (C-2), 106.0 (C-1′), 94.0 (C-5′), 91.4 (C-3′), 56.2 (OCH_3), 55.9 (OCH_3); m/z LC-MS (ES^+) 181 $[M-Cl_2CCH]^+$, 277 ($[M+H]^+$, Cl^{35}), 279 ($[M+H]^+$, Cl^{35}/Cl^{37}), 281 ($[M+H]^+$, Cl^{37}).

3,3-Dichloro-1-(2′-hydroxy-4′,6′-dimethoxyphenyl)prop-2-en-1-one (2.64 g, 9.5 mmol, 1 eq) was dispersed in H_2O (95 ml). The reaction mixture was stirred vigorously for 4 h, adding 1 N NaOH (9.5 ml) over the first hour. 1 N HCl was added to neutralize the reaction mixture and the addition of EtOAc (100 ml) dissolved the precipitated product. The aqueous layer was extracted with EtOAc (3 × 50 ml) and the combined organic layers were washed with H_2O (50 ml) and brine (50 ml), dried over Na_2SO_4 and concentrated. Flash column chromatography (40 g silica gel, DCM:MeOH 97:3) yielded the title chlorochromene **15** (1.88 g, 82%) as a light yellow solid. m.p.: 158 - 160 °C; ⍰$_{max}$ (ATR): 1643 (C=O), 1602, 1319, 1160, 1114, 1088, 914, 847, 828 cm^{-1}; δ_H ($CDCl_3$, 400 MHz): 6.46 (d, *J* = 2.3 Hz, 1H, 8-H), 6.38 (d, *J* = 2.3 Hz, 1H, 6-H), 6.25 (s, 1H, 3-H), 3.93 (s, 3H, OCH_3), 3.88 (s, 3H, OCH_3); ⍰$_C$ ($CDCl_3$, 100 MHz): 176.2 (C-4), 164.4 (C-7), 161.2 (C-5), 160.1 (C-2), 153.5 (C-8a), 112.5 (C-3), 108.4 (C-4a), 97.0 (C-6), 92.9 (C-8), 56.6 (OCH_3), 56.0 (OCH_3); m/z LC-MS (ES^+) 241 ($[M+H]^+$, Cl^{35}), 243 ($[M+H]^+$, Cl^{37}), 263 ($[M+Na]^+$, 265 ($[M+Na]^+$, Cl^{37}).

General Procedure A: Synthesis of 5,7-dimethoxy-4-oxo-4H-chromene-2-carboxamides 16

To a solution of 5,7-dimethoxy-4-oxo-4H-chromene-2-carboxylic acid **13** (62 mg, 0.25 mmol, 1 eq) in dry DMF (0.34 ml) in a microwave vial, $POCl_3$ (38 g, 0.25 mmol, 1 eq) was added. The reaction mixture was stirred at rt for 10 min and then heated to 50 °C and stirred for another 50 min. The amine (0.25 mmol, 1 eq) was added and the mixture heated for 5 min to 160 °C in a microwave. H_2O (4.5 ml) was added, after the reaction was allowed to cool to rt and the precipitated product isolated by filtration. Flash column chromatography (4 g silica gel, hexane:acetone 3:1 - 1:1 (3% Et_3N)) afforded the desired product.

N-Phenyl 5,7-dimethoxy-4-oxo-4H-chromene-2-carboxamide **16a**

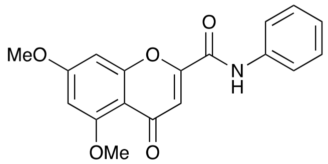

16a

Synthesised from chromene **13** according to General procedure A, using aniline (23 mg). The title product **16a** (39 mg, 48%) was obtained as a colourless solid. m.p.: 203 - 205 °C; ν_{max} (ATR): 1641 (C=O), 1595 (N-C=O), 1418, 1328, 1222, 1161, 1103, 857, 749, 692 cm^{-1}; δ_H (d^6-DMSO, 400 MHz): 10.57 (s, 1H, NH), 7.81 - 7.74 (m, 2H, 2'-H, 6'-H), 7.46 - 7.37 (m, 2H, 3'-H, 5'-H), 7.23 - 7.16 (m, 1H, 4'-H), 6.87 (d, *J* = 2.3 Hz, 1H, 8-H), 6.72 (s, 1H, 3-H), 6.57 (d, *J* = 2.3 Hz, 1H, 6-H), 3.92 (s, 3H, OCH_3), 3.85 (s, 3H, OCH_3); δ_C (d^6-DMSO, 100 MHz): 175.3 (C-4), 164.2 (C-7), 160.4 (C-5), 158.7 (C-8a), 157.7 (CONH), 153.0 (C-2), 137.5 (C-1'), 128.8 (C-3', C-5'), 124.9 (C-4'), 121.1 (C-2', C-6'), 112.8 (C-3), 108.9 (C-4a), 96.7 (C-6), 93.6 (C-8), 56.2 (OCH_3), 56.0 (OCH_3); HRMS (ES$^+$) found $[M+H]^+$ 326.1044, $C_{18}H_{15}NO_5$ requires *M* 326.1028.

N-(3',5'-Dimethoxyhenyl) 5,7-dimethoxy-4-oxo-4H-chromene-2-carboxamide **16b**

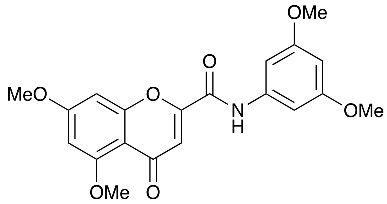

16b

Synthesised from 2-chlorochromene **13** according to General procedure A, using 3,5-dimethoxyaniline (38 mg). The title product **16b** (31 mg, 32%) was obtained as a colourless solid. m.p.: 220 - 222 °C; ν_{max} (ATR): 1651 (C=O), 1601 (N-C=O), 1543, 1455, 1418, 1338, 1262, 1202, 1151, 1054, 822 cm^{-1}; δ_H (d^6-DMSO, 400 MHz): 10.47 (s, 1H, NH), 7.07 (d, J = 2.2 Hz, 2H, 2'-H, 6'-H), 6.87 (d, J = 2.3 Hz, 1H, 8-H), 6.71 (s, 1H, 3-H), 6.57 (d, J = 2.3 Hz, 1H, 6-H), 6.35 (t, J = 2.2 Hz, 1H, 4'-H), 3.92 (s, 3H, OCH_3), 3.85 (s, 3H, OCH_3), 3.76 (s, 6H, 2x OCH_3); δ_C (d^6-DMSO, 100 MHz): 175.3 (C-4), 164.2 (C-7), 160.4 (C-5), 160.4 (C-3'), 158.7 (C-8a), 157.7 (CONH), 152.8 (C-2), 139.2 (C-1'), 112.8 (C-3), 108.9 (C-4a), 99.2 (C-2', C-6'), 96.8 (C-4'), 96.7 (C-6), 93.6 (C-8), 56.2 (OCH_3), 56.1 (OCH_3), 55.2 (OCH_3); HRMS (ES$^+$) found $[M+H]^+$ 386.1249, $C_{20}H_{19}NO_7$ requires *M* 386.1240.

N-(4'-Methylphenyl) 5,7-dimethoxy-4-oxo-4H-chromene-2-carboxamide **16c**

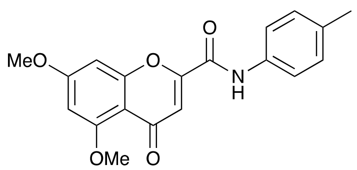

16c

Synthesised from 2-chlorochromene **13** according to General procedure C, using *p*-methylaniline (27 mg). The title product (38 mg, 44%) was obtained as a colourless solid. m.p.: 198 - 200 °C; ν_{max} (ATR): 1645 (C=O), 1597 (N-C=O), 1531, 1316, 1163, 1122, 1104, 1071, 806 cm^{-1}; δ_H (d^6-DMSO, 400 MHz): 10.51 (s, 1H, NH), 7.67 - 7.63 (m, 2H, 2'-H, 6'-H), 7.23 - 7.19 (m, 2H, 3'-H, 5'-H), 6.87 (d, J = 2.3 Hz, 1H, 8-H), 6.70 (s, 1H, 3-H), 6.57 (d, J = 2.3 Hz, 1H, 6-H), 3.92 (s, 3H, OCH_3), 3.85 (s, 3H, OCH_3), 2.30 (s, 3H); δ_C (d^6-DMSO, 100 MHz): 175.3 (C-4), 164.2 (C-7), 160.4 (C-75, 158.7 (C-8a), 157.5 (CONH), 153.1 (C-2), 135.0 (C-1'), 134.0 (C-4'), 129.2 (C-3', C-5'), 121.1 (C-2', C-6'), 112.7 (C-3), 108.9 (C-4a), 96.7 (C-6), 93.6 (C-8), 56.2 (OCH_3), 56.0 (OCH_3), 20.5 (CH_3); HRMS (ES$^+$) found [M+H]$^+$ 340.1202, $C_{19}H_{17}NO_5$ requires *M* 340.1185.

5,7-Dimethoxy-2-(4'-methylpiperazin-1'-yl)-4H-chromen-4-one **17a**

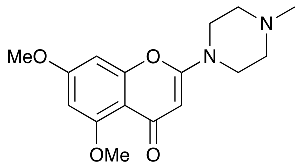

17a

A round bottom flask was charged with 5,7-dimethoxy-2-methylthio-4H-4-chromenone **14** (130 mg, 0.5 mmol, 1 eq), *N*-methyl-piperazine (0.55 ml, 5 mmol, 10 eq) and ethylene glycol (5 ml). The reaction mixture was heated to 160 °C and stirred for 3 h. After cooling to rt, H_2O (5 ml) was added and the reaction mixture extracted with DCM (3 × 2 ml). The combined organic layers were washed with H_2O (1.5 ml) and dried over Na_2SO_4 and concentrated under reduced pressure. Flash column chromatography (12 g silica gel, DCM:MeOH 50:1 - 9:1) yielded the title amine **17a** (39 mg, 25%) as a colourless solid.

m.p.: 71 – 73 °C; ν_{max} (ATR): 1634 (C=O), 1589, 1558, 1412, 1247, 1162, 1110, 1000, 808 cm^{-1}; δ_H ($CDCl_3$, 400 MHz): 6.33 (d, *J* = 2.3 Hz, 1H, 8-H), 6.32 (d, *J* = 2.3 Hz, 1H, 6-H), 5.33 (s, 1H, 3-H), 3.91 (s, 3H, OCH_3), 3.84 (s, 3H, OCH_3), 3.45 (t, *J* = 5.2 Hz, 4H, CH_2), 2.50 (t, *J* = 5.2 Hz, 4H, CH_2), 2.34 (s, 3H, CH_3); δ_C ($CDCl_3$, 100 MHz): 177.7 (C-4), 163.0 (C-2), 161.3 (C-7), 160.7 (C-5), 157.5 (C-8a), 107.6 (C-4a), 95.9 (C-6), 92.5 (C-8), 88.6 (C-3), 56.5 (OCH_3), 55.7 (OCH_3), 54.2 (CH_2), 46.2 (NCH_3), 44.7 (CH_2); HRMS (ES$^+$) found [M+H]$^+$ 305.1516, $C_{16}H_{20}N_2O_4$ requires *M* 305.1501.

General Procedure B: Synthesis of 2-amino- 5,7-Dimethoxy-4H-chromen-4-ones 8.

5,7-Dimethoxy-2-methylthio-4H-chromen-4-one **5** (1.009 g, 4 mmol, 1 eq) was suspended in DCM (28 ml). *Meta*-chloroperoxybenzoic acid (75%, 2.945 g, 12.8 mmol, 3.2 eq) was slowly added at 0 °C and the reaction mixture heated to 35 °C and stirred for 5.5 h. H_2O (16 ml) was added and the reaction mixture extracted with DCM (4 × 15 ml). The combined organic layers were washed consecutively with

saturated (sat.) aqueous $Na_2S_2O_5$ (15 ml), sat. aqueous $NaHCO_3$ (2 × 15 ml) and sat. aqueous NH_4Cl (15 ml), dried over Na_2SO_4 and concentrated. A sample of the crude 5,7-dimethoxy-2-(methylsulfonyl)-4H-chromen-4-one (~ 0.5mmol) in DCM was mixed with amine (296 mg, 3.4 mmol) and stirred for 20 h at rt followed by 26 h heating under reflux. The crude reaction mixture was concentrated and purified by flash column chromatography

5,7-Dimethoxy-2-morpholino-4H-chromen-4-one **17b**

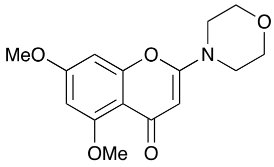

17b

5,7-Dimethoxy-2-morpholino-4H-chromen-4-one **17b** was synthesised according to General procedure B, using morpholine (296 mg, 3.4 mmol) and stirring for 20 h at rt followed by 26 h heating under reflux. The crude reaction mixture was concentrated and purified by flash column chromatography (12 g silica gel, DCM:MeOH 97:3 - 19:1), to afford the title enamide **8b** (47 mg, 32%) as a colourless solid. m.p.: 165 - 168 °C; ν_{max} (ATR): 1634 (C=O), 1593, 1558, 1242, 1242, 1108, 810 cm^{-1}; δ_H ($CDCl_3$, 400 MHz): 6.36 - 6.30 (m, 2H, 6-H, 8-H), 5.33 (s, 1H, 3-H), 3.92 (s, 3H, OCH_3), 3.85 (s, 3H, OCH_3), 3.80 (t, J = 5.0 Hz, 4H, 3'-H_2, 5'-H_2), 3.40 (t, J = 5.0 Hz, 4H, 2'-H_2, 6'-H_2); δ_C ($CDCl_3$, 100 MHz): 177.6 (C-4), 163.1 (C-7), 161.4 (C-2), 160.8 (C-5), 157.5 (C-8a), 107.7 (C-4a), 96.0 (C-6), 92.5 (C-8), 88.8 (C-3), 66.1 (C-3', C-5'), 56.6 (OCH_3), 55.7 (OCH_3), 44.9 (C-2', C-6'); HRMS (ES^+) found $[M+H]^+$ 292.1186, $C_{15}H_{17}NO_5$ requires M 292.1185.

5,7-Dimethoxy-2-(piperidin-1-yl)-4H-chromen-4-one **17c**

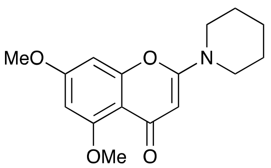

17c

5,7-Dimethoxy-2-(piperidin-1-yl)-4H-chromen-4-one **17c** was synthesised according to General procedure B, using piperidine (290 mg) and stirring for 20 h at rt. The crude reaction mixture was concentrated and EtOAc (20 ml) added. The organic layer was washed with sat. aqueous $NaHCO_3$ (2 × 15 ml) and the reaction mixture was concentrated again. Flash column chromatography (12 g silica gel, DCM:MeOH 97:3 - 19:1) yielded the title amine (72 mg, 50%) as a colourless solid. m.p.: 79 - 81 °C; ν_{max} (ATR): 1627 (C=O), 1595, 1556, 1397, 1245, 1202, 1159, 1140, 1117, 807, 744, 742 cm^{-1}; δ_H ($CDCl_3$, 400 MHz): 6.34 (d, J = 2.3 Hz, 1H, 8-H), 6.32 (d, J = 2.3 Hz, 1H, 6-H), 5.37 (s, 1H, 3-H), 3.91 (s,

3H, OCH_3), 3.85 (s, 3H, OCH_3), 3.45 - 3.38 (m, 4H, 2'-H_2, 6'-H_2), 1.70 - 1.61 (m, 6H, 3'-H_2, 4'-H_2, 5'-H_2); δ_C ($CDCl_3$, 100 MHz): 177.6 (C-4), 162.9 (C-7), 161.2 (C-2), 160.7 (C-5), 157.5 (C-8a), 107.5 (C-4a), 95.8 (C-6), 92.5 (C8), 88.1 (C-3), 56.6 (OCH_3), 55.7 (OCH_3), 46.0 (C-2', C-6'), 25.3 (C-3', C-5'), 24.3 (C-4'); HRMS (ES$^+$) found $[M+H]^+$ 290.1377, $C_{16}H_{19}NO_4$ requires *M* 290.1392.

2-(3'-Hydroxypropylamino)-5,7-dimethoxy-4H-chromen-4-one **17d**

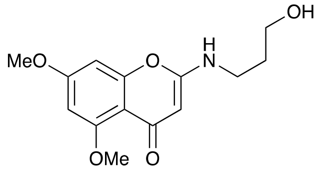

17d

2-(3'-Hydroxypropylamino)-5,7-dimethoxy-4H-chromen-4-one **17d** was synthesised according to General procedure B, using 3-aminopropan-1-ol (290 mg) and stirring for 20 h at rt. The crude reaction mixture was concentrated, H_2O (20 ml) added, and the precipitate collected by filtration. The aqueous layer was extracted with DCM (4 × 15 ml), the combined organic layers were dried over Na_2SO_4 and the extracts were combined with the precipitated product. The reaction mixture was concentrated and purified by flash column chromatography (12 g silica gel, DCM:MeOH 9:1), which yielded the title enamide (47 mg, 33%) as a colourless solid. m.p.: 143 - 146 °C; ν_{max} (ATR): 3380, 3196, 1640 (C=O), 1593, 1549, 1330, 1202, 1077, 804 cm^{-1}; δ_H (CD_3OD, 400 MHz): 6.52 (d, *J* = 2.3 Hz, 1H, 8-H), 6.46 (d, *J* = 2.3 Hz, 1H, 6-H), 5.21 (s, 1H, 3-H), 3.88 (s, 3H, OCH_3), 3.86 (s, 3H, OCH_3), 3.67 (t, *J* = 6.1 Hz, 2H, 3'-H), 3.34 (t, 7.1 Hz, 2H, 1'-H), 1.89 – 1.81 (m, 2H, 2'-H_2); δ_C (CD_3OD, 100 MHz): 179.6 (C-4), 165.0 (C-2), 164.5 (C-7), 161.7 (C-5), 158.6 (C-8a), 107.3 (C-4a), 96.7 (C-6), 93.8 (C-8), 85.62 (C-3), 60.2 (C-3'), 56.4 (OCH_3), 56.3 (OCH_3), 39.5 (C-1'), 32.7 (C-2'); HRMS (ES$^+$) found $[M+H]^+$ 280.1183, $C_{14}H_{17}NO_5$ requires *M* 280.1185.

2-aryl-5,7-dimethoxy-4H-chromen-4-ones 18

General procedure **C**

In a reaction tube of a carousel reactor *2-chloro-5,7-dimethoxy-4H-chromen-4-one* **6** (60 mg, 0.25 mmol, 1 eq), boronic acid (0.50 mmol, 2 eq), $Pd_2(dba)_3$ (6.9 mg, 3 mol%), PCy_3 (4.2 mg, 6 mol%) and Cs_2CO_3 (244 mg, 0.75 mmol, 3 eq) were suspended in dry dioxane (1.25 ml) under a nitrogen atmosphere. The reaction mixture was heated to 100 °C and stirred for 22 h.

5,7-Dimethoxy-2-phenyl-4H-chromen-4-one[20] **18a**

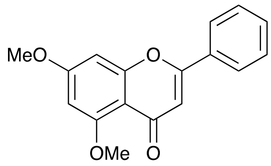

18a

5,7-Dimethoxy-2-phenyl-4H-chromen-4-one **18a** was synthesised according to General procedure C, using phenylboronic acid (61 mg). DCM (2 ml) and H_2O (2 ml) were added to the reaction mixture and the layers separated using a phase separator. The organic layer was concentrated and the residue was diluted with DCM (1 ml). The title product (59 mg, 83%) was obtained as a colourless solid by filtration through silica gel, using DCM (10 ml) to wash and then a 1:1 mixture of DCM and MeOH (10 ml) to elute the product. m.p.: 135 - 138 °C; ν_{max} (ATR): 1644 (C=O), 1606, 1345, 1156, 1116, 820, 764, 694 cm^{-1}; δ_H ($CDCl_3$, 400 MHz): 7.95 - 7.85 (m, 2H, 2'-H, 6'-H), 7.57 - 7.47 (m, 3H, 3'-H, 4'-H, 5'-H), 6.71 (s, 1H, 3-H), 6.60 (d, *J* = 2.3 Hz, 1H, 8-H), 6.41 (d, *J* = 2.3 Hz, 1H, 6-H), 3.99 (s, 3H, OCH_3), 3.94 (s, 3H, OCH_3); δ_C ($CDCl_3$, 100 MHz): 177.6 (C-4), 164.1 (C-7), 160.9 (C-5), 160.7 (C-2), 159.9 (C-8a), 131.6 (C-1'), 131.2 (C-4'), 129.0 (C-3', C-5'), 126.0 (C-2', C-6'), 109.4 (C-4a), 109.1 (C-3), 96.2 (C-6), 92.8 (C-8), 56.5 (OCH_3), 55.8 (OCH_3); m/z LC-MS (ES^+) 283 $[M+H]^+$.

2-(4'-Fluorophenyl)-5,7-dimethoxy-4H-chromen-4-one[22] **18b**

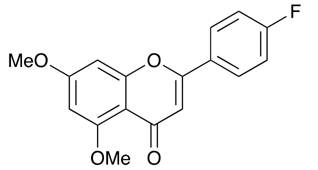

18b

Prepared according to General procedure C, using 4-fluorophenylboronic acid (70 mg). DCM (2 ml) and H_2O (2 ml) were added to the reaction mixture and the layers separated using a phase separator. The organic layer was concentrated and the residue was diluted in DCM (1 ml). The mixture was filtrated through silica gel, using DCM (10 ml) followed by 1:1 DCM:MeOH (10 ml) to elute the product. Flash column chromatography (4 g silica gel, DCM:MeOH 1:0 - 24:1) yielded the title product (38 mg, 50%) as a colourless solid. m.p.: 235 - 237 °C (lit.[22] m.p. 236.5 – 238.5 °C); ν_{max} (ATR): 1650 (C=O), 1602, 1343, 1216, 1198, 1163, 1117, 1100, 832, 812 cm^{-1}; δ_H ($CDCl_3$, 400 MHz): 7.91 - 7.85 (m, 2H, 2'-H, 6'-H), 7.24 - 7.15 (m, 2H, 3'-H, 5'-H), 6.63 (s, 1H, 3-H), 6.57 (d, J = 2.3 Hz, 1H, 8-H), 6.39 (d, J = 2.3 Hz, 1H, 6-H), 3.97 (s, 3H, OCH_3), 3.93 (s, 3H, OCH_3); δ_C ($CDCl_3$, 100 MHz): 177.4 (C-4), 164.5 (d, $^1J_{CF}$ = 252.5 Hz, C-4'), 164.1 (C-7), 160.9 (C-5), 159.8 (C-2), 159.7 (C-8a), 128.1 (d, $^3J_{CF}$ = 8.7 Hz, C-2', C-6'), 127.8 (d, $^4J_{CF}$ = 3.3 Hz, C-1'), 116.2 (d, $^2J_{CF}$ = 22.0 Hz, C-3', C-5'), 109.2 (C-4a), 108.9 (d, J = 1.0 Hz, C-3), 96.2 (C-6), 92.8 (C-8), 56.5 (OCH_3), 55.8 (OCH_3); δ_F ($CDCl_3$, 400 MHz): 107.94; m/z LC-MS (ES^+) 301 $[M+H]^+$.

5,7-Dimethoxy-2-(4’-methoxyphenyl)-4H-chromen-4-one[20] **18c**

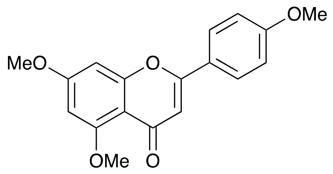

18c

Synthesised according to General procedure B, using 4-methoxyphenylboronic acid (76 mg). DCM (2 ml) and H_2O (2 ml) were added to the reaction mixture and the layers separated using a phase separator. The organic layer was concentrated and the residue was diluted in DCM (1 ml). The mixture was filtrated through silica gel, using DCM (10 ml) to wash and then a 1:1 mixture of DCM and MeOH (10 ml) to elute the product. Flash column chromatography (4 g silica gel, DCM:MeOH 1:0 - 24:1) afforded the title product (72 mg, 92%) as a colourless solid. m.p.: 155 - 157 °C; ν_{max} (ATR): 1640 (C=O), 1602, 1594, 1347, 1254, 1214, 1194, 1162, 1120, 1100, 1030, 830 cm^{-1}; δ_H ($CDCl_3$, 400 MHz): 7.85 - 7.79 (m, 2H, 3’-H, 5’-H), 7.03 - 6.97 (m, 2H, 2’-H, 6’-H), 6.59 (s, 1H, 3-H), 6.56 (d, *J* = 2.3 Hz, 1H, 8-H), 6.37 (d, *J* = 2.3 Hz, 1H, 6-H), 3.96 (s, 3H, OCH_3), 3.92 (s, 3H, OCH_3), 3.89 (s, 3H, OCH_3); δ_C ($CDCl_3$, 100 MHz): 177.6 (C-4), 163.9 (C-7), 162.0 (C-4’), 160.9 (C-5), 160.6 (C-2), 159.8 (C-8a), 127.6 (C-3’,C-5’), 123.8 (C-1’), 114.3 (C-2’, C-6’), 109.2 (C-4a), 107.7 (C-3), 96.1 (C-6), 92.8 (C-8), 56.4 (OCH_3), 55.7 (OCH_3), 55.5 (OCH_3); m/z LC-MS (ES^+) 313 $[M+H]^+$.

2-(3’-Biphenyl)-5,7-dimethoxy-4H-chromen-4-one **18d**

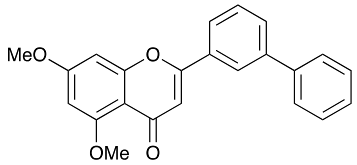

18d

Synthesised according to General procedure B, using 3-biphenylboronic acid (99 mg). DCM (2 ml) and H_2O (2 ml) were added to the reaction mixture and the layers separated using a phase separator. The organic layer was concentrated and the residue was diluted in DCM (1 ml). The mixture was filtrated through silica gel, using DCM (10 ml) to wash and then a 1:1 mixture of DCM and MeOH (10 ml) to elute the product. Flash column chromatography (4 g silica gel, DCM:MeOH 1:0 - 24:1) yielded the title product (57 mg, 50%) as a colourless solid. m.p.: 190 - 193 °C; ν_{max} (ATR): 1649 (C=O), 1603, 1336, 1213, 1154, 1119, 1103, 818, 762, 700 cm^{-1}; δ_H ($CDCl_3$, 400 MHz): 8.05 (td, *J* = 1.9, 0.5 Hz, 1H, 2’-H), 7.82 (ddd, *J* = 7.8, 1.9, 1.1 Hz, 1H, 6’-H), 7.70 (ddd, *J* = 7.8, 1.9, 1.1 Hz, 1H, 4’-H), 7.67 - 7.59 (m, 2H, 2’’-H, 6’’-H), 7.57 - 7.37 (m, 4H, 5’-H, 3’’-H, 4’’-H, 5’’-H), 6.73 (s, 1H, 3-H), 6.57 (d, *J* = 2.3 Hz, 1H, 8-H), 6.36 (d, *J* = 2.3 Hz, 1H, 6-H), 3.94 (s, 3H, OCH_3), 3.90 (s, 3H, OCH_3); δ_C ($CDCl_3$, 100 MHz): 177.6 (C-4),

164.1 (C-7), 160.9 (C-5), 160.5 (C-2), 159.9 (C-8a), 142.1 (C-3'), 140.3 (C-1''), 132.1 (C-1'), 129.9 (C-Ar), 129.4 (C-Ar), 129.0 (C-Ar), 127.9 (C-Ar), 127.2 (C-Ar), 124.8 (C-Ar), 124.7 (C-Ar), 109.4 (C-4a), 109.3 (C-3), 96.3 (C-6), 92.9 (C-8), 56.5 (OCH_3), 55.8 (OCH_3); HRMS (ES^+) found $[M+H]^+$ 359.1295, $C_{23}H_{18}O_4$ requires *M* 359.1283.

2-(4'-Ethylphenyl)-5,7-dimethoxy-4H-chromen-4-one **18e**

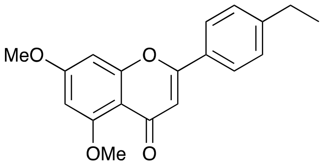

18e

Synthesised according to General procedure B, using 4-ethylphenylboronic acid (0.0750 g). DCM (2 ml) and H_2O (2 ml) were added to the reaction mixture and the layers separated using a phase separator. The organic layer was concentrated and the residue was diluted in DCM (1 ml). The mixture was filtrated through silica gel, using DCM (10 ml) to wash and then a 1:1 mixture of DCM and MeOH (10 ml) to elute the product. Flash column chromatography (4 g silica gel, DCM:MeOH 1:0 - 24:1) afforded the title product (57 mg, 74%) as a colourless solid. m.p.: 126 - 129 °C; ν_{max} (ATR): 1644 (C=O), 1606, 1343, 1214, 1158, 1114, 838, 820 cm^{-1}; δ_H ($CDCl_3$, 400 MHz): 7.82 - 7.77 (m, 2H, 2'-H, 6'-H), 7.36 - 7.31 (m, 2H, 3'-H, 5'-H), 6.66 (s, 1H, 3-H), 6.58 (d, *J* = 2.3 Hz, 1H, 8-H), 6.38 (d, *J* = 2.3 Hz, 1H, 6-H), 3.97 (s, 3H, OCH_3), 3.92 (s, 3H, OCH_3), 2.73 (q, *J* = 7.6 Hz, 2H, CH_2), 1.29 (t, *J* = 7.6 Hz, 3H, CH_3); δ_C ($CDCl_3$, 100 MHz): 177.7 (C-4), 164.0 (C-7), 160.9 (C-5), 160.8 (C-2), 159.9 (C-8a), 148.0 (C-4'), 128.9 (C-1'), 128.5 (C-3', C5'), 126.0 (C-2', C-6'), 109.3 (C-4a), 108.5 (C-3), 96.1 (C-6), 92.8 (C-8), 56.4 (OCH_3), 55.8 (OCH_3), 28.8 (CH_2), 15.3 (CH_3); HRMS (ES^+) found $[M+H]^+$ 311.1280, $C_{19}H_{18}O_4$ requires *M* 311.1283.

5,7-Dimethoxy-2-(3'-methoxyphenyl)-4H-chromen-4-one[23] **18f**

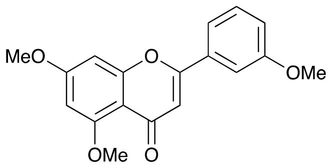

18f

Synthesised according to General procedure B, using 3-methoxyphenylboronic acid (76 mg). The reaction mixture was filtered through Celite® and concentrated. DCM (2 ml) and H_2O (2 ml) were added and the layers separated using a phase separator. The organic layer was concentrated and the title product (58 mg, 75%) was afforded by flash column chromatography (4 g silica gel, DCM:MeOH 1:0 - 19:1) as a light yellow solid. m.p.: 143 - 146 °C (lit.[23] m.p. 147 – 148 °C); ν_{max} (ATR): 1634 (C=O), 1592, 1456, 1350, 1279, 1120, 1107, 1058, 818 cm^{-1}; δ_H ($CDCl_3$, 400 MHz): 7.45 (dt, J = 8.0, 1.2 Hz, 1H, 6'-H),

7.43 - 7.35 (m, 2H, 2'-H, 5'-H), 7.03 (ddd, J = 7.8, 2.5, 1.2 Hz, 1H, 4'-H), 6.66 (s, 1H, 3-H), 6.56 (d, J = 2.3 Hz, 1H, 8-H), 6.37 (d, J = 2.3 Hz, 1H, 6-H), 3.95 (s, 3H, OCH_3), 3.91 (s, 3H, OCH_3), 3.88 (s, 3H, OCH_3); δ_C ($CDCl_3$, 100 MHz): 177.7 (C-4), 164.2 (C-7), 161.0 (C-5), 160.5 (C-2), 160.1 (C-8a), 160.0 (C-3'), 133.0 (C-1'), 130.1 (C-5'), 118.5 (C-6'), 117.0 (C-4'), 111.4 (C-2'), 109.5 (C-4a), 109.4 (C-3), 96.3 (C-6), 93.0 (C-8), 56.6 (OCH_3), 55.9 (OCH_3), 55.6 (OCH_3); m/z LC-MS (ES^+) 313 $[M+H]^+$.

5,7-Dimethoxy-2-(3',4'-dimethoxyphenyl)-4H-chromen-4-one[20] **18g**

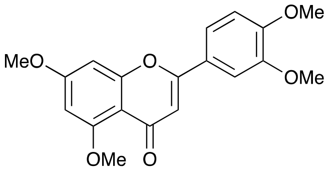

18g

Synthesised according to General procedure B, using 3,4-dimethoxyphenylboronic acid (91 mg). The reaction mixture was filtered through Celite® and concentrated. DCM (2 ml) and H_2O (2 ml) were added and the layers separated using a phase separator. The organic layer was concentrated and the title product (61 mg, 71%) was obtained by flash column chromatography (4 g silica gel, DCM:MeOH 1:0 - 19:1) as a colourless solid. m.p.: 191 - 192 °C; ν_{max} (ATR): 1644 (C=O), 1600, 1355, 1254, 1220, 1138, 1118, 1018, 831, 805 cm^{-1}; δ_H ($CDCl_3$, 400 MHz): 7.51 (dd, J = 8.5, 2.1 Hz, 1H, 6'-H), 7.32 (d, J = 2.1 Hz, 1H, 2'-H), 6.96 (d, J = 8.5 Hz, 1H, 5'-H), 6.61 (s, 1H, 3-H), 6.56 (d, J = 2.3 Hz, 1H, 8-H), 6.38 (d, J = 2.3 Hz, 1H, 6-H), 3.99 - 3.94 (m, 9H, 3 × OCH_3), 3.92 (s, 3H, OCH_3); δ_C ($CDCl_3$, 100 MHz): 177.8 (C-4), 164.1 (C-7), 161.0 (C-5), 160.8 (C-2), 160.0 (C-8a), 151.8 (C-4'), 149.4 (C-3'), 124.2 (C-1'), 119.6 (C-6'), 111.2 (C-5'), 109.4 (C-4a), 108.7 (C-2'), 108.1 (C-3), 96.2 (C-6), 93.0 (C-8), 56.6 (OCH_3), 56.2 (OCH_3), 56.2 (OCH_3), 55.9 (OCH_3); m/z LC-MS (ES^+) 343 $[M+H]^+$.

5,7-Dimethoxy-2-(3',4'-methylenedioxyphenyl)-4H-chromen-4-one **18h**

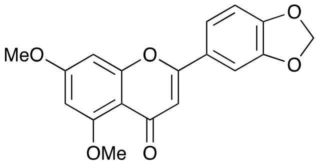

18h

Synthesised according to General procedure B, using 3,4-methylenedioxyphenylboronic acid (83 mg). The reaction mixture was filtered through Celite® and concentrated. DCM (2 ml) and H_2O (2 ml) were added and the layers separated using a phase separator. The organic layer was concentrated and the pure title product (25 mg, 30%) was afforded by flash column chromatography (4 g silica gel, DCM:MeOH 1:0 - 19:1) as a colourless solid. m.p.: 233 - 236 °C; ν_{max} (ATR): 1652 (C=O), 1612, 1451, 1328, 1105, 921, 807 cm^{-1}; δ_H ($CDCl_3$, 400 MHz): 7.43 (dd, *J* = 8.2, 1.8 Hz, 1H, 6'-H), 7.31 (d, *J* = 1.8 Hz,

1H, 2'-H), 6.91 (d, *J* = 8.2 Hz, 1H, 5'-H), 6.58 - 6.53 (m, 2H, 3-H, 8-H), 6.37 (d, J = 2.3 Hz, 1H, 6-H), 6.06 (s, 2H, OCH_2O), 3.95 (s, 3H, OCH_3), 3.91 (s, 3H, OCH_3); δ_C ($CDCl_3$, 100 MHz): 177.7 (C-4), 164.1 (C-7), 161.1 (C-5), 160.5 (C-2), 159.9 (C-8a), 150.4 (C-4'), 148.5 (C-3'), 125.7 (C-1'), 121.1 (C-6'), 109.4 (C-4a), 108.8 (C-5'), 108.3 (C-3), 106.2 (C-2'), 102.0 (CH_2), 96.3 (C-6), 92.9 (C-8), 56.6 (OCH_3), 55.9 (OCH_3); m/z LC-MS (ES^+) 327 $[M+H]^+$.

5,7-Dimethoxy-2-(4'-methoxycarbonylphenyl)-4H-chromen-4-one **18i**

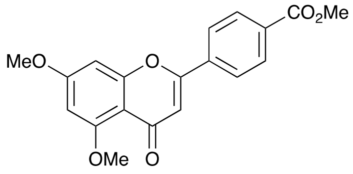

18i

Synthesised according to General procedure B, using 4-methoxycarbonylphenylboronic acid (90 mg). The reaction mixture was filtered through Celite® and concentrated. DCM (2 ml) and H_2O (2 ml) were added and the layers separated using a phase separator. The organic layer was concentrated and flash column chromatography (4 g silica gel, DCM:MeOH 1:0 - 19:1) yielded the title product (58 mg, 68%) as a colourless solid. m.p.: 202 - 204 °C; ν_{max} (ATR): 1715 (C=O), 1640 (C=O), 1594, 1567, 1277, 1218, 1109, 1058, 826, 772, 699 cm^{-1}; δ_H ($CDCl_3$, 400 MHz): 8.18 - 8.13 (m, 2H, 4'-H, 5'-H), 7.97 - 7.92 (m, 2H, 2'-H, 6'-H), 6.74 (s, 1H, 3-H), 6.59 (d, J = 2.3 Hz, 1H, 8-H), 6.40 (d, J = 2.3 Hz, 1H, 6-H), 3.99 - 3.95 (m, 6H, COOC**H**$_3$, OCH_3), 3.93 (s, 3H, OCH_3); δ_C ($CDCl_3$, 100 MHz): 177.5 (C-4), 166.4 (**C**OOCH$_3$), 164.4 (C-7), 161.1 (C-5), 160.0 (C-2), 159.5 (C-8a), 135.8 (C-1'), 132.4 (C-4'), 130.2 (C-3', C-5'), 126.0 (C-2', C-6'), 110.5 (C-3), 109.5 (C-4a), 96.5 (C-6), 93.0 (C-8), 56.6 (OCH_3), 56.0 (OCH_3), 52.6 (COO**C**H$_3$); HRMS (ES^+) found $[M+H]^+$ 341.1035, $C_{19}H_{16}O_6$ requires *M* 341.1025.

5,7-Dimethoxy-2-(2'-methylphenyl)-4H-chromen-4-one **18j**

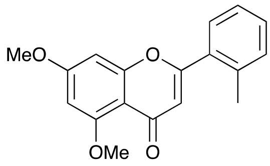

18j

In a microwave vial, 2-chloro-5,7-dimethoxy-4H-chromen-4-one **15** (120 mg, 0.5 mmol, 1 eq) *o*-tolylboronic acid (136 mg, 1 mmol, 2 eq), $Pd_2(dba)_3$ (14 mg, 3 mol%), CyJohnPhos (11 mg, 6 mol%) and Cs_2CO_3 (489 mg, 1.5 mmol, 3 eq) were suspended in dioxane (2.5 ml) under a nitrogen atmosphere. The reaction mixture was heated in the microwave to 150 °C for 30 min. After allowing to cool to rt, the crude mixture was filtered through Celite® and concentrated The residue was dissolved in H_2O (15 ml) and EtOAc (15 ml). The layers were separated and the aqueous layer was extracted two more

times with EtOAc (2 × 25 ml). The combined organic layers were washed with H_2O (25 ml), dried over Mg_2SO_4 and concentrated. The title product (112 mg, 76%) was afforded by reversed phase column chromatography (H_2O:MeCN 0:1 - 1:0, 0.1% TFA) m.p.: 107 – 109 °C; ν_{max} (ATR): 1642 (C=O), 1605, 1333, 1158, 1102, 723 cm^{-1}; δ_H ($CDCl_3$, 400 MHz): 7.55 - 7.46 (m, 1H, 6'-H), 7.44 - 7.35 (m, 1H, 4'-H), 7.35 - 7.27 (m, 2H, 3'-H, 5'-H), 6.48 (d, *J* = 2.3 Hz, 1H, 8-H), 6.39 (d, *J* = 2.3 Hz, 1H, 6-H), 6.36 (s, 1H, 3-H), 3.96 (s, 3H, OCH_3), 3.88 (s, 3H, OCH_3), 2.47 (s, 3H, CH_3); δ_C ($CDCl_3$, 101 MHz): 177.7 (C-4), 164.3 (C-7), 163.4 (C-2), 161.1 (C-5), 160.3 (C-8a), 136.8 (C-2'), 132.4 (C-1'), 131.3 (ArC), 130.6 (C-4'), 129.2 (C-6'), 126.3 (ArC), 113.5 (C-3), 109.2 (C-4a), 96.4 (C-6), 92.8 (C-8), 56.6 (OCH_3), 55.9 (OCH_3), 20.7 (CH_3); HRMS (ES^+) found $[M+H]^+$ 297.1132, $C_{18}H_{17}O_4$ requires *M* 297.1127.

2-(3'-Methanesulfonylphenyl)-5,7-dimethoxy-4H-chromen-4-one **18k**

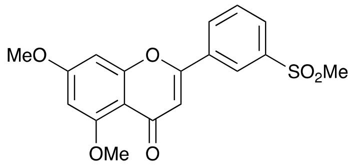

18k

In a microwave vial 2-chloro-5,7-dimethoxy-4H-chromen-4-one **15** (120 mg, 0.5 mmol, 1 eq) 3-(methylthio)phenylboronic acid (168 mg, 1 mmol, 2 eq), $Pd_2(dba)_3$ (14 mg, 3 mol%), CyJohnPhos (11 mg, 6 mol%) and Cs_2CO_3 (489 mg, 1.5 mmol, 3 eq) were suspended in dioxane (2.5 ml) under a nitrogen atmosphere. The reaction mixture was heated in the microwave to 150 °C for 30 min. After allowing to cool to rt, the crude mixture was filtered through Celite® and concentrated The residue was dissolved in H_2O (15 ml) and EtOAc (15 ml). The layers were separated and the aqueous layer was extracted two more times with EtOAc (2 × 25 ml). The combined organic layers were washed with H_2O (25 ml), dried over Mg_2SO_4 and concentrated. reversed phase column chromatography (H_2O:MeCN 0:1 - 1:0, 0.1% TFA) afforded *5,7-dimethoxy-2-(3'-methylthiophenyl)-4H-chromen-4-one* by as a light yellow solid(96 mg, 58%). m.p.: 169 – 171 °C; ν_{max} (ATR): 1639 (C=O), 1607, 1345, 1212, 1119, 816 cm^{-1}; δ_H ($CDCl_3$, 400 MHz): 7.76 - 7.68 (m, 1H, 2'-H), 7.65 - 7.56 (m, 1H, 6'-H), 7.45 - 7.30 (m, 2H, 5'-H, 4'-H), 6.67 (s, 1H, 3-H), 6.57 (d, *J* = 2.3 Hz, 1H, 8-H), 6.38 (d, *J* = 2.3 Hz, 1H, 6-H), 3.96 (s, 3H, OCH_3), 3.92 (s, 3H, OCH_3), 2.55 (s, 3H, SCH_3); δ_C ($CDCl_3$, 101 MHz): 177.7 (C-4), 164.3 (C-7), 161.0 (C-5), 160.3 (C-2), 160.0 (C-8a), 140.0 (C-3'), 132.3 (C-1'), 129.4 (C-5'), 129.1 (C-4'), 123.8 (C-2'), 122.7 (C-6'), 109.5 (C-3), 109.4 (C-4a), 96.4 (C-6), 93.0 (C-8), 56.6 (OCH_3), 56.0 (OCH_3), 15.9 (SCH_3); HRMS (ES^+) found $[M+H]^+$ 329.0842, $C_{18}H_{17}O_4S$ requires *M* 329.0842. To a solution of *5,7-dimethoxy-2-(3'-methylthiophenyl)-4H-chromen-4-one* (82 mg, 0.25 mmol, 1 eq) in MeOH (1.25 mL) and THF (1.25 mL) was added a solution of Oxone® (768 mg, 2.5 mmol, 10 eq) in H_2O (2 mL) dropwise at 0 °C. The resulting mixture was stirred at rt overnight. The solution was extracted with DCM (3 × 5 ml), the combined organic layers washed

with brine and dried over anhydrous Mg_2SO_4. The resulting solution was filtered and concentrated under reduced pressure to afford the title chromene as a colourless solid, (81 mg, 90%). m.p.: 239 – 241 °C (decomposition; ν_{max} (ATR): 1650 (C=O), 1611, 1349, 1299, 1144, 1121 cm^{-1}; δ_H ($CDCl_3$, 400 MHz): 8.55 - 8.44 (m, 1H, 2'-H), 8.18 - 8.02 (m, 2H, 4'-H, 6'-H), 7.80 - 7.67 (m, 1H, 5'-H), 6.75 (s, 1H, 3-H), 6.63 (d, *J* = 2.3 Hz, 1H, 8-H), 6.41 (d, *J* = 2.3 Hz, 1H, 6-H), 3.95 (d, *J* = 10.5 Hz, 6H, 2 × OCH_3), 3.13 (s, 3H, SO_2CH_3); δ_C ($CDCl_3$, 101 MHz): 177.2 (C-4), 164.6 (C-7), 161.1 (C-5), 160.0 (C-8a), 158.3 (C-2), 141.9 (C-3'), 133.4 (C-1'), 130.9 (ArC), 130.4 (C-5'), 129.7 (ArC), 125.0 (C-2'), 110.4 (C-3), 109.4 (C-4a), 96.8 (C-6), 93.0 (C-8), 56.6 (OCH_3), 56.1 (OCH_3), 44.7 (SO_2CH_3); HRMS (ES^+) found $[M+H]^+$ 361.0750, $C_{18}H_{17}O_6S$ requires *M* 361.0746.

2-(4'-Methanesulfonylphenyl)-5,7-dimethoxy-4H-chromen-4-one **18l**

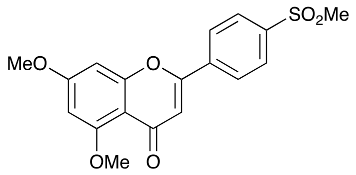

18l

In a microwave vial 2-chloro-5,7-dimethoxy-4H-chromen-4-one **15** (60 mg, 0.25 mmol, 1 eq) 4-(methylthio)phenylboronic acid (84 mg, 0.5 mmol, 2 eq), $Pd_2(dba)_3$ (7 mg, 3 mol%), CyJohnPhos (5 mg, 6 mol%) and Cs_2CO_3 (244 mg, 0.75 mmol, 3 eq) were suspended in dioxane (1.25 ml) under a nitrogen atmosphere. The reaction mixture was heated in the microwave to 150 °C for 30 min. After allowing to cool to rt, the crude mixture was filtered through Celite® and concentrated The residue was dissolved in H_2O (10 ml) and EtOAc (10 ml). The layers were separated and the aqueous layer was extracted two more times with EtOAc (2 × 25 ml). The combined organic layers were washed with H_2O (25 ml), dried over Mg_2SO_4 and concentrated. Purification by reversed phase column chromatography (H_2O:MeCN 0:1 - 1:0, 0.1% TFA) afforded *5,7-dimethoxy-2-(4'-methylthiophenyl)-4H-chromen-4-one* as a light yellow solid(55 mg, 67%). m.p.: 163 – 165 °C; ν_{max} (ATR): 1643 (C=O), 1599, 1347, 1119, 817 cm^{-1}; δ_H ($CDCl_3$, 400 MHz): 7.86 - 7.71 (m, 2H, 2'-H, 6'-H), 7.40 - 7.28 (m, 2H, 3'-H, 5'-H), 6.64 (s, 1H, 3-H), 6.56 (d, *J* = 2.3 Hz, 1H, 8-H), 6.38 (d, *J* = 2.3 Hz, 1H, 6-H), 3.96 (s, 3H, OCH_3), 3.91 (s, 3H, OCH_3), 2.54 (s, 3H, SCH_3).; δ_C ($CDCl_3$, 101 MHz): 177.8 (C-4), 164.2 (C-7), 161.0 (C-5), 160.6 (C-2), 160.0 (C-8a), 143.5 (C-4'), 127.8 (C-1'), 126.3 (C-3', C-6'), 125.9 (C-3', C-5'), 109.4 (C-4a), 108.4 (C-3), 96.3 (C-6), 93.0 (C-8), 56.6 (OCH_3), 55.9 (OCH_3), 15.2 (SCH_3); HRMS (ES^+) found $[M+H]^+$ 329.0843, $C_{18}H_{17}O_4S$ requires *M* 329.0848. To a solution of *5,7-dimethoxy-2-(4'-methylthiophenyl)-4H-chromen-4-one* (33 mg, 0.1 mmol, 1 eq) in MeOH (0.5 mL) and THF (0.5 mL) was added a solution of Oxone® (184 mg, 0.6 mmol, 6 eq) in H_2O (1 mL) dropwise at 0 °C. The resulting mixture was stirred at rt. After 24 h LC-MS analysis still showed the presence of starting material. Another 4 eq of Oxone® (123 mg, 4 mmol, 4 eq)

in H_2O (0.5 ml) were added and the mixture was stirred for another 24 h. The solution was then extracted with DCM (2 × 5 ml), the combined organic layers washed with brine and dried over anhydrous Mg_2SO_4. The resulting solution was filtered and concentrated under reduced pressure. Flash column chromatography (12 g silica gel, DCM:MeOH 1:0 - 19:1) afforded the title product (32 mg, 89%) as a colourless solid. m.p.: 299 – 300 °C (decomposition); ν_{max} (ATR): 1640 (C=O), 1343, 1148, 1120, 839, 775 cm^{-1}; δ_H ($CDCl_3$, 400 MHz): 8.15 - 8.00 (m, 4H, 2′-H, 6′-H, 3′-H, 5′-H), 6.75 (s, 1H, 3-H), 6.59 (d, *J* = 2.3 Hz, 1H, 8-H), 6.41 (d, *J* = 2.3 Hz, 1H, 6-H), 3.97 (s, 3H, OCH_3), 3.93 (s, 3H, OCH_3), 3.11 (s, 3H, CH_3); δ_C ($CDCl_3$, 101 MHz): 177.2 (C-4), 164.6 (C-7), 161.2 (C-5), 160.0 (C-8a), 158.4 (C-2), 142.6 (C-4′), 136.9 (C-1′), 128.2 (2 × ArC), 126.9 (2 × ArC), 111.1 (C-3), 109.5 (C-4a), 96.7 (C-6), 93.0 (C-8), 56.7 (OCH_3), 56.0 (OCH_3), 44.6 (CH_3); HRMS (ES^+) found $[M+H]^+$ 361.0749, $C_{18}H_{17}O_6S$ requires *M* 361.0746.

5,7-Dimethoxy-2-(pyrid-4′-yl)-4H-chromen-4-one[24] **18m**

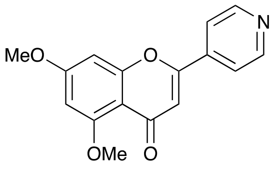

18m

2-Chloro-5,7-dimethoxy-4H-chromen-4-one **15** (60 mg, 0.25 mmol, 1 eq), 4-pyridineboronic acid (62 mg, 0.50 mmol, 2 eq), $Pd_2(dba)_3$ (4.6 mg, 2 mol%), Cy_3P (3.4 mg, 4.8 mol%), K_3PO_4 (0.33 ml of a 1.27 M solution in H_2O, 0.425 mmol, 1.7 eq) and dioxane (0.67 ml) were mixed in a reaction tube of a carousel reactor under a nitrogen atmosphere. The reaction mixture was heated to 100°C and stirred for 22 h. After allowing to cool to rt, the crude mixture was filtered through Celite® and concentrated the residue was dissolved in 1 N HCl (5 ml) and washed with DCM (3 ml). Then 1 N NaOH was added to the aqueous layer until it was basic and the product was extracted DCM (3 × 5 ml). The combined organic layers were dried over Na_2SO_4 and concentrated. Flash column chromatography (4 g silica gel, DCM:MeOH 1:0 - 19:1) yielded the title product (11 mg, 15%) as a colourless solid. m.p.: 229 - 230 °C; ν_{max} (ATR): 1652 (C=O), 1594, 1413, 1349, 1322, 1200, 1166, 1119, 822, 646 cm^{-1}; δ_H ($CDCl_3$, 400 MHz): 8.84 - 8.75 (m, 2H, 3′-H, 5′-H), 7.77 - 7.71 (m, 2H, 2′-H, 6′-H), 6.79 (s, 1H, 3-H), 6.60 (d, *J* = 2.3 Hz, 1H, 8-H), 6.42 (d, *J* = 2.3 Hz, 1H, 6-H), 3.98 (s, 3H, OCH_3), 3.95 (s, 3H, OCH_3); δ_C ($CDCl_3$, 100 MHz): 177.1 (C-4), 164.6 (C-7), 161.1 (C-5), 159.9 (C-2), 157.9 (C-8a), 150.9 (C-3′, C-5′), 139.1 (C-1′), 119.6 (C-2′, C-6′), 111.1 (C-3), 109.6 (C-4a), 96.6 (C-6), 93.0 (C-8), 56.6 (OCH_3), 56.0 (OCH_3); HRMS (ES^+) found $[M+H]^+$ 284.0903, $C_{16}H_{13}NO_4$ requires *M* 284.0923.

5,7-Dimethoxy-2-(6′-methylpyridazin-4′-yl)-4H-chromen-4-one **18n**

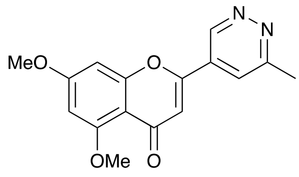

18n

To a solution of $[Ir(COD)OMe]_2$ (7.5 mg, 3 mol%), dtbpy (6 mg, 6 mol%) and B_2pin_2 (286 mg, 1.125 mmol, 3 eq) in degassed, dry MTBE (1.9 ml) under an argon atmosphere, 3-methylpyridazine (0.07 ml, 0.75 mmol, 2 eq) was added. The reaction was stirred at 50 °C overnight and subsequently concentrated. To the crude intermediate under an argon atmosphere were added $Pd(amphos)Cl_2$ (13.3 mg, 5 mol%), K_3PO_4 (159 mg, 0.75 mmol, 2 eq), 5,7-dimethoxy-2-chloro-4H-chromen-4-one **15** (90 mg, 0.375 mmol, 1 eq) and a 9:1 mixture of DMAC (0.9 ml) and H_2O (0.1 ml) as solvent. The mixture was stirred at 70 °C for 4 h and then filtered through Celite® and concentrated. The reaction mixture was diluted with H_2O and extracted with DCM (3 × 5ml). The organic extracts were collected, dried over $MgSO_4$ and concentrated. Flash column chromatography (12 g silica gel, hexane:(EtOAc:EtOH 3:1) 4:1 - 0:1) gave the title product (35 mg, 31 %) as a colourless solid. m.p.: 279 – 282 °C (decomposition); ν_{max} (ATR): 1652 (C=O), 1613, 1341, 1162, 1124, 831 cm^{-1}; δ_H ($CDCl_3$, 600 MHz): 9.45 (d, *J* = 2.1 Hz, 1H, 2'-H), 7.71 (d, *J* = 2.1 Hz, 1H, 6'-H), 6.82 (s, 1H, 3-H), 6.59 (d, *J* = 2.3 Hz, 1H, 8-H), 6.42 (d, *J* = 2.3 Hz, 1H, 6-H), 3.97 (s, 3H, OCH_3), 3.94 (s, 3H, OCH_3), 2.85 (s, 3H, CH_3); δ_C ($CDCl_3$, 151 MHz): 176.5 (C-4), 164.8 (C-7), 161.3 (C-5), 160.7 (C-5'), 159.9 (C-8a), 155.8 (C-2), 145.0 (C-2'), 130.0 (C-1'), 122.1 (C-6'), 112.2 (C-3), 109.7 (C-4a), 96.9 (C-6), 93.0 (C-8), 56.7 (OCH_3), 56.1 (OCH_3), 22.7 (CH_3); HRMS (ES^+) found $[M+H]^+$ 299.1014, $C_{16}H_{15}N_2O_4$ requires *M* 299.1032.

5,7-Dimethoxy-2-(5'-methyl-1',3',4'-oxadiazol-2'-yl)-4H-chromen-4-one **19**

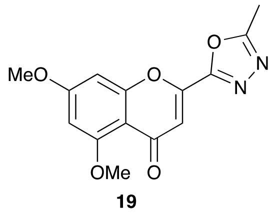

19

5,7-Dimethoxy-4-oxo-4H-chromene-2-carboxylic acid **13** (125 mg, 0.5 mmol, 1 eq), acethydrazide (41 mg, 0.55 mmol, 1.1 eq) and PyBOP (286 mg, 0.55 mmol, 1.1 eq) were dissolved in dry DMF (1.5 ml) and DIPEA (0.19 ml, 1.1 mmol, 2.2 eq) was added. The reaction mixture was stirred at rt overnight and then concentrated. The crude product and Burgess reagent (238 mg, 1 mmol, 2 eq) were dissolved in dry 1,2-dichloroethane under an argon atmosphere and heated to 140 °C for 3 h in a microwave. The title product (51 mg, 36%) was obtained by flash column chromatography (24 g silica gel, DCM:MeOH 1:0-19:1) and subsequent trituration with DCM as a colourless solid. m.p.: 231 – 234 °C (decomposition); ν_{max} (ATR): 1637 (C=O), 1608, 1569, 1315, 1136, 1073, 819 cm^{-1}; δ_H ($CDCl_3$, 700 MHz):

6.90 (s, 1H, 3-H), 6.64 (d, *J* = 2.3 Hz, 1H, 8-H), 6.40 (d, *J* = 2.3 Hz, 1H, 6-H), 3.95 (s, 3H, OCH_3), 3.89 (s, 3H, OCH_3), 2.67 (s, 3H, CH_3); δ_C ($CDCl_3$, 176 MHz): 176.0 (C-4), 165.4 (C-4'), 165.0 (C-7), 161.2 (C-5), 159.7 (C-8a), 159.1 (C-1'), 146.7 (C-2), 114.0 (C-3), 110.0 (C-4a), 97.1 (C-6), 93.2 (C-8), 56.6 (OCH_3), 56.1 (OCH_3), 11.3 (CH_3); HRMS (ES$^+$) found $[M+H]^+$ 289.0828, $C_{14}H_{13}N_2O_5$ requires *M* 289.0824.

5,7-Dimethoxy-2-(1',3'-oxazol-2'-yl)-4H-chromen-4-one **20**

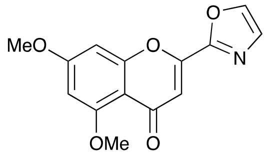

20

5,7-Dimethoxy-4-oxo-4H-chromene-2-carboxylic acid **41** (250 mg, 1 mmol, 1 eq), aminoacetaldehyde diethyl acetal (0.15 ml, 1.1 mmol, 1.1 eq) and PyBOP (572 mg, 1.1 mmol, 1.1 eq) were dissolved in dry DMF (3 ml) and DIPEA (0.38 ml, 2.2 mmol, 2.2 eq) was added. The reaction mixture was stirred at rt overnight and then concentrated. Purification by flash column chromatography (40 g silica gel, DCM:MeOH 1:0-19:1) afforded *N-(2',2'-Diethoxyethyl)-5,7-dimethoxy-4-oxo-4H-chromene-2-carboxamide* as a colourless solid (330 mg, 90%). m.p.: 106 – 108 °C (decomposition); ν_{max} (ATR): 3497 (NH), 2976, 1658 (C=O), 1608 (C=O), 1345, 1057, 826 cm^{-1}; δ_H ($CDCl_3$, 700 MHz): 7.04 (t, *J* = 6.0 Hz, 1H, NH), 6.96 (s, 1H, 3-H), 6.47 (d, *J* = 2.3 Hz, 1H, 8-H), 6.38 (d, *J* = 2.3 Hz, 1H, 6-H), 4.62 (t, *J* = 5.1 Hz, 1H, 2'-H), 3.93 (s, 3H, OCH_3), 3.90 (s, 3H, OCH_3), 3.81 - 3.73 (m, 2H, 1'-H), 3.67 - 3.51 (m, 4H, 2 × $OC\mathbf{H_2}CH_3$), 1.25 (t, *J* = 7.0 Hz, 6H, 2 × $OCH_2C\mathbf{H_3}$); δ_C ($CDCl_3$, 176 MHz): 177.1 (C-4), 164.7 (C-7), 161.3 (C-5), 159.6 (C=O), 159.1 (C-8a), 152.4 (C-2), 114.1 (C-3), 109.9 (C-4a), 100.6 (C-2'), 96.7 (C-6), 93.0 (C-8), 63.5 (C-1'), 56.6 (OCH_3), 56.0 (OCH_3), 42.4 (2 × $O\mathbf{C}H_2CH_3$), 15.5 (2 × $OCH_2\mathbf{C}H_3$); HRMS (ES$^+$) found $[M+H]^+$ 366.1552, $C_{18}H_{24}NO_7$ requires *M* 366.1553.

TFA (1.68 ml, 22 mmol, 110 eq) was added to a solution of *N-(2',2'-diethoxyethyl)-5,7-dimethoxy-4-oxo-4H-chromene-2-carboxamide* (73 mg, 0.2 mmol, 1 eq) in dry DCM (2.5 ml) under an argon atmosphere. The reaction mixture was stirred at rt for 2.5 h and then concentrated. The crude product and Burgess reagent (238 mg, 1 mmol, 2 eq) were dissolved in dry 1,2-dichloroethane under an argon atmosphere and heated to 140 °C for 3 h in a microwave. The reaction mixture was concentrated and the title oxazole (16 mg, 28%) obtained by flash column chromatography (12 g silica gel, Et_2O:MeOH 1:0-9:1) as a colourless solid. m.p.: 205 – 208 °C (decomposition); ν_{max} (ATR): 1657 (C=O), 1610, 1332, 1116, 828 cm^{-1}; δ_H ($CDCl_3$, 700 MHz): 7.84 (d, J = 0.8 Hz, 1H, 4'-H), 7.39 (d, J = 0.8 Hz, 1H, 3'), 6.94 (s, 1H, 3-H), 6.66 (d, J = 2.3 Hz, 1H, 8-H), 6.40 (d, J = 2.3 Hz, 1H, 6-H), 3.95 (s, 3H, OCH_3), 3.89 (s, 3H, OCH_3); δ_C ($CDCl_3$, 176 MHz): 176.6 (C-4), 164.7 (C-7), 161.1 (C-5), 159.8 (C-8a), 155.1 (C-1'), 149.1 (C-2), 140.9

(C-4'), 129.8 (C-3'), 112.5 (C-3), 110.0 (C-4a), 96.9 (C-6), 93.2 (C-8), 56.6 (OCH_3), 56.1 (OCH_3); HRMS (ES^+) found $[M+H]^+$ 274.0686, $C_{14}H_{12}NO_5$ requires *M* 274.0715.

5,7-Dimethoxy-4H-chromen-4-one-2-carbonitrile **21**

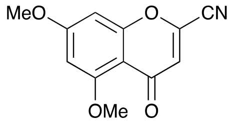

21

Phosphorus oxychloride (0.15 ml, 1.6 mmol, 1 eq) was added to a solution of *5,7-dimethoxy-4-oxo-4H-chromene-2-carboxylic acid* **13** (400 mg, 1.6 mmol, 1 eq) in dry DMF (3.2 ml). The reaction mixture was stirred 10 min at rt and another 50 min at 50 °C and then added to ammonium hydroxide solution (35% NH_3 in H_2O, 3.2 ml) at 0 °C and stirred for 1 h. The resulting precipitate was collected by filtration and dried. The title carboxamide (291 mg, 73%) was obtained by flash column chromatography (40 g silica gel, DCM:MeOH 1:0-9:1) afforded *5,7-dimethoxy-4H-chromen-4-one-2-carboxamide*[25] as a colourless solid. m.p.: 268 – 269 °C (decomposition) (lit.[25] m.p.: 268 – 270 °C); ν_{max} (ATR): 3519 (NH), 3385 (NH), 3144 (NH), 1652 (C=O), 1608 (C=O), 1330, 1069, 639 cm^{-1}; δ_H (d^6-DMSO , 700 MHz): 8.40 (s, 1H, NH), 8.07 (s, 1H, NH), 6.77 (d, *J* = 2.4 Hz, 1H, 8-H), 6.56 (s, 1H, 3-H), 6.54 (d, *J* = 2.4 Hz, 1H, 6-H), 3.88 (s, 3H, OCH_3), 3.83 (s, 3H, OCH_3); δ_C (d^6-DMSO, 176 MHz): 175.5 (C-4), 164.1 (C-7), 160.7 ($CONH_2$), 160.4 (C-5), 158.7 (C-8a), 153.1 (C-2), 112.2 (C-3), 108.8 (C-4a), 96.5 (C-6), 93.4 (C-8), 56.2 (OCH_3), 55.9 (OCH_3); HRMS (ES^+) found $[M+H]^+$ 250.0686, $C_{12}H_{12}NO_5$ requires *M* 250.0715. Trifluoroacetic anhydride (0.21 ml, 1.54 mmol, 2.2 eq) and pyridine (0.23 ml, 2.81 mmol, 4 eq) were added to a solution of 5,7-dimethoxy-4H-chromen-4-one-2-carboxamide **111** (175 mg, 0.70 mmol, 1 eq) in dry DMF (3.5 ml) at 0 °C under an argon atmosphere. The reaction mixture was stirred at rt overnight and concentrated. Flash column chromatography (24 g silica gel DCM:MeOH 1:0-19:1) afforded the title nitrile **21**(145 mg, 90%) as a colourless solid. m.p.: 188 – 190 °C (lit.[25] m.p.: 189 – 191 °C); ν_{max} (ATR): 2253 (CN), 1652 (C=O), 1608, 1341, 1157, 1078, 852, 799 cm^{-1}; δ_H ($CDCl_3$, 400 MHz): 6.66 (s, 1H, 3-H), 6.46 (d, *J* = 2.3 Hz, 1H, 8-H), 6.40 (d, *J* = 2.3 Hz, 1H, 6-H), 3.94 (s, 3H, OCH_3), 3.90 (s, 3H, OCH_3); δ_C ($CDCl_3$, 176 MHz): 174.2 (C-4), 165.3 (C-7), 161.3 (C-5), 159.9 (C-8a), 135.9 (C-2), 122.1 (C-3), 111.9 (CN), 110.3 (C-4a), 97.4 (C-6), 93.0 (C-8), 56.7 (OCH_3), 56.2 (OCH_3); HRMS (ES^+) found $[M+H]^+$ 232.0614, $C_{12}H_{10}NO_4$ requires *M* 232.0610.

5,7-Dimethoxy-2-(1H-1',2',3',4'-tetrazol-5'-yl)-4H-chromen-4-one[25] **22**

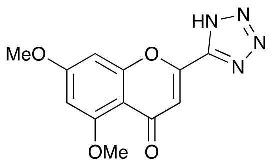

22

Dry toluene (0.75 ml) was added to *5,7-dimethoxy-4H-chromen-4-one-2-carbonitrile* **21** (75 mg, 0.33 mmol, 1 eq), NaN_3 (28 mg, 0.43 mmol, 1.3 eq) and $Et_3N.HCl$ (59 mg, 0.43 mmol, 1.3 eq) under an argon atmosphere. The reaction mixture was stirred at 95 °C for 48 h. H_2O (10 ml) was added, the aqueous phase was separated and the organic phase was further washed with H_2O. The combined aqueous layers were cooled to 0 °C, aqueous sodium nitrite solution (20 wt%, 0.1 ml) and sulfuric acid (20 wt%, 0.1 ml) were added and the solution was stirred for 30 min. The product was extracted with EtOAc (5 × 20 ml) and the combined organic layers were dried over Na_2SO_4 and concentrated. The title tetrazole (15 mg, 17%) was obtained by recrystallization from MeOH as a colourless solid. m.p.: 242 – 243 °C (lit.[25] m.p.: 244 – 245 °C); ν_{max} (ATR): 1651 (C=O), 1602, 1312, 1161, 1092, 834 cm^{-1}; δ_H (d^6-DMSO, 700 MHz): 6.80 (s, 1H, 3-H), 6.73 (d, *J* = 2.3 Hz, 1H, 8-H), 6.57 (d, *J* = 2.3 Hz, 1H, 6-H), 3.92 (s, 3H, OCH_3), 3.85 (s, 3H, OCH_3); δ_C (d^6-DMSO, 176 MHz): 174.6 (C-4), 164.2 (C-7), 160.5 (C-5), 159.0 (C-8a), 152.8 (C-1’), 149.7 (C-2), 111.8 (C-3), 108.8 (C-4a), 96.7 (C-6), 93.3 (C-8), 56.2 (OCH_3), 56.1 (OCH_3); HRMS (ES^+) found $[M+H]^+$ 275.0787, $C_{12}H_{11}N_4O_4$ requires *M* 275.0780.

5,7-Dimethoxy-2-(1’,3’-thiazol-2’-yl)-4H-chromen-4-one **23**

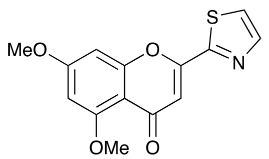

23

5,7-Dimethoxy-4H-chromen-4-one-2-carbonitrile **21** (1.340 mg, 5.8 mmol, 1 eq) was added to a slurry of $NaSH.xH_2O$ (70%, 1.392 mg, 17.4 mmol, 3 eq) and $MgCl_2.6H_2O$ (1.296 mg, 6.4 mmol, 1.1 eq) in DMF (10 ml) and stirred at 60 °C for 20 h. H_2O (50 ml) was added and the resulting precipitate was collected, washed with H_2O, resuspended in 1N HCl and stirred for 30 min. The precipitate was collected again, washed with $CHCl_3$ to remove unreacted starting material, and dried. Flash column chromatography (40 g silica gel DCM:MeOH 19:1-17:3) afforded *5,7-dimethoxy-4H-chromen-4-one-2-carbothioamide* (629 mg, 41%) as a yellow solid. m.p.: 247 - 249 °C (decomposition); ν_{max} (ATR): 3382, 3301, 3198, 1623 (C=O), 1595, 1416, 1224, 1125, 826 cm^{-1}; δ_H (d^6-DMSO, 700 MHz): 10.51 (s, 1H, NH), 10.06 (s, 1H, NH), 6.87 (s, 1H, 3-H), 6.87 (d, *J* = 2.3 Hz, 1H, 8-H), 6.54 (d, *J* = 2.3 Hz, 1H, 6-H), 3.88 (s, 3H, OCH_3), 3.83 (s, 3H, OCH_3); δ_C (d^6-DMSO, 176 MHz): 187.9 ($CSNH_2$) 175.3 (C-4), 164.2 (C-7), 160.3 (C-5), 158.2 (C-8a), 155.0 (C-2), 114.0 (C-3), 108.5 (C-4a), 96.6 (C-6), 93.5 (C-8), 56.2 (OCH_3), 55.9 (OCH_3); HRMS (ES^+)

found $[M+H]^+$ 266.0500, $C_{12}H_{12}NO_4S$ requires *M* 266.0487. *5,7-Dimethoxy-4H-chromen-4-one-2-carbothioamide* (121 mg, 0.46 mmol, 1 eq) and bromoacetaldehyde diethyl acetal (226 mg, 1.14 mmol, 2.5 eq) were suspended in THF (2 ml) and EtOH (0.57 ml) and a drop of H_2O was added. The reaction mixture was heated to 140 °C for 1 h in a microwave and subsequently concentrated. Purification by flash column chromatography (24 g silica gel, hexane:(EtOAc:EtOH 3:1) 9:1-2:3) afforded the title thiazole **23** as a light yellow solid (45 mg, 34%); m.p.: 169 – 171 °C; ν_{max} (ATR): 3107, 3067, 2945, 1644 (C=O), 1604, 1331, 1158, 1096, 829 cm^{-1}; δ_H ($CDCl_3$, 700 MHz): 8.01 (d, *J* = 3.1 Hz, 1H, 3’-H), 7.60 (d, *J* = 3.1 Hz, 1H, 4’-H), 7.02 (s, 1H, 3-H), 6.58 (d, *J* = 2.3 Hz, 1H, 8-H), 6.39 (d, *J* = 2.3 Hz, 1H, 6-H), 3.95 (s, 3H, OCH_3), 3.91 (s, 3H, OCH_3); δ_C ($CDCl_3$, 176 MHz): 176.9 (C-4), 164.5 (C-7), 161.2 (C-5), 159.7 (C-1’), 159.6 (C-8a), 154.6 (C-2), 145.1 (C-3’), 122.5 (C-4’), 109.9 (C-3), 109.9 (C-4a), 96.7 (C-6), 93.0 (C-8), 56.6 (OCH_3), 56.0 (OCH_3); HRMS (ES^+) found $[M+H]^+$ 290.0489, $C_{14}H_{12}NO_4S$ requires *M* 290.0487; accompanied by the 5-demethylated analogue *5-hydroxy-7-methoxy-2-(1’,3’-thiazol-2’-yl)-4H-chromen-4-one* (47 mg, 37%) m.p.: 204 - 206 °C; ν_{max} (ATR): 3112, 3087, 3059, 1658 (C=O), 1613, 1579, 1321, 1193, 1155, 968 cm^{-1}; δ_H ($CDCl_3$, 700 MHz): 12.60 (s, 1H, OH), 8.05 (d, *J* = 3.1 Hz, 1H, 3’-H), 7.66 (d, *J* = 3.1 Hz, 1H, 4’-H), 7.09 (s, 1H, 3-H), 6.52 (d, *J* = 2.3 Hz, 1H, 8-H), 6.40 (d, *J* = 2.3 Hz, 1H, 6-H), 3.90 (s, 3H, OCH_3); δ_C ($CDCl_3$, 176 MHz): 182.2 (C-4), 166.0 (C-7), 162.5 (C-5), 159.2 (C-1’), 157.5 (C-8a), 157.4 (C-2), 145.4 (C-3’), 123.3 (C-4’), 106.6 (C-3), 106.4 (C-4a), 98.8 (C-6), 93.0 (C-8), 56.1 (OCH_3); HRMS (ES^+) found $[M+H]^+$ 276.0338, $C_{13}H_{10}NO_4S$ requires *M* 276.0331.

5,7-Dimethoxy-2-(1’H-pyrazol-3’-yl)-4H-chromen-4-one **24**

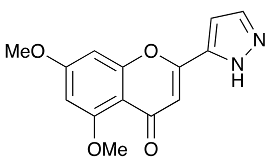

24

KOH (10 ml of a 3M solution in EtOH, 12 mmol, 6 eq) was added to a mixture of 2-hydroxy-4,6-dimethoxyacetophenone (392 mg, 2 mmol, 1 eq) and 1H-pyrrazole-5-carbaldehyde (0.19 ml, 2 mmol, 1 eq). The reaction mixture was stirred for 36 h at 50 °C and subsequently acidified to pH 2 with 3M HCl and extracted with DCM (3 × 10 ml). The combined organic layers were dried over Na_2SO_4 and concentrated. Iodine (15 mg, 0.3 mol%) was added to a stirred solution of the crude product in DMSO (3 ml) at 50 °C. Then the reaction mixture was heated to 140 °C and stirred for 2.5 h. After cooling to rt, H_2O (20 ml) and DCM (20 ml) were added. The layers were separated and the aqueous layer was extracted two more times with DCM (2 × 20 ml). The combined organic layers were washed with aqueous $Na_2S_2O_3$ solution (0.5%, 50 ml), dried over $MgSO_4$ and concentrated. The title product (62 mg, 11%) was obtained by flash column chromatography (24 g silica gel, Et_2O:MeOH 1:0-8:2) as a

colourless solid. m.p.: 255 – 257 °C (decomposition); ν_{max} (ATR): 3250 (NH), 1651 (C=O), 1606, 1324, 1132, 818, 766 cm^{-1}; δ_H (d^6-DMSO, 700 MHz 13.49 (s, 1H, NH), 7.99 - 7.91 (m, 1H, 4'-H), 6.95 - 6.88 (m, 1H, 5'-H), 6.75 (d, *J* = 2.4 Hz, 1H, 8-H), 6.56 (s, 1H, 3-H), 6.51 (d, *J* = 2.4 Hz, 1H, 6-H), 3.89 (s, 3H, OCH_3), 3.83 (s, 3H, OCH_3); δ_C (d^6-DMSO, 176 MHz): 175.3 (C-4), 163.6 (C-7), 160.3 (C-5), 159.1 (C-8a), 156.3 (C-2), 143.4 (C-1'), 130.6 (C-4'), 108.7 (C-4a), 107.2 (C-3), 104.3 (C-5'), 96.3 (C-6), 93.3 (C-8), 56.1 (OCH_3), 56.0 (OCH_3); HRMS (ES$^+$) found [M+H]$^+$ 273.0903, $C_{14}H_{13}N_2O_4$ requires *M* 273.0875.

5,7-Dimethoxy-2-(1'-methyl-1'H-pyrazol-5'-yl)-4H-chromen-4-one **25** & *5,7-Dimethoxy-2-(1'-methyl-1'H-pyrazol-3'-yl)-4H-chromen-4-one* **26**

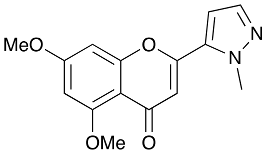

25

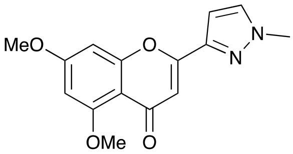

26

5,7-Dimethoxy-2-(1'H-pyrazol-3'-yl)-4H-chromen-4-one **24** (95 mg, 0.35 mmol, 1 eq) was added to a slurry of NaH (60% dispersed in mineral oil, 22 mg, 0.56 mmol, 1.6 eq) in dry DMF (1 ml) at 0 °C under an argon atmosphere and the resulting mixture was stirred for 15 min at 0 °C and another 15 min at rt. MeI (0.03 ml, 0.42 mmol, 1.2 eq) was added at 0 °C and the mixture was stirred another at rt overnight. H_2O (1 ml) was added and the product was extracted with DCM (3 × 2 ml). The combined organic layers were dried over Na_2SO_4 and concentrated. Flash column chromatography (24 g silica gel, hexane:(EtOAc:EtOH 3:1) 4:1-1:4) afforded the title products **25** (16 mg, 16%) and **26** (33 mg, 33%) as colourless solids. *5,7-Dimethoxy-2-(1'-methyl-1'H-pyrazol-5'-yl)-4H-chromen-4-one* **25**: m.p.: 180 – 181 °C; ν_{max} (ATR): 1651 (C=O), 1607, 1354, 1112, 840, 783 cm^{-1}; δ_H ($CDCl_3$, 400 MHz): 7.52 (d, J = 2.1 Hz, 1H, 4'-H), 6.69 (d, J = 2.1 Hz, 1H, 5'-H), 6.53 - 6.42 (m, 2H, 8-H, 3-H), 6.39 (d, J = 2.3 Hz, 1H, 6-H), 4.16 (s, 3H, CH_3), 3.95 (s, 3H, OCH_3), 3.90 (s, 3H, OCH_3); δ_C ($CDCl_3$, 151 MHz): 176.8 (C-4), 164.5 (C-7), 161.2 (C-5), 159.7 (C-8a), 153.0 (C-2), 138.8 (C-4'), 134.8 (C-1'), 112.0 (C-3), 109.3 (C-4a), 108.8 (C-5'), 96.6 (C-6), 92.8 (C-8), 56.6 (OCH_3), 56.0 (OCH_3), 39.8 (CH_3); HRMS (ES$^+$) found [M+H]$^+$ 287.1034, $C_{15}H_{15}N_2O_4$ requires *M* 287.1032. *5,7-Dimethoxy-2-(1'-methyl-1'H-pyrazol-3'-yl)-4H-chromen-4-one* **26**: m.p.: 169 – 171 °C; ν_{max} (ATR): 1650 (C=O), 1604, 1328, 1202, 1079, 772 cm^{-1}; δ_H ($CDCl_3$, 400 MHz): 7.44 (d, *J* = 2.3 Hz, 1H, 4'-H), 6.74 - 6.65 (m, 2H, 5'-H, 3-H), 6.62 (d, *J* = 2.3 Hz, 1H, 8-H), 6.36 (d, *J* = 2.3 Hz, 1H, 6-H), 4.01 (s, 3H, CH_3), 3.94 (s, 3H, OCH_3), 3.88 (s, 3H, OCH_3); δ_C ($CDCl_3$, 151 MHz): 177.6 (C-4), 164.0 (C-7), 161.0 (C-5), 159.9 (C-8a), 156.2 (C-2), 144.6 (C-1'), 131.9 (C-4'), 109.8 (C-4a), 109.0 (C-3), 105.6 (C-5'), 96.4 (C-6), 93.2 (C-8), 56.6 (OCH_3), 55.9 (OCH_3), 39.8 (CH_3); HRMS (ES$^+$) found [M+H]$^+$ 287.1035, $C_{15}H_{15}N_2O_4$ requires *M* 287.1032.

General procedure for 7-O acylation of chrysin

The appropriate acylating agent (1.3 equiv.) was added dropwise to a solution of chrysin (1.0 equiv.) and N,N-diisopropylethylamine (3 equiv.) in 20 ml of dry DMF at 0˚C. After stirring at room temperature for 1 h addition of water resulted in the formation of a bulky precipitate which was isolated by filtration.

5-Hydroxy-4-oxo-2-phenyl-4H-chromen-7-yl benzoate [26] **27**

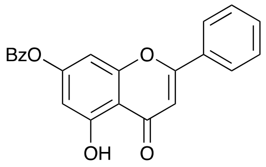

27

Following the standard procedure, using benzoyl chloride as the acylating agent, afforded the title ester as a yellow solid. The crude material was recrystallised from ethyl acetate/hexane to yield *5-hydroxy-4-oxo-2-phenyl-4-chromen-7-yl benzoate* as a yellow solid (4.33 g, 61 %). m.p. 168-171 °C (lit[27] 173-174); δ_H (400 MHz, $CDCl_3$): 12.77 (1 H, s, O*H*), 8.22-8.20 (2 H, dd, *J* = 8.2, 1.5 Hz, 2', 6'-*H*), 7.91 (2 H, dd, *J* = 8.2, 1.5 Hz, 3', 5'-*H*), 7.70-7.66 (1 H, m, 4'-*H*), 7.57-7.53 (5 H, m, Ar*H*), 7.01 (1 H, d, *J* = 2.1 Hz, 6-*H*), 6.76 (1 H, s, 3-*H*), 6.74 (1 H, d, *J* = 2.1 Hz, 8-*H*); δ_C (101 MHz, $CDCl_3$) 182.9 (*C*-4), 164.7 (*C*-1''), 164.2 (*C*-7), 162.0 (*C*-2), 156.8 (*C*-5), 156.3 (*C*-9), 134.0 (*C*-5''), 132.1 (*C*-4'), 131.0 (*C*-2''), 130.3 (*C*-3'', 7''), 129.1 (*C*-4'', 6''), 128.8 (*C*-1'), 128.7 (*C*-3', 5'), 126.4 (*C*-2', 6'), 109.0 (*C*-10), 106.2 (*C*-6), 105.6 (*C*-3), 101.2 (*C*-8); *m/z* (ES^+) 359 MH^+.

5-Hydroxy-4-oxo-2-phenyl-4H-chromen-7-yl acetate **28**

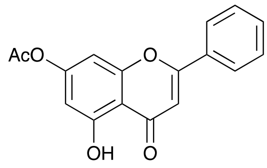

28

Following the standard procedure using acetic anhydride as the acylating agent afforded the title ester as a yellow solid (1.0 g, 35 %). m.p. 161-163 °C (lit[27] 168-169 °C); δ_H (600 MHz, $CDCl_3$): 12.73 (1 H, s, O***H***), 7.90-7.88 (2 H, m, 2', 6'-***H***), 7.57-7.52 (3 H, m, 3', 4', 5'-***H***), 6.86 (1 H, d, *J* = 2.0 Hz, 6-***H***), 6.73 (1 H, s, 3-***H***), 6.57 (1 H, d, *J* = 2.0 Hz, 8-***H***), 2.34 (3 H, s, 2''-$\boldsymbol{H}_3$); δ_C (151 MHz, $CDCl_3$): 182.8 (***C***-4), 168.3 (***C***-1''), 164.6 (***C***-2), 161.8 (***C***-5), 156.7 (***C***-7), 155.9 (***C***-9), 132.1 (***C***-4'), 130.9 (***C***-1'), 129.1 (***C***-2', 6'), 126.3 (***C***-3', 5'), 108.9 (***C***-10), 106.1 (***C***-6), 105.4 (***C***-3), 100.9 (***C***-8), 21.1 (***C***-2''); *m/z* (ES^+) 297 MH^+; data in agreement with that reported in the literature.[27]

5-Hydroxy-4-oxo-2-phenyl-4-chromen-7-yl hexanoate **29**

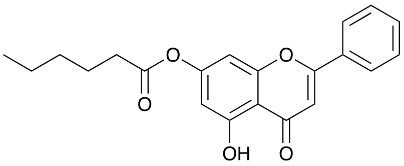

29

Synthesised according to the standard procedure using hexanoyl chloride. The crude material was recrystallised from ethyl acetate/hexane to yield *5-hydroxy-4-oxo-2-phenyl-4-chromen-7-yl hexanoate* as a yellow crystalline solid (138 mg, 50 %). R_f 0.62 (petrol/ethyl acetate, 7:3); m.p 137-138 **°**C. υ_{max} (ATR) 3080 (O-H), 2956, 1763 (C=O), 1648, 1613, 1588, 1489, 1264, 1127, 1097, 1023, 819, 768, 685, 676 cm^{-1}; δ_H (400 MHz, d_6-DMSO): 12.86 (1 H, s, O*H*), 8.15-8.13 (2 H, m, 2', 6'-*H*), 7.66-7.59 (3 H, m, 3', 4', 5'-*H*), 7.18 (1 H, s, 3-*H*), 7.13 (1 H, d, *J* = 2.0 Hz, 6-*H*), 6.68 (1 H, d, *J* = 2.0 Hz, 8-*H*), 2.65-2.61 (2 H, m, 2''-H_2), 1.69-1.65 (2 H, m, 3''-H_2), 1.36-1.34 (4 H, m, 4'', 5''-H_2) 0.93-0.91 (3 H, m, 6''-H_3); δ_C (101 MHz, d_6-DMSO) 181.6 (*C*-4), 170.0 (C-1''), 163.1 (*C*-2), 159.7 (*C*-5), 155.2 (*C*-7), 154.8 (*C*-9), 131.4 (*C*-4'), 129.3 (*C*-1'), 128.1 (*C*-3', 5'), 125.6 (*C*-2', 6'), 107.2 (*C*-10), 104.7 (*C*-6), 104.3 (*C*-3), 100.7 (*C*-8), 32.4 (*C*-2''), 29.5 (*C*-3''), 22.8 (*C*-4''), 20.7 (*C*-5''), 12.7 (*C*-6''); *m/z* HRMS (ES^+) [^{35}Cl]MH^+ calculated for $C_{21}H_{21}O_5$ 353.1389, found 353.1409 MH^+.

General procedure for 7-O alkylation of chrysin

To a stirred solution of chrysin (**2**, 1 eq, 1.97 mmol) in acetone (50 mL) was added potassium carbonate (0.5 eq) followed by the corresponding alkyl halide (5 eq, 9.85 mmol) and heated under reflux overnight. The reactions were concentrated to dryness and taken up into water (15 mL) and acidified with 2 M HCl (pH 1-2). The resulting aqueous solution was extracted with DCM (3 × 30 mL), dried ($MgSO_4$) and evaporated *in vacuo*. Purification by column chromatography on silica eluting with a suitable solvent system or recrystallisation gave the corresponding 5-hydroxy-7-alkoxy-2-phenylchromen-4-ones.

7-Benzyloxy-5-hydroxy-2-phenylchromen-4-one[28] **30**

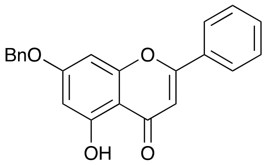

30

Obtained as a yellow solid (436 mg, 64 %). R_f 0.66 (petrol/ ethyl acetate, 7:3); m.p 175-178 °C (lit 177-178 °C); υ_{max} (ATR) 3063 (O-H), 1660 (C=O), 1613, 1583, 1447, 1350, 1160, 1028, 834, 819, 760, 673 cm^{-1}; δ_H (400 MHz, $CDCl_3$): 12.73 (1 H, s, O*H*), 7.89-7.87 (2 H, m, 2', 6'-*H*), 7.54-7.44 (3 H, m, 3', 4', 5'-*H*), 7.43-7.42 (5 H, m, Ar*H*), 6.67 (1 H, s, 3-*H*), 6.59 (1 H, d, *J* = 2.2 Hz, 6-*H*), 6.47 (1 H, d, *J* = 2.2 Hz, 8-*H*), 5.15 (2 H, s, 2''-H_2); δ_C (101 MHz, $CDCl_3$): 182.4 (*C*-4), 164.6 (*C*-7), 164.0 (*C*-2), 162.2 (*C*-5), 157.7 (*C*-9), 135.7 (*C*-3''), 131.8 (*C*-1'), 131.3 (*C*-4'), 129.0 (*C*-5'', 7''), 128.7 (*C*-3', 5'), 128.3 (*C*-6''), 127.4 (*C*-2',6'), 126.3 (*C*-4'', 8''), 105.9 (*C*-3), 105.9 (*C*-10), 98.9 (*C*-6), 93.5 (*C*-8), 70.4 (*C*-2''); *m/z* (ES$^+$) 345.1 MH$^+$; Elemental analysis [found C 76.42 %, H 4.68 %, N 0.06 %], (required C 76.73 %, H 4.68 %, N 0 %).

7-(Cyclopropylmethoxy)-5-hydroxy-2-phenylchromen-4-one **31**

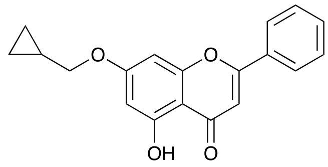

31

Obtained as a yellow solid (245 mg, 40 %). m.p 174-174 °C; Found C 74.08 %, H 5.21 %, $C_{19}H_{16}O_4$ requires C 74.01 %, H 5.23 %. υ_{max} (ATR) 3009 (O-H), 1660 (C=O), 1613, 1586, 1448, 1377, 1328, 1167, 998, 839, 825, 812, 762 cm^{-1}; δ_H (400 MHz, $CDCl_3$): 12.70 (1 H, s, O*H*), 7.89-7.87 (2 H, dd, *J* = 7.8, 1.8 Hz, 2', 6'-*H*), 7.54-7.52 (3 H, m, 3', 4', 5'-*H*), 6.67 (1 H, s, 3-*H*), 6.51 (1 H, d, *J* = 2.2 Hz, 6-*H*), 6.38 (1 H, d, *J* = 2.2 Hz, 8-*H*), 3.98 (2 H, d, *J* = 14.7 Hz, 1''-H_2), 1.34-1.28 (1H, m, 2''-*H*), 0.71-0.67 (2 H, m, CH_2), 0.41-0.39 (2 H, m, CH_2); δ_C (175 MHz, $CDCl_3$): 182.4 (*C*-4), 165.0 (*C*-7), 163.9 (*C*-2), 162.1 (*C*-5), 157.8 (*C*-9), 131.8 (*C*-1'), 131.3 (*C*-4'), 129.0 (*C*-3', 5'), 126.2 (*C*-2' 6'), 105.8 (*C*-3), 105.6 (*C*-10), 98.6 (*C*-6), 93.1 (*C*-8), 73.3 (*C*-1''), 9.9 (*C*-2''), 3.3 (*C*-3'', 4''); *m/z* HRMS (ES$^+$) MH$^+$ 309.1138, $C_{19}H_{17}O_4$ requires M, 309.1127.

7-(Cyclopentylmethoxy)-5-hydroxy-2-phenylchromen-4-one **32**

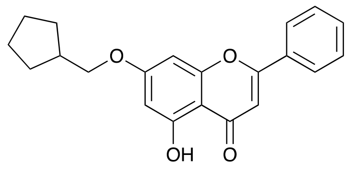

32

Obtained as a pale yellow solid (63 mg, 10 %). R_f 0.47 (petrol/ ethyl acetate, 7:3); m.p 137-139 °C; Found C 75.02 %, H 5.97 %, $C_{20}H_{20}O_4$ requires C 74.98 %, H 5.99 %. υ_{max} (ATR) 3063 (O-H), 1658 (C=O), 1621, 1611, 1587, 1450, 1331, 1163, 844, 821, 760 cm^{-1}; δ_H (400 MHz, $CDCl_3$): 12.69 (1 H, s, O*H*), 7.89

(2 H, d, *J* = 6.4 Hz, 2’, 6’-*H*), 7.54-7.52 (3 H, m, 3’, 4’, 5’-*H*), 6.67 (1 H, s, 3-*H*), 6.51 (1 H, d, *J* = 2.3 Hz, 6-*H*), 6.38 (1 H, d, *J* = 2.3 Hz, 8-*H*), 3.92 (2 H, d, *J* = 6.9 Hz, 1’’-H_2), 2.44-2.36 (1 H, m, 2’’-*H*), 1.89-1.84 (2 H, m, CH_2), 1.65-1.61 (4 H, m, 4’’, 5’’-H_2), 1.41-1.32 (2 H, m, CH_2); δ_C (101 MHz, $CDCl_3$): 182.4 (*C*-4), 165.3 (*C*-7), 163.9 (*C*-2), 162.1 (*C*-5), 157.8 (*C*-9), 131.7 (*C*-4’), 131.4 (*C*-1’), 129.0 (*C*-3’, 5’), 126.2 (*C*-2’, 4’), 105.8 (*C*-3), 105.5 (*C*-10), 98.6 (*C*-6), 93.1 (*C*-8), 72.8 (*C*-1’’), 38.8 (*C*-2’’), 29.4 (*C*-3’’, 6’’), 25.4 (*C*-4’’, 5’’); *m/z* (ES^+) 337 MH^+.

5-Hydroxy-7-pentoxy-2-phenylchromen-4-one **33**

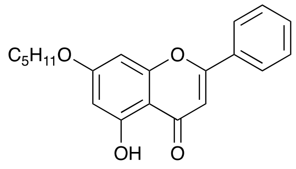

33

Obtained as a pale yellow solid (60 mg, 9 %). R_f 0.46 (petrol/ ethyl acetate, 7:3); m.p 167-170 °C; Found C 73.81 %, H 6.24 %, $C_{20}H_{20}O_4$ requires C 74.06 %, H 6.21 %; υ_{max} (ATR) 3077 (O-H), 1659 (C=O), 1614, 1587, 1333, 1168, 839, 823, 766, 674 cm^{-1}; δ_H (400 MHz, $CDCl_3$): 12.70 (1 H, s, O*H*), 7.89 (2 H, dd, *J* = 7.8, 1.9 Hz, 2’, 6’-*H*), 7.56-7.50 (3 H, m, 3’, 4’, 5’-*H*), 6.67 (1 H, s, 3-*H*), 6.50 (1 H, d, *J* = 2.2 Hz, 6-*H*), 6.37 (1 H, d, *J* =2.2 Hz, 8-*H*), 4.04 (2 H, t, *J* = 6.6 Hz, 1’’-H_2), 1.84-1.81 (2 H, m, 4’’-H_2), 1.46-1.41 (4 H, m, 2’’, 3’’-H_2), 0.95 (3 H, t, *J* = 7.0 Hz, 5’’-H_3); δ_C (101 MHz, $CDCl_3$): 182.4 (*C*-4), 165.2 (*C*-7), 163.9 (*C*-2), 162.1 (*C*-5), 157.8 (*C*-9), 131.7 (*C*-1’), 131.4 (*C*-4’), 129.0 (*C*-3’, 5’), 126.2 (*C*-2’, 6’), 105.8 (*C*-3), 105.6 (*C*-10), 98.6 (*C*-6), 93.1 (*C*-8), 68.7 (*C*-1’’), 28.6 (*C*-2’’), 28.0 (*C*-3’’), 22.4 (*C*-4’’), 14.0 (*C*-5’’); *m/z* HRMS (ES^+) [^{35}Cl]MH^+ found 325.1446 MH^+; $C_{20}H_{21}O_4$ requires *M*, 325.1440.

5-Hydroxy-2-phenyl-7-(tetrahydropyran-2’’-methoxy) chromen-4-one **34**

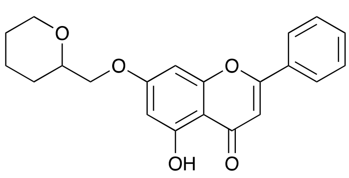

34

Obtained as a yellow solid (53 mg, 8 %). R_f 0.39 (petrol/ ethyl acetate, 7:3); m.p 119-124 °C; υ_{max} (ATR) 3072 (O-H), 1734, 1651 (C=O), 1605, 1581, 1504, 1435, 1350, 1169, 1160, 1102, 1003, 862, 825 cm^{-1}; δ_H (400 MHz, $CDCl_3$): 12.70 (1 H, s, O*H*), 7.88 (2 H, dd, *J* = 7.8, 1.8 Hz, 2’, 6’-*H*), 7.55-7.26 (3 H, m, 3’, 4’, 5’-*H*), 6.67 (1 H, s, 3-*H*), 6.54 (1 H, d, *J* = 2.2 Hz, 6-*H*), 6.41 (1 H, d, *J* =2.2 Hz, 8-*H*), 4.09-4.03 (2 H, m, OCH_2), 3.96-3.95 (1 H, m, 2“-*H*), 3.77-3.73 (1 H, m, 6’’-*H*), 3.56-3.50 (1 H, m, 6’’-*H*), 1.95-1.92 (1 H, m,

3''-*H*) 1.72-1.45 (5 H, m, 2 x CH_2, 3''-*H*); δ_C (101 MHz, $CDCl_3$) 182.4 (*C*-4), 164.9 (*C*-7), 164.0 (*C*-2), 162.1 (*C*-5), 157.7 (*C*-9), 131.8 (*C*-4'), 131.3 (*C*-1'), 129.0 (*C*-3', 5'), 126.3 (*C*-2', 6'), 107.0 (*C*-10), 105.9 (*C*-3), 98.7 (*C*-6), 93.3 (*C*-8), 75.5 (*C*-2''), 71.9 (OCH_2), 68.5 (*C*-6''), 28.1 (*C*-3''), 25.8 (*C*-4''), 23.0 (*C*-5''); *m/z* HRMS (ES^+) [^{35}Cl]MH^+ calculated for $C_{21}H_{21}O_5$ 353.1389, found 353.1383 MH^+.

5-Hydroxy-2-phenyl-7-(3-pyridylmethoxy) chromen-4-one **35**

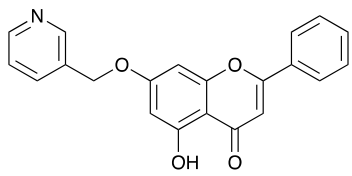

35

Obtained as a pale yellow solid (77 mg, 11 %). R_f 0.36 (hexane/ ethyl acetate, 7:3); m.p 167-170 °C; Found C 72.61 %, H 4.42 %, N 4.07 %, $C_{21}H_{15}NO_4$ requires C 73.04 %, H 4.38 %, N 4.06 %. υ_{max} (ATR) 3062 (O-H), 1660 (C=O), 1614, 1582, 1449, 1352, 1332, 1171, 1028, 835, 818 cm^{-1}; δ_H (400 MHz, $CDCl_3$): 12.76 (1 H, s, O*H*), 8.72 (1 H, d, *J* = 2.0 Hz, 2''-*H*), 8.64-8.62 (1 H, dt, *J* = 4.9, 2.0 Hz, 6''-*H*), 7.90-7.88 (2 H, m, 2', 6'-*H*), 7.81-7.79 (1 H, dt, 7.9, 2.0 Hz, 4''-*H*), 7.57-7.51 (3 H, m, 3', 4', 5'-*H*), 7.37 (1 H, dd, 7.9, 4.9 Hz, 5''-*H*), 6.69 (1 H, s, 3-*H*), 6.59 (1 H, d, *J* = 2.2 Hz, 6-*H*), 6.47 (1 H, d, *J* = 2.2 Hz, 8-*H*), 5.17 (2 H, s, OCH_2); δ_C (101 MHz, $CDCl_3$): 182.4 (*C*-4), 164.1 (*C*-7), 162.3 (*C*-2), 157.7 (*C*-5), 156.7 (*C*-9), 149.8 (*C*-2''), 148.9 (*C*-6''), 135.3 (Ar*C*), 131.9 (Ar*C*), 131.4 (*C*-3''), 131. 2 (*C*-1'), 129.1 (*C*-3', 5'), 126.3 (*C*-2', 6'), 123.6 (*C*-4'), 106.1 (*C*-3), 105.9 (*C*-10), 98.9 (*C*-6), 93.5 (*C*-8), 67.9 (OCH_2); *m/z* HRMS (ES^+) [^{35}Cl]MH^+ calculated for $C_{21}H_{16}NO_4$ 346.1079, found 346.1087 MH^+.

Synthesis of 7-amino-5-hydroxy-flavone derivatives

At 0°C, a solution of chrysin **1** (8.5 g, 33.4 mmol) in pyridine (80 mL) and CH_2Cl_2 (320 mL) was treated dropwise with trifluoromethanesulfonic anhydride (6.0 mL, 34.7 mmol). The solution was allowed to warm to room temperature and was then stirred at room temperature for 18 h. The solvents were removed *in vacuo* and the mixture was purified by column chromatography on silica eluting with hexane: ethyl acetate (7:3) to afford *5-hydroxy-4-oxo-2-phenyl-4H-chromen-7-yl trifluoromethanesulfonate* (7.2 g, 56 %). R_f 0.32 (hexane/ ethyl acetate, 4:1); m.p. 131-133 °C (lit[29] 129-130 °C); δ_H (400 MHz, $CDCl_3$): 12.96 (1 H, s, 5-O***H***), 7.91-7.89 (2 H, m, 3', 5'-***H***), 7.60-7.55 (3 H, m, 2', 4', 6'-***H***), 6.98 (1 H, d, *J* = 2.3 Hz, 6-***H***), 6.79 (1 H, s, 3-***H***), 6.74 (1 H, d, *J* = 2.3 Hz, 8-***H***); δ_C (101 MHz, $CDCl_3$): 182.6 (***C***=O), 165.3 (***C***-2), 162.5 (***C***-5), 156.6 (***C***-7), 153.2 (***C***-9), 132.5 (***C***-1'), 130.4 (***C***-4'), 129.3 (***C***-3', 5'), 126.5 (***C***-2', 6'), 110.4 (***C***F_3), 106.4 (***C***-10), 105.1 (***C***-3), 100.8 (***C***-6, 8); ^{19}F NMR (376 MHz, $CDCl_3$) ppm -73.58; *m/z* HRMS (ES^+) MH^+ calculated for $C_{16}H_{10}O_6F_3S$ 387.0150, found 387.0159 MH^+; data in

agreement with that reported in the literature.[29] 7-trifluoromethanesulfonyl-chrysin (1 eq) and the corresponding amine (4 eq) were added to a microwave vial and the vessel was sealed and heated under microwave irradiation for 20 min at 200 °C. The reaction was poured into water and extracted with DCM (3 × 20 mL), washed with brine (1 × 20 mL) and evaporated to yield the corresponding 7-aminochrysin derivatives following trituration with ether to remove trace impurities.

5-Hydroxy-7-((4-methoxybenzyl)amino)-2-phenyl-4H-chromen-4-one **36**

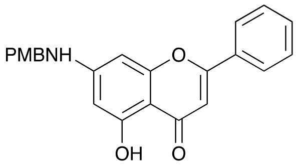

36

Obtained following the standard procedure as a yellow solid (75 mg, 77 %). m.p 211-213 °C; υ_{max} (ATR) 3020 (O-H), 1656 (C=O), 1651, 1608, 1581, 1513, 1440, 1392, 1220, 1183, 1033, 800, 756, 673 cm^{-1}; δ_H (700 MHz, $CDCl_3$): 7.85 (2 H, dd, *J* = 8.1, 1.6, Hz, 2', 6'-*H*), 7.51-7.50 (3 H, m, 3', 4', 5'-*H*), 7.28 (2 H, d, *J* = 8.6 Hz, 3'', 5''-*H*), 6.91 (2 H, d, *J* = 8.6 Hz, 2'', 6''-*H*), 6.58 (1 H, s, 3-*H*), 6.15 (1 H, d, *J* = 2.0 Hz, 6-*H*), 6.05 (1 H, d, *J* = 2.0 Hz, 8-*H*) 4.34 (2 H, s, CH_2), 3.81 (3 H, s, OCH_3); δ_C (175 MHz, $CDCl_3$): 181.6 (*C*=O), 163.0 (*C*-5), 161.8 (*C*-2), 159.1 (*C*-9), 158.4 (*C*-7), 154.0 (Ar*C*), 131.6 (Ar*C*), 131.4 (Ar*C*), 129.6 (Ar*C*), 128.9 (2 × Ar*C*), 128.7 (2 × Ar*C*), 126.1 (2 × Ar*C*), 114.2 (2 × Ar*C*), 105.7 (*C*-3), 103.4 (*C*-10), 95.9 (*C*-6), 89.5 (*C*-8), 55.3 (CH_3), 47.0 (CH_2); *m/z* HRMS (ES^+) MH^+ calculated for $C_{23}H_{20}NO_4$ 374.1392, found 374.1398 MH^+.

5-Hydroxy-2-phenyl-7-(pyrrolidinyl-1-yl)-4H-chromen-4-one [30] **37**

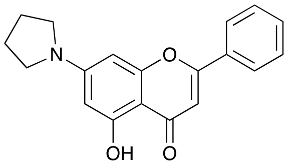

37

*O*btained following the standard procedure as a yellow solid (53 mg, 33 %). m.p 230-232 °C; υ_{max} (ATR) 3033 (O-H), 2848, 1660 (C=O), 1605, 1445, 1408, 1249, 1220, 1027, 861, 809, 761, 670 cm^{-1}; δ_H (700 MHz, $CDCl_3$): 7.87 (2 H, dd, *J* = 7.8, 1.8, Hz, 2', 6'-*H*), 7.51-7.49 (3 H, m, 3', 4', 5'-*H*), 6.58 (1 H, s, 3-*H*), 6.06 (1 H, d, *J* = 2.0 Hz, 6-*H*), 6.00 (1 H, d, *J* = 2.0 Hz, 8-*H*) 3.38-3.36 (4 H, m, 2'', 5''-H_2), 2.06-2.04 (4 H, m, 3'', 4''-H_2); δ_C (175 MHz, $CDCl_3$): 181.3 (*C*=O), 162.8 (*C*-7), 161.4 (*C*-2), 158.0 (*C*-5), 152.7 (*C*-9), 131.7

(*C*-1'), 131.3 (*C*-4'), 128.9 (*C*-2', 6'), 126.0 (*C*-3', 5'), 105.4 (*C*-3), 102.1 (*C*-10), 95.1 (*C*-6), 89.3 (*C*-8), 47.8 (*C*-2'', 5''), 25.3 (*C*-3'', 4''); *m/z* (ES^+) 307 MH^+.

5-Hydroxy-2-phenyl-7-(piperidin-1-yl)-4H-chromen-4-one[30] **38**

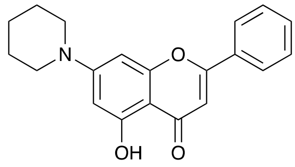

38

Obtained following the standard procedure as a yellow solid (103 mg, 62 %). R_f 0.57 (hexane/ ethyl acetate, 7:3); m.p 202-204 °C; υ_{max} (ATR) 3062 (O-H), 2936, 1661 (C=O), 1609, 1561, 1513, 1495, 1447, 1405, 1357, 1295, 1271, 1194, 1112, 1026, 903, 879, 833, 812, 764, 687, 637, 640, 618 cm^{-1}; δ_H (400 MHz, d_6-DMSO): 12.65 (1 H, br, s, 5-O*H*), 8.08 (2 H, dd, *J* = 8.1, 1.7, Hz, 2', 6'-*H*), 7.60-7.57 (3 H, m, 3', 4', 5'-*H*), 6.89 (1 H, s, 3-*H*), 6.65 (1 H, d, *J* = 2.3 Hz, 6-*H*), 6.33 (1 H, d, *J* = 2.3 Hz, 8-*H*) 3.46 (4 H, t, *J* = 5.3 Hz, 2'', 6''-H_2), 1.64-1.56 (6 H, m, 3'', 4'', 5''-H_2); δ_C (101 MHz, d_6-DMSO): 181.2 (*C*=O), 162.9 (*C*-7), 161.4 (*C*-2), 158.2 (*C*-5), 155.7 (*C*-9), 132.2 (*C*-1'), 131.4 (*C*-4'), 129.5 (*C*-2', 6'), 126.7 (*C*-3', 5'), 105.4 (*C*-3), 102.1 (*C*-10), 96.3 (*C*-6), 91.4 (*C*-8), 48.2 (*C*-2'', 6''), 25.3 (*C*-3'', 5''), 24.5 (*C*-4''); *m/z* HRMS (ES^+) MH^+ calculated for $C_{20}H_{20}NO_3$ 32.1443, found 322.1448 MH^+.

5-Hydroxy-7-morpholino-2-phenyl-4H-chromen-4-one [30] **39**

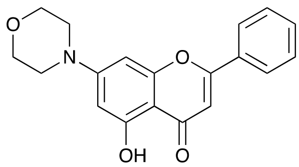

39

5-Hydroxy-7-morpholino-2-phenyl-4*H*-chromen-4-one was obtained as a yellow solid (82 mg, 49 %). R_f 0.66 (hexane/ ethyl acetate, 7:3); m.p 239-242 °C; υ_{max} (ATR) 3063 (O-H), 1665 (C=O), 1608, 1583, 1562, 1446, 1397, 1103, 1034, 817, 761, 686, 643, 632 cm^{-1}; δ_H (700 MHz, $CDCl_3$): 7.87 (2 H, dd, *J* = 8.0, 1.6, Hz, 2', 6'-*H*), 7.53-7.50 (3 H, m, 3', 4', 5'-*H*), 6.61 (1 H, s, 3-*H*), 6.40 (1 H, d, *J* = 2.2 Hz, 6-*H*), 6.29 (1 H, d, *J* = 2.2 Hz, 8-*H*) 3.86 (4 H, t, *J* = 5.0 Hz, 2'', 6''-H_2), 3.34 (4 H, t, *J* = 5.0 Hz, 3'', 6''-H_2); δ_C (175 MHz, $CDCl_3$): 181.8 (*C*=O), 163.5 (*C*-7), 161.7 (*C*-2), 158.0 (*C*-5), 155.9 (*C*-9), 131.6 (*C*-1'), 129.0 (*C*-2', 4', 6'), 126.1 (*C*-3', 5'), 105.8 (*C*-3), 104.0 (*C*-10), 97.0 (*C*-6), 91.6 (*C*-8), 66.3 (*C*-3'', 5''), 47.3 (*C*-2'', 4''); *m/z* HRMS (ES^+) MH^+ calculated for $C_{19}H_{18}NO_4$ 324.1236, found 324.1227 MH^+.

Synthesis of 2''-(7-methoxy-4-oxo-2-phenylchromen-5-oxy)acetate derivatives

A mixture of *5-hydroxy-7-methoxy-2-phenyl-4H-chromen-4-one* (101 mg, 0.375 mmol, 1.5 eq), appropriate triflate or halide (0.25 mmol, 1 eq) and K_2CO_3 (104 mg, 0.75 mmol, 3 eq) in dry MeCN (1 ml) under a nitrogen atmosphere was stirred at rt overnight. H_2O (2 ml) was added to the reaction mixture. The aqueous phase was extracted with EtOAc (3 × 2 ml), the organic extracts collected, washed with brine, dried over $MgSO_4$ and concentrated. The crude product together with LiOH.H_2O (31 mg, 0.75 mmol, 3 eq) was stirred in a 1:1 mixture of H_2O (3 ml) and THF (3 ml) at rt for 3 h. The mixture was acidified to pH 2-3 with 1N HCl and the aqueous phase was extracted with DCM (3 × 5 ml). The combined organic layers were dried over $MgSO_4$ and concentrated. Flash column chromatography (12 g silica gel, DCM:MeOH 1:0 - 19:1) yielded in the product.

2''-(7-Methoxy-4-oxo-2-phenylchromen-5-oxy) acetic acid **40**

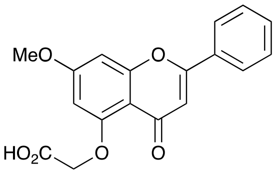

40

Obtained, using ethyl bromoacetate, as a white solid (47 mg, 51 %). υ_{max} (ATR) 3076 (O-H), 1734 (C=O), 1627 (C=O), 1595, 1450, 1371, 1352, 1190, 1168, 1129, 1115, 1051, 1024, 913, 870, 826, 766, 677, 606 cm^{-1}; δ_H (700 MHz, d_6-DMSO): 12.19 (1 H, br, s, O*H*), 8.04 (2 H, dd, *J* = 8.1, 1.6 Hz, 2', 6'-*H*), 7.55-7.54 (3 H, m, 3', 4', 5'-*H*), 6.93 (1 H, d, *J* = 2.3 Hz, 6-*H*), 6.81 (1 H, s, 3-*H*), 6.42 (1 H, d, *J* = 2.3 Hz, 8-*H*), 4.81 (2 H, s, 2''-H_2), 3.86 (3 H, s, OCH_3); δ_C (175 MHz, d_6-DMSO): 176.3 (*C*=O), 170.1 (*C*=O), 164.0 (*C*-7), 160.4 (*C*-2), 159.5 (*C*-5), 158.9 (*C*-9), 131.9 (*C*-1'), 131.2 (*C*-4'), 129.4 (*C*-2', 6'), 126.4 (*C*-3' 5'), 109.0 (*C*-10), 108.5 (*C*-3), 98.8 (*C*-6), 94.7 (*C*-8), 66.4 (*C*-2''), 56.5 (O*C*H_3); *m/z* HRMS (ES$^+$) MH$^+$ calculated from $C_{18}H_{15}O_6$ 327.0869, found 327.0872 MH$^+$.

2''-(7-Methoxy-4-oxo-2-phenylchromen-5-oxy) propanoic acid **41**

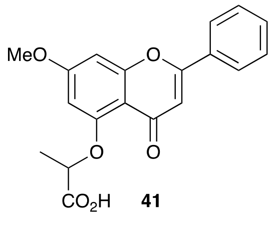

41

Obtained using ethyl 2-bromopropionate as an off white solid (38 mg, 41 %). m.p 170-174 °C; υ_{max} (ATR) 3070 (O-H), 1741 (C=O), 1631 (C=O), 1589, 1490, 1449, 1360, 1298, 1204, 1165, 1125, 1053, 959, 913, 886, 802, 768, 647, 601 cm^{-1}; δ_H (700 MHz, d$_6$-DMSO): 13.16 (1 H, s, O*H*), 8.04 (2 H, dd, *J* = 8.2, 1.6 Hz, 2', 6'-*H*), 7.56-7.54 (3 H, m, 3', 4', 5'-*H*), 6.94 (1 H, d, *J* = 2.3 Hz, 6-*H*), 6.80 (1 H, s, 3-*H*), 6.40 (1 H, d, *J* = 2.3 Hz, 8-*H*), 4.90 (1 H, q, *J* = 6.8 Hz, 2''-*H*), 3.86 (3 H, s, OC*H*$_3$), 1.56 (3 H, d, *J* = 6.5 Hz, 3''-*H*$_3$); δ_C (175 MHz, d$_6$-DMSO): 176.5 (*C*=O), 173.0 (*C*=O), 164.0 (*C*-7), 160.5 (*C*-2), 159.5 (*C*-5), 158.5 (*C*-9), 132.0 (*C*-1'), 131.1 (*C*-4'), 129.4 (*C*-2', 6'), 126.4 (*C*-3', 5'), 109.2 (*C*-10), 108.4 (*C*-3), 99.9 (*C*-6), 95.0 (*C*-8), 74.5 (*C*-2''), 56.5 (O*C*H$_3$), 18.7 (*C*-3''); *m/z* HRMS (ES$^+$) MH$^+$ calculated for $C_{19}H_{17}O_6$ 341.1025, found 341.1026 MH$^+$.

2-[(7'-Methoxy-4'-oxo-2'-phenyl-4'H-chromen-5'-yl)oxy]heptanoic acid **42**

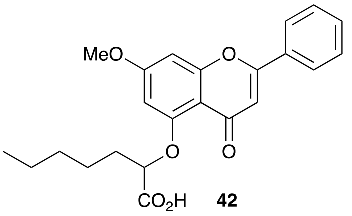

42

Obtained using *ethyl 2-(((trifluoromethyl)sulfonyl)oxy)heptanoate* (77 mg, 0.25 mnmol, 1 eq) as a colourless solid(61 mg, 62 %). m.p.: 104 – 105 °C; ν_{max} (ATR): 2929, 2860, 1743 (C=O), 1630 (C=O), 1592, 1354, 1161, 1110, 768 cm^{-1}; δ_H (CDCl$_3$, 400 MHz): 13.67 (br. s, 1H, COOH), 7.95 - 7.84 (m, 2H, 2''-H, 6''-H), 7.60 - 7.47 (m, 3H, 4''-H, 3''-H, 5''-H), 6.76 (s, 1H, 3'-H), 6.73 (d, *J* = 2.2 Hz, 1H, 8'-H), 6.47 (d, *J* = 2.2 Hz, 1H, 6'-H), 4.83 (t, *J* = 5.3 Hz, 1H, 2-H), 3.94 (s, 3H, OCH$_3$), 2.23 - 2.07 (m, 2H, 3-H$_2$), 1.66 - 1.45 (m, 2H, 4-H$_2$), 1.42 - 1.22 (m, 4H,2 × CH$_2$), 0.89 (t, *J* = 7.1 Hz, 3H, 7-H$_3$); δ_C (CDCl$_3$, 101 MHz): 179.0 (C-4'), 172.5 (C-1), 164.9 (C-7'), 162.9 (C-2'), 159.6 (C-8a'), 159.0 (C-5'), 132.1 (C-4''), 131.1 (C-1''), 129.3 (C-3'', C-5''), 126.4 (C-2'', C-6''), 109.6 (C-4a'), 108.4 (C-3'), 101.4 (C-6'), 95.2 (C-8'), 82.0 (C-2), 56.2 (OCH$_3$), 33.0 (C-3), 31.7 (CH$_2$), 24.5 (C-4), 22.6 (CH$_2$), 14.1 (C-7); HRMS (ES$^+$) found [M+H]$^+$ 397.1663, $C_{23}H_{25}O_6$ requires *M* 397.1651.

2-[(7'-Methoxy-4'-oxo-2'-phenyl-4'H-chromen-5'-yl)oxy]nonanoic acid **43**

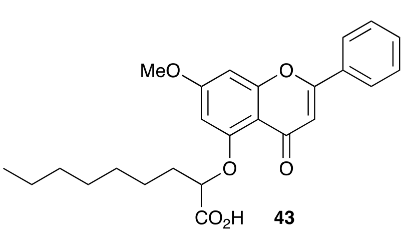

43

Obtained using *ethyl 2-(((trifluoromethyl)sulfonyl)oxy)nonanoate* (84 mg, 0.25 mnmol, 1 eq) as a colourless solid (65 mg, 61 %). m.p.: 143 – 145 °C; ν_{max} (ATR): 2926, 2852, 1739 (C=O), 1610 (C=O), 1591, 1357, 1168, 766 cm^{-1}; δ_H (CDCl$_3$, 400 MHz): 13.59 (br. s, 1H, COOH), 7.96 - 7.83 (m, 2H, 2''-H, 6''-

H), 7.61 - 7.47 (m, 3H, 4''-H, 3''-H, 5''-H), 6.76 (s, 1H, 3'-H), 6.73 (d, *J* = 2.2 Hz, 1H, 8'-H), 6.47 (d, *J* = 2.2 Hz, 1H, 6'-H), 4.83 (t, *J* = 5.3 Hz, 1H, 2-H), 3.94 (s, 3H, OCH_3), 2.25 - 2.06 (m, 2H, 3-H_2), 1.67 - 1.46 (m, 2H, 4-H_2), 1.42 - 1.21 (m, 8H, 4 × CH_2), 0.87 (t, *J* = 6.7 Hz, 3H, 9-H_3); δ_C ($CDCl_3$, 101 MHz): 179.0 (C-4'), 172.6 (C-1), 164.9 (C-7'), 162.9 (C-2'), 159.6 (C-8a'), 159.0 (C-5'), 132.1 (C-4''), 131.1 (C-1''), 129.3 (C-3'', C-5''), 126.4 (C-2'', C-6''), 109.6 (C-4a'), 108.4 (C-3'), 101.4 (C-6'), 95.2 (C-8'), 82.0 (C-2), 56.2 (OCH_3), 33.0 (C-3), 31.9 (CH_2), 29.5 (CH_2), 29.2 (CH_2), 24.8 (C-4), 22.8 (CH_2), 14.2 (C-9); HRMS (ES^+) found $[M+H]^+$ 425.1967, $C_{25}H_{29}O_6$ requires *M* 425.1964.

2-[(7'-Methoxy-4'-oxo-2'-phenyl-4'H-chromen-5'-yl)oxy]undecanoic acid **44**

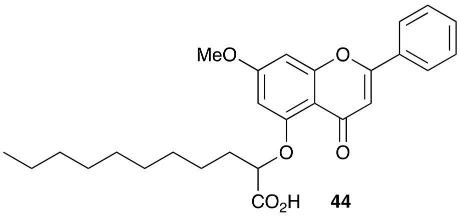

44

Obtained using *ethyl 2-(((trifluoromethyl)sulfonyl)oxy)undecanoate* (91 mg, 0.25 mnmol, 1 eq) as a colourless solid (90 mg, 80 %). m.p.: 96 – 98 °C; ν_{max} (ATR): 2914, 2851, 1728 (C=O), 1617 (C=O), 1597, 1363, 1165, 1111, 855 cm^{-1}; δ_H ($CDCl_3$, 400 MHz): 13.68 (br. s, 1H, COOH), 7.95 - 7.82 (m, 2H, 2''-H, 6''-H), 7.64 - 7.46 (m, 3H, 4''-H, 3''-H, 5''-H), 6.76 (s, 1H, 3'-H), 6.73 (d, *J* = 2.2 Hz, 1H, 8'-H), 6.47 (d, *J* = 2.2 Hz, 1H, 6'-H), 4.83 (t, *J* = 5.3 Hz, 1H, 2-H), 3.94 (s, 3H, OCH_3), 2.30 - 1.99 (m, 2H, 3-H_2), 1.70 - 1.44 (m, 2H, 4-H_2), 1.43 - 1.16 (m, 12H, 6 × CH_2), 0.87 (t, *J* = 6.6 Hz, 3H, 11-H_3); δ_C ($CDCl_3$, 101 MHz): 179.0 (C-4'), 172.5 (C-1), 164.9 (C-7'), 162.9 (C-2'), 159.6 (C-8a'), 159.0 (C-5'), 132.1 (C-4''), 131.1 (C-1''), 129.3 (C-3'', C-5''), 126.4 (C-2'', C-6''), 109.6 (C-4a'), 108.4 (C-3'), 101.4 (C-6'), 95.2 (C-8'), 82.0 (C-2), 56.2 (OCH_3), 33.0 (C-3), 32.0 (CH_2), 29.6 (CH_2), 29.6 (CH_2), 29.5 (CH_2), 29.4 (CH_2), 24.8 (C-4), 22.8 (CH_2), 14.3 (C-11) ; HRMS (ES^+) found $[M+H]^+$ 453.2285, $C_{27}H_{33}O_6$ requires *M* 453.2277.

2''-(7-Methoxy-4-oxo-2-phenylchromen-5-oxy)dodecanoic acid **45**

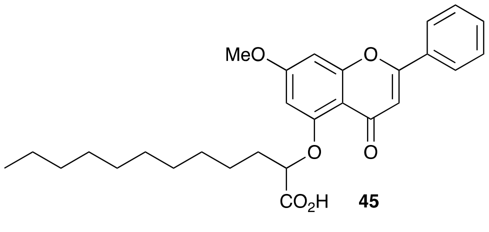

45

Obtained using *methyl 2-bromododecanoate* (294 mg, 1 mmol) as a colourless solid. (222 mg, 95%). m.p.: 98 - 99 °C; ν_{max} (ATR): 2917 (COOH), 2848, 1748 (C=O), 1640 (C=O), 1598, 1353, 1194, 1159, 1107 cm^{-1}; δ_H ($CDCl_3$, 400 MHz): 7.95 - 7.88 (m, 2H, 2''-H, 6''-H), 7.62 - 7.51 (m, 3H, 3''-H, 4''-H, 5''-H), 6.79 (s, 1H 3'-H), 6.75 (d, *J* = 2.2 Hz, 1H, 8'-H), 6.50 (d, *J* = 2.2 Hz, 1H, 6'-H), 4.86 (t, *J* = 5.3 Hz, 1H, 2-H), 3.96

(s, 3H, OCH_3), 2.23 - 2.11 (m, 2H, 3-H_2), 1.67 – 1.51 (m, 2H, 4-H_2), 1.43 - 1.23 (m, 14H, 7 × CH_2), 0.89 (t, J=6.9 Hz, 3H, 12-H_3); δ_C ($CDCl_3$, 100 MHz): 179.0 (C-4'), 172.5 (C-1), 164.9 (C-7'), 162.9 (C-2'), 159.6 (C-8a'), 159.0 (C-5'), 132.1 (C-4''), 131.1 (C-1''), 129.3 (C-3'', C-5''), 126.4 (C-2'', C6''), 109.6 (C-4a'), 108.4 (C-3'), 101.4 (C-6'), 95.3 (C-8'), 82.0 (C-2), 56.2 (OCH_3), 33.1 (C-3), 32.0 (CH_2), 29.7 (CH_2), 29.7 (CH_2), 29.6 (CH_2), 29.5 (CH_2), 29.4 (CH_2), 24.8 (C-4), 22.8 (CH_2), 14.3 (C-12)*;* m/z LC-MS (ES^+) 467 $[M+H]^+$.*m/z* HRMS (ES^+) MH^+ calculated for $C_{28}H_{35}0_6$ 467.2434, found 467.2463 MH^+.

2-[(7'-Methoxy-4'-oxo-2'-phenyl-4'H-chromen-5'-yl)oxy]tetradecanoic acid **46**

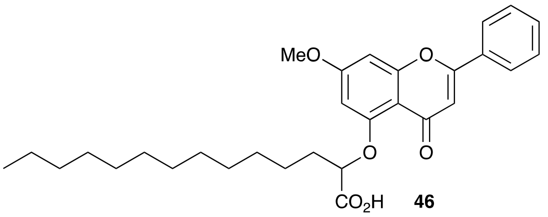

Obtained using ethyl 2-(((trifluoromethyl)sulfonyl)oxy)-tetradecanoate (101 mg, 0.25 mmol, 1 eq) as a colourless solid (0.103 g, 83 %). m.p.: 99 – 101 °C; ν_{max} (ATR): 2916, 2848, 1745 (C=O), 1639 (C=O), 1597, 1355, 1194, 1159 cm^{-1}; δ_H ($CDCl_3$, 400 MHz): 13.68 (br. s, 1H, COOH), 7.93 - 7.86 (m, 2H, 2''-H, 6''-H), 7.60 - 7.49 (m, 3H, 4''-H, 3''-H, 5''-H), 6.76 (s, 1H, 3'-H), 6.73 (d, *J* = 2.2 Hz, 1H, 8'-H), 6.47 (d, *J* = 2.2 Hz, 1H, 6'-H), 4.83 (t, *J* = 5.3 Hz, 1H, 2-H), 3.94 (s, 3H, OCH_3), 2.22 - 2.07 (m, 2H, 3-H_2), 1.66 - 1.46 (m, 2H, 4-H_2), 1.45 - 1.10 (m, 12H, 6 × CH_2), 0.87 (t, *J* = 6.8 Hz, 3H, 14-H_3); δ_C ($CDCl_3$, 101 MHz): 179.0 (C-4'), 172.6 (C-1), 164.9 (C-7'), 162.9 (C-2'), 159.6 (C-8a'), 159.0 (C-5'), 132.1 (C-4''), 131.0 (C-1''), 129.3 (C-3'', C-5''), 126.4 (C-2'', C-6''), 109.5 (C-4a'), 108.3 (C-3'), 101.4 (C-6'), 95.2 (C-8'), 82.0 (C-2), 56.2 (OCH_3), 33.0 (C-3), 32.1 (CH_2), 29.8 (2 × CH_2), 29.7 (CH_2), 29.7 (CH_2), 29.6 (CH_2), 29.5 (CH_2), 29.5 (CH_2), 24.8 (C-4), 22.8 (CH_2), 14.3 (C-14); HRMS (ES^+) found $[M+H]^+$ 495.2762, $C_{30}H_{39}O_6$ requires *M* 495.2747.

2''-(7-Methoxy-4-oxo-2-phenylchromen-5-oxy)-3''-methylbutanoic acid **47**

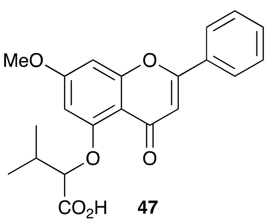

Obtained, using *ethyl 2-bromo-3-methylbutanoate,* as a white solid (12 mg, 65 %). m.p 193-196 °C; υ_{max} (ATR) 3086 (O-H), 2942, 1732 (C=O), 1620 (C=O), 1587, 1573, 1495, 1391 1353, 1208, 1159, 1113, 1100, 1019, 984, 882, 827, 773, 740, 679, 615 cm^{-1}; δ_H (700 MHz, d_6-DMSO): 8.06 (2 H, dd, *J* = 8.0, 1.5 Hz, 2', 6'-*H*), 7.59-7.56 (3 H, m, 3', 4', 5'-*H*), 6.88 (1 H, d, *J* = 2.3 Hz, 6-*H*), 6.77 (1 H, s, 3-*H*), 6.30 (1 H, d, *J* = 2.3 Hz, 8-*H*) 4.59 (1 H, br, s, 2''-*H*), 3.87 (3 H, s, OCH_3), 2.27 (1 H, septd, *J* = 6.8, 4.3 Hz, 3''-*H*), 1.11

(3 H, d, *J* = 6.8 Hz, C*H*$_3$), 1.08 (3 H, d, *J* = 6.8 Hz, C*H*$_3$); δ_C (175 MHz, $CDCl_3$): 176.2 (*C*=O), 163.9 (*C*=O), 160.2 (*C*-5) , 159.6 (*C*-2, 7), 159.3 (*C*-9), 131.9 (*C*-1'), 131.3 (*C*-4'), 129.5 (*C*-3', 5'), 126.4 (*C*-2', 6'), 109.0 (*C*-10), 108.3 (*C*-3), 98.1 (*C*-6, 2''), 94.0 (*C*-8), 56.4 (O*C*H$_3$), 31.4 (*C*-3''), 19.2 (*C*H$_3$), 17.6 (*C*H$_3$); *m/z* HRMS (ES$^+$) MH$^+$ calculated for $C_{21}H_{21}O_6$ 369.1338, found 369.1356 MH$^+$.

2''-(7-Methoxy-4-oxo-2-phenylchromen-5-oxy)phenylacetic acid **48**

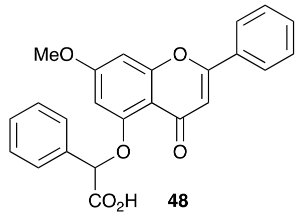

48

Obtained, using *ethyl 2-bromo-2-phenylacetate,* as a white solid (96 mg, 100 %). m.p 227-228 °C; υ_{max} (ATR) 3069 (O-H), 1731 (C=O), 1632 (C=O), 1614, 1573, 1494, 1448, 1357, 1283, 1206, 1160, 1111, 1020, 906 840, 827, 766, 689, 672 cm^{-1}; δ_H (700 MHz, d_6-DMSO): 13.37 (1 H, s, O*H*), 8.08 (2 H, dd, *J* = 7.5, 2.1 Hz, Ar*H*), 7.81-7.79 (3 H, m, Ar*H*), 7.60 (2 H, dd, *J* = 5.0, 2.4 Hz, Ar*H*), 7.45-7.43 (3 H, m, Ar*H*), 6.98 (1 H, d, *J* = 2.2 Hz, 6-*H*), 6.84 (1 H, s, 3-*H*), 6.65 (1 H, d, *J* = 2.2 Hz, 8-*H*), 6.03 (1 H, s, 2''-*H*), 3.89 (3 H, s, OC*H*$_3$); δ_C (175 MHz, d_6-DMSO): 176.3 (*C*=O), 170.9 (*C*=O), 163.9 (*C*-7), 160.5 (*C*-2), 159.7 (*C*-5), 157.9 (C-9), 132.0 (Ar*C*), 131.2 (Ar*C*), 129.5 (2 × Ar*C*), 128.8 (3 × Ar*C*), 127.5 (3 × Ar*C*), 126.5 (2 × Ar*C*), 109.4 (*C*-10), 108.7 (*C*-3), 99.2 (*C*-6), 94.8 (*C*-8), 78.8 (*C*-2''), 56.5 (O*C*H$_3$); *m/z* HRMS (ES$^+$) MH$^+$ calculated for $C_{24}H_{19}O_6$ 403.1182, found 403.1198 MH$^+$.

2''-(4-Chlorophenyl)-(7-methoxy-4-oxo-2-phenylchromen-5-oxy) acetic acid **49**

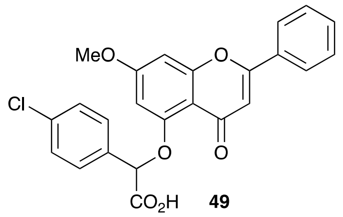

49

Obtained using ethyl 2-bromo-2-(4'-phenyl)acetate as a white solid (32 mg, 49 %). m.p 215-218 °C; υ_{max} (ATR) 3092 (O-H), 1732 (C=O), 1631 (C=O), 1613, 1573, 1491, 1446, 1360, 1332, 1201, 1163, 1112, 1090, 1015, 909, 848, 840, 820, 763, 687, 672, 646, 620 cm^{-1}; δ_H (700 MHz, d_6-DMSO): 13.44 (1 H, s, O*H*), 8.04 (2 H, d, *J* = 8.2, Hz, Ar*H*), 7.81 (2 H, d, *J* = 8.2 Hz, Ar*H*), 7.58-7.54 (3 H, m, Ar*H*), 7.50 (2 H, d, *J* = 8.5 Hz, Ar*H*), 6.95 (1 H, d, *J* = 2.2 Hz, 6-*H*), 6.80 (1 H, s, 3-*H*), 6.43 (1 H, d, *J* = 2.2 Hz, 8-*H*), 6.05 (1 H, s, 2''-*H*); δ_C (175 MHz, d_6-DMSO): 176.4 (*C*=O), 170.8 (*C*=O), 164.1 (*C*-7), 160.7 (*C*-2), 159.9 (*C*-5), 158.0 (*C*-9), 135.5 (Ar*C*), 133.8 (Ar*C*), 132.2 (Ar*C*), 131.5 (Ar*C*), 129.7 (2 × Ar*C*), 129.5 (2 × Ar*C*), 129.1

(2 × Ar*C*), 126.7 (2 × Ar*C*), 109.6 (*C*-10), 108.9 (*C*-3), 99.3 (*C*-6), 95.1 (*C*-8), 78.2 (*C*-2''), 56.8 (O*C*H$_3$); *m/z* HRMS (ES$^+$) MH$^+$ calculated for $C_{24}H_{18}O_6Cl$ 437.0792, found 437.0801 [^{35}Cl]MH$^+$.

2''-(7-Methoxy-4-oxo-2-phenylchromen-5-oxy)-3''-phenylpropanoic acid **50**

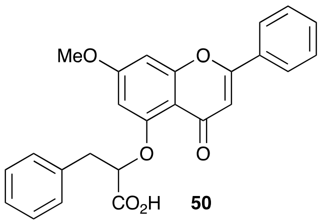

50

Obtained, using *ethyl 2-bromo-3-phenylpropionate,52* as a white solid (11 mg, 59 %). m.p 171-173 °C; υ_{max} (ATR) 3049 (O-H), 1743 (C=O), 1633 (C=O), 1604, 1588, 1574, 1449, 1356, 1202, 1162, 1106, 1056, 913, 849, 825, 762, 687, 643 cm^{-1}; δ_H (700 MHz, d$_6$-DMSO): 8.06 (2 H, dd, *J* = 7.9, 1.4 Hz, Ar*H*), 7.59-7.57 (3 H, m, Ar*H*), 7.53 (2 H, d, *J* = 7.4 Hz, Ar*H*), 7.25 (2 H, t, *J* = 7.4 Hz, Ar*H*), 7.19 (1 H, t, *J* = 7.4 Hz, Ar*H*), 6.88 (1 H, d, *J* = 2.3 Hz, 6-*H*), 6.85 (1 H, s, 3-*H*), 6.26 (1 H, d, *J* = 2.3 Hz, 8-*H*), 4.93 (1 H, br, s, 2''-*H*), 3.83 (3 H, s, OC*H*$_3$), 3.27-3.20 (2 H, m, 5''-*H*$_2$); δ_C (175 MHz, d$_6$-DMSO): 176.3, (*C*=O, *C*=O), 163.9 (*C*-2, 7), 160.3 (*C*-9), 159.5 (*C*-5), 131.9 (Ar*C*), 131.2 (Ar*C*), 130.4 (2 × Ar*C*), 129.5 (3 × Ar*C*), 128.4 (2 × Ar*C*), 126.9 (Ar*C*), 126.4 (2 × Ar*C*), 108.9 (*C*-10), 108.5 (*C*-3, 6), 98.6 (*C*-8), 81.6 (*C*-2''), 56.4 (O*C*H$_3$), 38.6 (*C*-3''); *m/z* HRMS (ES$^+$) MH$^+$ calculated for $C_{25}H_{21}O_6$ 417.1338, found 417.1321 MH$^+$.

2-(7'-Methoxy-4'-oxo-2'-phenyl-4'H-chromen-5'-oxy)-6-((1'''-oxo-pentylamino)hexanoic acid **52**

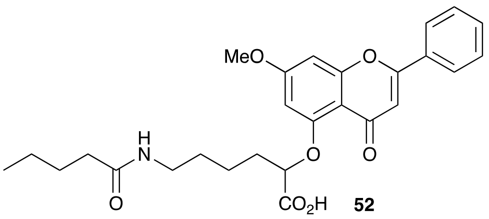

52

A solution of *methyl 6-((benzyloxycarbonyl)amino)-2-bromohexanoate* (637 mg, 1.8 mmol, 1.5 eq) in dry DMF (3 ml) was added to a mixture of 7-methoxychrysin (318 mg, 1.2 mmol, 1 eq) and K_2CO_3 (327 mg, 2.4 mmol, 2 eq) in dry DMF (7 ml) under an argon atmosphere. The reaction mixture was stirred at 60°C for 16 h. H_2O (10 ml) was added and the product was extracted with EtOAc (3 × 30ml). The combined organic layers were washed with H_2O (10 ml) and brine (10 ml), dried over Na_2SO_4 and the solvent was evaporated. Flash column chromatography (40 g silica gel, hexane:EtOAc 9:1 - 0:1) afforded *Methyl 6-((benzyloxycarbonyl)amino)-2-(7''-methoxy-4''-oxo-2''-phenyl-4''H-chromen-5''-oxy)hexanoate* (600 mg, 93%) as a colourless solid. m.p.: 109 - 111 °C; ν_{max} (ATR): 3406 (NH), 2951 (NH), 1721 (C=O), 1640 (C=O), 1598, 1163, 1120, 769 cm^{-1}; δ_H (CDCl$_3$, 700 MHz): 7.83 – 7.80 (m, 2H,

2′′′-H, 6′′′-H), 7.54 - 7.47 (m, 3H, 3′′′-H, 4′′′-H, 5′′′-H), 7.38 - 7.23 (m, 5H, 2′-H, 3′-H, 4′-H, 5′-H, 6′-H), 6.61 (d, *J* = 2.3 Hz, 1H, 8′′-H), 6.57 (s, 1H, 3′′-H), 6.22 (d, *J* = 2.3 Hz, 1H, 6′′-H), 5.60 - 5.56 (br m, 1H, NH), 5.11 - 5.05 (m, 2H, $ArCH_2$), 4.67 (dd, *J* = 7.6, 4.3 Hz, 1H, 2-H), 3.88 (s, 3H, OCH_3), 3.75 (s, 3H, $COOCH_3$), 3.33 – 3.23 (m, 2H, 6-H_2), 2.17 – 2.03 (m, 2H, 3-H_2), 1.80 - 1.57 (m, 4H, 4-H_2, 5-H_2); δ_C ($CDCl_3$, 175 MHz): 177.2 (C-4′′), 171.6 (C-1), 163.9 (C-7′′), 160.9 (C-2′′), 160.0 (C-8a′′), 159.0 (C-5′′), 156.8 (NHC=O), 137.1 (C-1′), 131.7 (C-1′′′), 131.3 (C-4′′′), 129.1 (C-3′′′, C-5′′′), 128.5 (C-3′, C-5′), 128.0 (C-2′, C-6′), 128.0 (C-4′), 126.1 (C-2′′′, C-6′′′), 110.1 (C-4a′′), 109.3 (C-3′′), 99.2 (C-6′′), 94.3 (C-8′′), 78.2 (C-2), 66.5 ($ArCH_2$), 55.9 (OCH_3), 52.5 (COO**C**H_3), 40.4 (C-6), 32.0 (C-3), 29.0 (C-5), 21.8 (C-4); HRMS (ES^+) found $[M+H]^+$ 546.2136, $C_{31}H_{32}NO_8$ requires *M* 546.2128.

HBr (0.5 ml, 33 wt. % solution in AcOH) was added to *methyl 6-((benzyloxycarbonyl)amino)-2-(7′′-methoxy-4′′-oxo-2′′-phenyl-4′′H-chromen-5′′-oxy) hexanoate* (259 mg, 0.5 mmol, 1 eq) under an argon atmosphere and the mixture was stirred for 2 h. After adding dry Et_2O (25 ml) and stirring for another 10 min, the solid was collected by filtration, washed with Et_2O and dried, which yielded *6-methoxy-5-(7′-methoxy-4′-oxo-2′-phenyl-4′H-chromen-5′-oxy)-6-oxo-hexylammonium bromide* **51** (230 mg, 98%) as a yellow solid. m.p.: 144 - 145 °C (decomposition); ν_{max} (ATR): 2851 (NH), 1739 (C=O), 1631 (C=O), 1593, 1366, 1208 cm^{-1}; δ_H (d^6-DMSO, 700 MHz): 8.08 - 8.02 (m, 2H, 2′′-H, 6′′-H), 7.68 (br s, 3H, NH_3^+), 7.62 - 7.54 (m, 3H, 3′′-H, 4′′-H, 5′′-H), 6.95 (d, *J* = 2.3 Hz, 1H, 8′-H), 6.75 (s, 1H, 3′-H), 6.30 (d, *J* = 2.3 Hz, 1H, 6′-H), 4.98 (t, *J* = 5.9 Hz, 1H, 5-H), 3.88 (s, 3H, OCH_3), 3.70 (s, 3H, $COOCH_3$), 2.85 – 2.80 (m, 2H, 1-H_2), 1.98 – 1.93 (m, 2H, 4-H_2), 1.68 - 1.51 (m, 4H, 2-H_2, 3-H_2); δ_C (d^6-DMSO, 175 MHz): 175.5 (C-4′), 170.9 (C-6), 163.4 (C-7′), 159.8 (C-2′), 159.2 (C-8a′), 158.1 (C-5′), 131.5 (C-4′′), 130.8 (C-1′′), 129.1 (C-3′′, C-5′′), 126.0 (C-2′′, C-6′′), 108.8 (C-4a′), 108.2 (C-3′), 98.4 (C-6′), 94.3 (C-8′), 76.4 (C-5), 56.1 (OCH_3), 52.2 (COO**C**H_3), 38.7 (C-1), 31.4 (C-4), 26.5 (C-2), 21.3 (C-3); HRMS (ES^+) found $[M+H]^+$ 412.1765, $C_{23}H_{26}NO_6$ requires *M* 412.1760.

Valeric acid (0.11 ml, 1.02 mmol, 2 eq) was added to a solution of *N*-(3-dimethylaminopropyl)-N′-ethylcarbodiimide hydrochloride (214 mg, 1.12 mmol, 2.2 eq) in dry DCM (7.5 ml) at 0 °C and the mixture was stirred for 30 min. After the addition of DMAP (25 mg, 0.20 mmol, 0.4 eq), *N,N*-diisopropylethylamine (164 mg, 1.27 mmol, 2.5 eq) and *6-methoxy-5-(7′-methoxy-4′-oxo-2′-phenyl-4′H-chromen-5′-oxy)-6-oxo-hexylammonium bromide* **51** (250 mg, 0.51 mmol, 1 eq) the reaction mixture was stirred for 2.5 h at rt. Sat. aqueous NH_4Cl solution (20ml) was added and the mixture was diluted with EtOAC (60 ml). The organic layer was separated and washed with sat. aqueous NH_4Cl solution (2 × 30ml), sat. aqueous $NaHCO_3$ solution (30ml) and brine (30 ml) and dried over Na_2SO_4. The solvent was removed under reduced pressure and flash column chromatography (24 g silica gel, EtOAc) yielded *methyl 2-(7′-methoxy-4′-oxo-2′-phenyl-4′H-chromen-5′-oxy)-6-((1′′′-oxo-*

pentylamino)hexanoate (175 mg, 70%) as a colourless solid. m.p.: 108 - 110 °C; ν_{max} (ATR): 3280 (NH), 2954, 2930, 2862, 1756 (C=O), 1646 (C=O), 1609, 1163, 1124 cm^{-1}; δ_H ($CDCl_3$, 400 MHz): 7.92 - 7.82 (m, 2H, 2''-H, 6''-H), 7.58 - 7.45 (m, 3H, 3''-H, 4''-H, 5''-H), 6.90 – 6.83 (br m, 1H, NH), 6.61 (d, *J* = 2.3 Hz, 1H, 8'-H), 6.59 (s, 1H, 3'-H), 6.18 (d, *J* = 2.3 Hz, 1H, 6'-H), 4.67 (dd, *J* = 7.6, 4.0 Hz, 1H, 2-H), 3.87 (s, 3H, OCH_3), 3.76 (s, 3H, $COOCH_3$), 3.44 - 3.23 (m, 2H, 6-H_2), 2.27 - 2.19 (m, 2H, 2'''-H_2), 2.19 - 1.98 (m, 2H, 3-H_2), 1.87 - 1.53 (m, 6H, 4-H_2, 5-H_2, 3'''-H_2), 1.37 – 1.27 (m, 2H, 4'''-H_2), 0.87 (t, *J* = 7.3 Hz, 3H, 5'''-H_3); δ_C ($CDCl_3$, 100 MHz): 177.4 (C-4'), 174.2 (C-1'''), 171.5 (C-1), 164.2 (C-7'), 161.5 (C-2'), 160.2 (C-8a'), 159.1 (C-5'), 131.6 (C-4''), 131.5 (C-1''), 129.2 (C-3'', C-5''), 126.2 (C-2'', C-6''), 109.6 (C-4a'), 108.8 (C-3'), 98.7 (C-6'), 94.1 (C-8'), 77.8 (C-2), 56.0 (OCH_3), 52.5 ($COOCH_3$), 38.4 (C-6), 36.4 (C-2'''), 31.6 (C-3), 28.3 (C-5/C-3'''), 28.1 (C-5/C-3'''), 22.7 (C-4'''), 21.6 (C-4), 14.0 (C-5'''); HRMS (ES$^+$) found [M+H]$^+$ 496.2344, $C_{28}H_{34}NO_7$ requires *M* 496.2335. *Methyl 2-(7'-methoxy-4'-oxo-2'-phenyl-4'H-chromen-5'-oxy)-6-((1'''-oxo-pentylamino)-hexanoate* (110 mg, 0.22 mmol, 1 eq) was dissolved in a 1:1 mixture of THF (5 ml) and H_2O (5 ml) and LiOH.H_2O (19 mg, 0.44 mmol, 2 eq) was added. The reaction mixture was stirred for 1.5 h at rt and then neutralized with 1M HCl. The solvent was removed under reduced pressure. Flash column chromatography (24 g silica gel, DCM:MeOH 19:1 - 7:3) afforded the title product **52** (100 mg, 94%) as a colourless solid. m.p.: 186 - 188 °C (decomposition); ν_{max} (ATR): 3265 (NH), 2934, 2870, 1635 (C=O), 1628 (C=O), 1591, 1162, 1109 cm^{-1}; δ_H (d^6-DMSO, 700 MHz): 8.09 - 7.99 (m, 2H, 2''-H, 6''-H), 7.74 (t, *J* = 5.6 Hz, 1H, NH), 7.63 - 7.53 (m, 3H, 3''-H, 4''-H, 5''-H), 6.83 (br s, 1H, 8'-H), 6.78 (s, 1H, 3'-H), 6.43 (br s, 1H, 6'-H), 4.52 (br t, *J* = 5.7 Hz, 1H, 2-H), 3.85 (s, 3H, OCH_3), 3.01 – 2.91 (m, 2H, 6-H_2), 1.99 (t, *J* = 7.5 Hz, 2H, 2'''-H_2), 1.88 – 1.78 (m, 2H, 3-H_2), 1.46 – 1.31 (m, 6H, 4-H_2, 5-H_2, 3'''-H_2), 1.22 – 1.16 (m, 2H, 4'''-H_2), 0.80 (t, *J* = 7.4 Hz, 3H, 5'''-H_2); δ_C (d^6-DMSO 175 MHz): 176.8 (C-4'), 172.3 (C-1), 171.8 (C-1'''), 163.9 (C-7'), 160.2 (C-2'), 159.2 (C-8a'), 158.7 (C-5'), 131.6 (C-4''), 130.7 (C-1''), 129.1 (C-3'', C-5''), 126.0 (C-2'', C-6''), 108.4 (C-4a'), 107.9 (C-3'), 97.9 (C-6'), 93.0 (C-8'), 79.5 (C-2), 55.9 (OCH_3), 38.4 (C-6), 35.1 (C-2'''), 30.9 (C-3), 29.1 (C-5), 27.4 (C-3'''), 22.0 (C-4), 21.8 (C-4'''), 13.7 (C-5''')*;* HRMS (ES$^+$) found [M+H]$^+$ 482.2184, $C_{27}H_{32}NO_7$ requires *M* 482.2179.

Synthesis of quercetin analogue 53

3,7-Bisbenzyloxy-2-(3',4'-bisenzyloxyphenyl)-5-hydroxychromen-4-one **53**

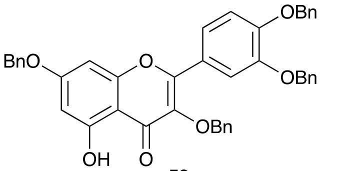

53

Benzyl bromide (2.38 ml, 20 mmol, 10 eq) was added to quercetin **3** (677 mg, 2 mmol, 1 eq) and K_2CO_3 (2.764 g, 20 mmol, 10 eq) in dry DMF (12 ml) under an argon atmosphere. The reaction mixture was stirred at 80 °C for 7 h. H_2O (20 ml) was added and the product was extracted with DCM (3 × 20 ml). The combined organic layers were washed with H_2O (2 × 20 ml), dried over $MgSO_4$ and concentrated. Flash column chromatography (80 g silica gel, hexane:EtOAc 4:1 – 7:3) afforded the title product (616 mg, 46 %) as a yellow solid. m.p.: 139 - 141 °C (lit. m.p. 140 - 142 °C); ν_{max} (ATR): 2922, 2851, 1654, 1592, 1494, 1201, 1165, 1015 cm^{-1}; δ_H ($CDCl_3$, 400 MHz): 12.74 (s, 1H), 7.78 – 7.71 (m, 1H), 7.61 – 7.56 (m, 1H), 7.53 – 7.20 (m, 21H), 6.99 (d, *J* = 8.7 Hz, 1H), 6.49 (d, *J* = 2.2 Hz, 1H), 6.47 (d, *J* = 2.2 Hz, 1H), 5.28 (s, 2H), 5.16 (s, 2H), 5.07 (s, 2H), 5.02 (s, 2H); δ_C ($CDCl_3$, 100 MHz): 178.9, 164.6, 162.2, 156.8, 156.5, 151.2, 148.3, 137.6, 137.0, 136.8, 136.6, 135.9, 128.9, 128.9, 128.8, 128.6, 128.5, 128.4, 128.4, 128.2, 128.0, 127.6, 127.5, 127.3, 123.6, 122.7, 115.4, 113.8, 106.3, 98.7, 93.2, 74.5, 71.2, 71.0, 70.6; HRMS (AP$^+$) found $[M+H]^+$ 663.2411, $C_{43}H_{35}O_7$ requires *M* 663.2383.

Methyl 2-{[3',7'-bisbenzyloxy-2-(3'',4''-bisbenzyloxyphenyl)-4'-oxo-4'H-chromen-5'-yl]oxy} dodecanoate **54**

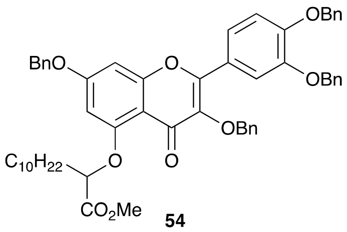

54

Methyl α-bromododecanoate (440 mg, 1.5 mmol, 2 eq) was added to a mixture of *3,7-bisbenzyloxy-2-(3',4'-bisbenzyloxyphenyl)-5-hydroxychromen-4-one* **53** (497 mg, 0.75 mmol, 1 eq) and K_2CO_3 (207 mg, 1.5 mmol, 2 eq) in dry DMF (2 ml) under an argon atmosphere. The reaction mixture was stirred at 60 °C for 18 h. H_2O (5 ml) was added and the product was extracted with DCM (3 × 5 ml). The combined organic layers were dried over Na_2SO_4 and concentrated. Flash column chromatography (24 g silica gel, DCM:MeOH 1:0 – 19:1), followed by titration with MeOH afforded the title ether (446 mg, 68 %) as a colourless semisolid. ν_{max} (ATR): 2924, 2854, 1751, 1625, 1604, 1430, 1191 cm^{-1}; δ_H ($CDCl_3$, 700 MHz): 7.74 – 7.69 (m, 1H), 7.54 – 7.50 (m, 1H), 7.49 – 7.27 (m, 17H), 7.25 – 7.20 (m, 3H), 6.95 (d, *J* = 8.6 Hz, 1H), 6.57 (d, *J* = 1.8 Hz, 1H), 6.31 (d, *J* = 2.3 Hz, 1H), 5.23 (s, 2H), 5.17 (d, *J* = 10.9 Hz, 1H), 5.10 (s, 2H), 5.00 (d, *J* = 10.8 Hz, 1H), 4.94 (s, 2H), 4.69 (t, *J* = 6.4 Hz, 1H), 3.75 (s, 3H), 2.23 – 2.15 (m, 1H), 2.11 – 2.04 (m, 1H), 1.66 – 1.59 (m, 2H), 1.40 – 1.21 (m, 14H), 0.88 (t, *J* = 7.1 Hz, 3H); δ_C ($CDCl_3$, 175 MHz): 173.6, 171.9, 162.6, 159.4, 158.8, 153.2, 150.6, 148.4, 139.9, 137.2, 137.2, 137.0, 135.8, 129.0, 128.9, 128.7, 128.6, 128.3, 128.1, 128.1, 127.9, 127.7, 127.5, 127.3, 124.1, 122.2, 115.4, 113.9, 110.4, 99.1, 95.0, 78.7, 74.3, 71.2, 71.0, 70.6, 52.3, 32.8, 32.0, 29.7, 29.5, 29.5, 29.3, 25.1, 22.8, 14.3; HRMS (AP$^+$) found $[M+H]^+$ 875.4194, $C_{56}H_{59}O_9$ requires *M* 875.4159.

2-{[3',7'-(Dihydroxy)-2'-(3'',4''-dihydroxyphenyl)-4'-oxo-4'H-chromen-5'-yl]oxy}dodecanoic acid **55**

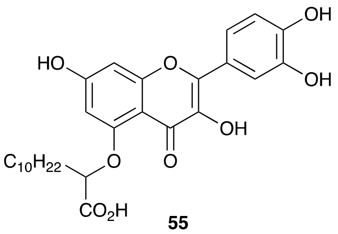

55

Methyl 2-{[3',7'-bisbenzyloxy-2-(3'',4''-bisbenzyloxyphenyl)-4'-oxo-4'H-chromen-5'-yl]oxy} dodecanoate (333 mg, 0.38 mmol, 1 eq) was dissolved in a 4:1 mixture of THF (7.6 ml) and H_2O (1.9 ml) and KOH (128 mg, 2.28 mmol, 6 eq) added. The reaction mixture was stirred for 24 h at rt. The mixture was acidified with 1M HCl to pH 1 and extracted with DCM (3 × 5 ml). The combined organic layers were dried over Na_2SO_4 and concentrated. EtOH (5.7 ml) and EtOAc (5.7 ml) were added to the crude product and Pd/C (5% Pd basis, 95 mg) under an argon atmosphere, which was then exchanged for a hydrogen atmosphere. The reaction mixture was stirred at rt for 18 h, filtered through celite and concentrated. Flash column chromatography (12 g silica gel, DCM:MeOH 1:0 – 9:1) afforded the title product (62 mg, 33 %) as a yellow semisolid. ν_{max} (ATR): 3310, 2918, 2850, 2461, 1712, 1591, 1500, 1327, 1169, 1104 cm^{-1}; δ_H (MeOD, 700 MHz): 7.74 (d, *J* = 2.1 Hz, 1H), 7.63 (dd, *J* = 8.5, 2.2 Hz, 1H), 6.89 (d, *J* = 8.5 Hz, 1H), 6.57 (d, *J* = 2.0 Hz, 1H), 6.34 (d, *J* = 2.1 Hz, 1H), 4.81 (dd, *J* = 6.8, 4.8 Hz, 1H), 2.17 – 2.04 (m, 2H), 1.67 – 1.51 (m, 2H), 1.44 – 1.20 (m, 14H), 0.87 (t, *J* = 7.1 Hz, 3H); δ_C (MeOD, 175 MHz): 174.9, 173.7, 164.4, 160.2, 159.8, 148.6, 146.3, 145.5, 138.6, 124.1, 121.4, 116.3, 115.8, 107.0, 99.9, 97.2, 80.4, 33.5, 33.1, 30.7, 30.6, 30.5, 30.5, 30.3, 25.8, 23.7, 14.4; HRMS (ES$^+$) found [M+H]$^+$ 501.2142, $C_{27}H_{33}O_9$ requires *M* 501.2125.

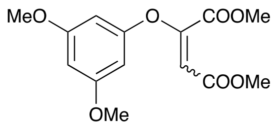
MeO
O
COOMe
COOMe
OMe

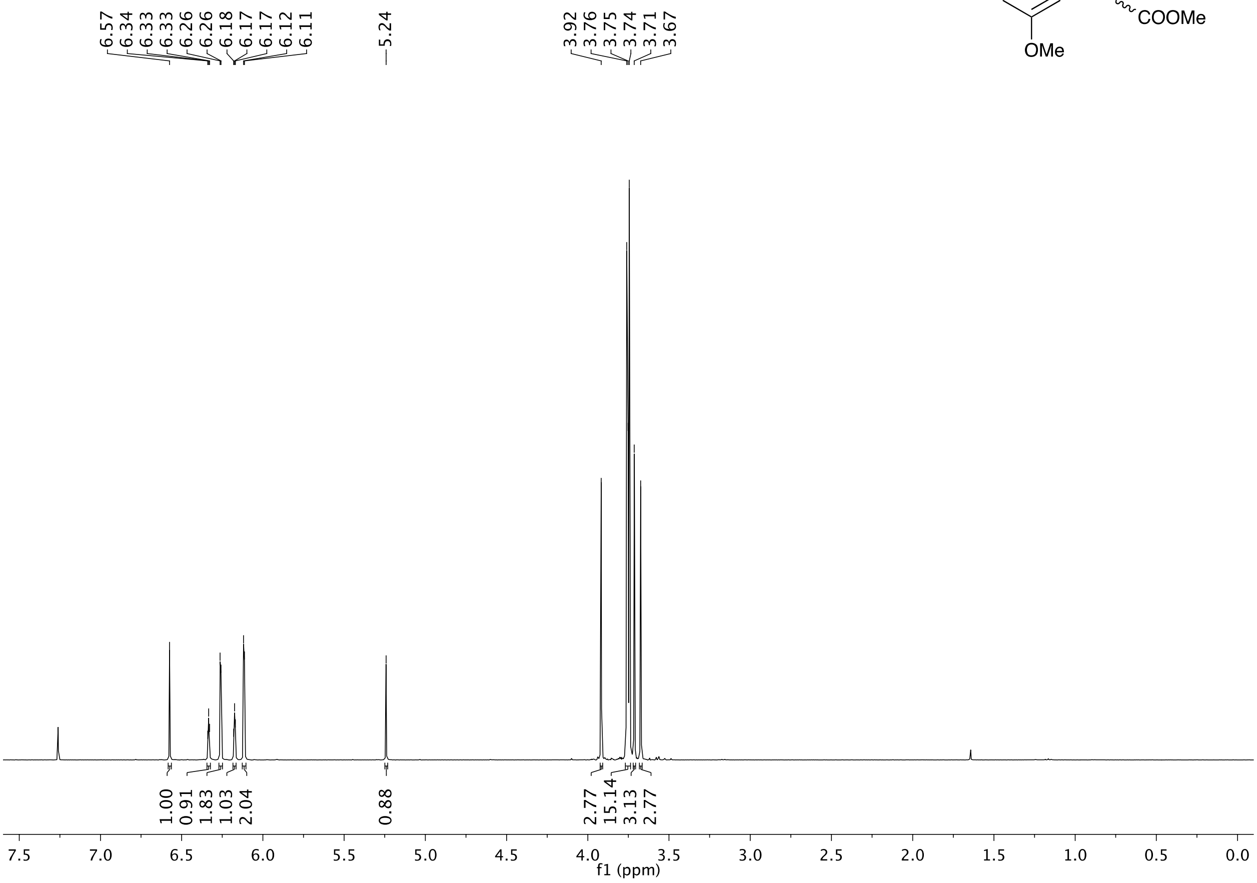
6.57
6.34
6.33
6.33
6.26
6.26
6.18
6.17
6.12
6.11
5.24
3.92
3.76
3.75
3.74
3.71
3.67
1.00
0.91
1.83
1.03
2.04
0.88
2.77
15.14
3.13
2.77
7.5 7.0 6.5 6.0 5.5 5.0 4.5 4.0 3.5 3.0 2.5 2.0 1.5 1.0 0.5 0.0
f1 (ppm)

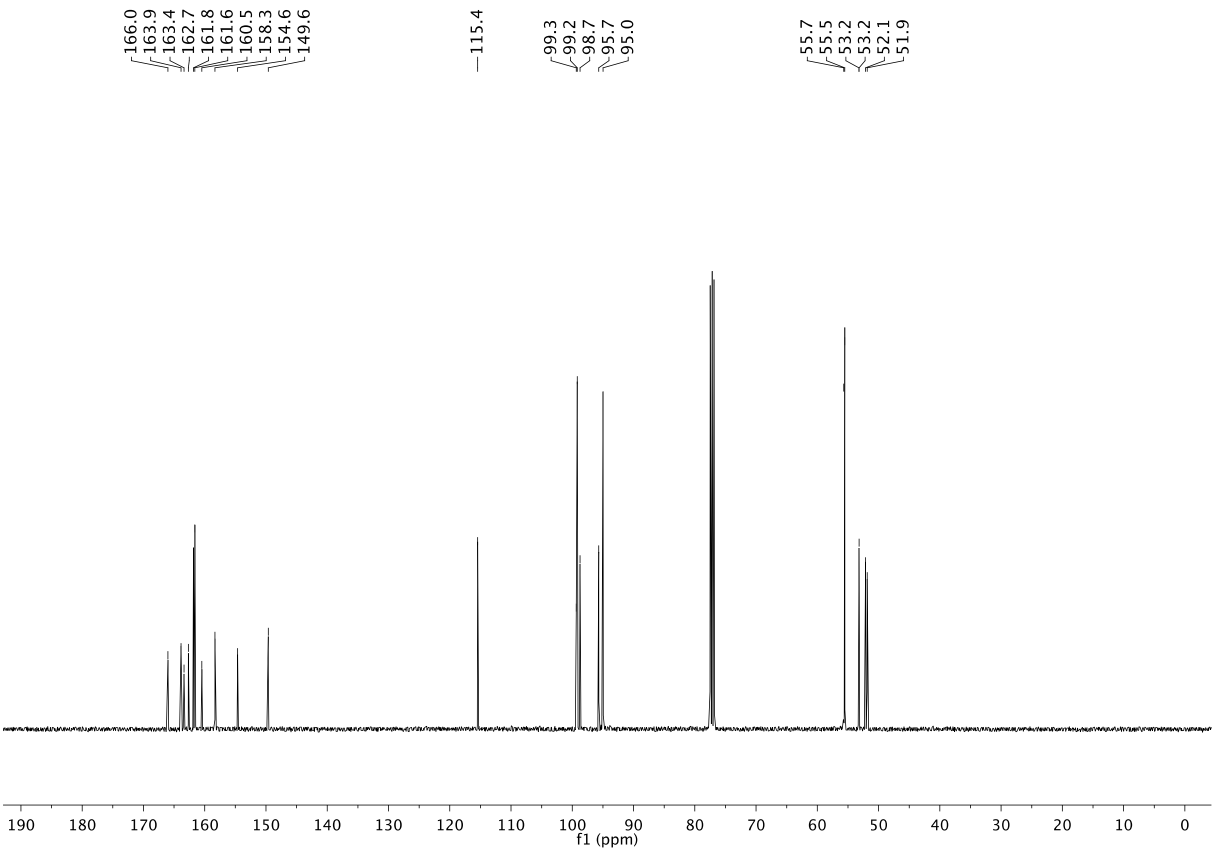
166.0
163.9
163.4
162.7
161.8
161.6
160.5
158.3
154.6
149.6
115.4
99.3
99.2
98.7
95.7
95.0
55.7
55.5
53.2
53.2
52.1
51.9
190 180 170 160 150 140 130 120 110 100 90 80 70 60 50 40 30 20 10 0
f1 (ppm)

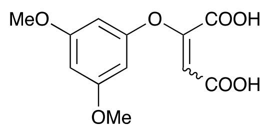

MeO
O
COOH
COOH
OMe

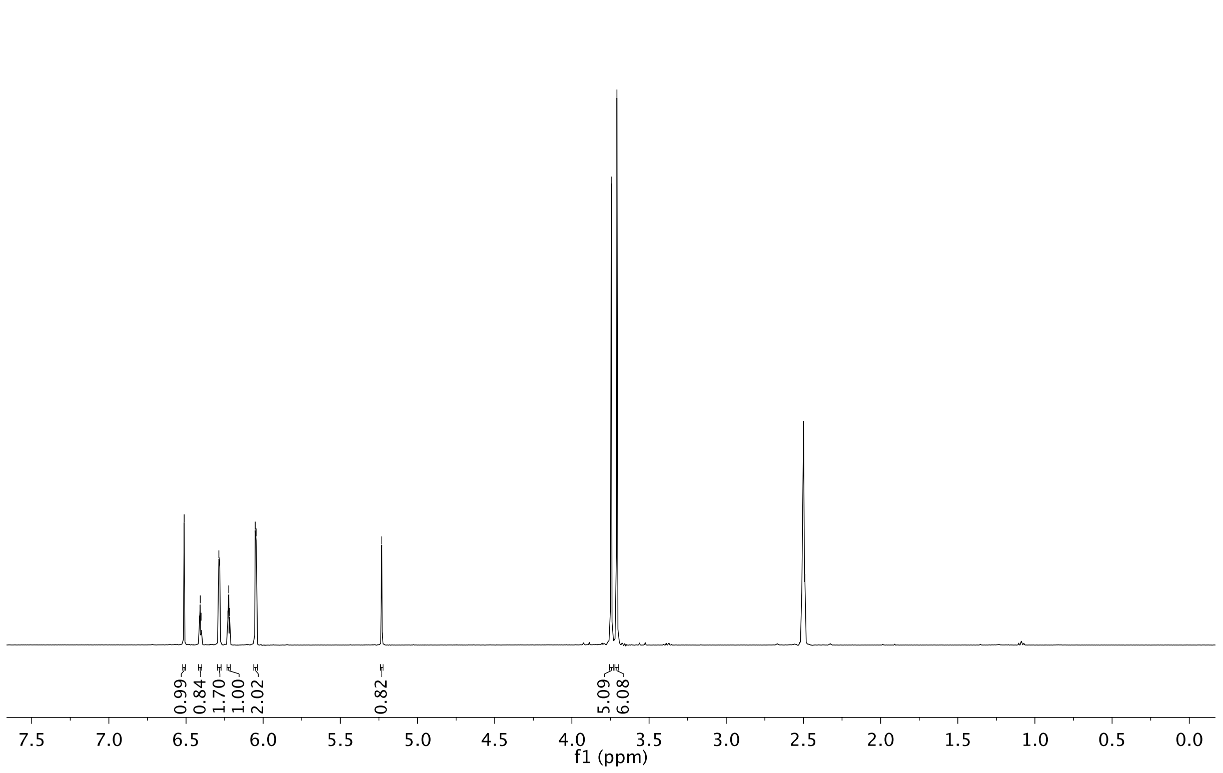

6.51
6.41
6.41
6.40
6.29
6.28
6.23
6.22
6.22
6.05
6.05
5.23
3.75
3.71
0.99
0.84
1.70
1.00
2.02
0.82
5.09
6.08
7.5 7.0 6.5 6.0 5.5 5.0 4.5 4.0 3.5 3.0 2.5 2.0 1.5 1.0 0.5 0.0
f1 (ppm)

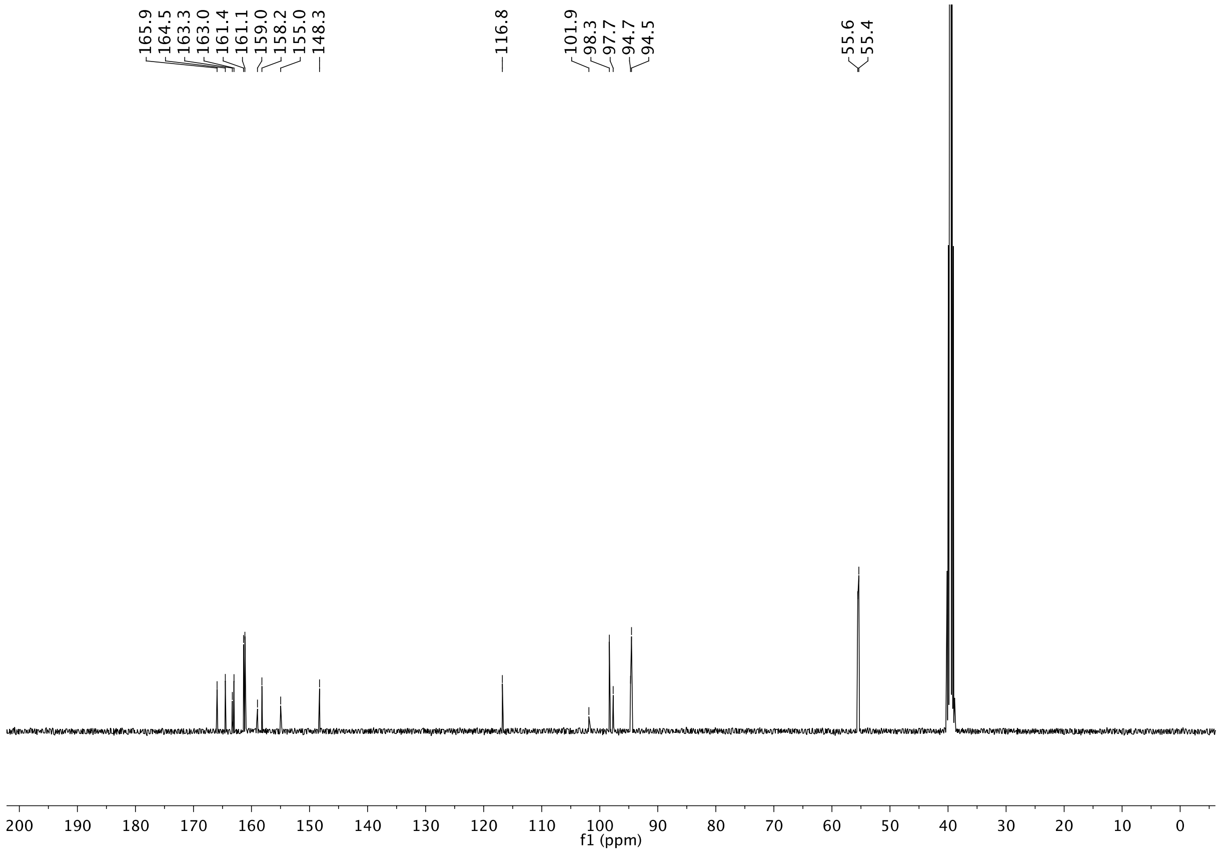

165.9
164.5
163.3
163.0
161.4
161.1
159.0
158.2
155.0
148.3
116.8
101.9
98.3
97.7
94.7
94.5
55.6
55.4
200 190 180 170 160 150 140 130 120 110 100 90 80 70 60 50 40 30 20 10 0
f1 (ppm)

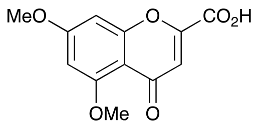
MeO
O
CO_2H
OMe
O

13

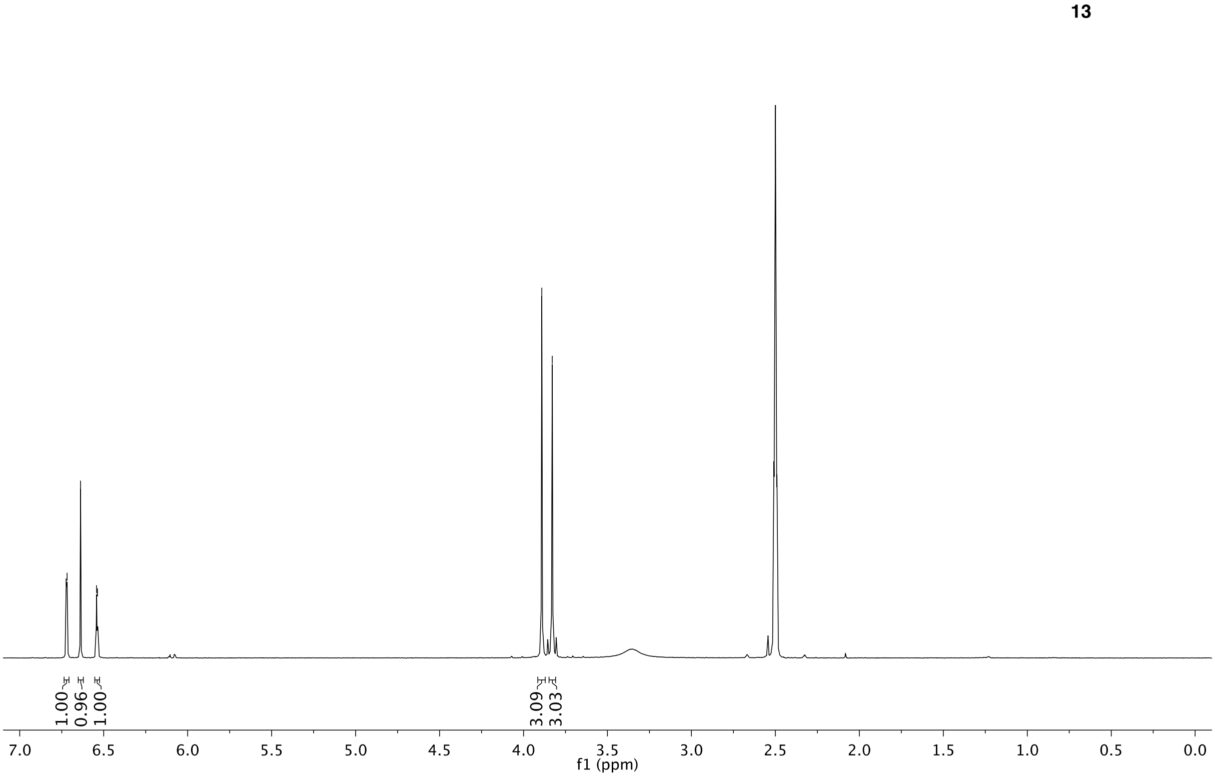
6.72
6.72
6.64
6.54
6.54
3.89
3.83
1.00
0.96
1.00
3.09
3.03
7.0 6.5 6.0 5.5 5.0 4.5 4.0 3.5 3.0 2.5 2.0 1.5 1.0 0.5 0.0
f1 (ppm)

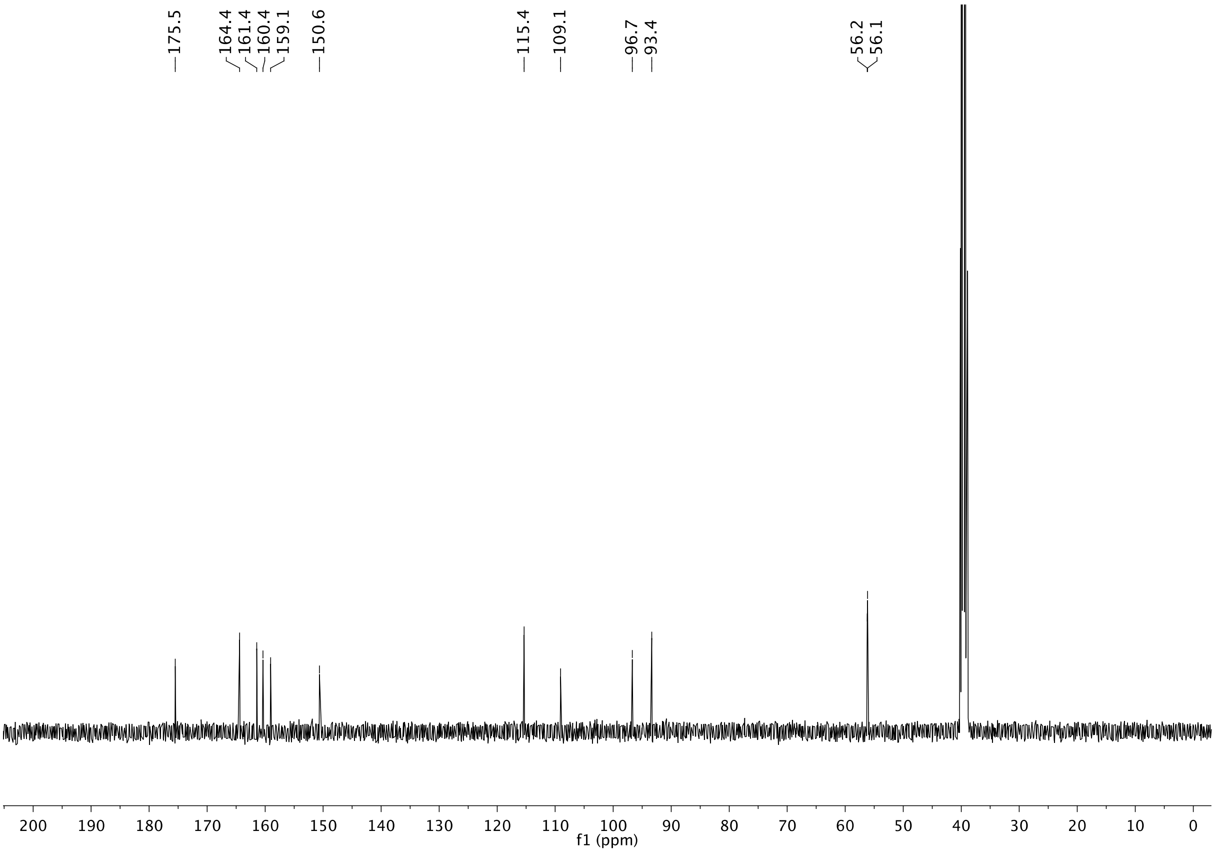
175.5
164.4
161.4
160.4
159.1
150.6
115.4
109.1
96.7
93.4
56.2
56.1
200 190 180 170 160 150 140 130 120 110 100 90 80 70 60 50 40 30 20 10 0
f1 (ppm)

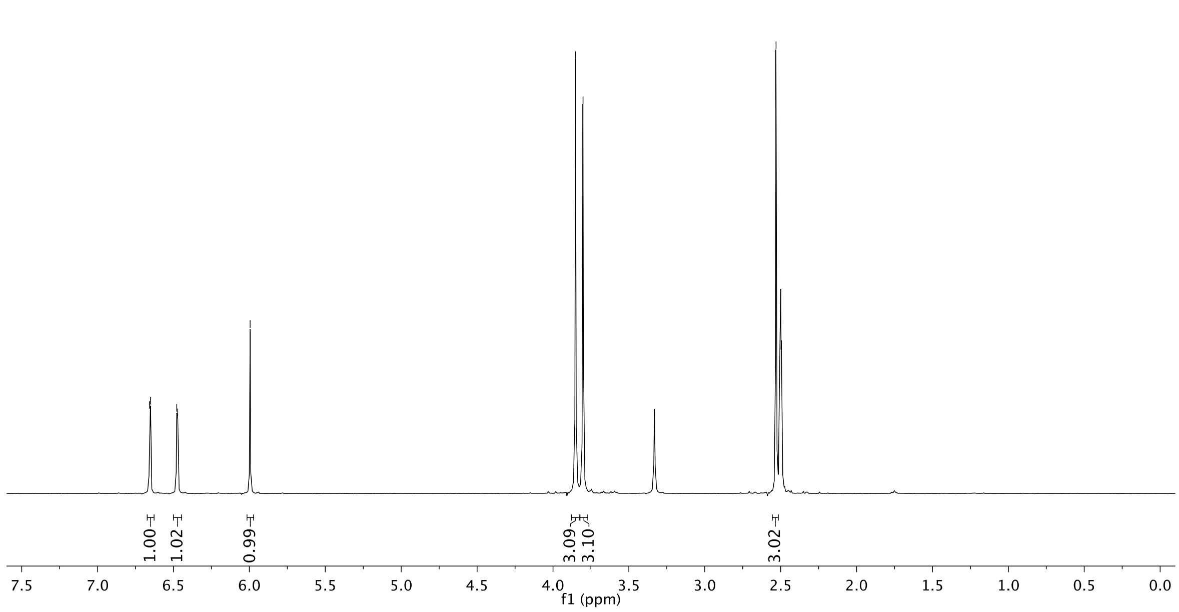
6.66
6.65
6.48
6.47
6.00
3.85
3.80
2.53
1.00
1.02
0.99
3.09
3.10
3.02
7.5 7.0 6.5 6.0 5.5 5.0 4.5 4.0 3.5 3.0 2.5 2.0 1.5 1.0 0.5 0.0
f1 (ppm)

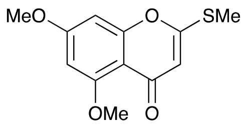
MeO
O
SMe
OMe
O

14

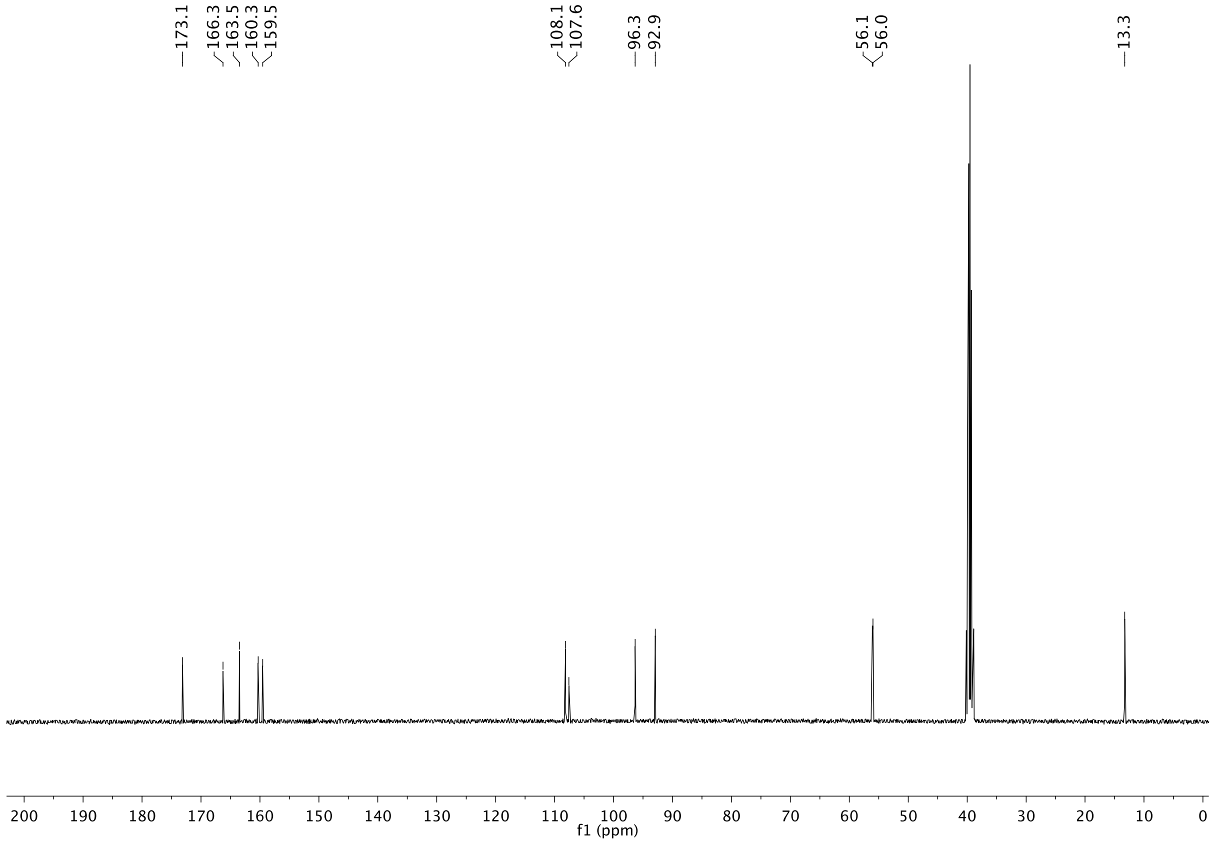
173.1
166.3
163.5
160.3
159.5
108.1
107.6
96.3
92.9
56.1
56.0
13.3
200 190 180 170 160 150 140 130 120 110 100 90 80 70 60 50 40 30 20 10 0
f1 (ppm)

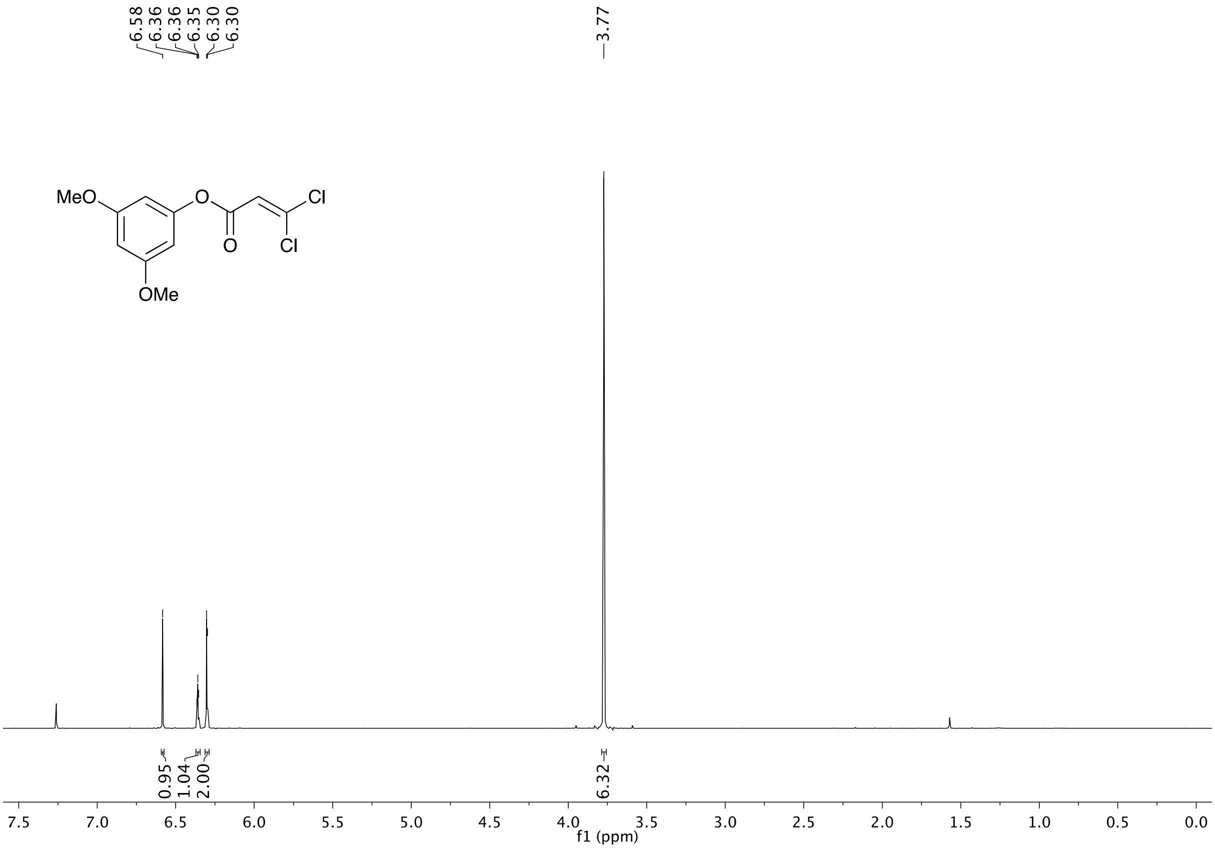
6.58
6.36
6.36
6.35
6.30
6.30
3.77
MeO
O
Cl
O
Cl
OMe
0.95
1.04
2.00
6.32
7.5 7.0 6.5 6.0 5.5 5.0 4.5 4.0 3.5 3.0 2.5 2.0 1.5 1.0 0.5 0.0
f1 (ppm)

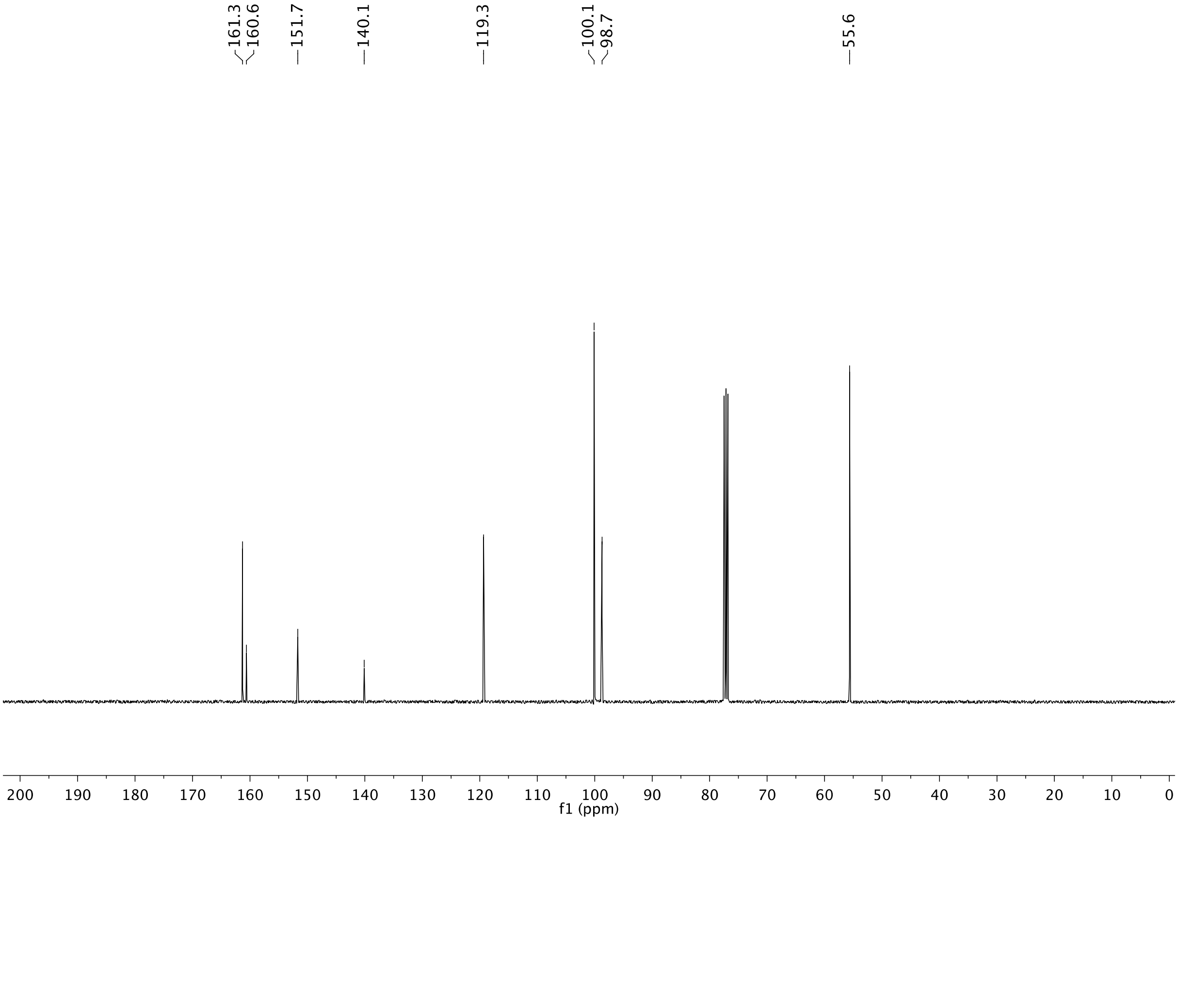
161.3
160.6
151.7
140.1
119.3
100.1
98.7
55.6
200 190 180 170 160 150 140 130 120 110 100 90 80 70 60 50 40 30 20 10 0
f1 (ppm)

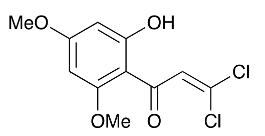
MeO
OH
Cl
OMe
O
Cl

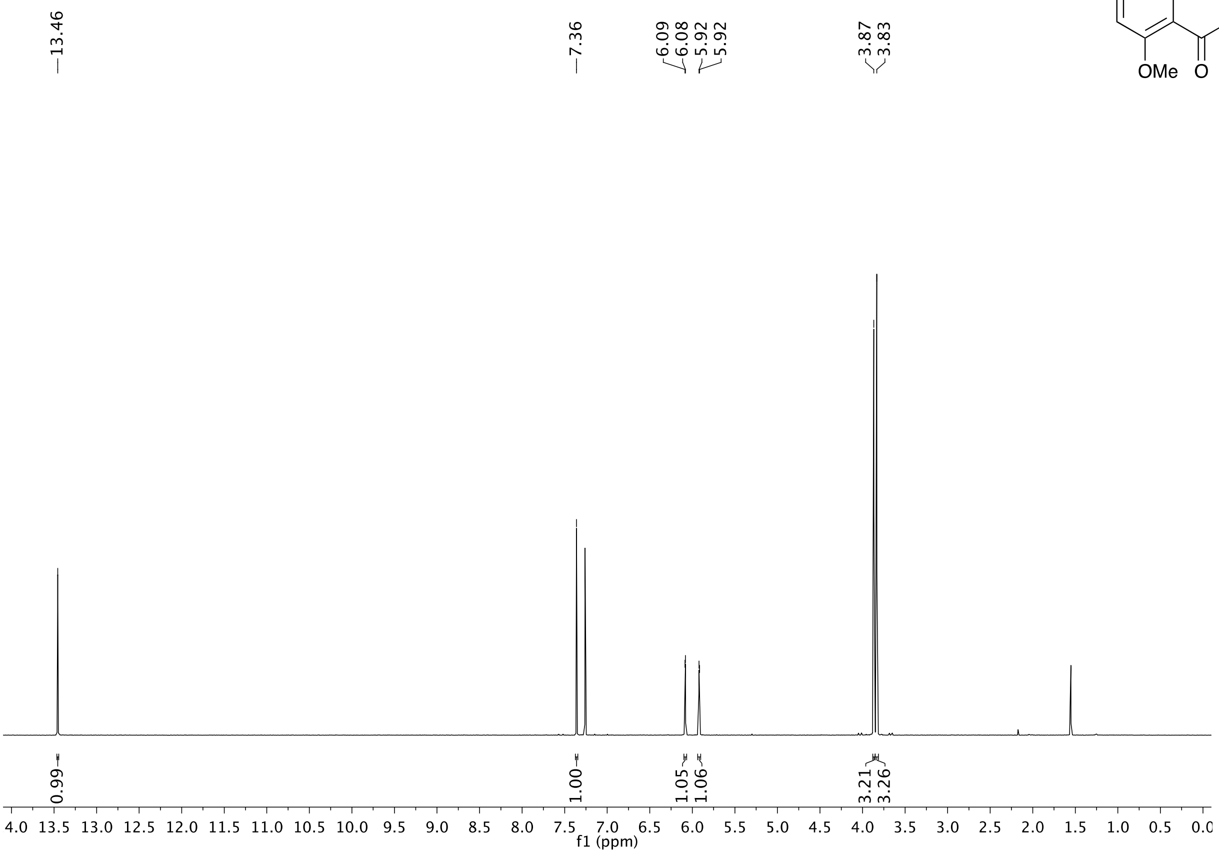
13.46
7.36
6.09
6.08
5.92
5.92
3.87
3.83
0.99
1.00
1.05
1.06
3.21
3.26
4.0 13.5 13.0 12.5 12.0 11.5 11.0 10.5 10.0 9.5 9.0 8.5 8.0 7.5 7.0 6.5 6.0 5.5 5.0 4.5 4.0 3.5 3.0 2.5 2.0 1.5 1.0 0.5 0.0
f1 (ppm)

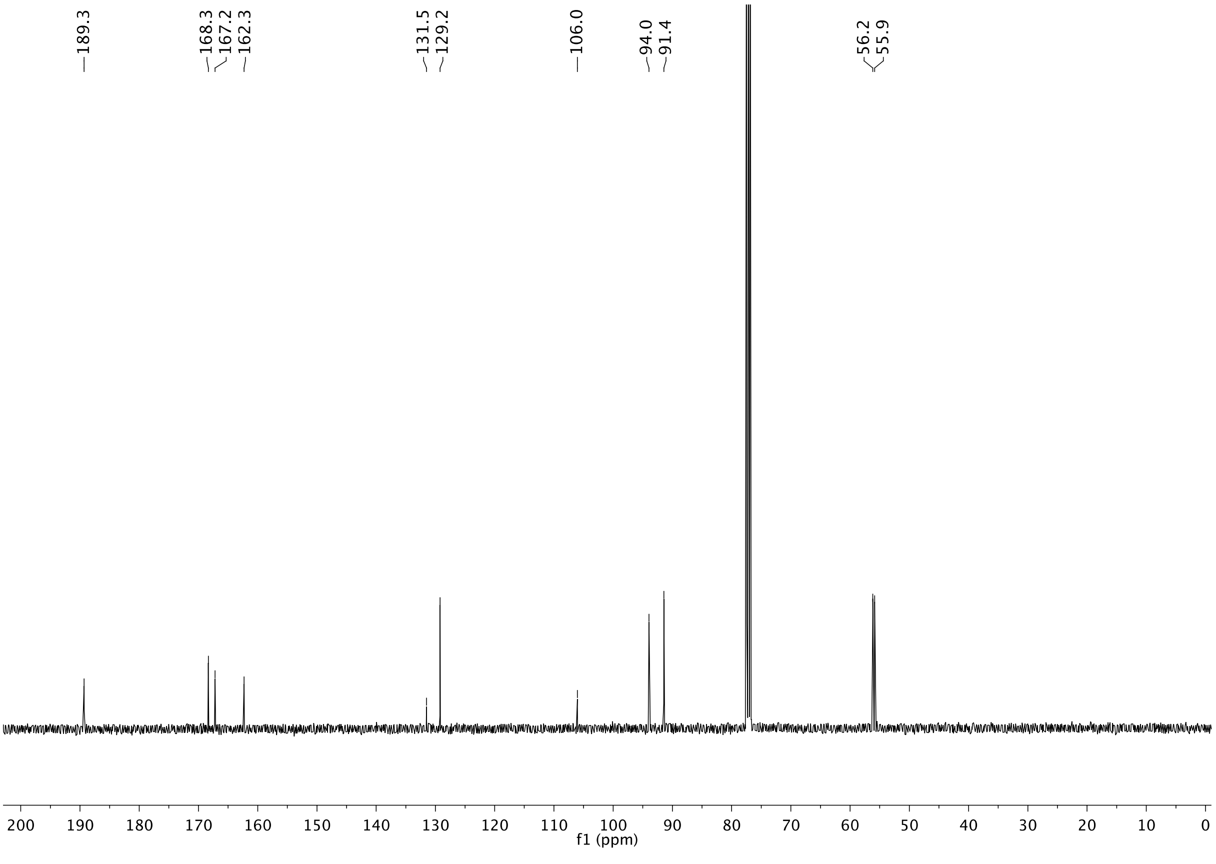
189.3
168.3
167.2
162.3
131.5
129.2
106.0
94.0
91.4
56.2
55.9
200 190 180 170 160 150 140 130 120 110 100 90 80 70 60 50 40 30 20 10 0
f1 (ppm)

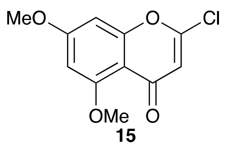
MeO
O
Cl
OMe
O

15

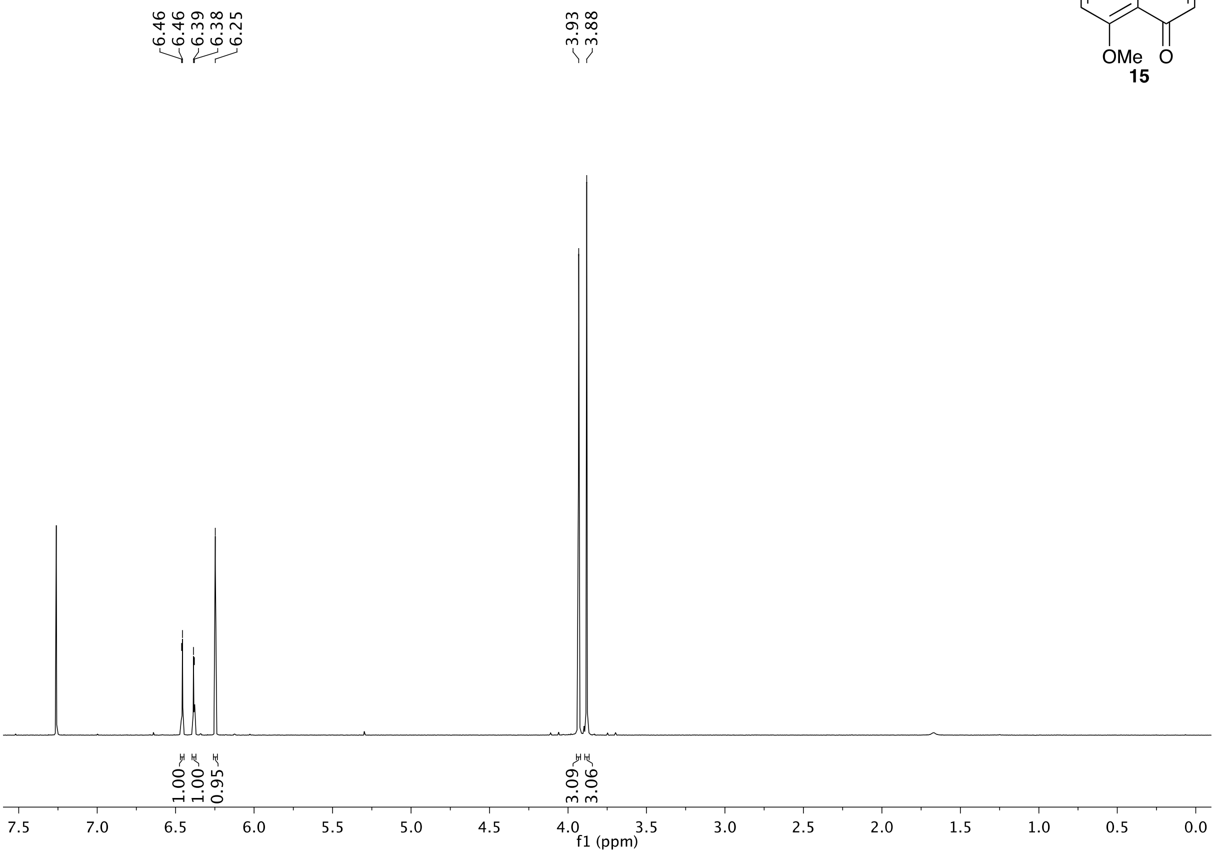
6.46
6.46
6.39
6.38
6.25
3.93
3.88
1.00
1.00
0.95
3.09
3.06
7.5 7.0 6.5 6.0 5.5 5.0 4.5 4.0 3.5 3.0 2.5 2.0 1.5 1.0 0.5 0.0
f1 (ppm)

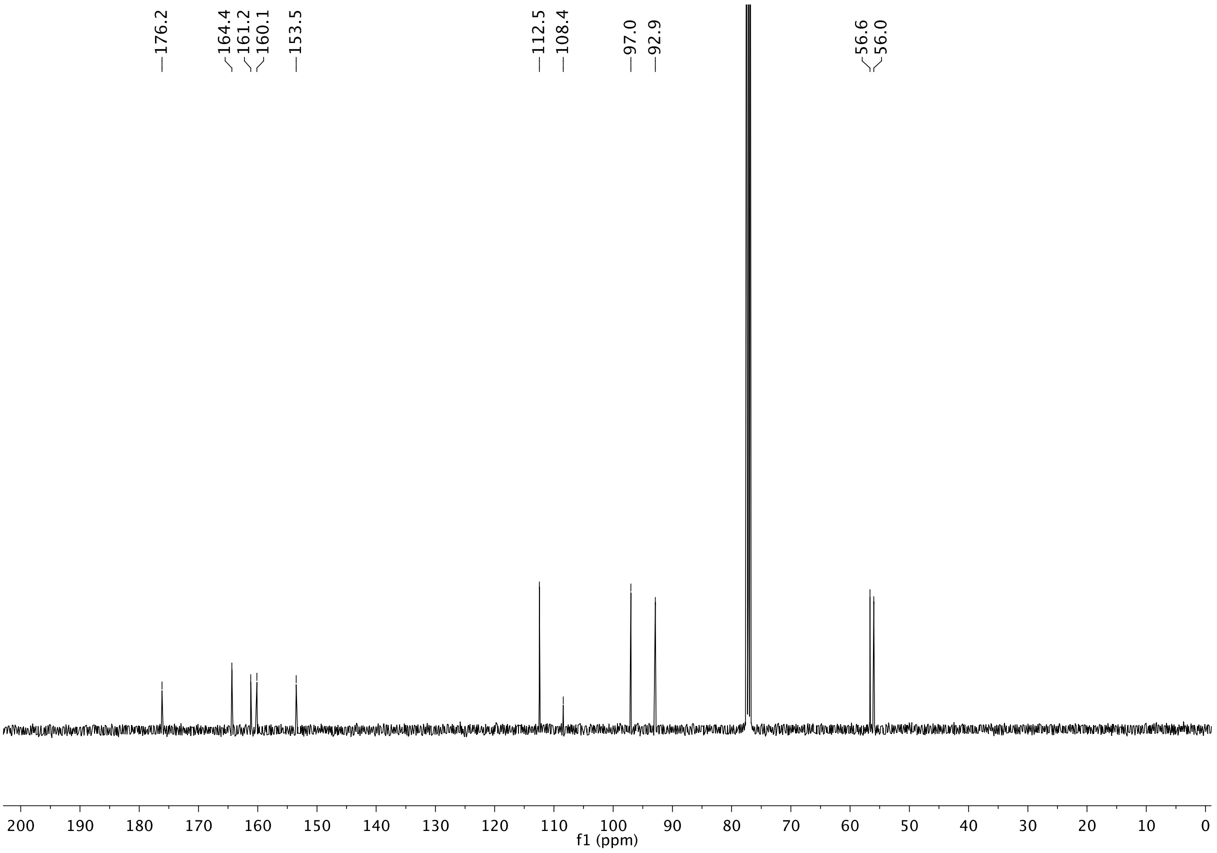
176.2
164.4
161.2
160.1
153.5
112.5
108.4
97.0
92.9
56.6
56.0
200 190 180 170 160 150 140 130 120 110 100 90 80 70 60 50 40 30 20 10 0
f1 (ppm)

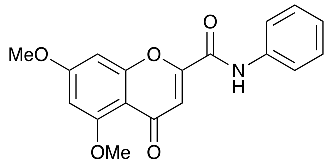

16a

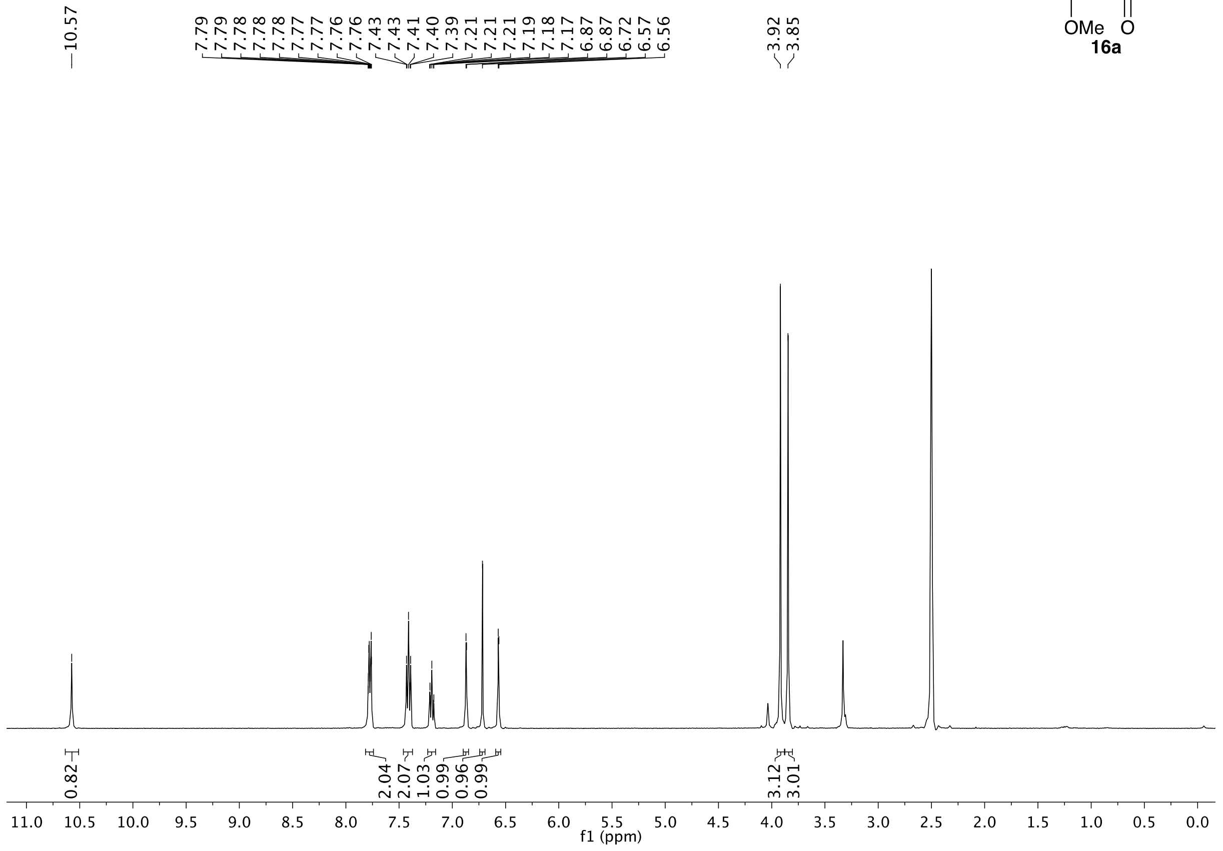

10.57
7.79
7.79
7.78
7.78
7.78
7.77
7.77
7.76
7.76
7.43
7.43
7.41
7.40
7.39
7.21
7.21
7.21
7.19
7.18
7.17
6.87
6.87
6.72
6.57
6.56
3.92
3.85
0.82
2.04
2.07
1.03
0.99
0.96
0.99
3.12
3.01
f1 (ppm)

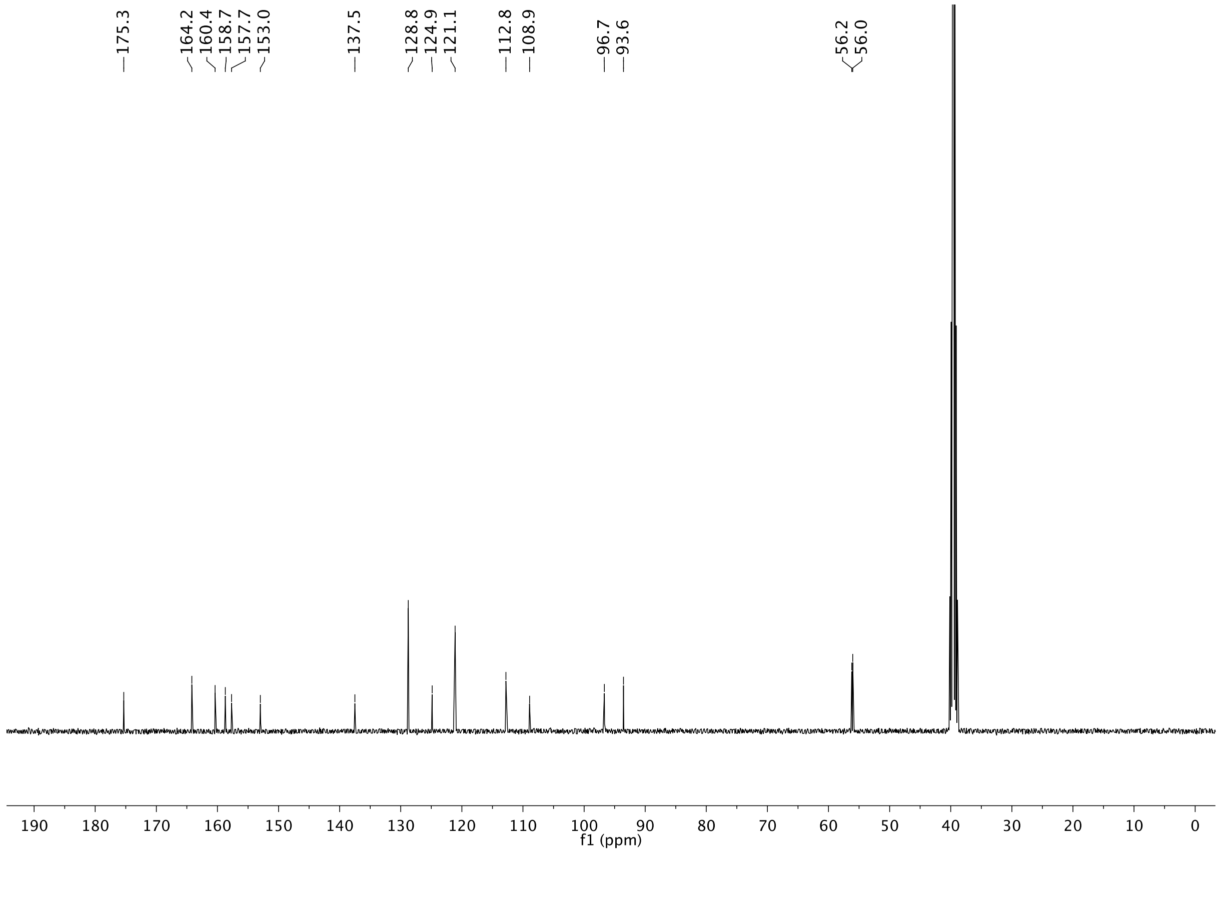

175.3
164.2
160.4
158.7
157.7
153.0
137.5
128.8
124.9
121.1
112.8
108.9
96.7
93.6
56.2
56.0
f1 (ppm)

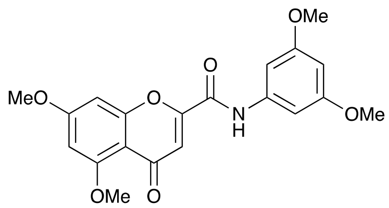
OMe
O
MeO
O
N
H
OMe
OMe
O

16b

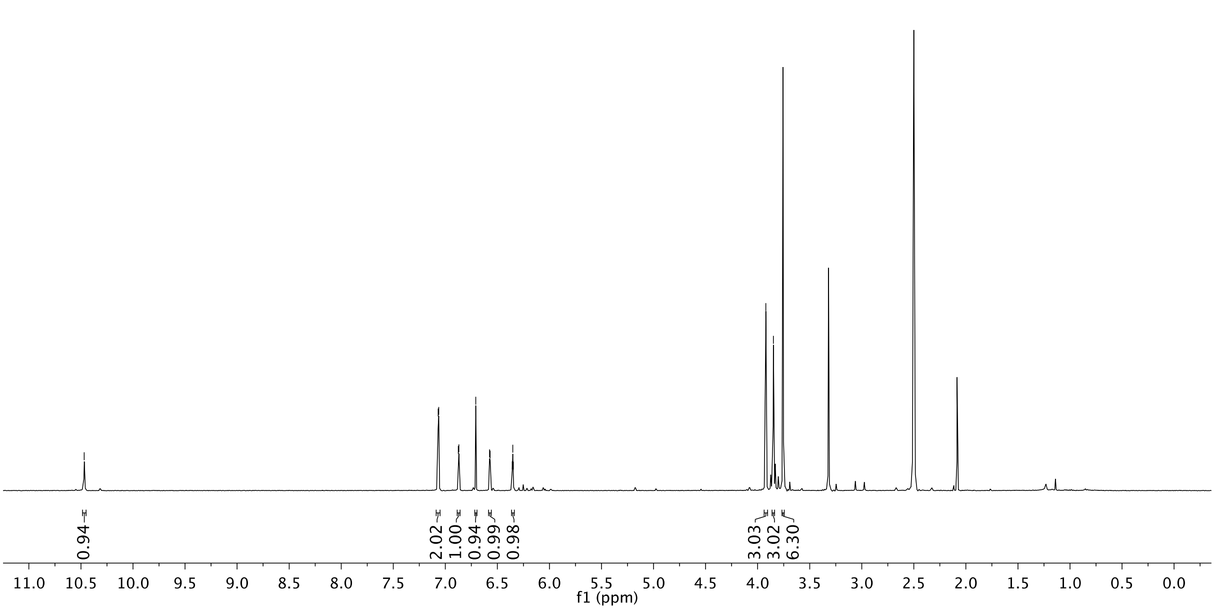
10.47
7.07
7.06
6.88
6.87
6.71
6.58
6.57
6.36
6.35
6.35
3.92
3.85
3.76
0.94
2.02
1.00
0.94
0.99
0.98
3.03
3.02
6.30
11.0 10.5 10.0 9.5 9.0 8.5 8.0 7.5 7.0 6.5 6.0 5.5 5.0 4.5 4.0 3.5 3.0 2.5 2.0 1.5 1.0 0.5 0.0
f1 (ppm)

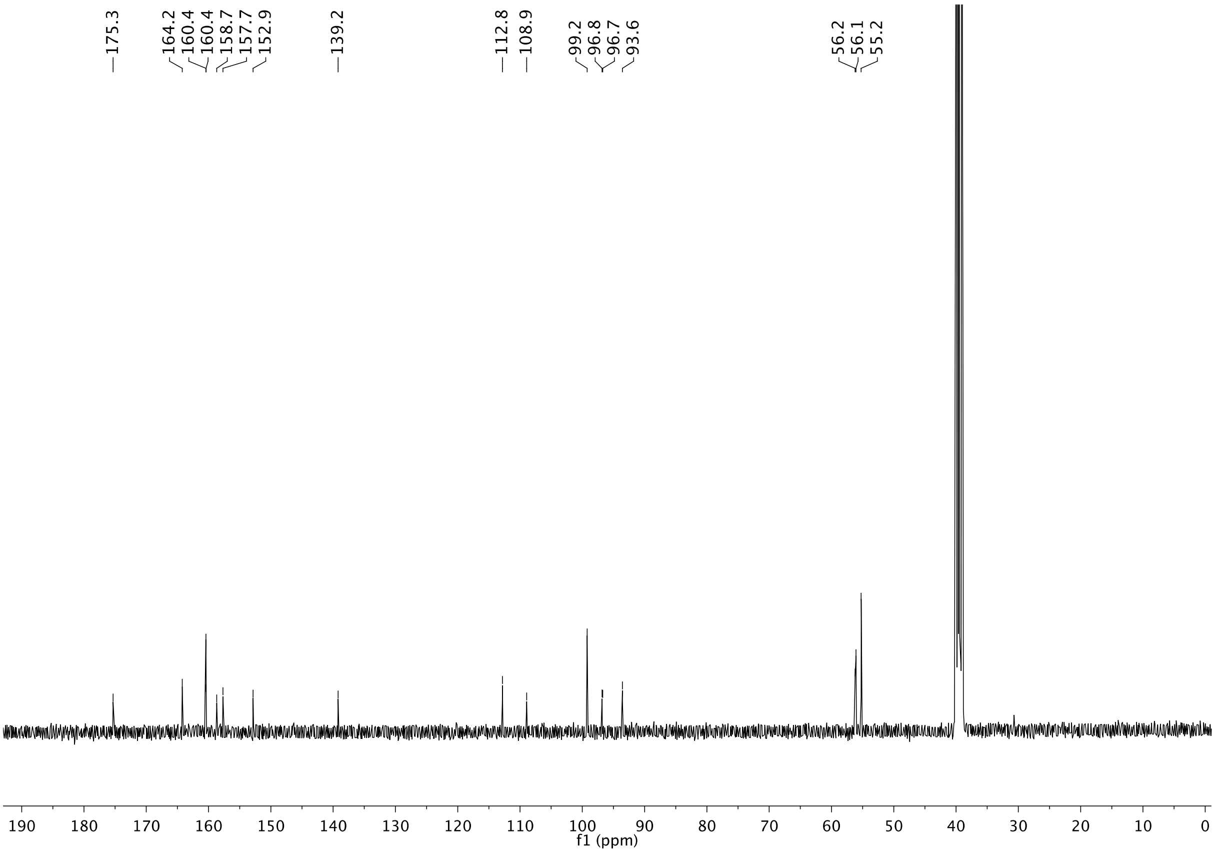
175.3
164.2
160.4
160.4
158.7
157.7
152.9
139.2
112.8
108.9
99.2
96.8
96.7
93.6
56.2
56.1
55.2
190 180 170 160 150 140 130 120 110 100 90 80 70 60 50 40 30 20 10 0
f1 (ppm)

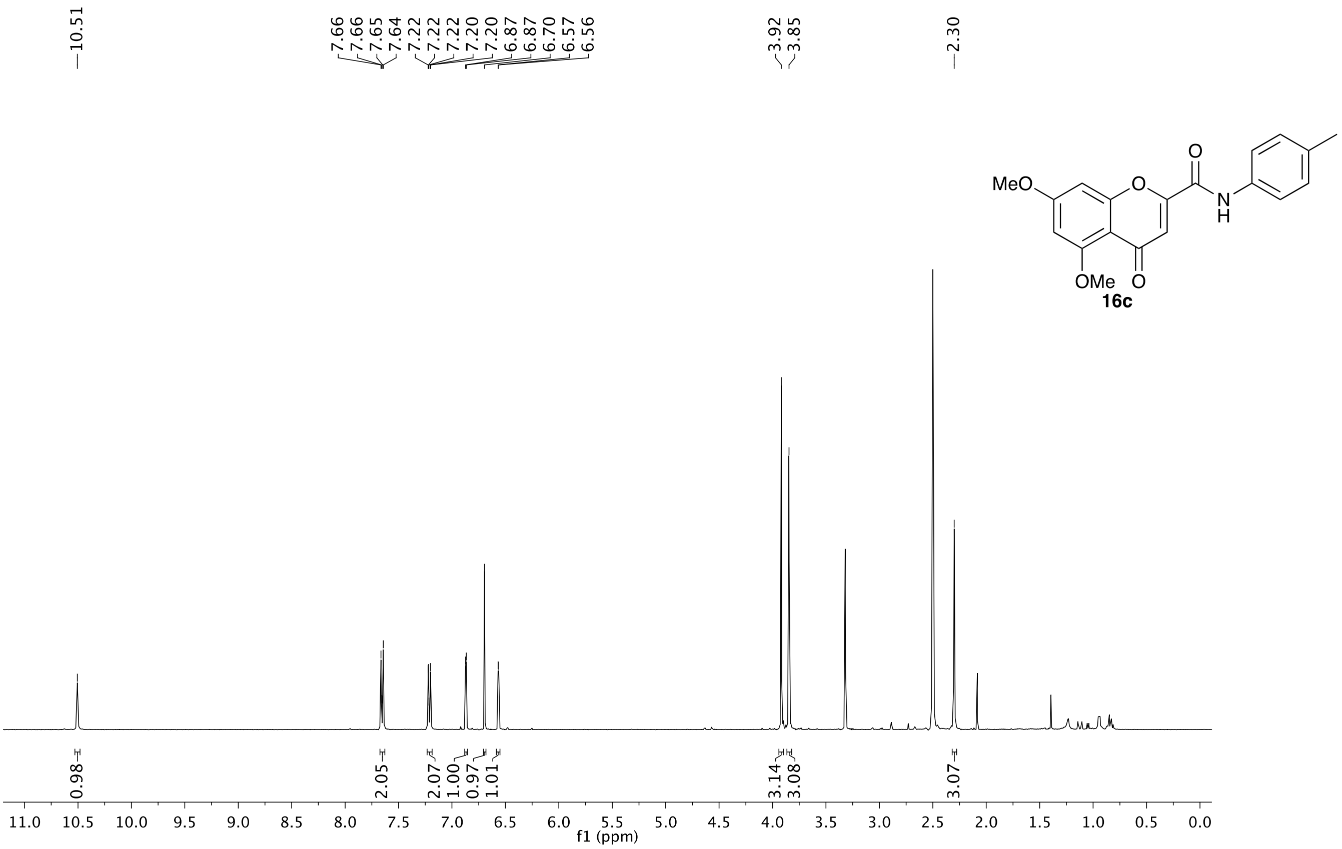
10.51
7.66
7.66
7.65
7.64
7.22
7.22
7.22
7.20
7.20
6.87
6.87
6.70
6.57
6.56
3.92
3.85
2.30
MeO
O
O
N
H
OMe
O
16c
0.98
2.05
2.07
1.00
0.97
1.01
3.14
3.08
3.07
11.0 10.5 10.0 9.5 9.0 8.5 8.0 7.5 7.0 6.5 6.0 5.5 5.0 4.5 4.0 3.5 3.0 2.5 2.0 1.5 1.0 0.5 0.0
f1 (ppm)

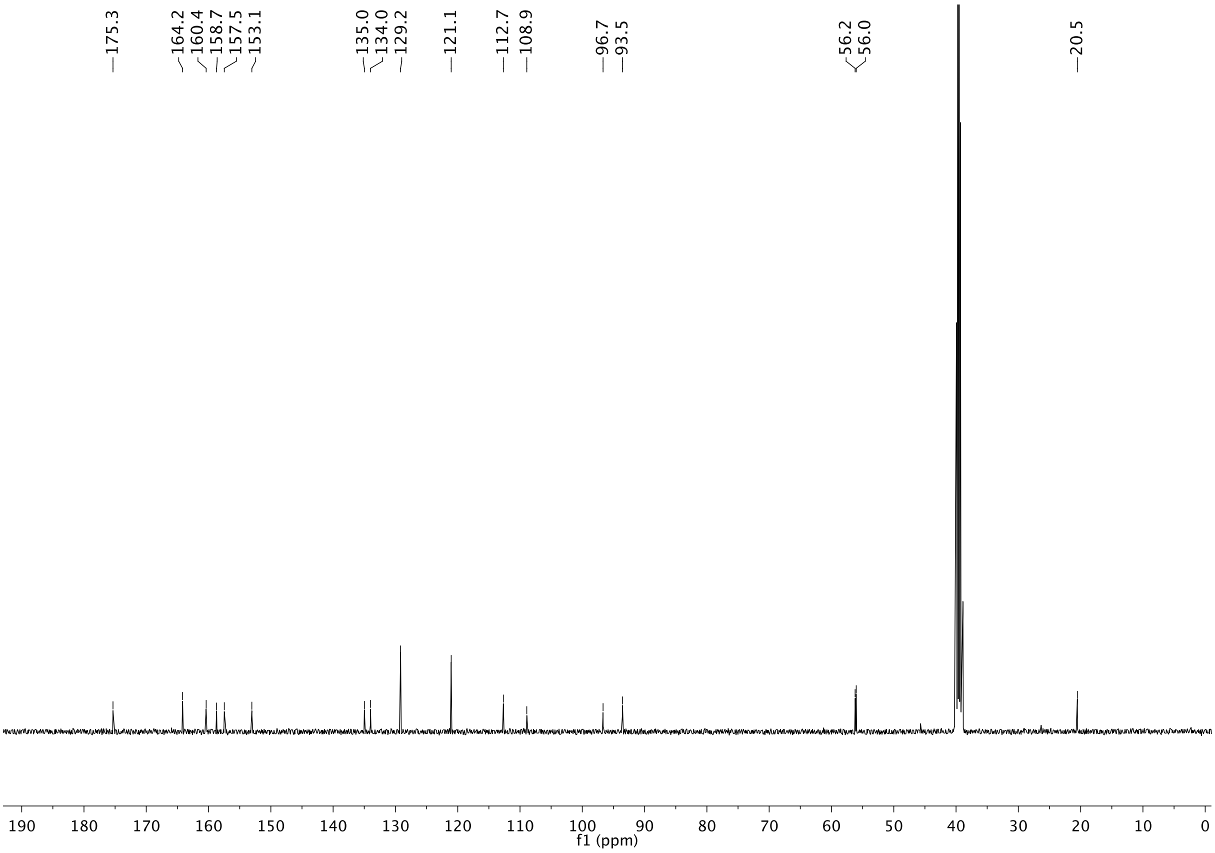
175.3
164.2
160.4
158.7
157.5
153.1
135.0
134.0
129.2
121.1
112.7
108.9
96.7
93.5
56.2
56.0
20.5
190 180 170 160 150 140 130 120 110 100 90 80 70 60 50 40 30 20 10 0
f1 (ppm)

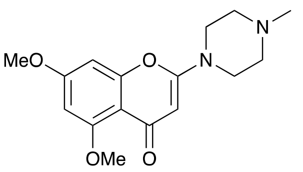
MeO
O
N
N
OMe
O

17a

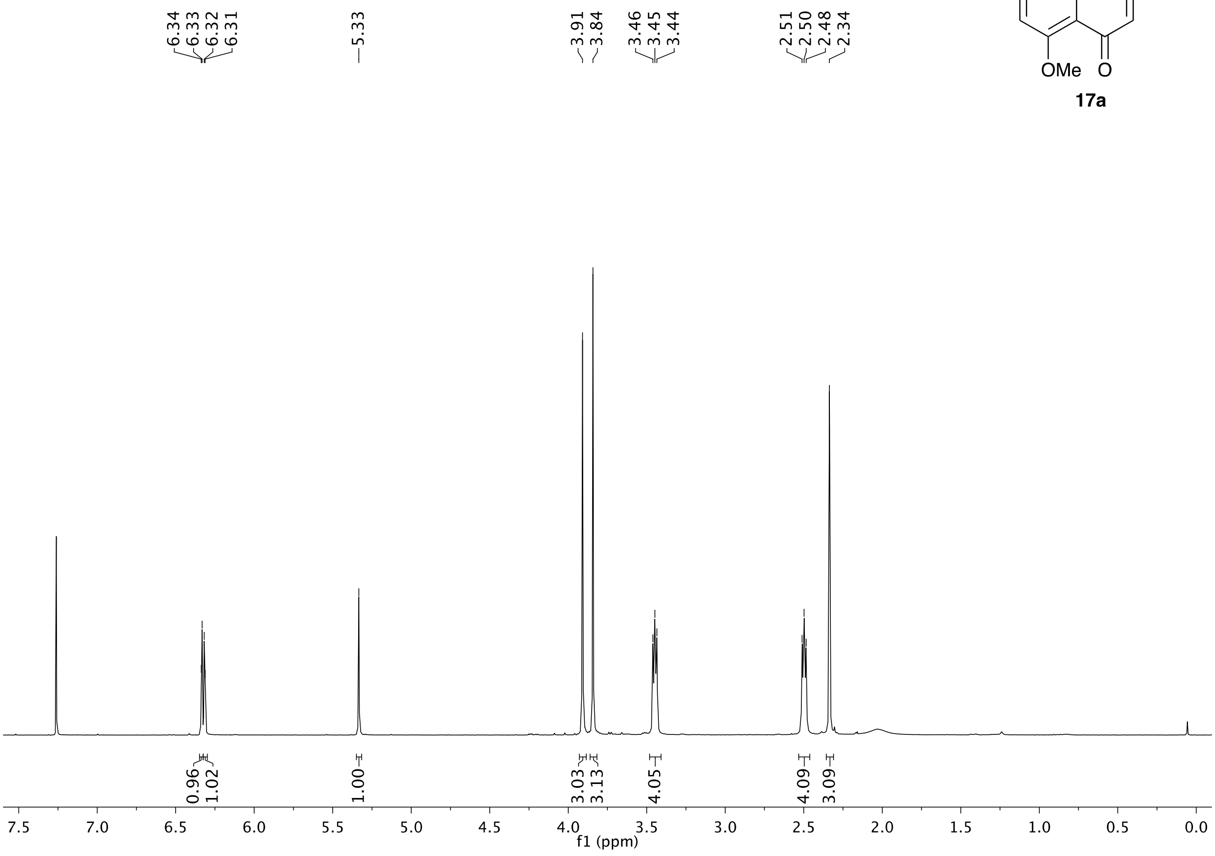
6.34
6.33
6.32
6.31
5.33
3.91
3.84
3.46
3.45
3.44
2.51
2.50
2.48
2.34
0.96
1.02
1.00
3.03
3.13
4.05
4.09
3.09
7.5 7.0 6.5 6.0 5.5 5.0 4.5 4.0 3.5 3.0 2.5 2.0 1.5 1.0 0.5 0.0
f1 (ppm)

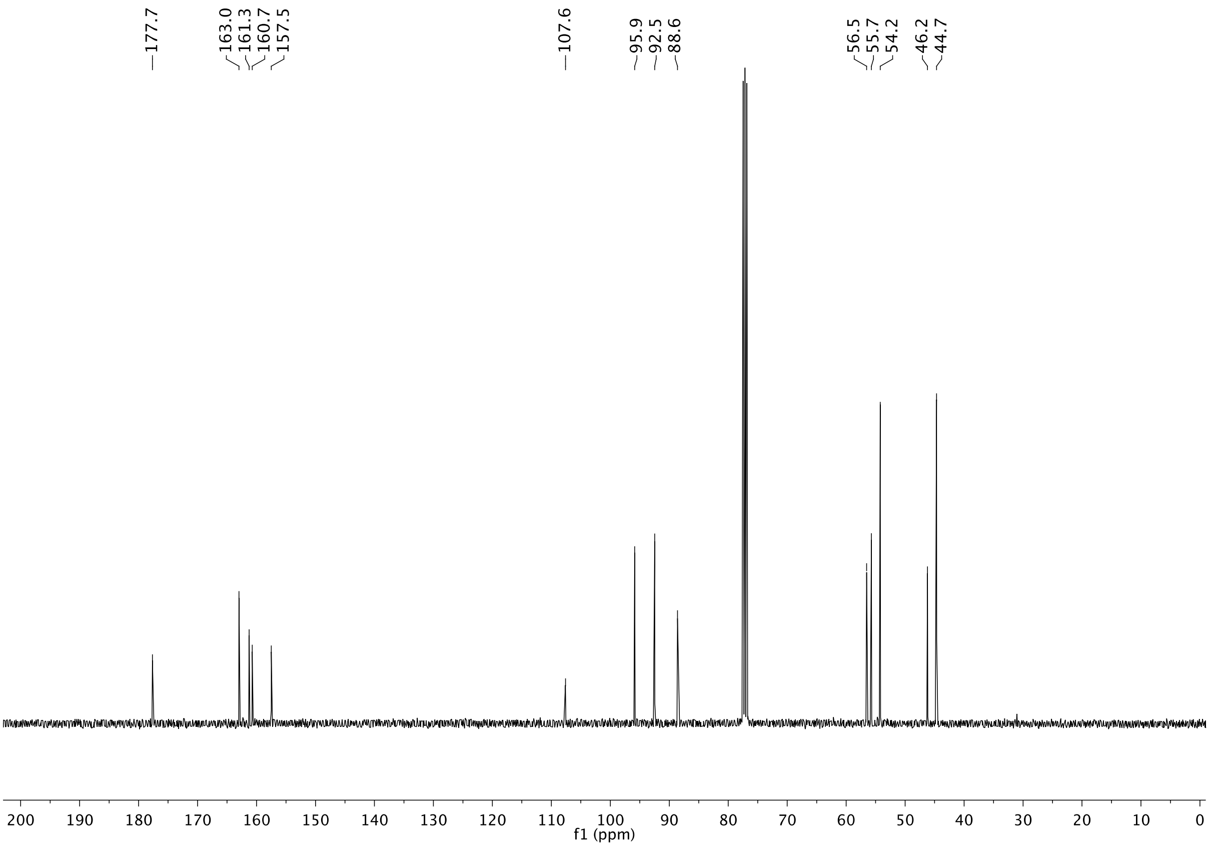
177.7
163.0
161.3
160.7
157.5
107.6
95.9
92.5
88.6
56.5
55.7
54.2
46.2
44.7
200 190 180 170 160 150 140 130 120 110 100 90 80 70 60 50 40 30 20 10 0
f1 (ppm)

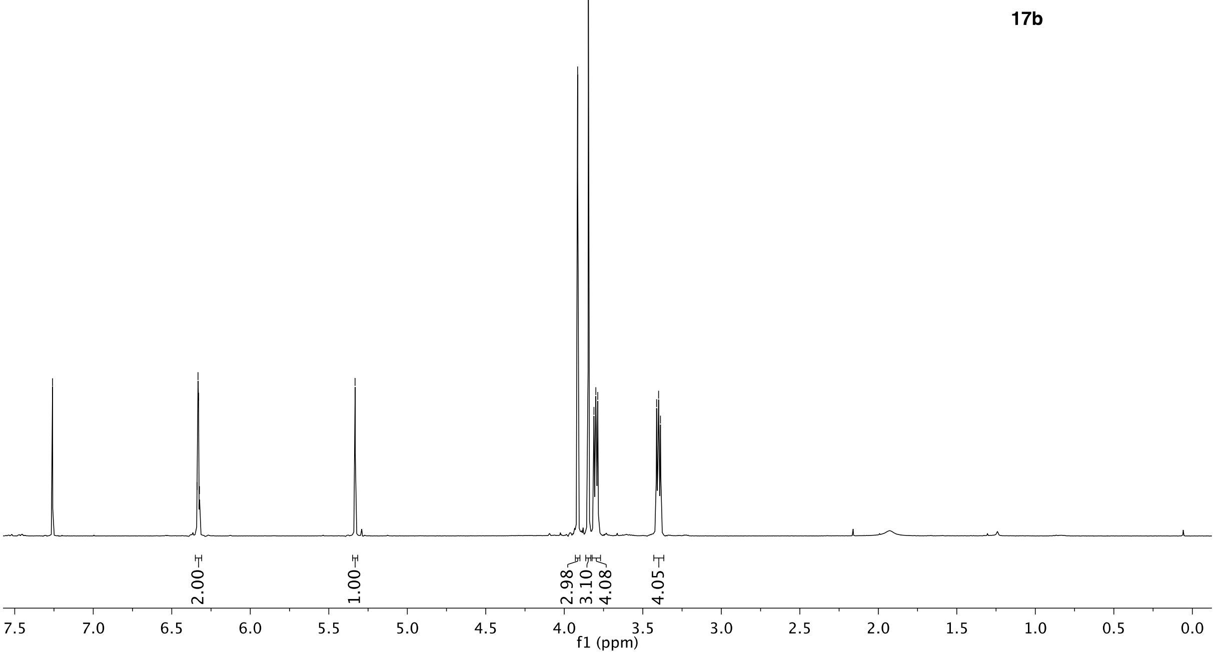
7.26
6.34
6.33
6.33
6.32
5.33
3.92
3.85
3.81
3.80
3.79
3.41
3.40
3.39
2.00
1.00
2.98
3.10
4.08
4.05
7.5 7.0 6.5 6.0 5.5 5.0 4.5 4.0 3.5 3.0 2.5 2.0 1.5 1.0 0.5 0.0
f1 (ppm)

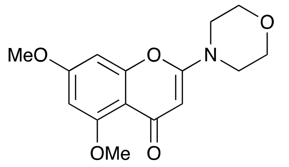
MeO
O
N
O
OMe
O

17b

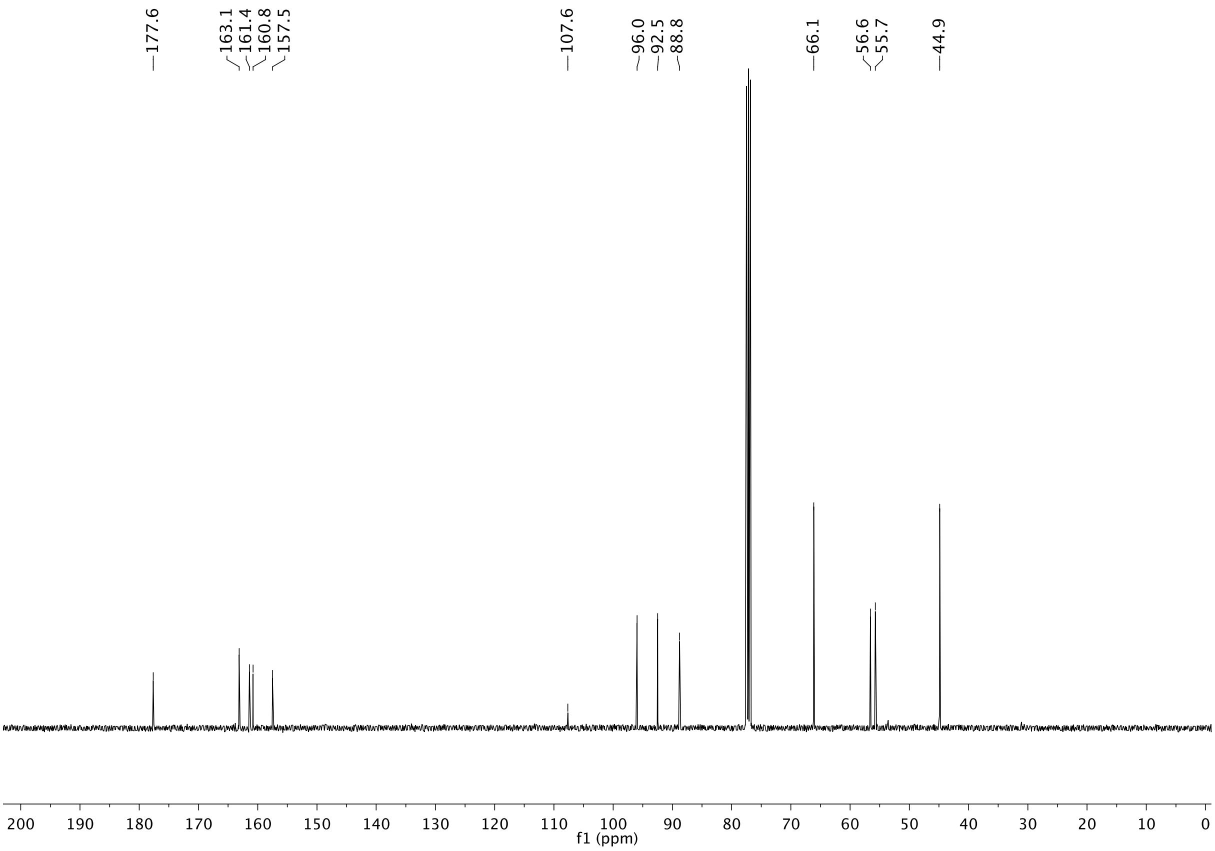
177.6
163.1
161.4
160.8
157.5
107.6
96.0
92.5
88.8
66.1
56.6
55.7
44.9
200 190 180 170 160 150 140 130 120 110 100 90 80 70 60 50 40 30 20 10 0
f1 (ppm)

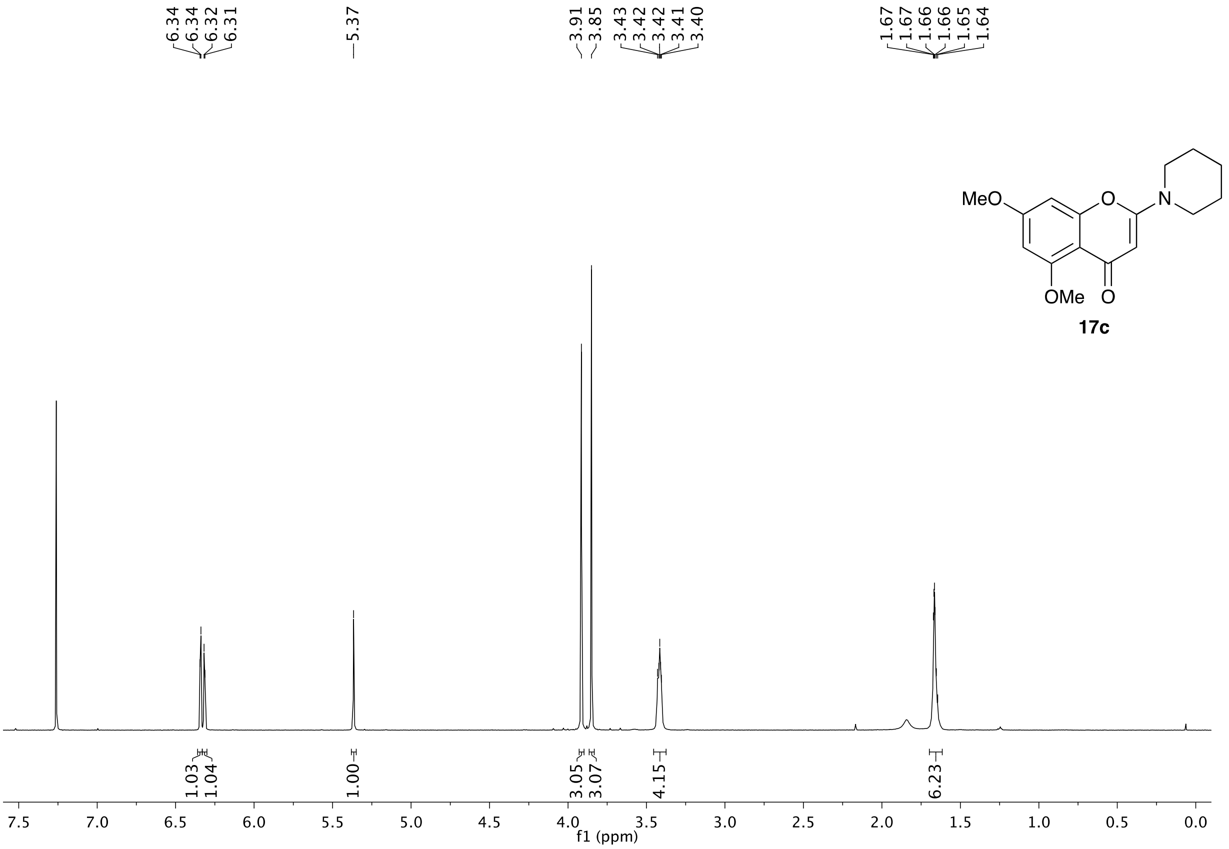
6.34 6.34 6.32 6.31
5.37
3.91 3.85 3.43 3.42 3.42 3.41 3.40
1.67 1.67 1.66 1.66 1.65 1.64
1.03 1.04 1.00 3.05 3.07 4.15 6.23
7.5 7.0 6.5 6.0 5.5 5.0 4.5 4.0 3.5 3.0 2.5 2.0 1.5 1.0 0.5 0.0
f1 (ppm)
MeO
O
N
OMe
O

17c

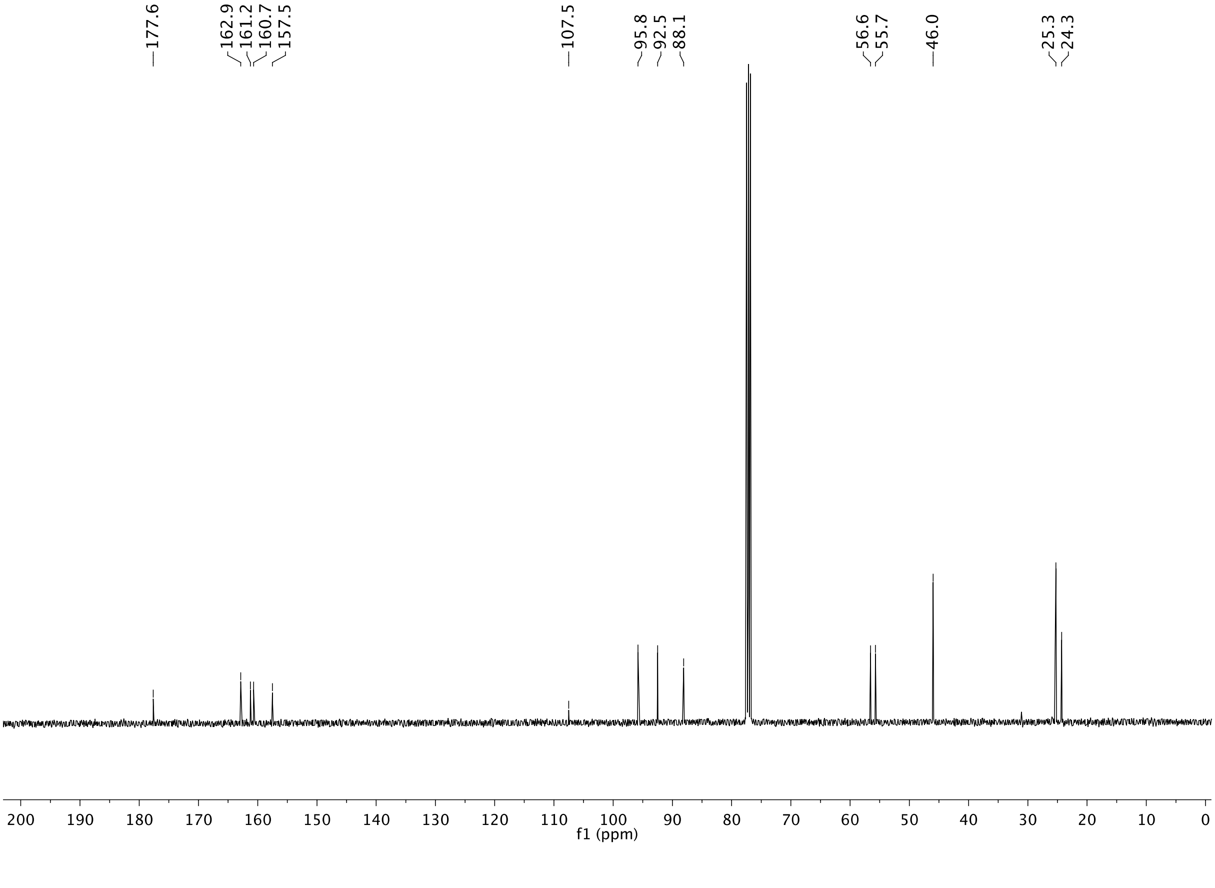
177.6
162.9 161.2 160.7 157.5
107.5
95.8 92.5 88.1
56.6 55.7
46.0
25.3 24.3
200 190 180 170 160 150 140 130 120 110 100 90 80 70 60 50 40 30 20 10 0
f1 (ppm)

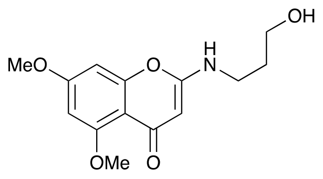
MeO
O
H
N
OH
OMe
O

17d

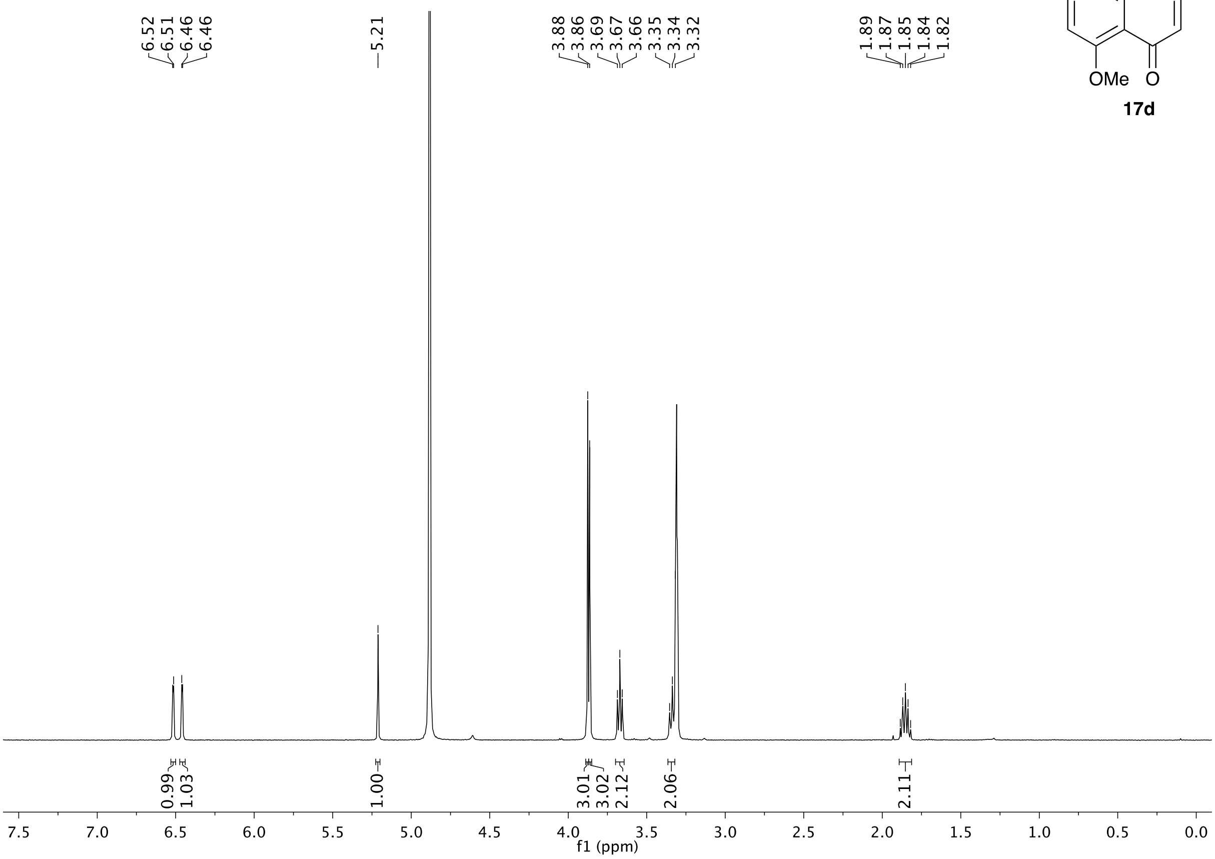
6.52
6.51
6.46
6.46
5.21
3.88
3.86
3.69
3.67
3.66
3.35
3.34
3.32
1.89
1.87
1.85
1.84
1.82
0.99
1.03
1.00
3.01
3.02
2.12
2.06
2.11
7.5 7.0 6.5 6.0 5.5 5.0 4.5 4.0 3.5 3.0 2.5 2.0 1.5 1.0 0.5 0.0
f1 (ppm)

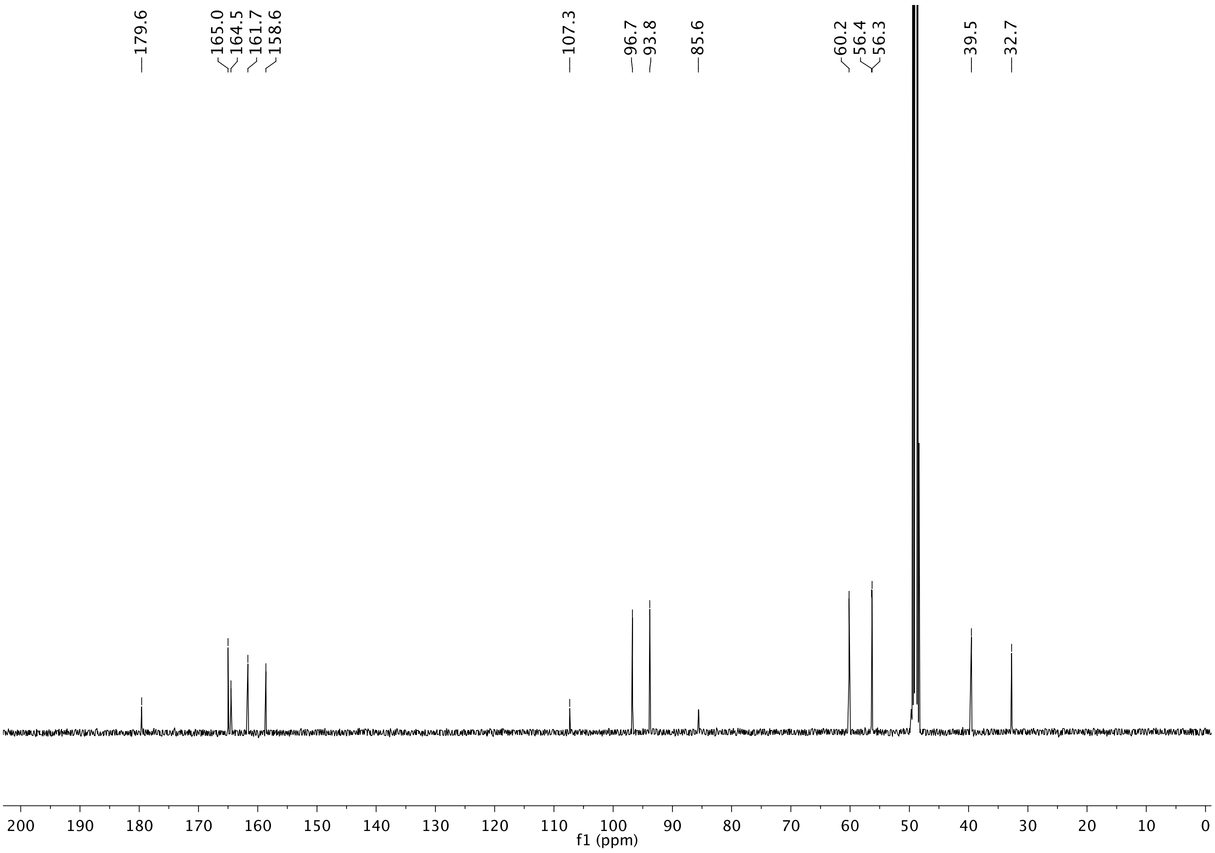
179.6
165.0
164.5
161.7
158.6
107.3
96.7
93.8
85.6
60.2
56.4
56.3
39.5
32.7
200 190 180 170 160 150 140 130 120 110 100 90 80 70 60 50 40 30 20 10 0
f1 (ppm)

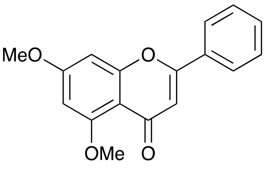
MeO
O
OMe
O

18a

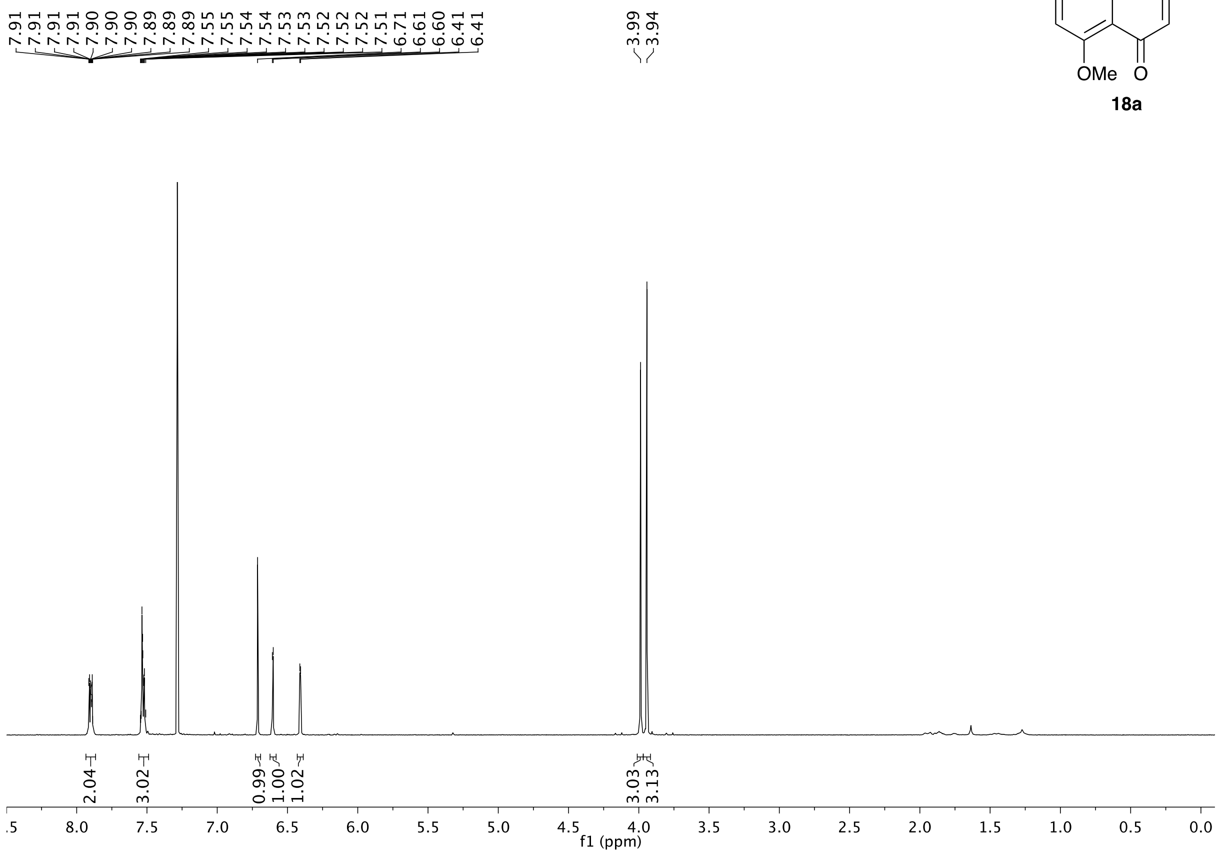
7.91
7.91
7.91
7.91
7.90
7.90
7.89
7.89
7.55
7.55
7.54
7.54
7.53
7.53
7.52
7.52
7.51
6.71
6.61
6.60
6.41
6.41
3.99
3.94
2.04
3.02
0.99
1.00
1.02
3.03
3.13
f1 (ppm)

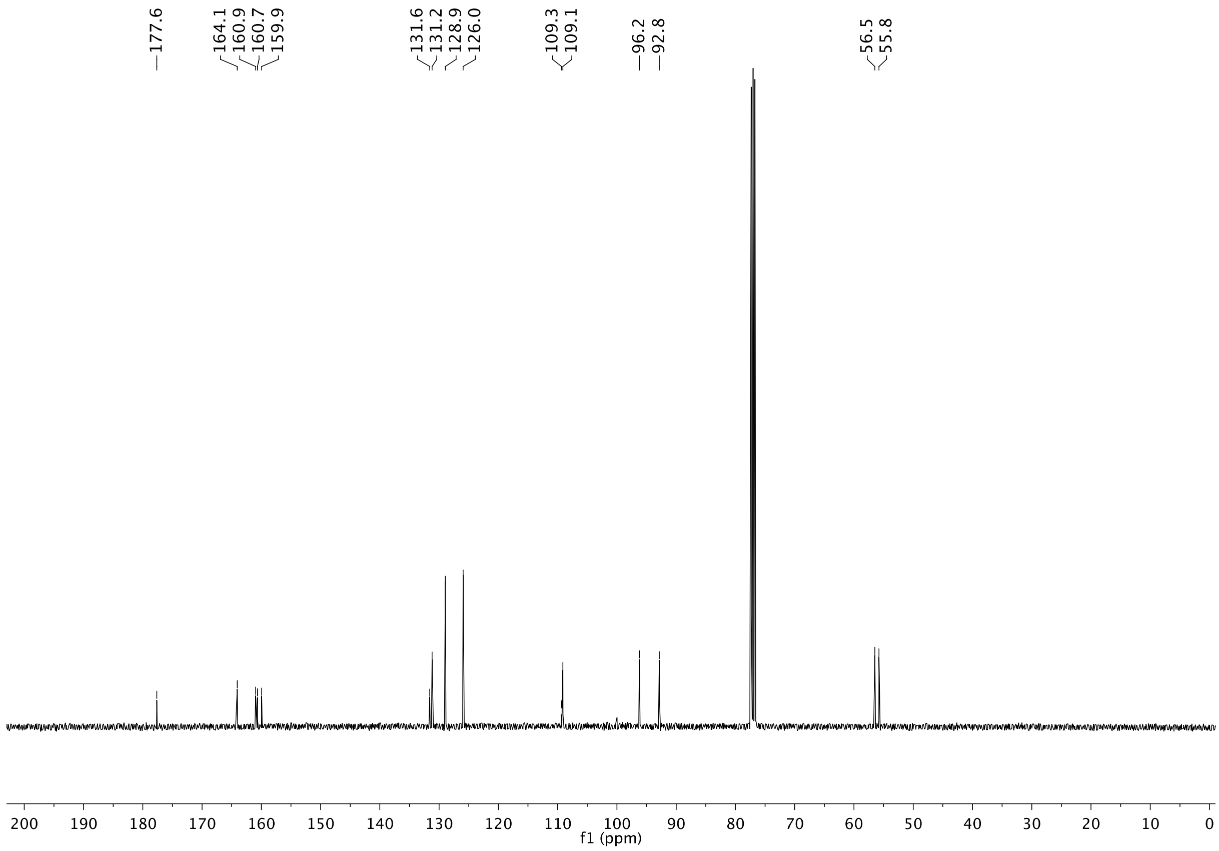
177.6
164.1
160.9
160.7
159.9
131.6
131.2
128.9
126.0
109.3
109.1
96.2
92.8
56.5
55.8
f1 (ppm)

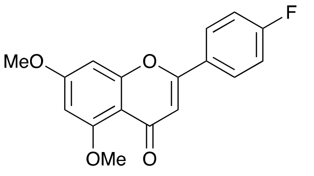
MeO
O
F
OMe
O

18b

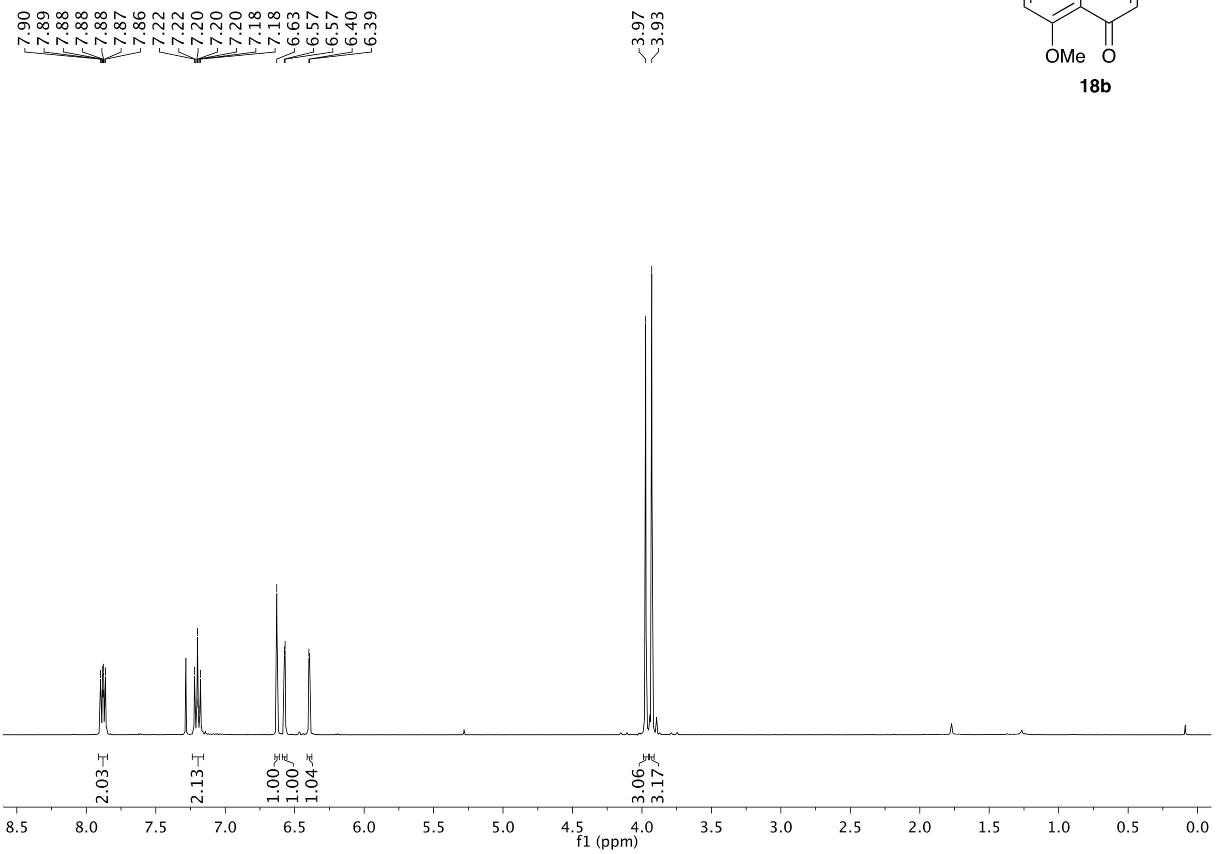
7.90
7.89
7.88
7.88
7.88
7.87
7.86
7.22
7.22
7.20
7.20
7.20
7.18
7.18
6.63
6.57
6.57
6.40
6.39
3.97
3.93
2.03
2.13
1.00
1.00
1.04
3.06
3.17
8.5 8.0 7.5 7.0 6.5 6.0 5.5 5.0 4.5 4.0 3.5 3.0 2.5 2.0 1.5 1.0 0.5 0.0
f1 (ppm)

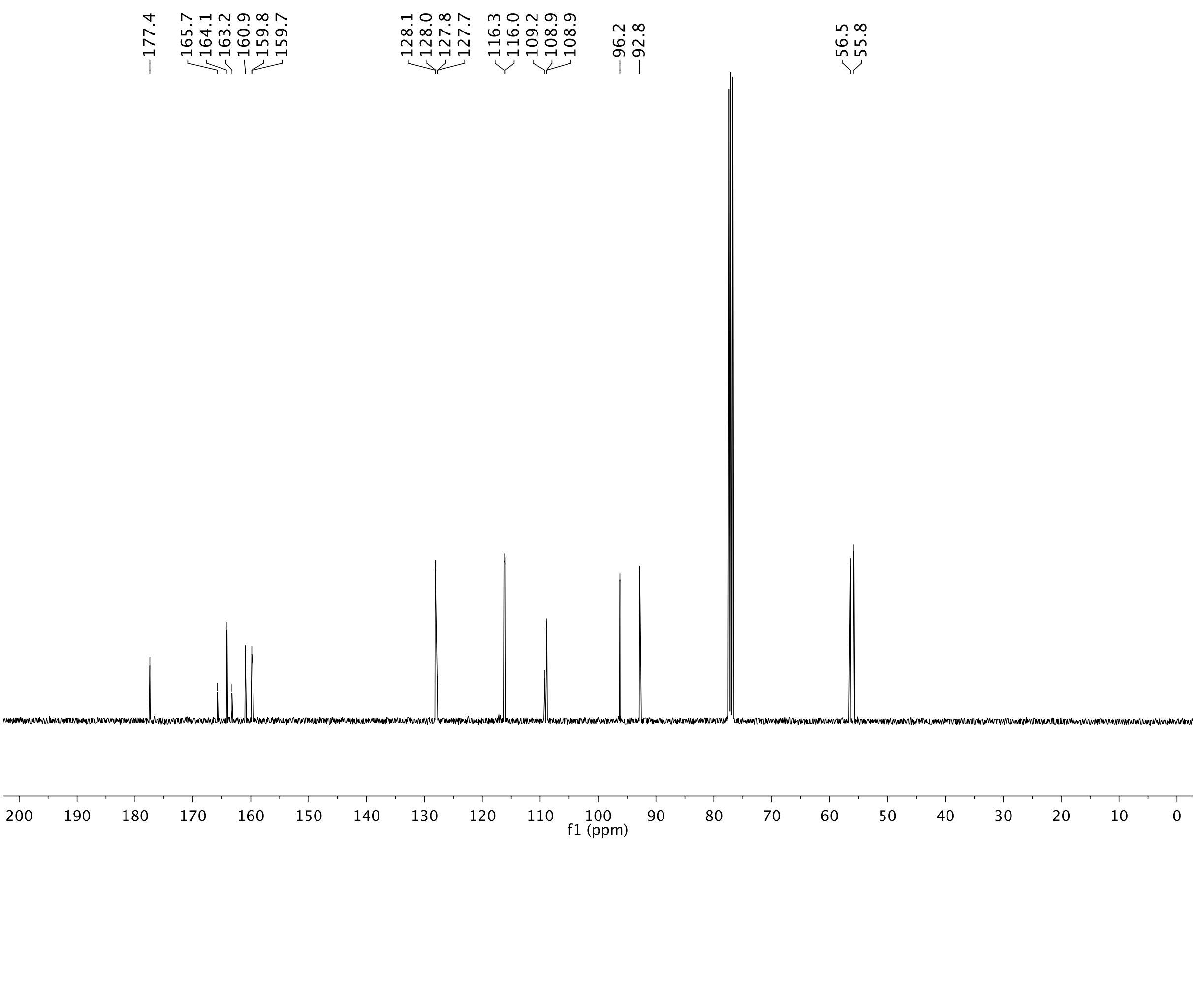
177.4
165.7
164.1
163.2
160.9
159.8
159.7
128.1
128.0
127.8
127.7
116.3
116.0
109.2
108.9
108.9
96.2
92.8
56.5
55.8
200 190 180 170 160 150 140 130 120 110 100 90 80 70 60 50 40 30 20 10 0
f1 (ppm)

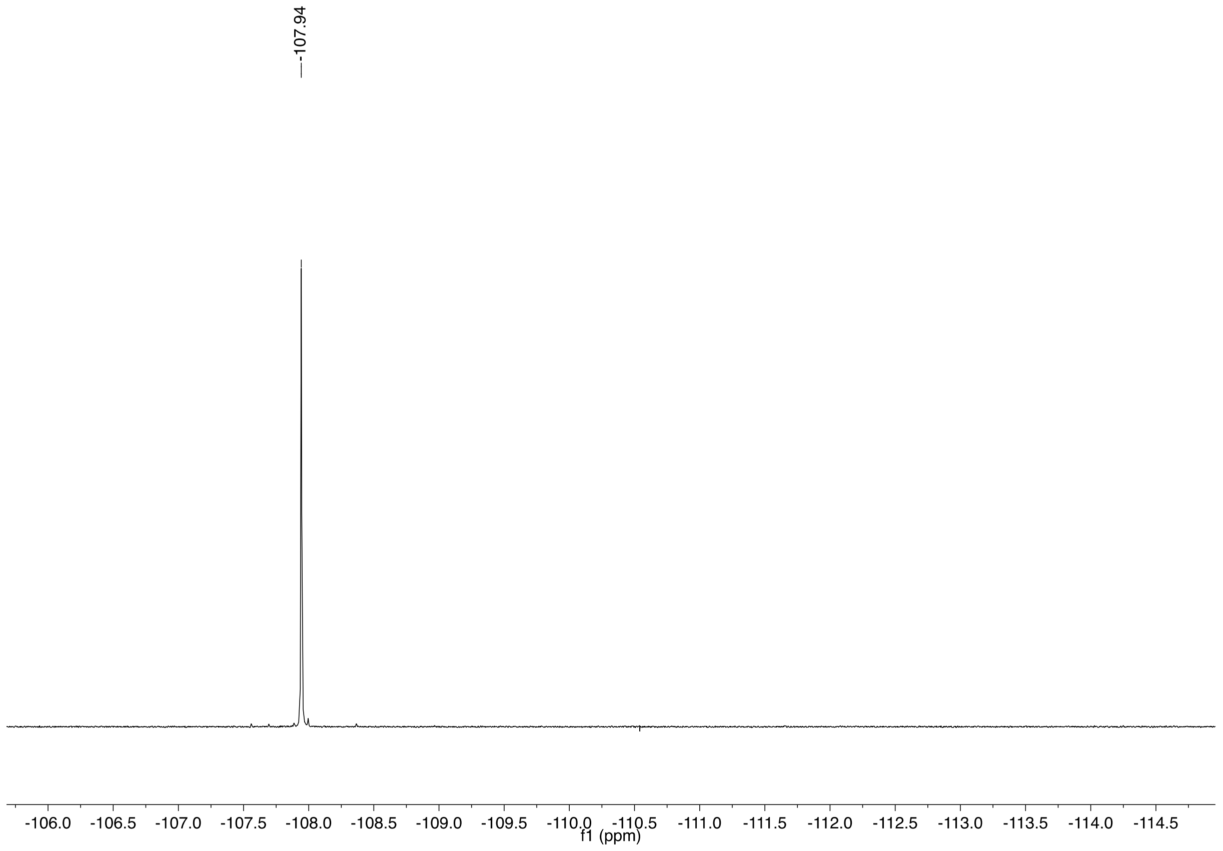

-107.94
-106.0 -106.5 -107.0 -107.5 -108.0 -108.5 -109.0 -109.5 -110.0 -110.5 -111.0 -111.5 -112.0 -112.5 -113.0 -113.5 -114.0 -114.5
f1 (ppm)

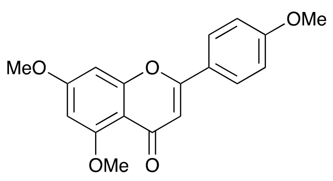
MeO
OMe
OMe
O

18c

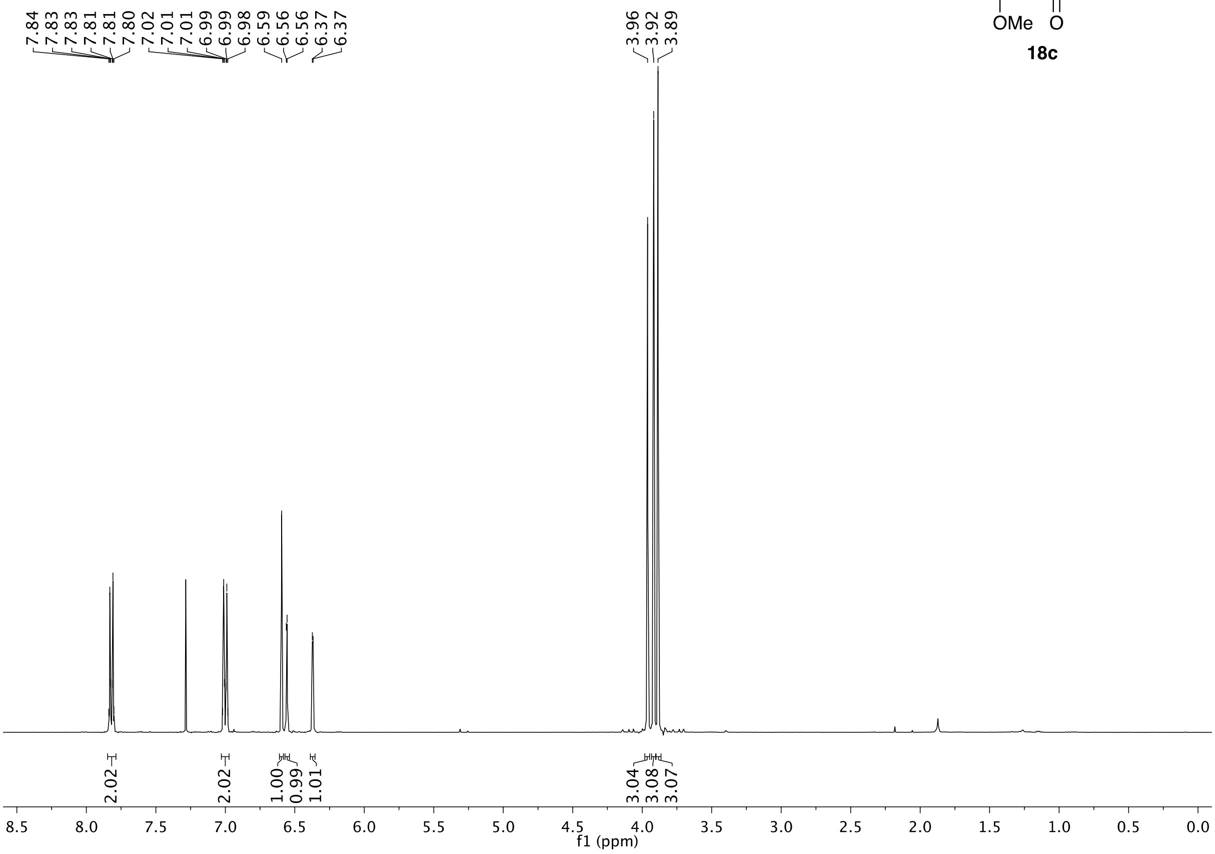
7.84
7.83
7.83
7.81
7.81
7.80
7.02
7.01
7.01
6.99
6.99
6.98
6.59
6.56
6.56
6.37
6.37
3.96
3.92
3.89
2.02
2.02
1.00
0.99
1.01
3.04
3.08
3.07
f1 (ppm)

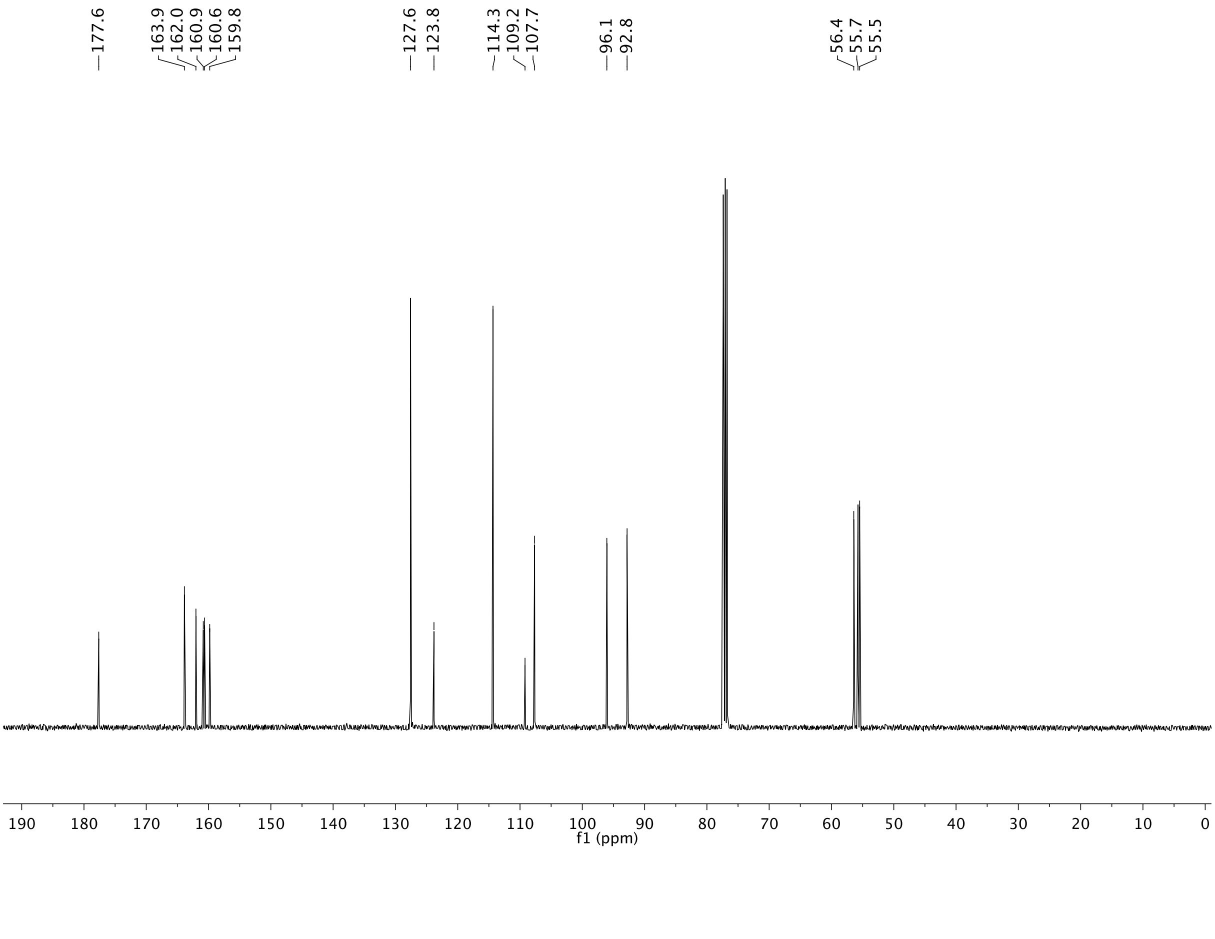
177.6
163.9
162.0
160.9
160.6
159.8
127.6
123.8
114.3
109.2
107.7
96.1
92.8
56.4
55.7
55.5
f1 (ppm)

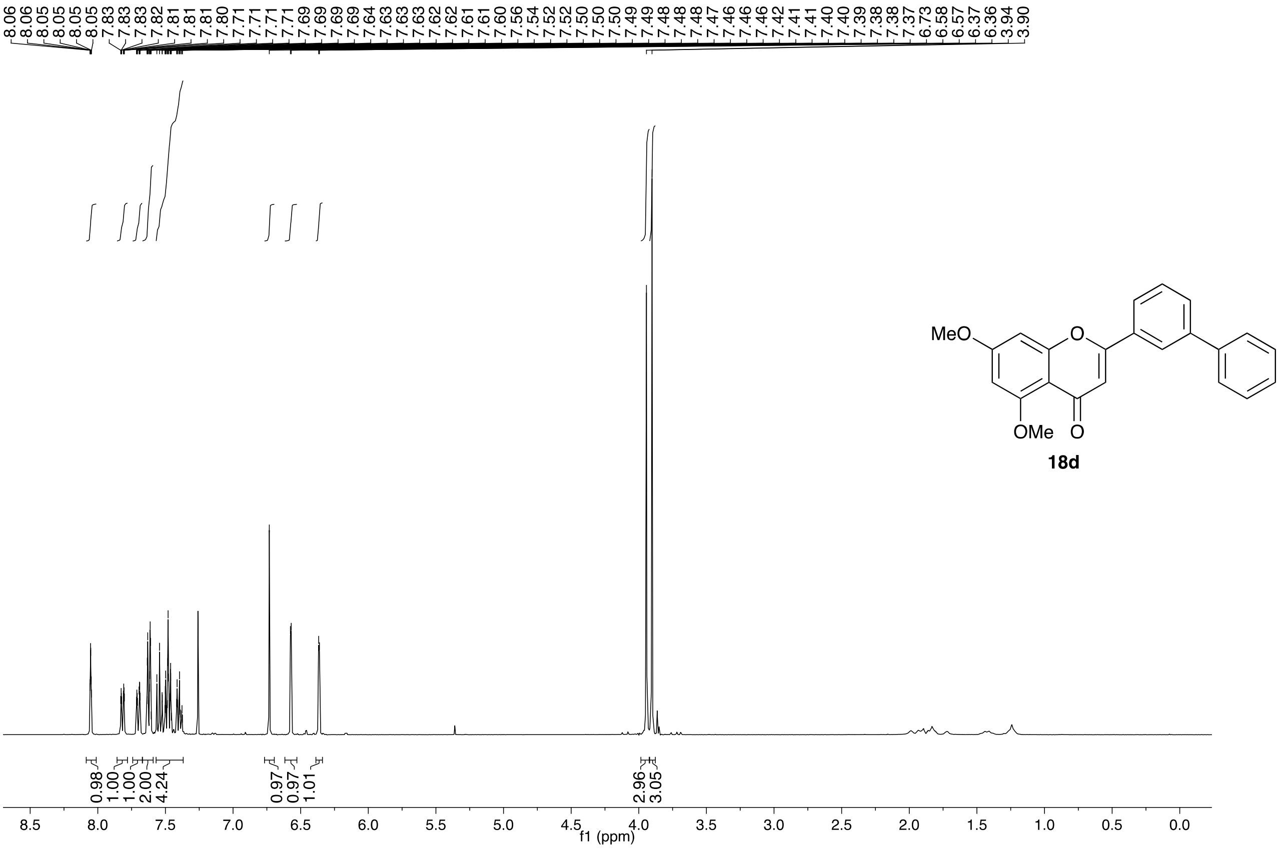
8.06 8.06 8.05 8.05 8.05 7.83 7.83 7.83 7.82 7.81 7.81 7.80 7.71 7.71 7.71 7.69 7.69 7.69 7.64 7.63 7.63 7.62 7.61 7.61 7.60 7.56 7.54 7.52 7.52 7.50 7.50 7.49 7.49 7.48 7.48 7.47 7.46 7.46 7.42 7.41 7.40 7.39 7.38 7.38 7.37 6.73 6.58 6.57 6.37 6.36 3.94 3.90
0.98 1.00 1.00 2.00 4.24 0.97 0.97 1.01 2.96 3.05
f1 (ppm)
MeO
O
OMe
O

18d

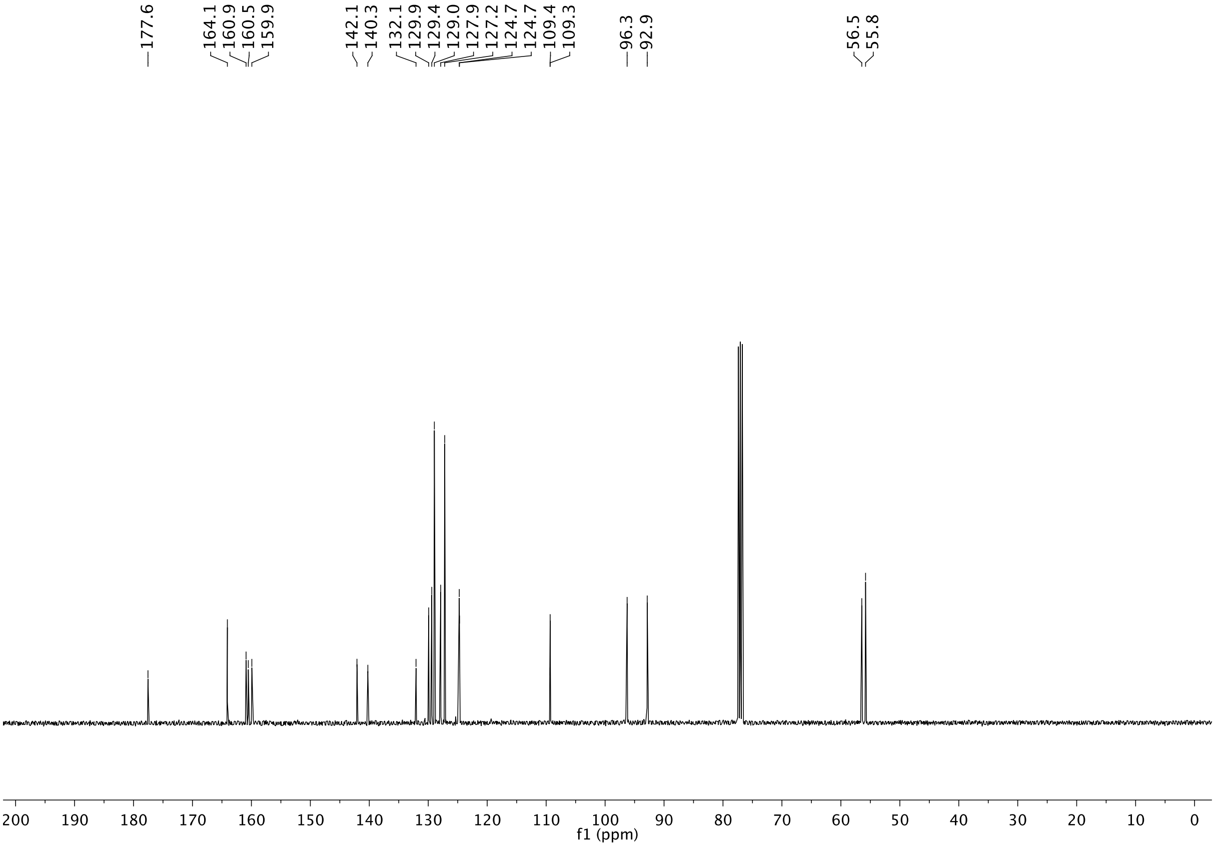
177.6 164.1 160.9 160.5 159.9 142.1 140.3 132.1 129.9 129.4 129.0 127.9 127.2 124.7 124.7 109.4 109.3 96.3 92.9 56.5 55.8
f1 (ppm)

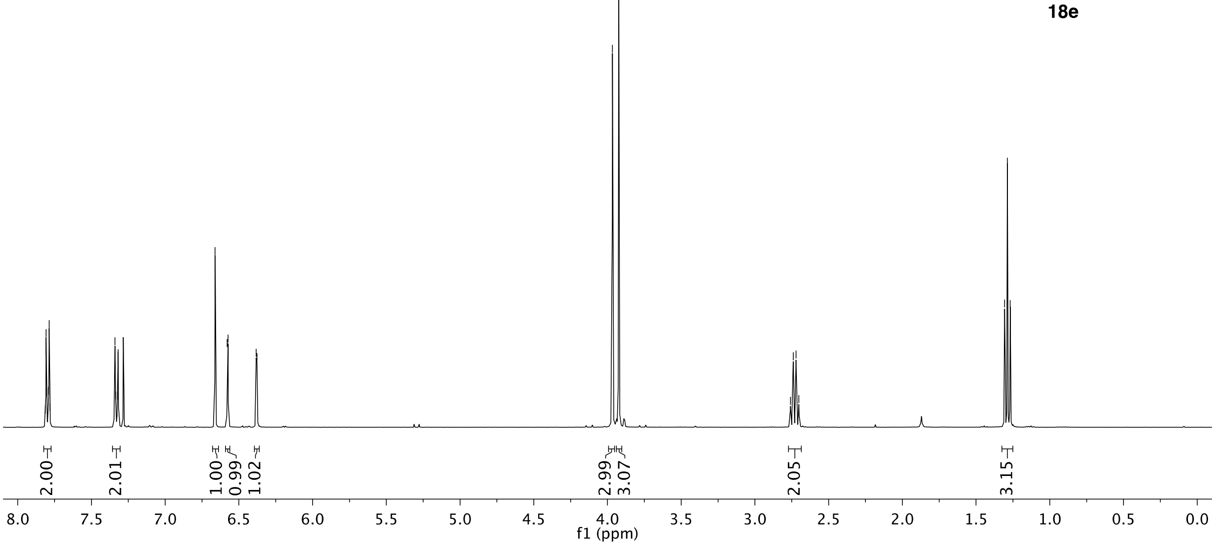
7.81 7.81 7.80 7.79 7.79 7.78 7.34 7.34 7.34 7.32 7.32 7.32 6.66 6.58 6.57 6.38 6.38
3.97 3.92
2.76 2.74 2.72 2.70
1.31 1.29 1.27
2.00 2.01 1.00 0.99 1.02 2.99 3.07 2.05 3.15
8.0 7.5 7.0 6.5 6.0 5.5 5.0 4.5 4.0 3.5 3.0 2.5 2.0 1.5 1.0 0.5 0.0
f1 (ppm)

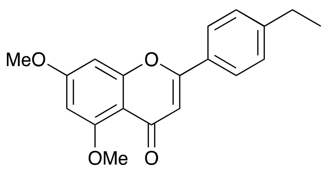
MeO
O
OMe
O

18e

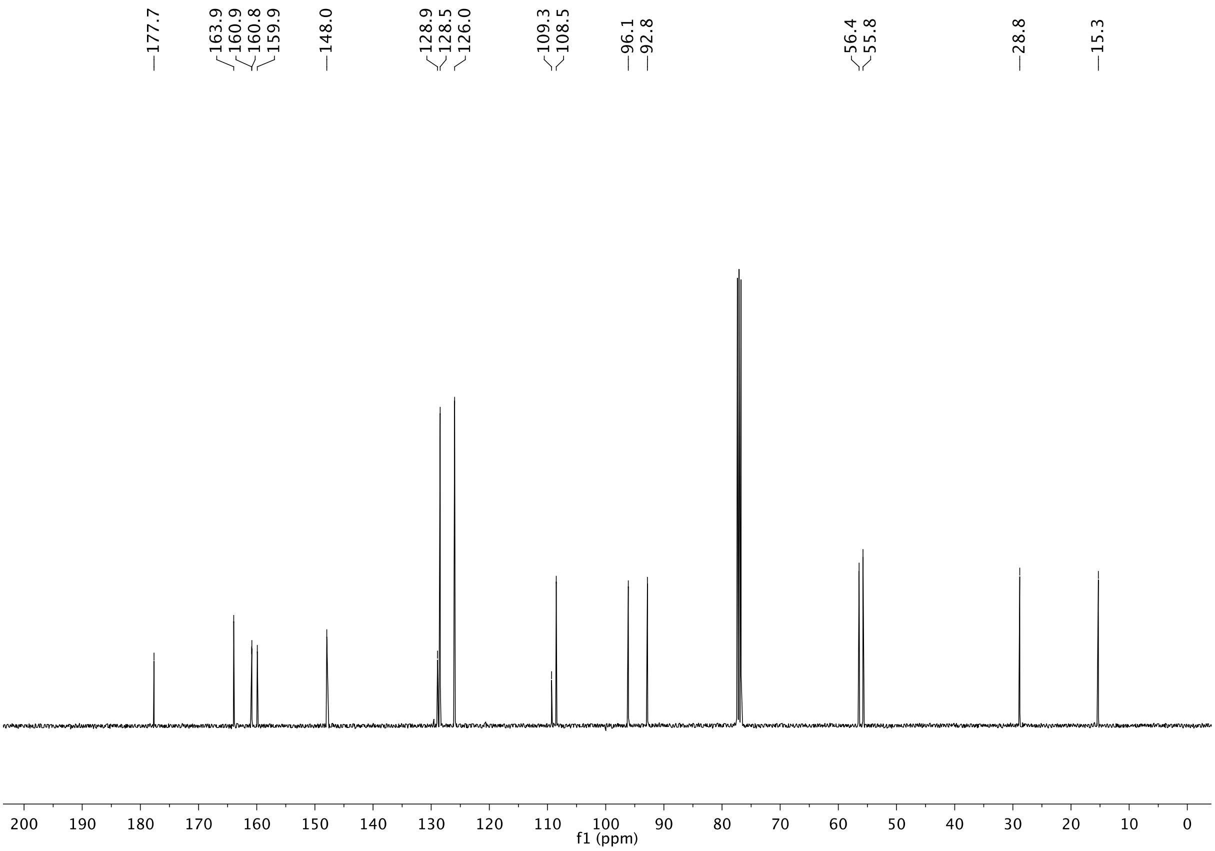
177.7
163.9 160.9 160.8 159.9
148.0
128.9 128.5 126.0
109.3 108.5
96.1 92.8
56.4 55.8
28.8
15.3
200 190 180 170 160 150 140 130 120 110 100 90 80 70 60 50 40 30 20 10 0
f1 (ppm)

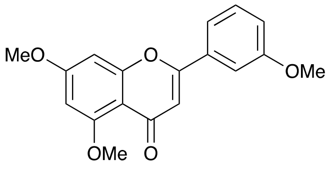
MeO
O
OMe
OMe
O

18f

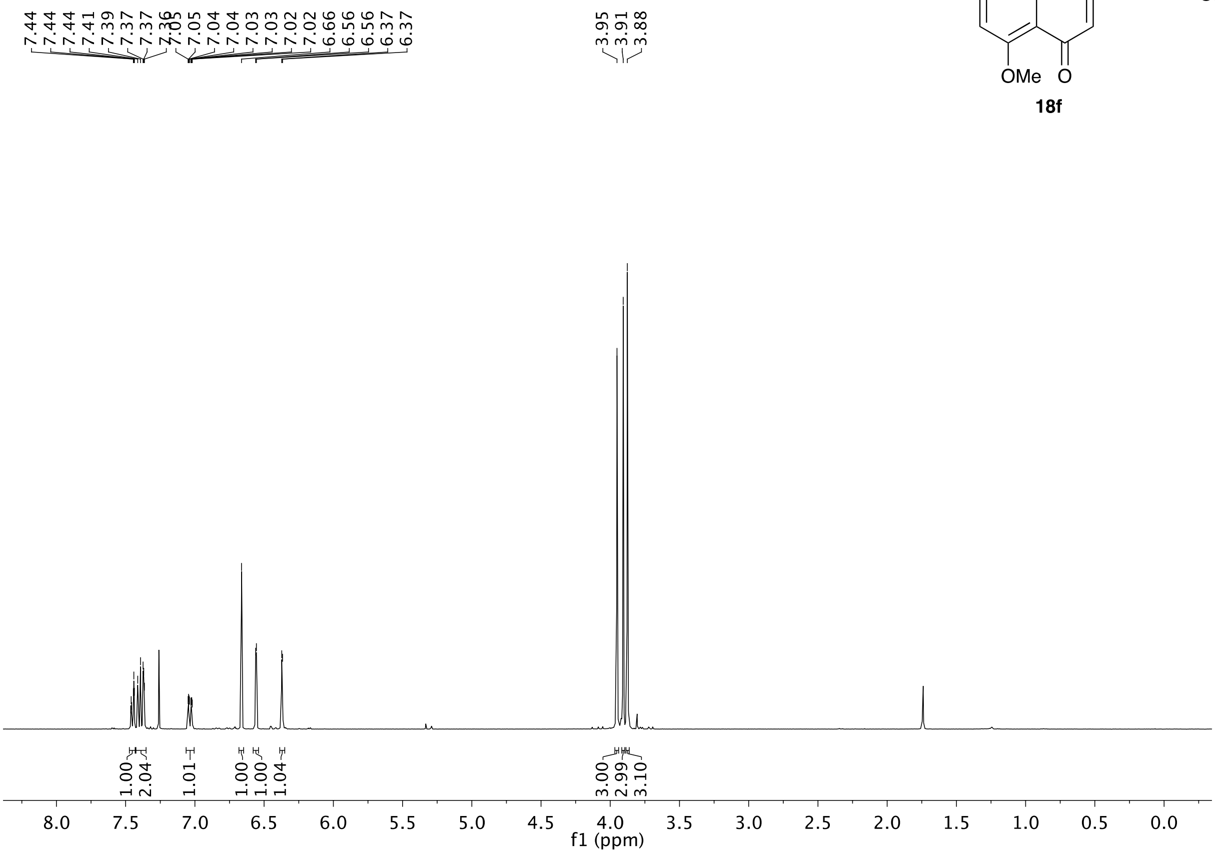
7.44
7.44
7.44
7.41
7.39
7.37
7.37
7.36
7.05
7.05
7.04
7.04
7.03
7.03
7.02
7.02
6.66
6.56
6.56
6.37
6.37
3.95
3.91
3.88
1.00
2.04
1.01
1.00
1.00
1.04
3.00
2.99
3.10
8.0 7.5 7.0 6.5 6.0 5.5 5.0 4.5 4.0 3.5 3.0 2.5 2.0 1.5 1.0 0.5 0.0
f1 (ppm)

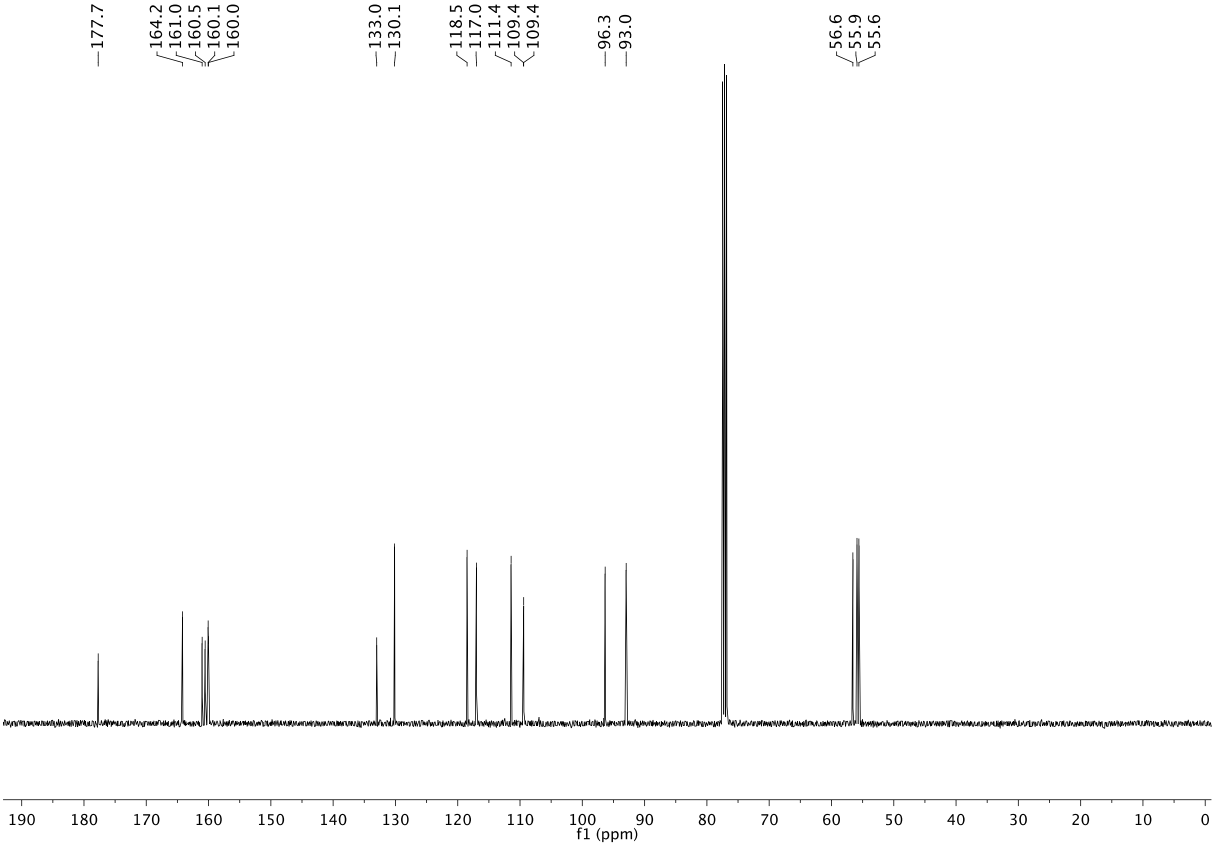
177.7
164.2
161.0
160.5
160.1
160.0
133.0
130.1
118.5
117.0
111.4
109.4
109.4
96.3
93.0
56.6
55.9
55.6
190 180 170 160 150 140 130 120 110 100 90 80 70 60 50 40 30 20 10 0
f1 (ppm)

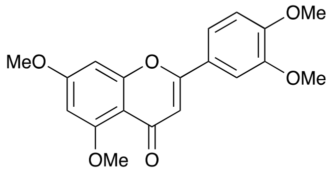
MeO
OMe
OMe
OMe
O

18g

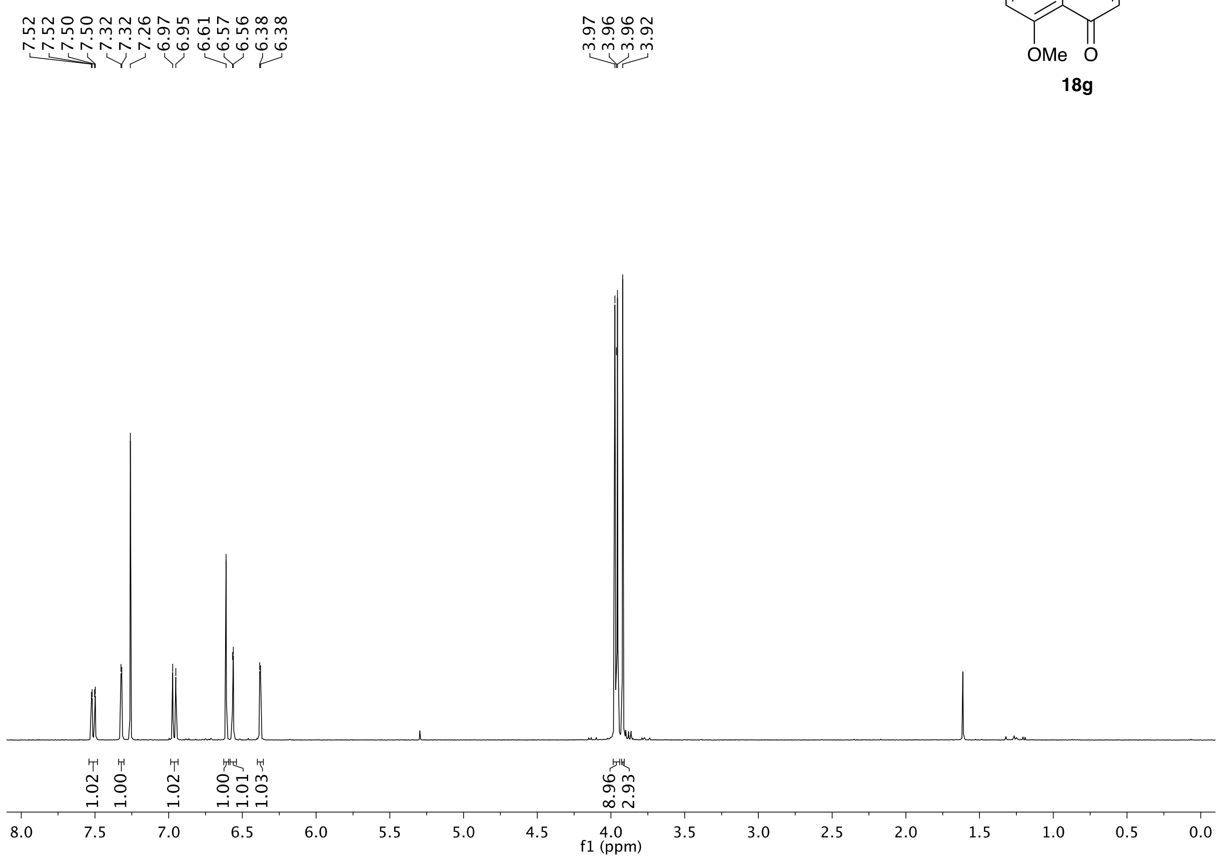
7.52
7.52
7.50
7.50
7.32
7.32
7.26
6.97
6.95
6.61
6.57
6.56
6.38
6.38
3.97
3.96
3.96
3.92
1.02
1.00
1.02
1.00
1.01
1.03
8.96
2.93
8.0 7.5 7.0 6.5 6.0 5.5 5.0 4.5 4.0 3.5 3.0 2.5 2.0 1.5 1.0 0.5 0.0
f1 (ppm)

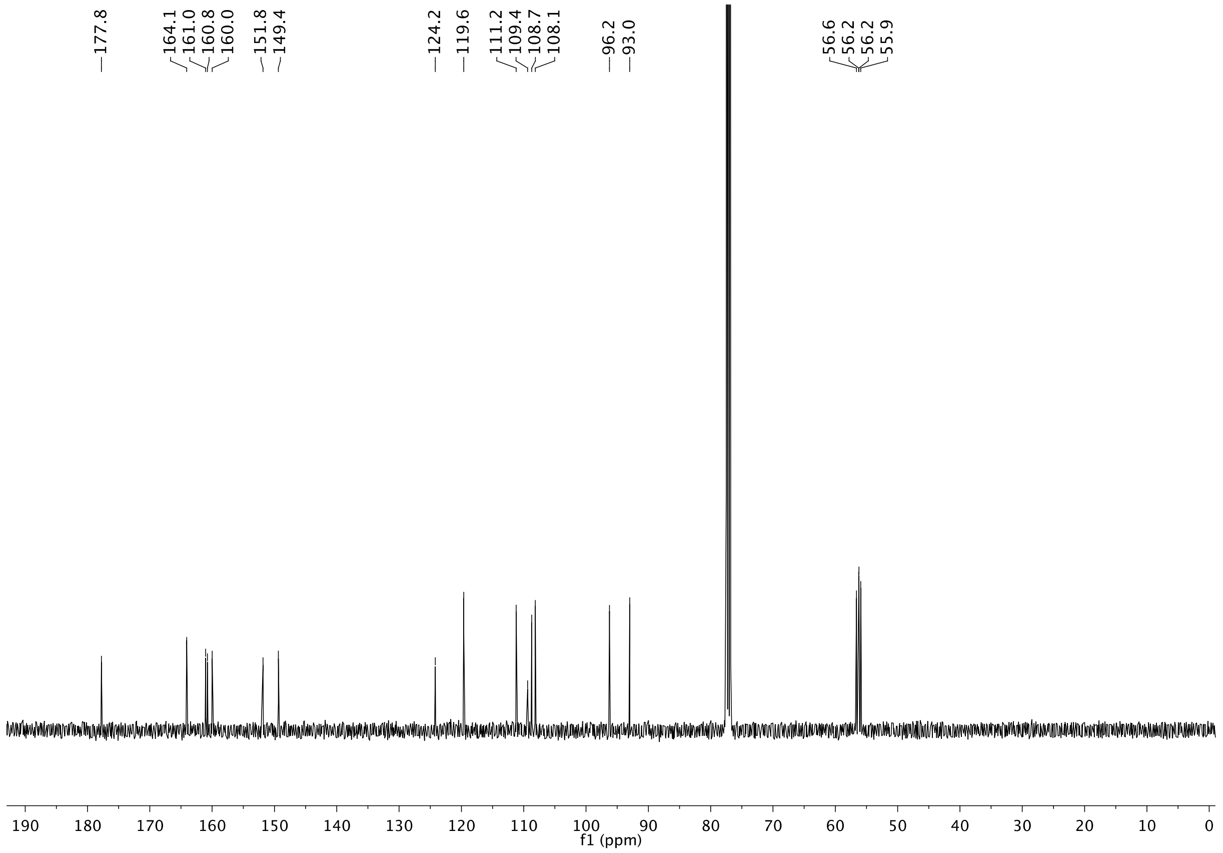
177.8
164.1
161.0
160.8
160.0
151.8
149.4
124.2
119.6
111.2
109.4
108.7
108.1
96.2
93.0
56.6
56.2
56.2
55.9
190 180 170 160 150 140 130 120 110 100 90 80 70 60 50 40 30 20 10 0
f1 (ppm)

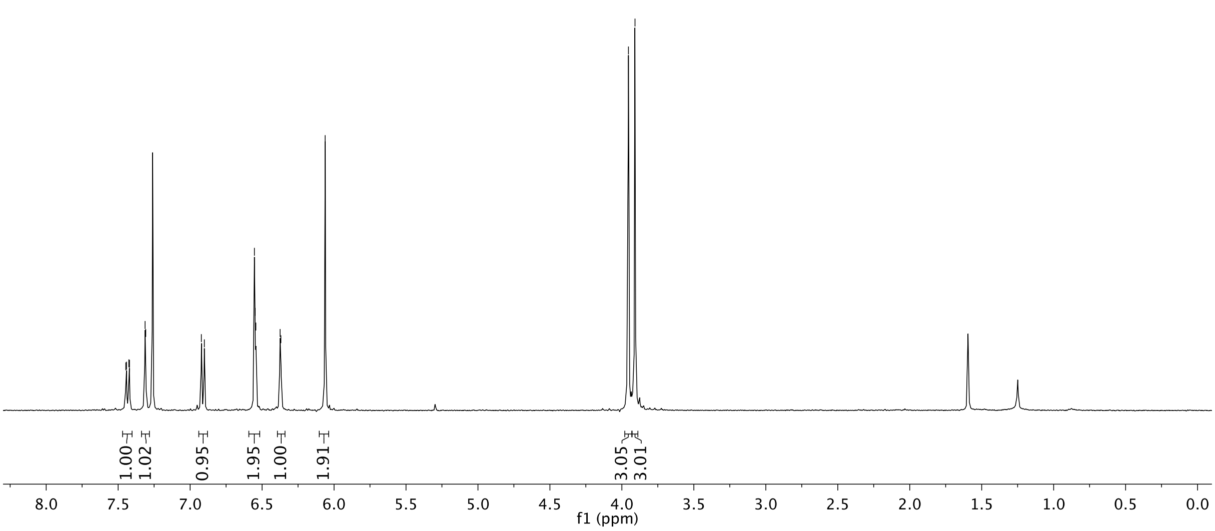
7.45
7.44
7.43
7.42
7.31
7.31
6.92
6.90
6.55
6.55
6.54
6.37
6.37
6.06
3.95
3.91
1.00
1.02
0.95
1.95
1.00
1.91
3.05
3.01
f1 (ppm)

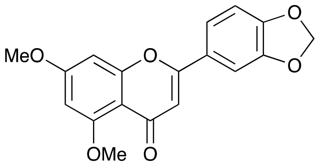
MeO
O
O
O
OMe
O

18h

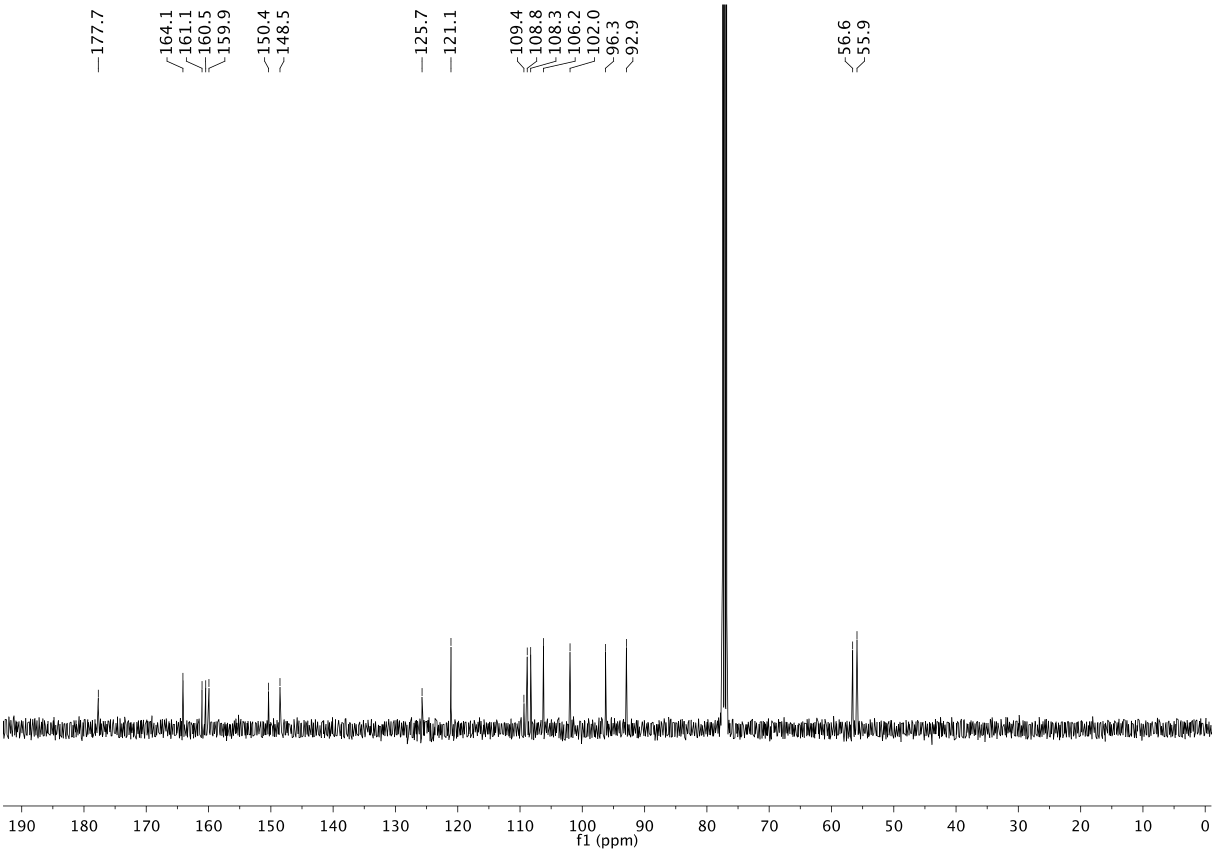
177.7
164.1
161.1
160.5
159.9
150.4
148.5
125.7
121.1
109.4
108.8
108.3
106.2
102.0
96.3
92.9
56.6
55.9
f1 (ppm)

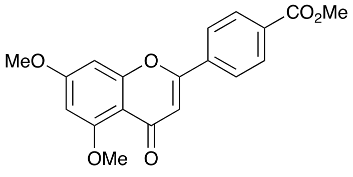
MeO
O
CO2Me
OMe
O

18i

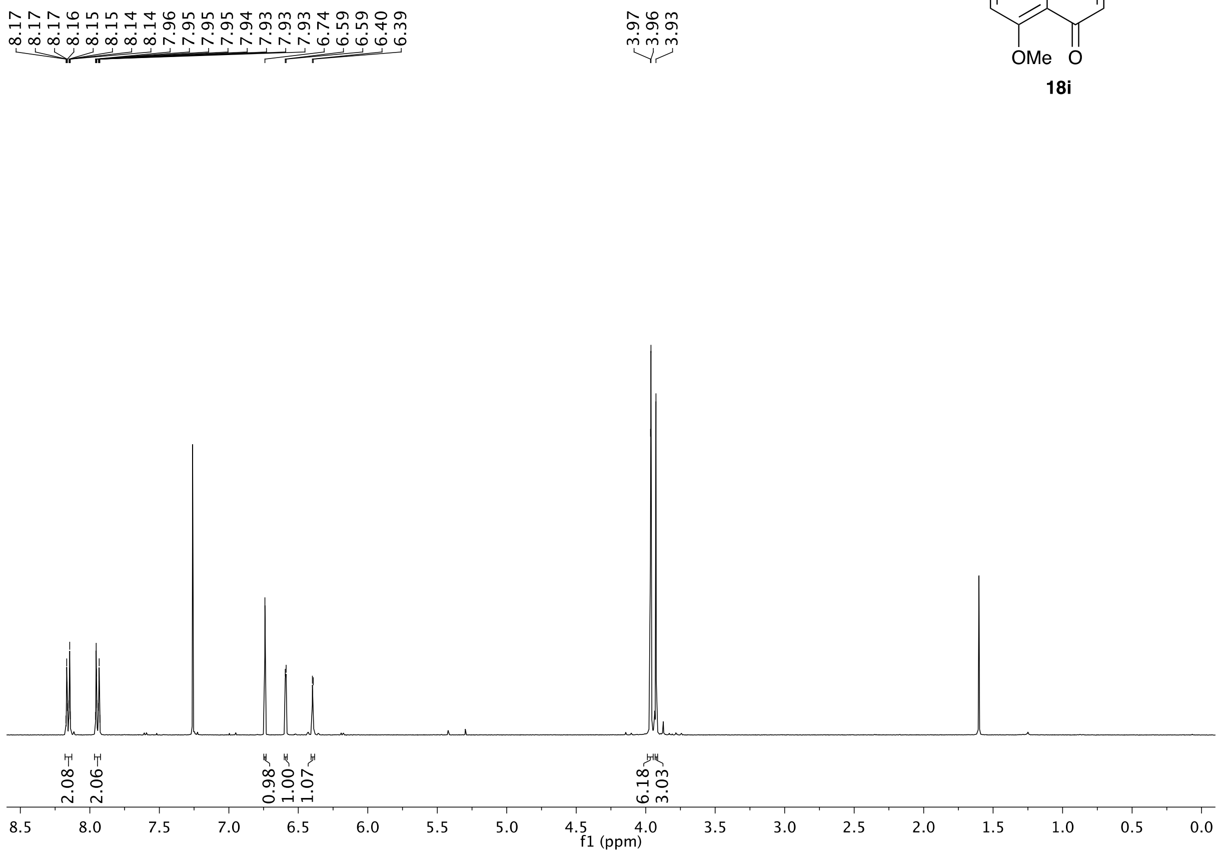
8.17
8.17
8.17
8.16
8.15
8.15
8.14
8.14
7.96
7.95
7.95
7.95
7.94
7.93
7.93
7.93
7.93
6.74
6.59
6.59
6.40
6.39
3.97
3.96
3.93
2.08
2.06
0.98
1.00
1.07
6.18
3.03
8.5 8.0 7.5 7.0 6.5 6.0 5.5 5.0 4.5 4.0 3.5 3.0 2.5 2.0 1.5 1.0 0.5 0.0
f1 (ppm)

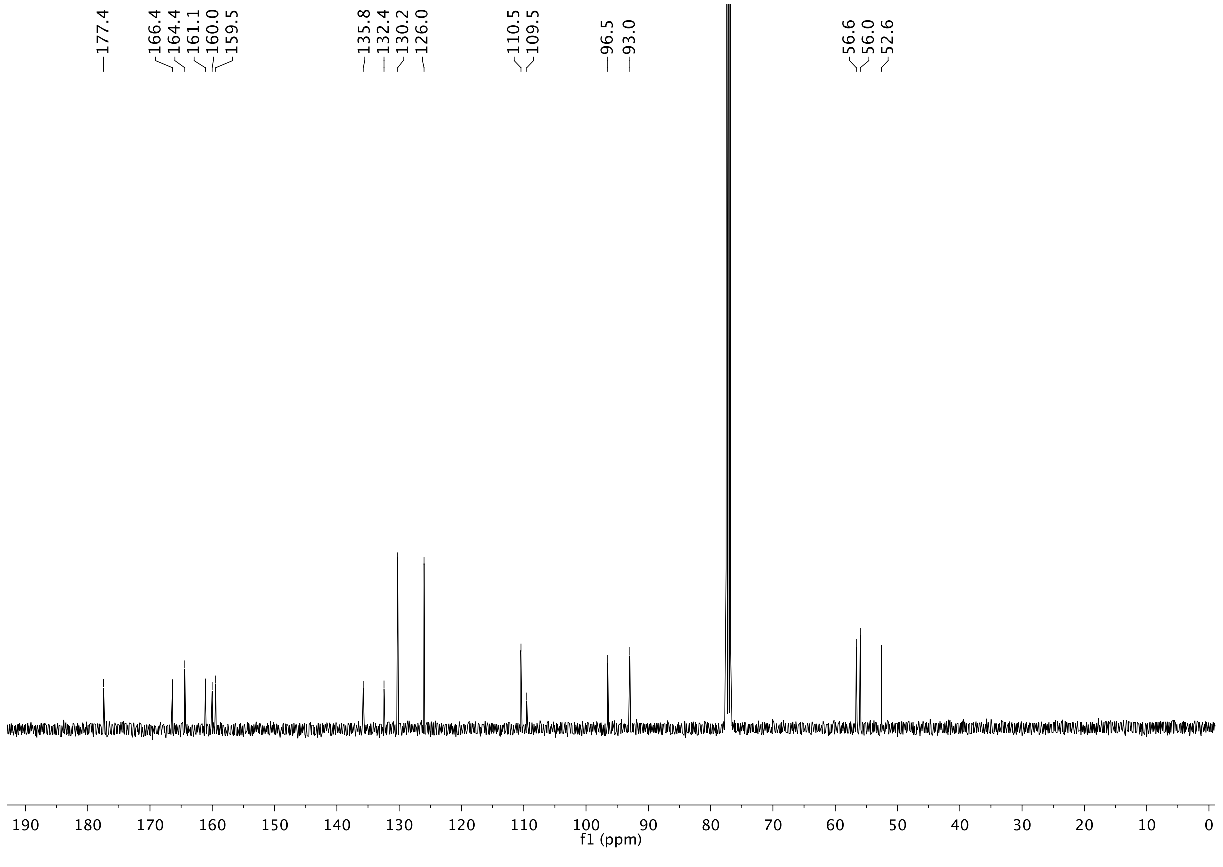
177.4
166.4
164.4
161.1
160.0
159.5
135.8
132.4
130.2
126.0
110.5
109.5
96.5
93.0
56.6
56.0
52.6
190 180 170 160 150 140 130 120 110 100 90 80 70 60 50 40 30 20 10 0
f1 (ppm)

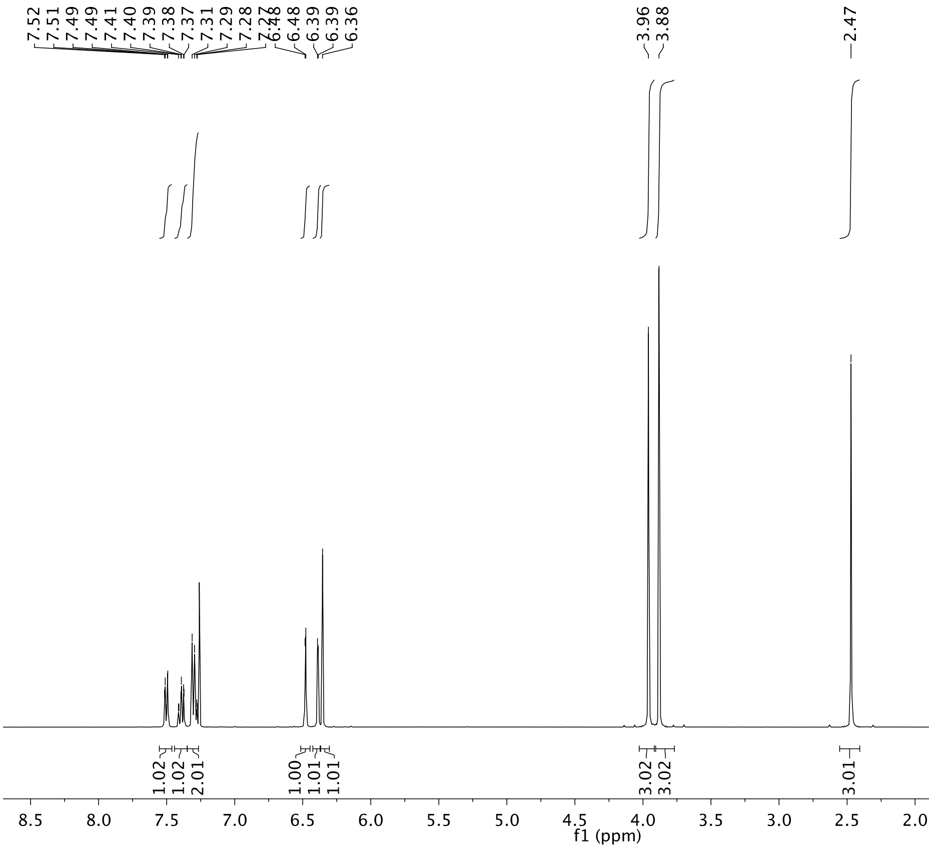
7.52
7.51
7.49
7.49
7.41
7.40
7.39
7.38
7.37
7.31
7.29
7.28
7.27
6.48
6.48
6.39
6.39
6.36
3.96
3.88
2.47
1.02
1.02
2.01
1.00
1.01
1.01
3.02
3.02
3.01
8.5 8.0 7.5 7.0 6.5 6.0 5.5 5.0 4.5 4.0 3.5 3.0 2.5 2.0 1.5 1.0 0.5 0.0
f1 (ppm)

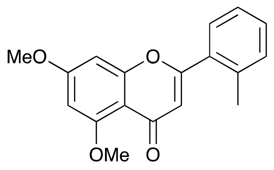
MeO
O
OMe
O

18j

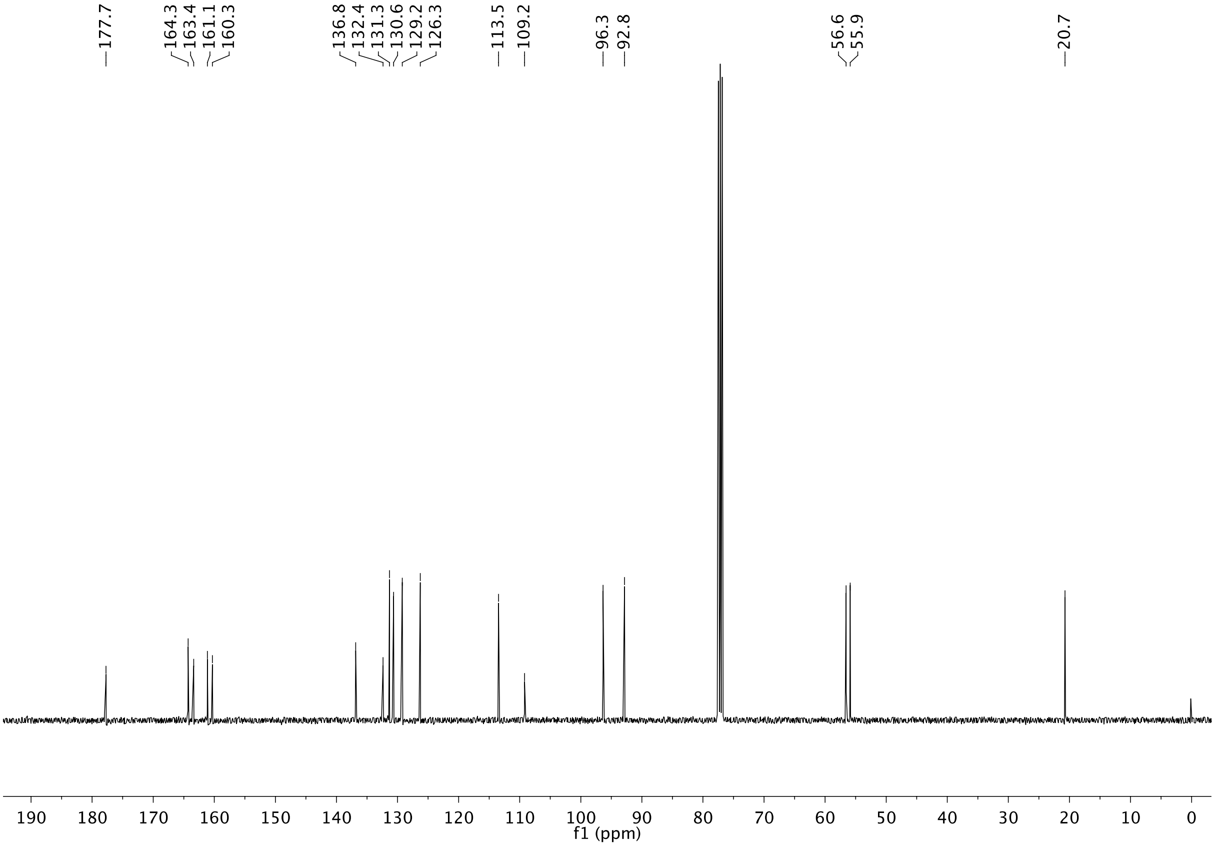
177.7
164.3
163.4
161.1
160.3
136.8
132.4
131.3
130.6
129.2
126.3
113.5
109.2
96.3
92.8
56.6
55.9
20.7
190 180 170 160 150 140 130 120 110 100 90 80 70 60 50 40 30 20 10 0
f1 (ppm)

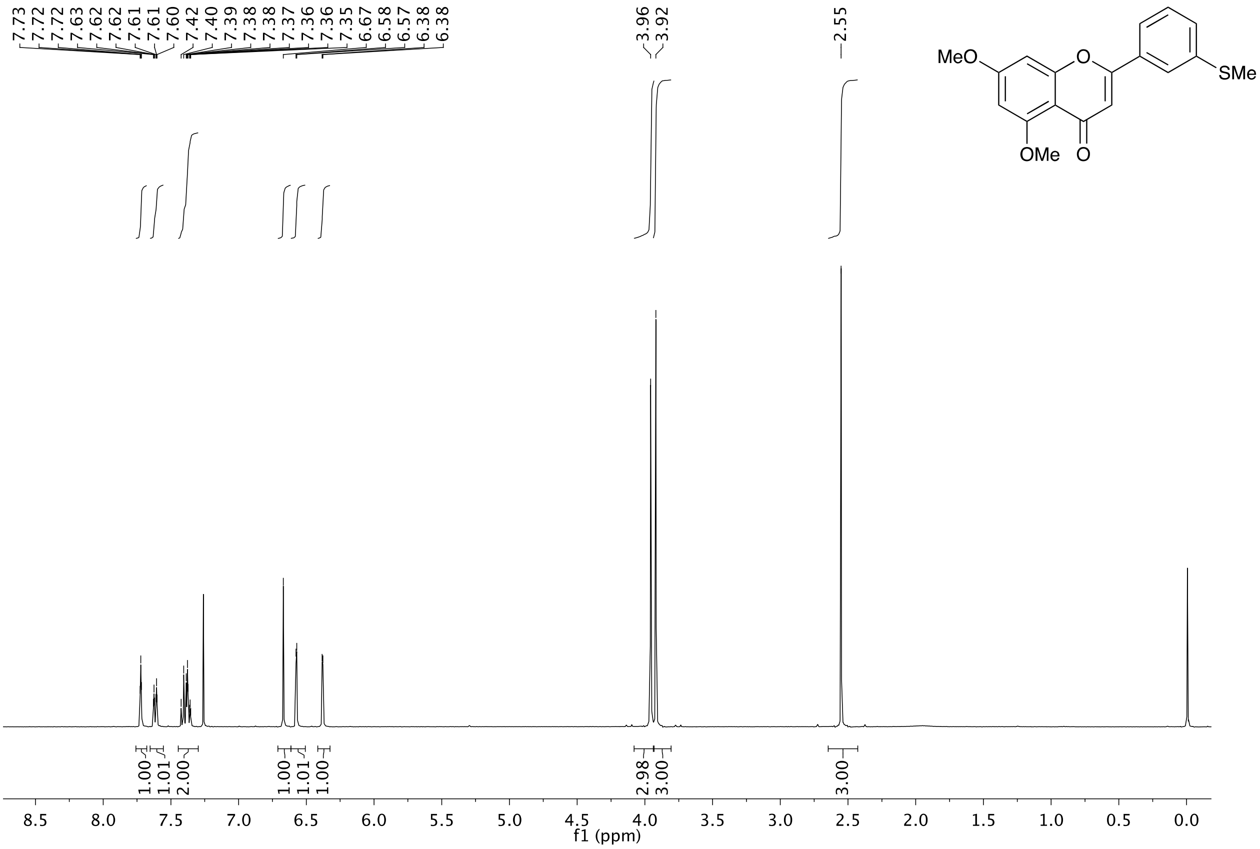
7.73
7.72
7.72
7.63
7.62
7.62
7.61
7.61
7.60
7.42
7.40
7.39
7.38
7.38
7.37
7.36
7.36
7.35
6.67
6.58
6.57
6.38
6.38
3.96
3.92
2.55
1.00
1.01
2.00
1.00
1.01
1.00
2.98
3.00
3.00
8.5 8.0 7.5 7.0 6.5 6.0 5.5 5.0 4.5 4.0 3.5 3.0 2.5 2.0 1.5 1.0 0.5 0.0
f1 (ppm)
MeO
O
SMe
OMe
O

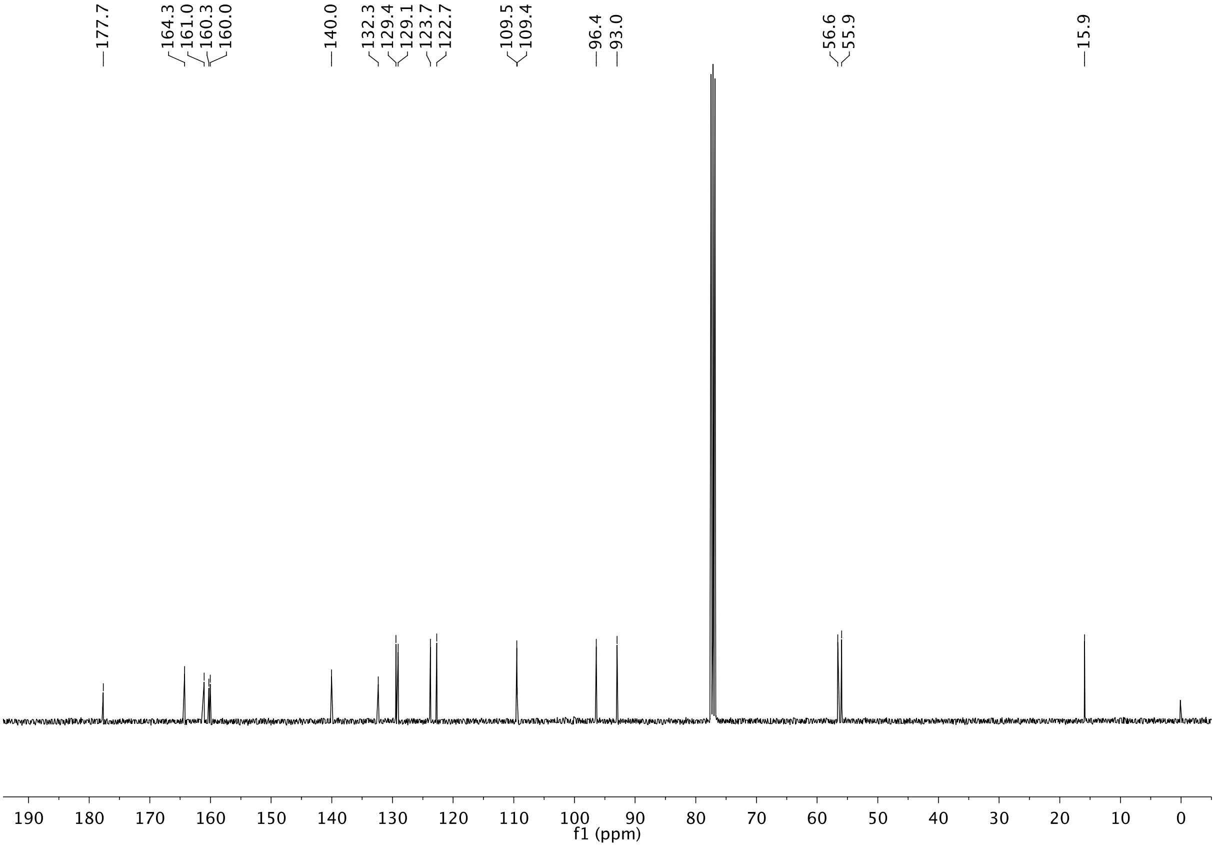
177.7
164.3
161.0
160.3
160.0
140.0
132.3
129.4
129.1
123.7
122.7
109.5
109.4
96.4
93.0
56.6
55.9
15.9
190 180 170 160 150 140 130 120 110 100 90 80 70 60 50 40 30 20 10 0
f1 (ppm)

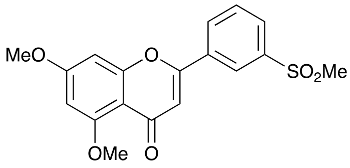
MeO
O
SO2Me
OMe
O

18k

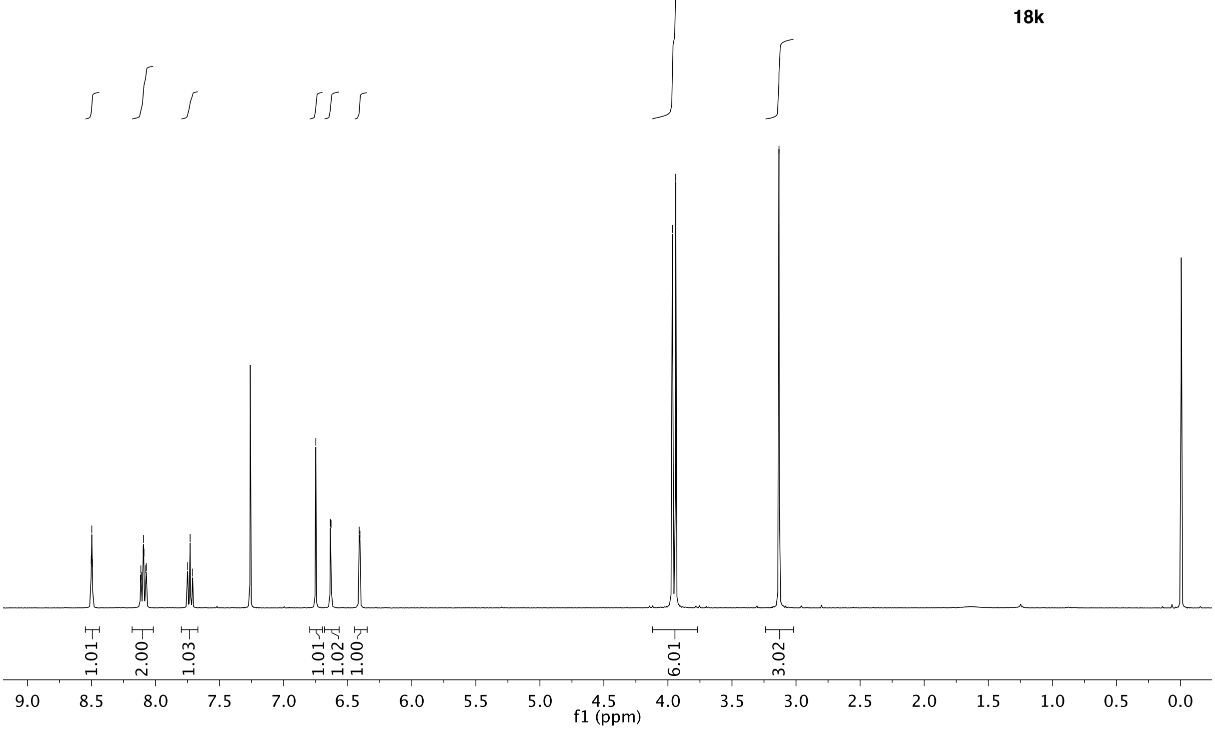
8.50
8.50
8.49
8.12
8.11
8.11
8.10
8.09
8.09
8.08
8.07
7.75
7.73
7.71
6.75
6.64
6.63
6.41
6.40
3.97
3.94
3.13
1.01
2.00
1.03
1.01
1.02
1.00
6.01
3.02
9.0 8.5 8.0 7.5 7.0 6.5 6.0 5.5 5.0 4.5 4.0 3.5 3.0 2.5 2.0 1.5 1.0 0.5 0.0
f1 (ppm)

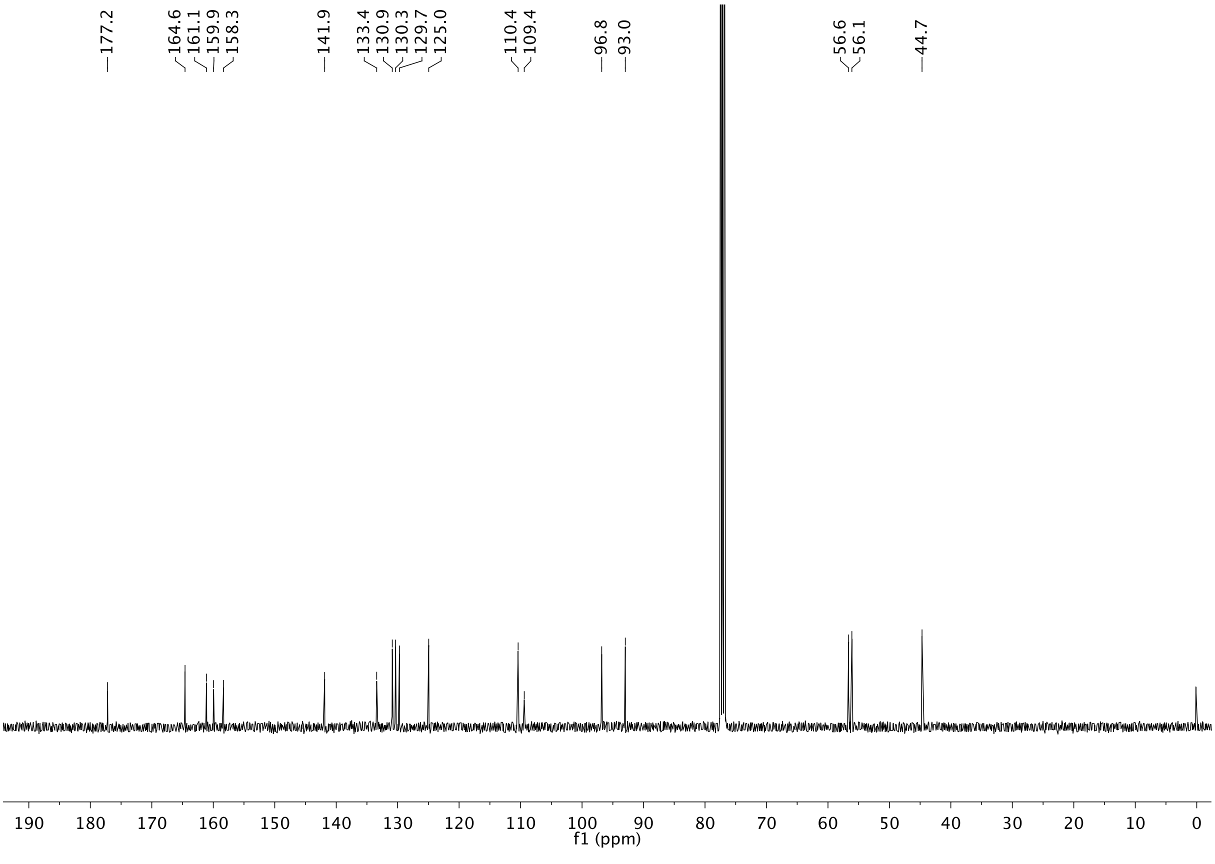
177.2
164.6
161.1
159.9
158.3
141.9
133.4
130.9
130.3
129.7
125.0
110.4
109.4
96.8
93.0
56.6
56.1
44.7
190 180 170 160 150 140 130 120 110 100 90 80 70 60 50 40 30 20 10 0
f1 (ppm)

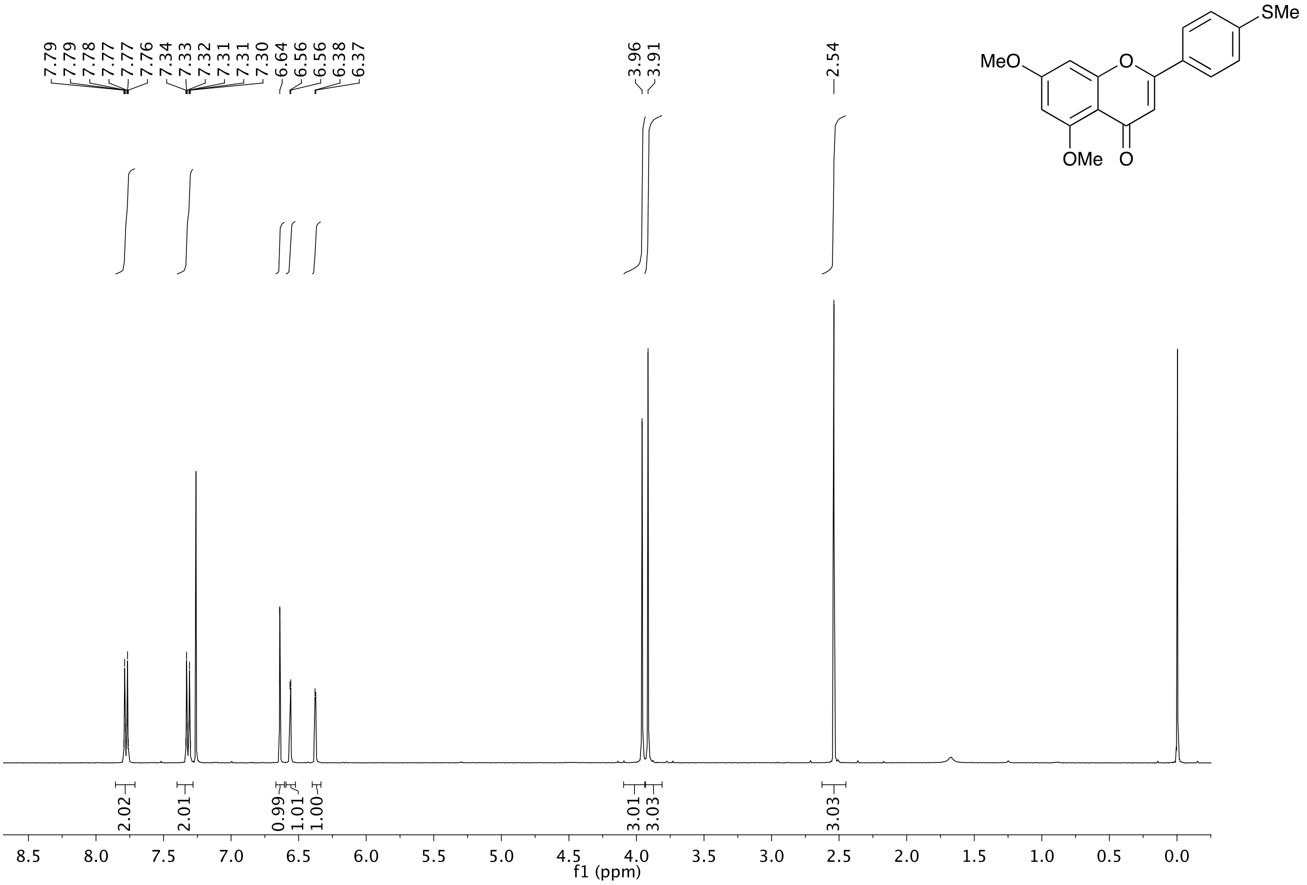
7.79
7.79
7.78
7.77
7.77
7.76
7.34
7.33
7.32
7.31
7.31
7.30
6.64
6.56
6.56
6.38
6.37
3.96
3.91
2.54
2.02
2.01
0.99
1.01
1.00
3.01
3.03
3.03
f1 (ppm)

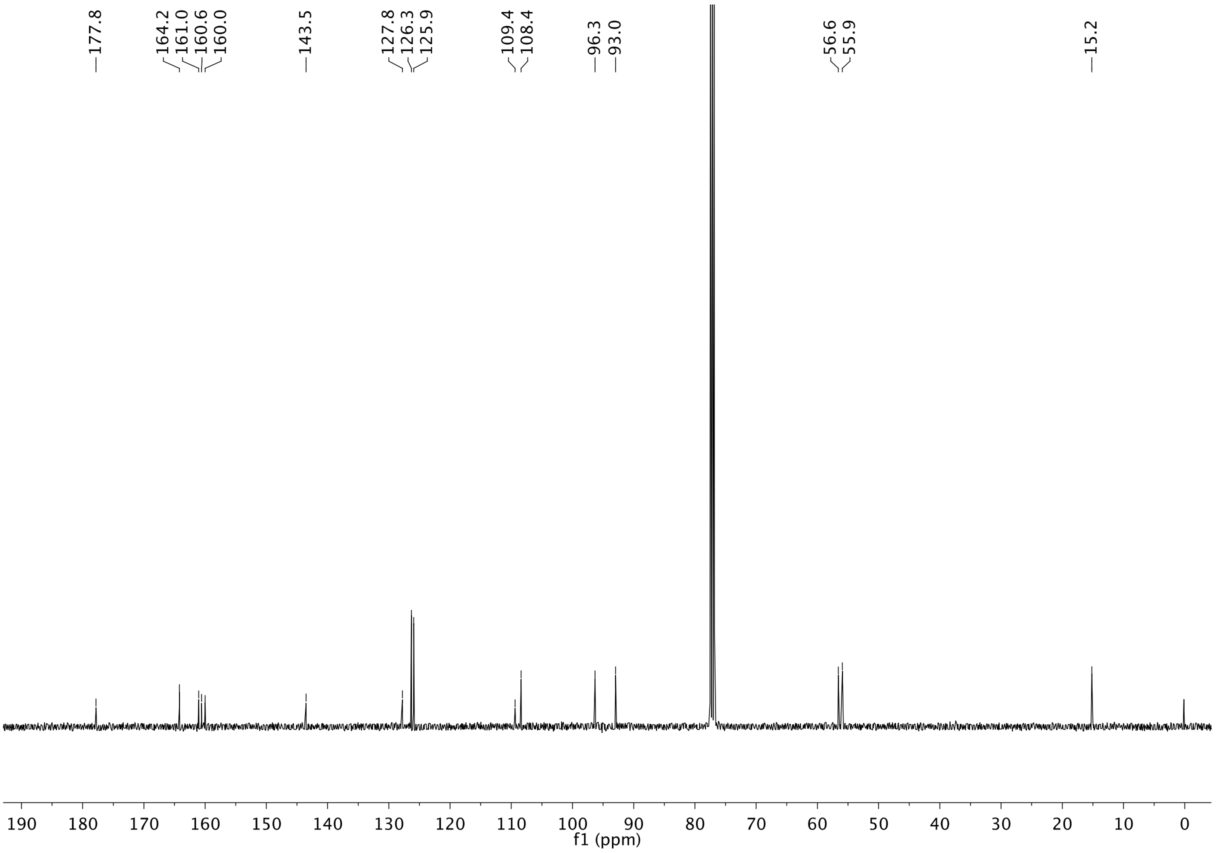
177.8
164.2
161.0
160.6
160.0
143.5
127.8
126.3
125.9
109.4
108.4
96.3
93.0
56.6
55.9
15.2
f1 (ppm)

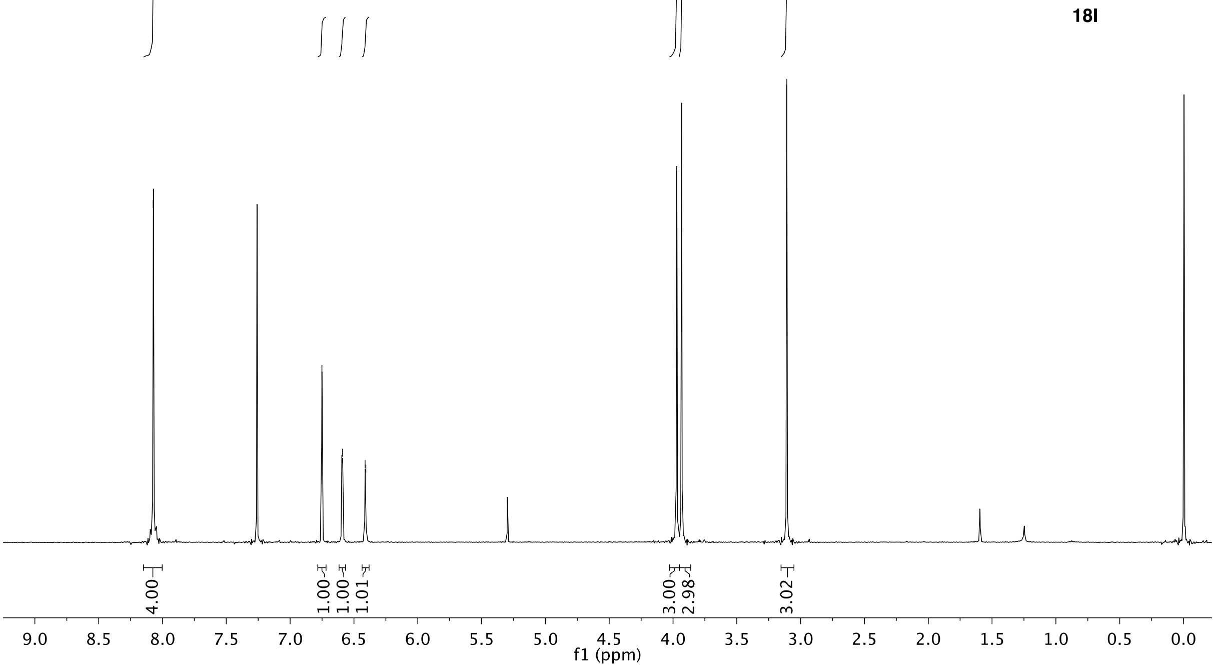

8.07
8.07
6.75
6.59
6.59
6.41
6.41
3.97
3.93
3.11
4.00
1.00
1.00
1.01
3.00
2.98
3.02
f1 (ppm)

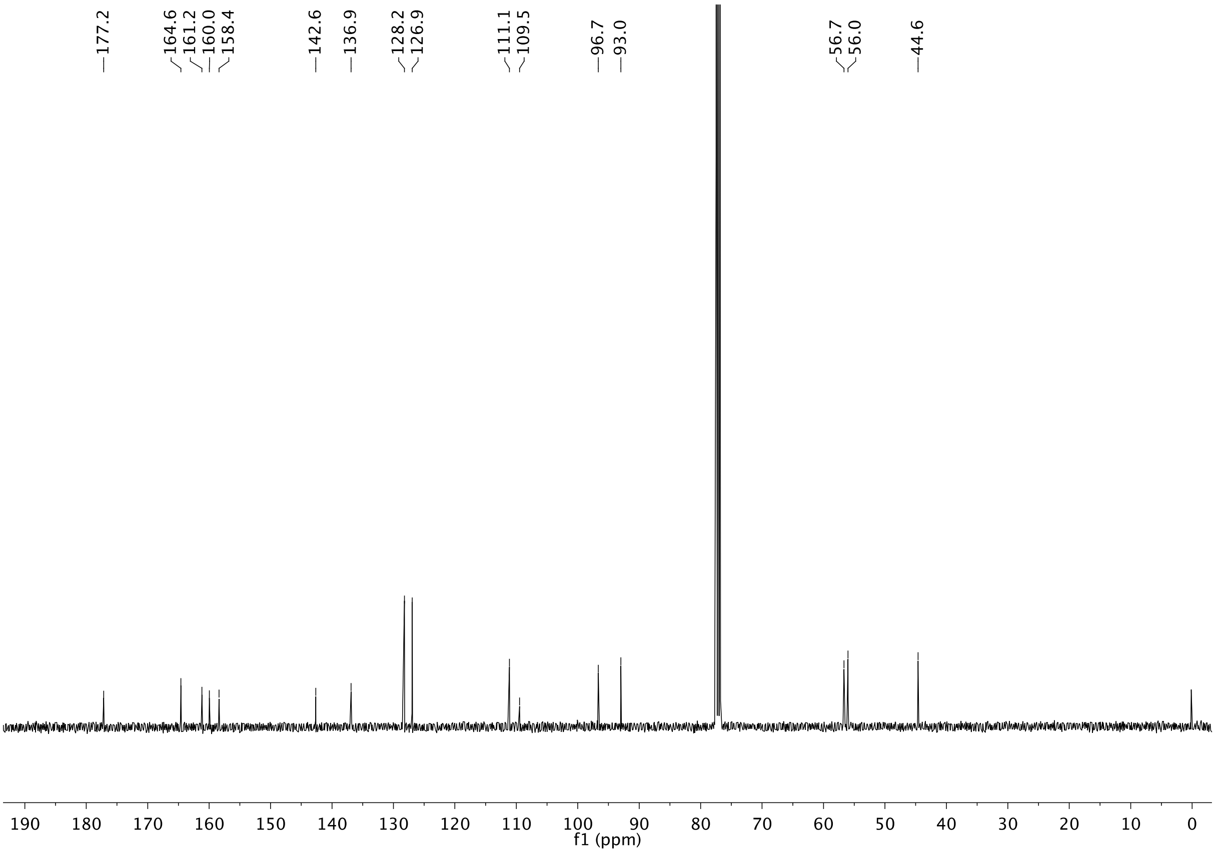

177.2
164.6
161.2
160.0
158.4
142.6
136.9
128.2
126.9
111.1
109.5
96.7
93.0
56.7
56.0
44.6
f1 (ppm)

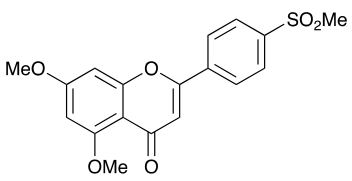

SO2Me
MeO
O
OMe
O

18l

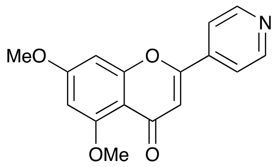

MeO
O
N
OMe
O

18m

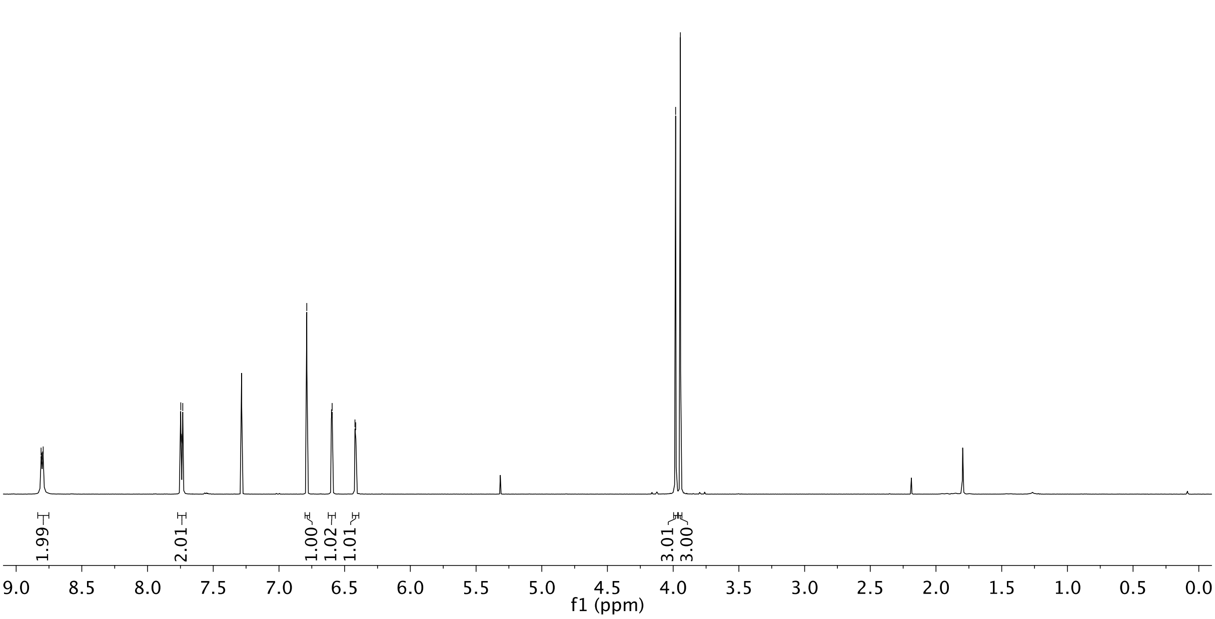

8.81
8.81
8.80
8.79
7.75
7.74
7.74
7.73
6.79
6.60
6.59
6.42
6.42
3.98
3.95
1.99
2.01
1.00
1.02
1.01
3.01
3.00
9.0 8.5 8.0 7.5 7.0 6.5 6.0 5.5 5.0 4.5 4.0 3.5 3.0 2.5 2.0 1.5 1.0 0.5 0.0
f1 (ppm)

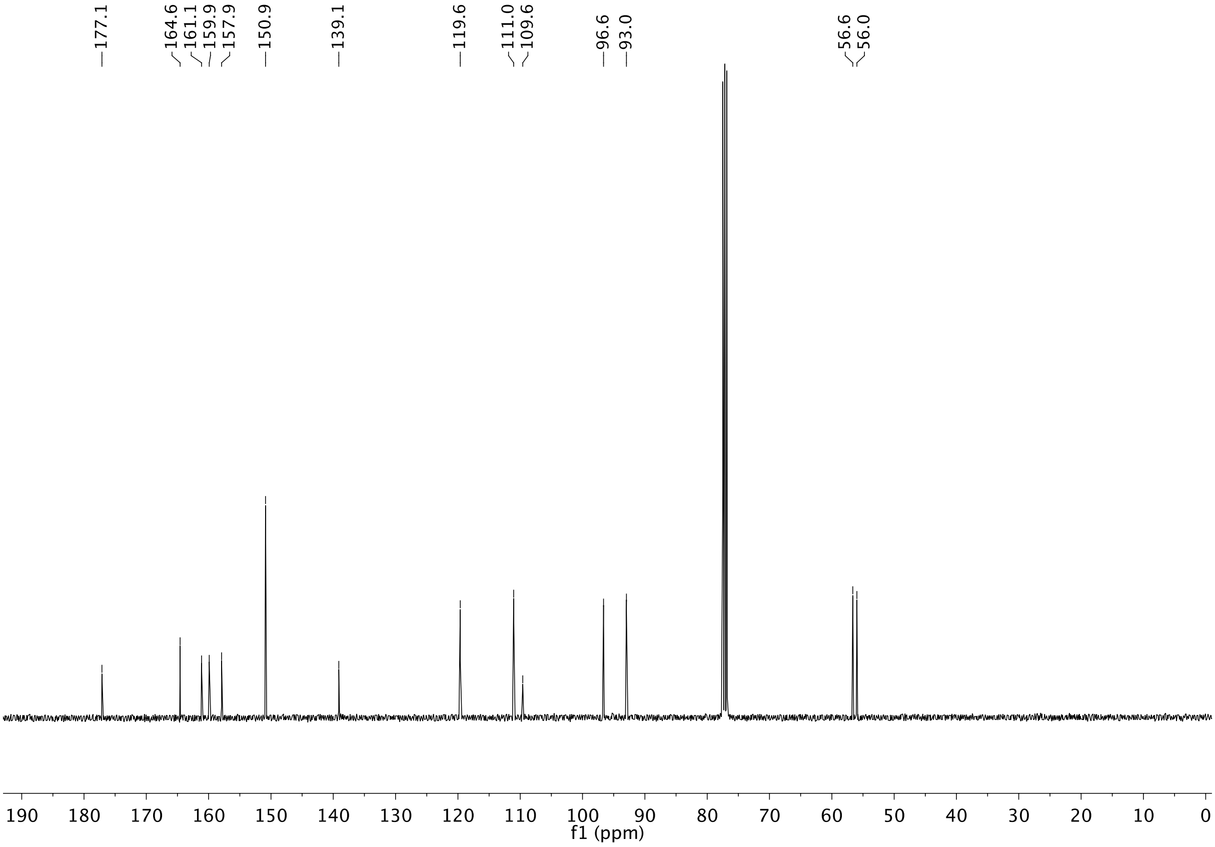

177.1
164.6
161.1
159.9
157.9
150.9
139.1
119.6
111.0
109.6
96.6
93.0
56.6
56.0
190 180 170 160 150 140 130 120 110 100 90 80 70 60 50 40 30 20 10 0
f1 (ppm)

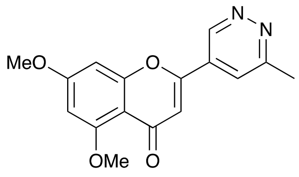
MeO
O
N
N
OMe
O

18n

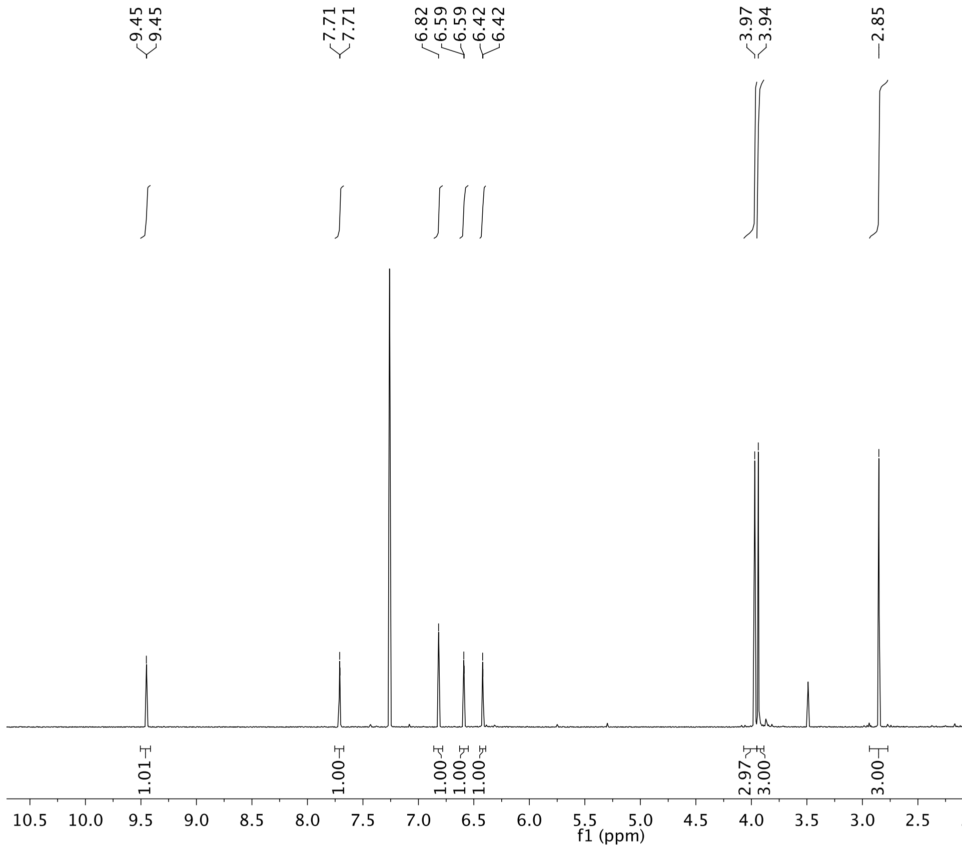
9.45
9.45
7.71
7.71
6.82
6.59
6.59
6.42
6.42
3.97
3.94
2.85
1.01
1.00
1.00
1.00
1.00
2.97
3.00
3.00
10.5 10.0 9.5 9.0 8.5 8.0 7.5 7.0 6.5 6.0 5.5 5.0 4.5 4.0 3.5 3.0 2.5 2.0 1.5 1.0 0.5 0.0
f1 (ppm)

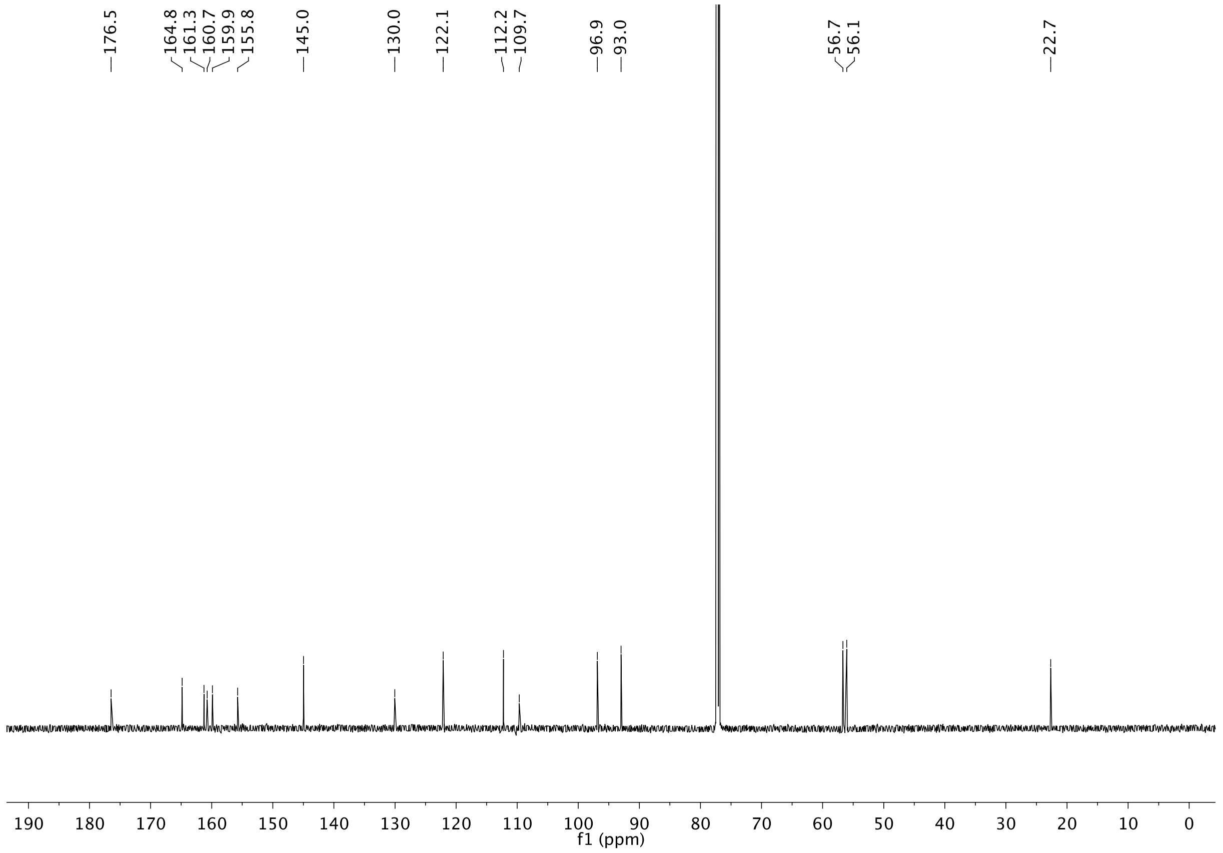
176.5
164.8
161.3
160.7
159.9
155.8
145.0
130.0
122.1
112.2
109.7
96.9
93.0
56.7
56.1
22.7
190 180 170 160 150 140 130 120 110 100 90 80 70 60 50 40 30 20 10 0
f1 (ppm)

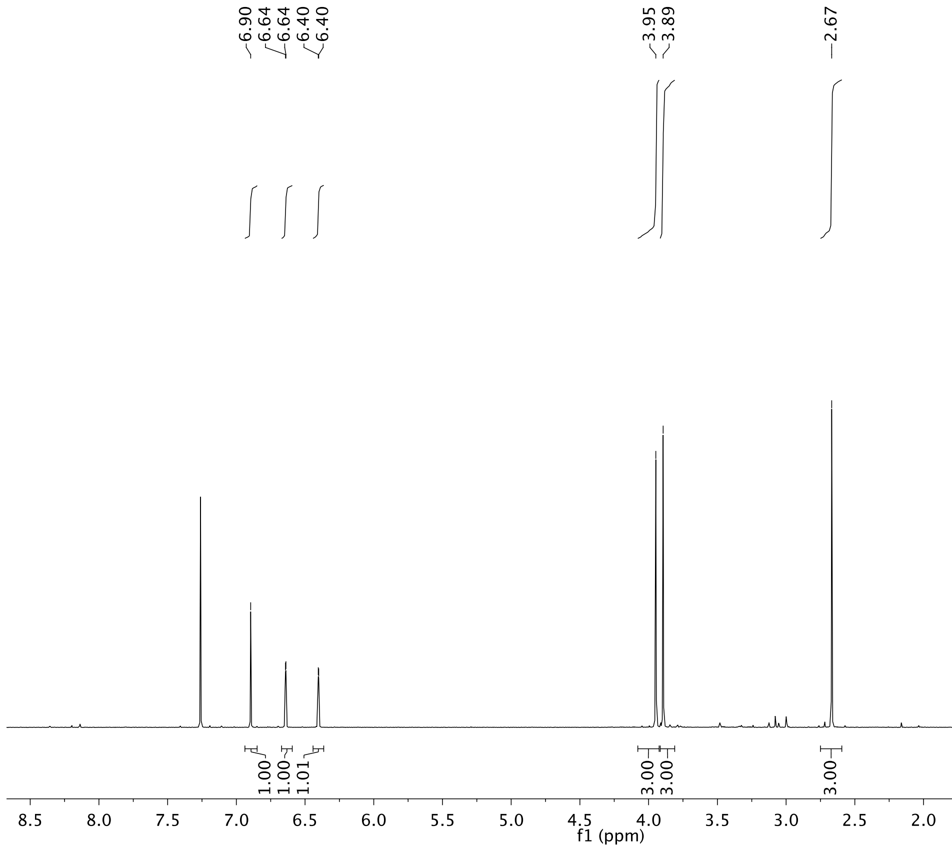
6.90
6.64
6.64
6.40
6.40
3.95
3.89
2.67
1.00
1.00
1.01
3.00
3.00
3.00
8.5 8.0 7.5 7.0 6.5 6.0 5.5 5.0 4.5 4.0 3.5 3.0 2.5 2.0 1.5 1.0 0.5 0.0
f1 (ppm)

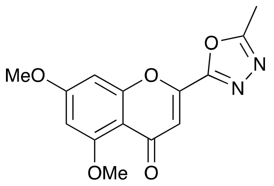
MeO
O
O
N
N
OMe
O

19

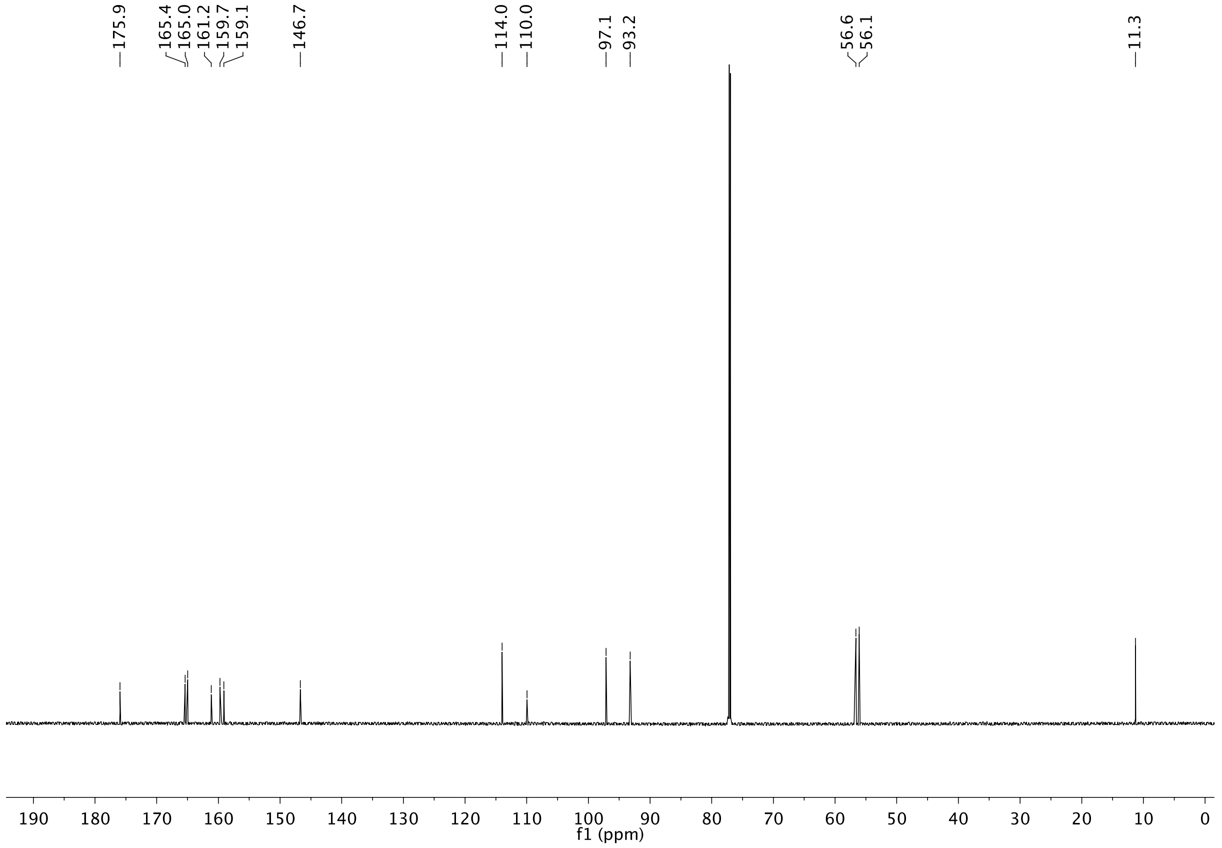
175.9
165.4
165.0
161.2
159.7
159.1
146.7
114.0
110.0
97.1
93.2
56.6
56.1
11.3
190 180 170 160 150 140 130 120 110 100 90 80 70 60 50 40 30 20 10 0
f1 (ppm)

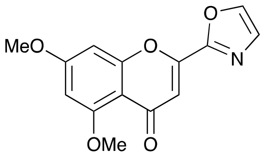
MeO
O
O
N
OMe
O

20

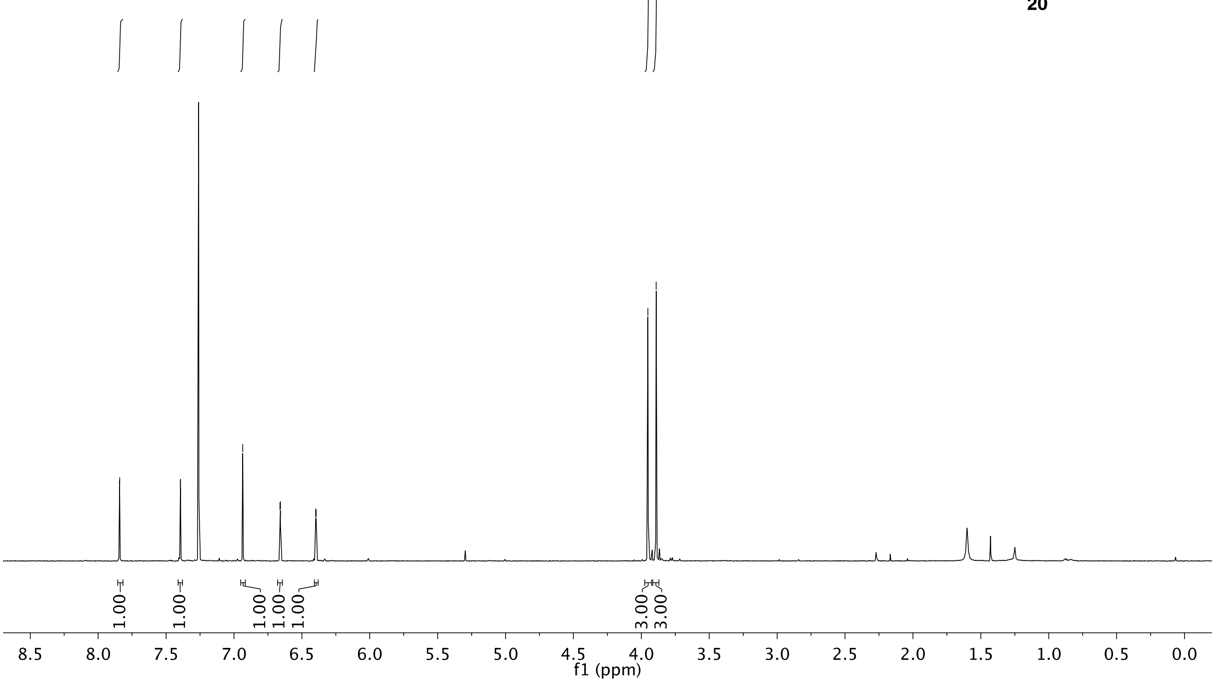
7.84
7.84
7.39
7.39
6.94
6.66
6.66
6.40
6.39
3.95
3.89
1.00
1.00
1.00
1.00
1.00
3.00
3.00
8.5 8.0 7.5 7.0 6.5 6.0 5.5 5.0 4.5 4.0 3.5 3.0 2.5 2.0 1.5 1.0 0.5 0.0
f1 (ppm)

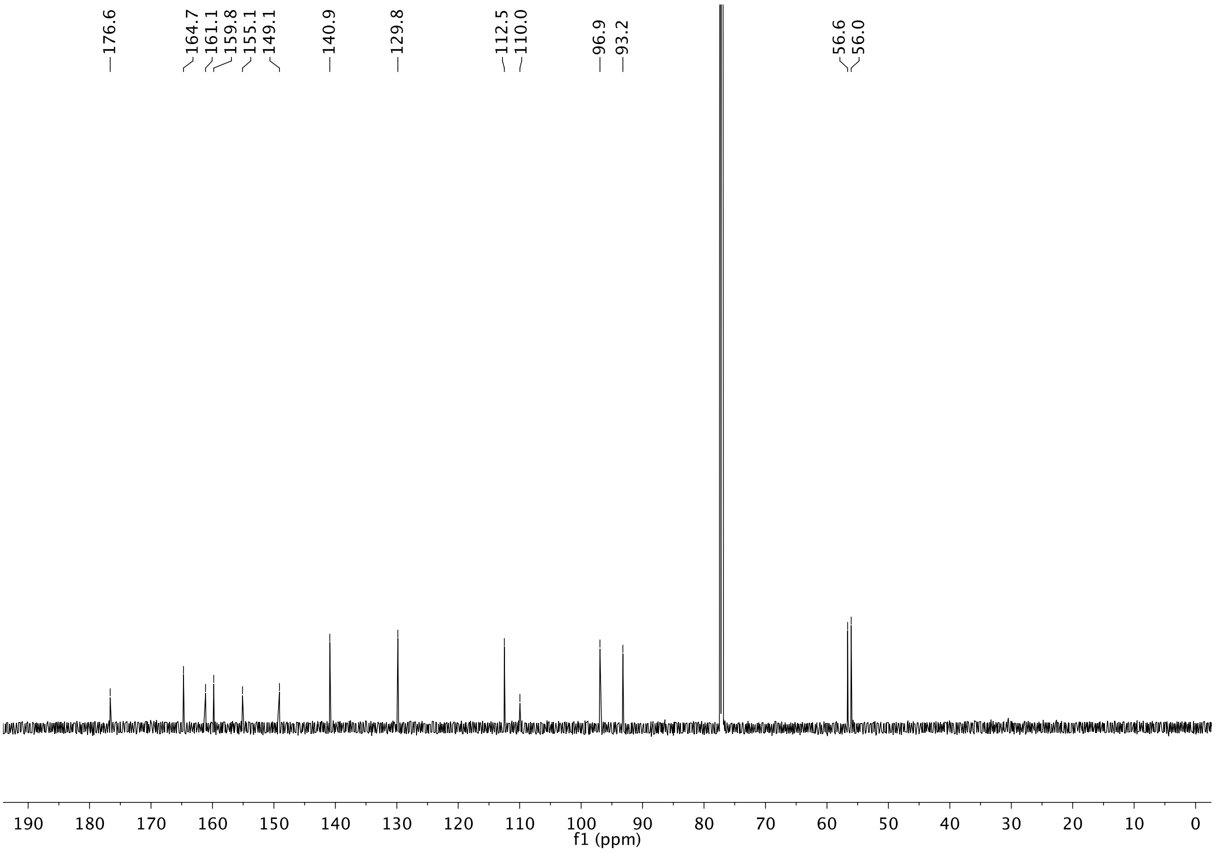
176.6
164.7
161.1
159.8
155.1
149.1
140.9
129.8
112.5
110.0
96.9
93.2
56.6
56.0
190 180 170 160 150 140 130 120 110 100 90 80 70 60 50 40 30 20 10 0
f1 (ppm)

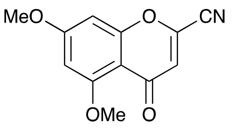
MeO
O
CN
OMe
O
21

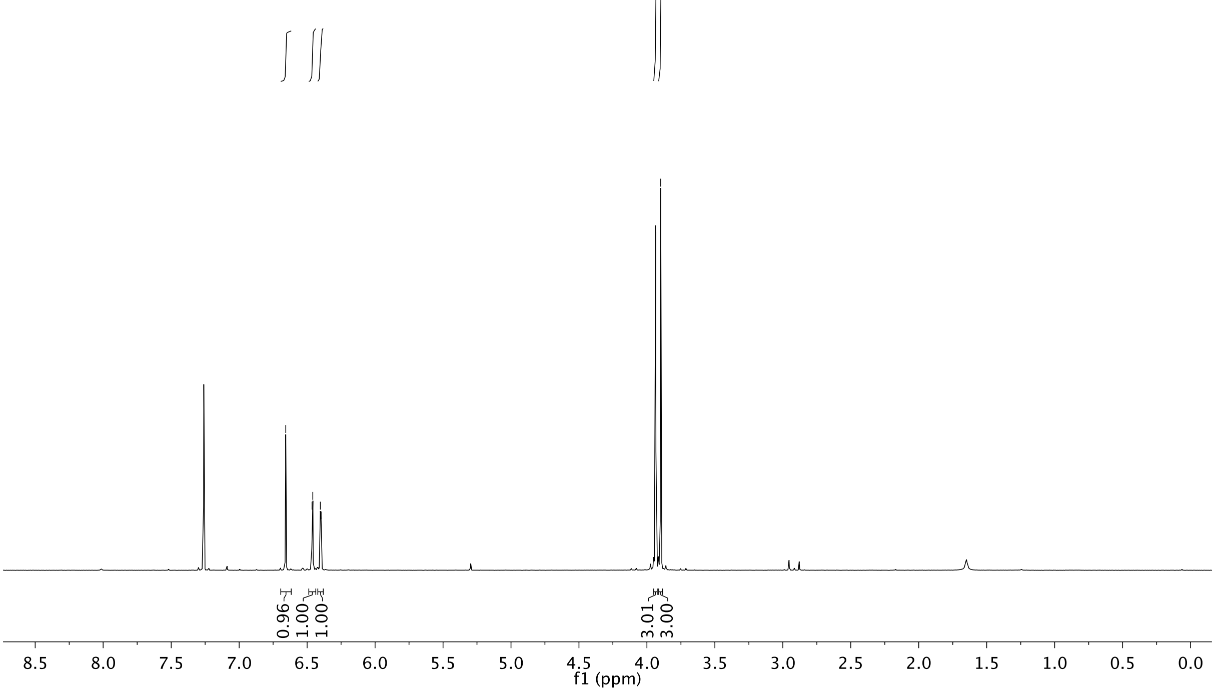
6.66
6.46
6.46
6.40
6.40
3.94
3.90
0.96
1.00
1.00
3.01
3.00
8.5 8.0 7.5 7.0 6.5 6.0 5.5 5.0 4.5 4.0 3.5 3.0 2.5 2.0 1.5 1.0 0.5 0.0
f1 (ppm)

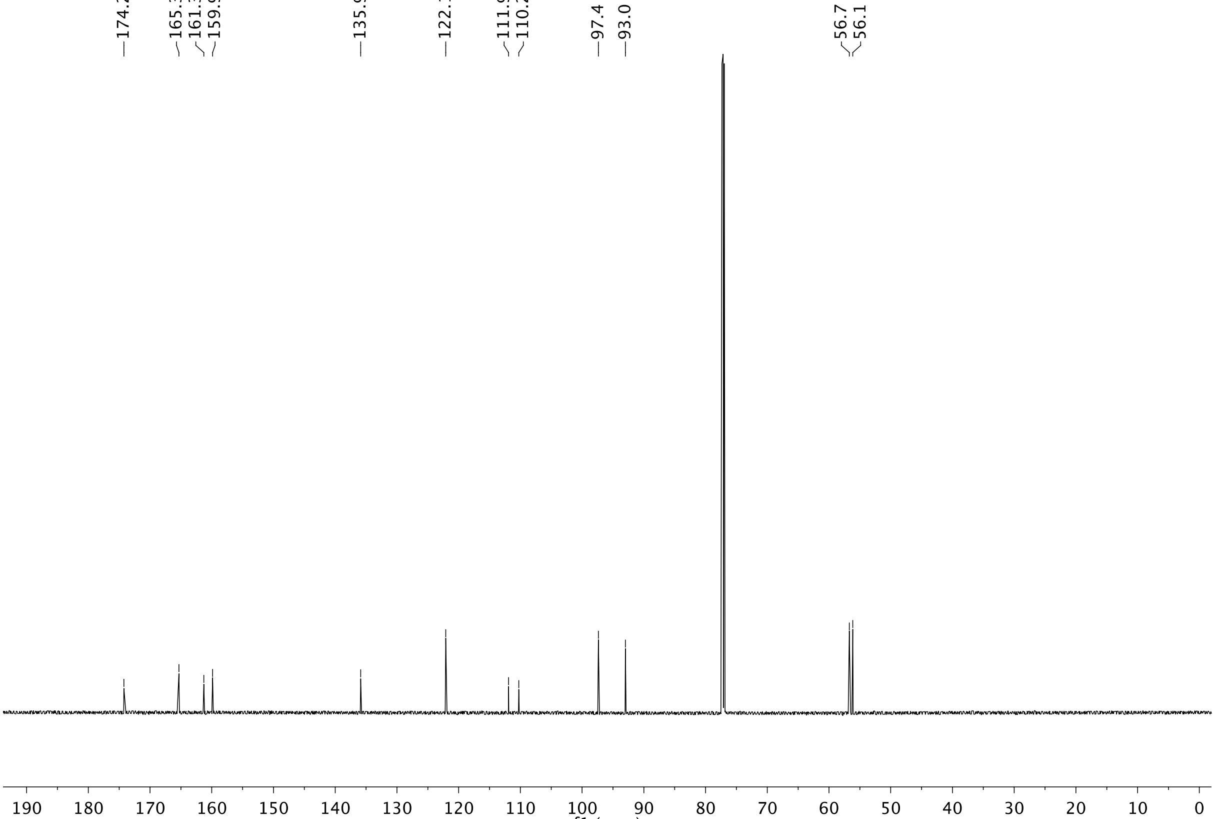
174.2
165.3
161.3
159.9
135.9
122.1
111.9
110.2
97.4
93.0
56.7
56.1
190 180 170 160 150 140 130 120 110 100 90 80 70 60 50 40 30 20 10 0
f1 (ppm)

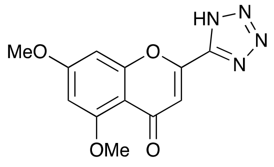

HN
N
N
N
MeO
O
OMe
O

22

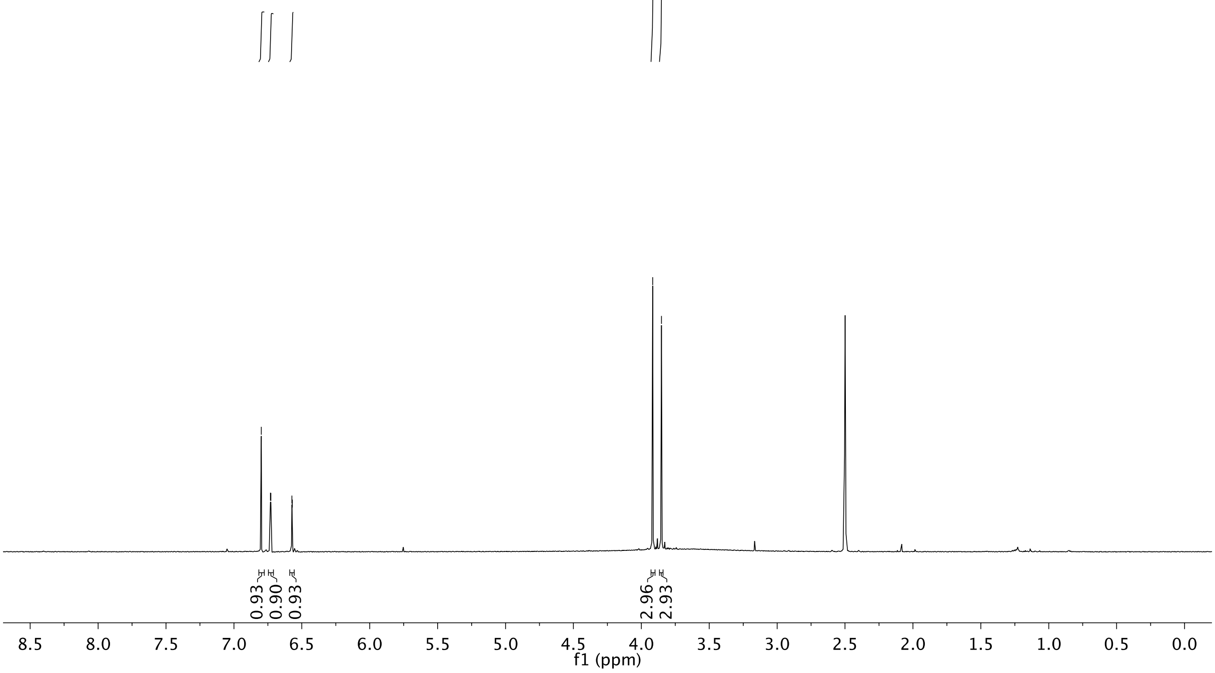

6.80
6.73
6.73
6.57
6.57
3.92
3.85
0.93
0.90
0.93
2.96
2.93
8.5 8.0 7.5 7.0 6.5 6.0 5.5 5.0 4.5 4.0 3.5 3.0 2.5 2.0 1.5 1.0 0.5 0.0
f1 (ppm)

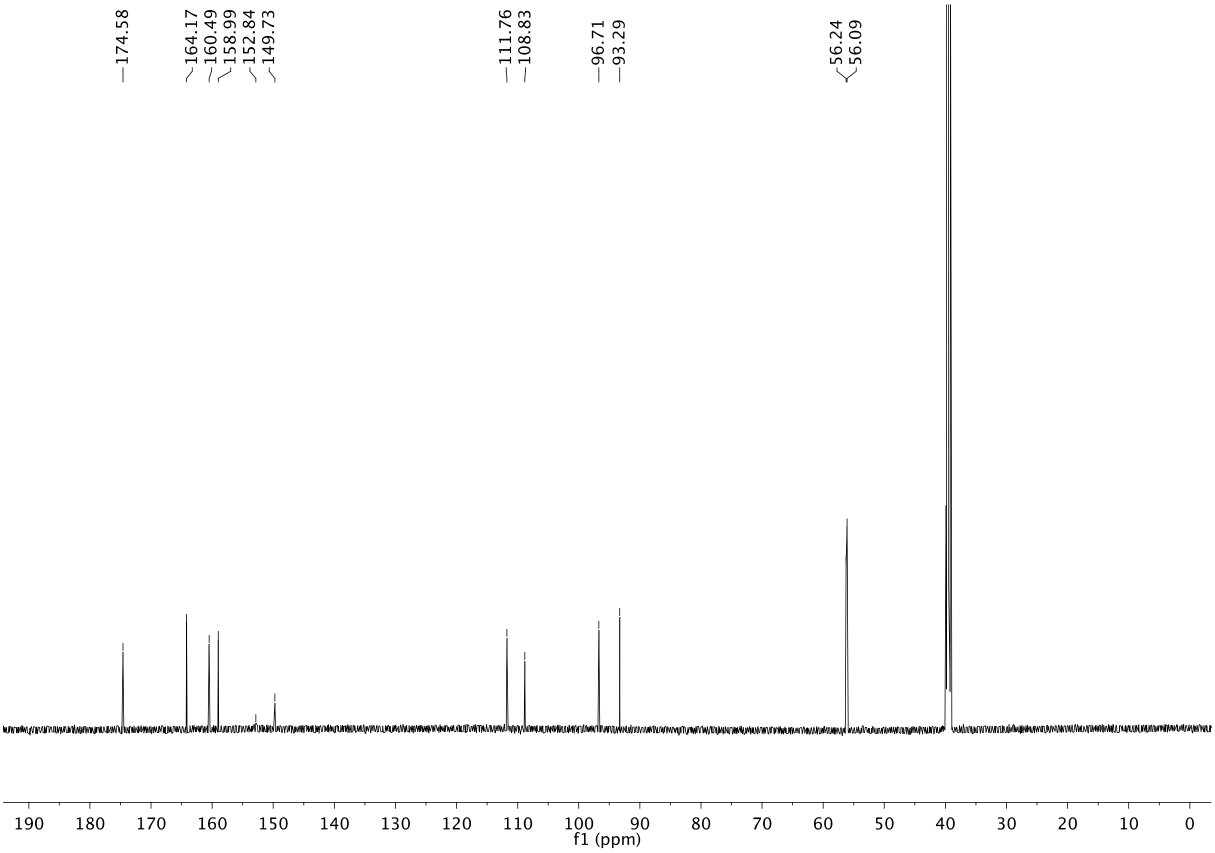

174.58
164.17
160.49
158.99
152.84
149.73
111.76
108.83
96.71
93.29
56.24
56.09
190 180 170 160 150 140 130 120 110 100 90 80 70 60 50 40 30 20 10 0
f1 (ppm)

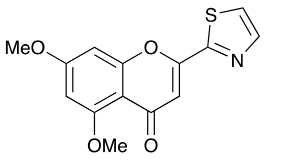
MeO
S
N
O
OMe
O

23

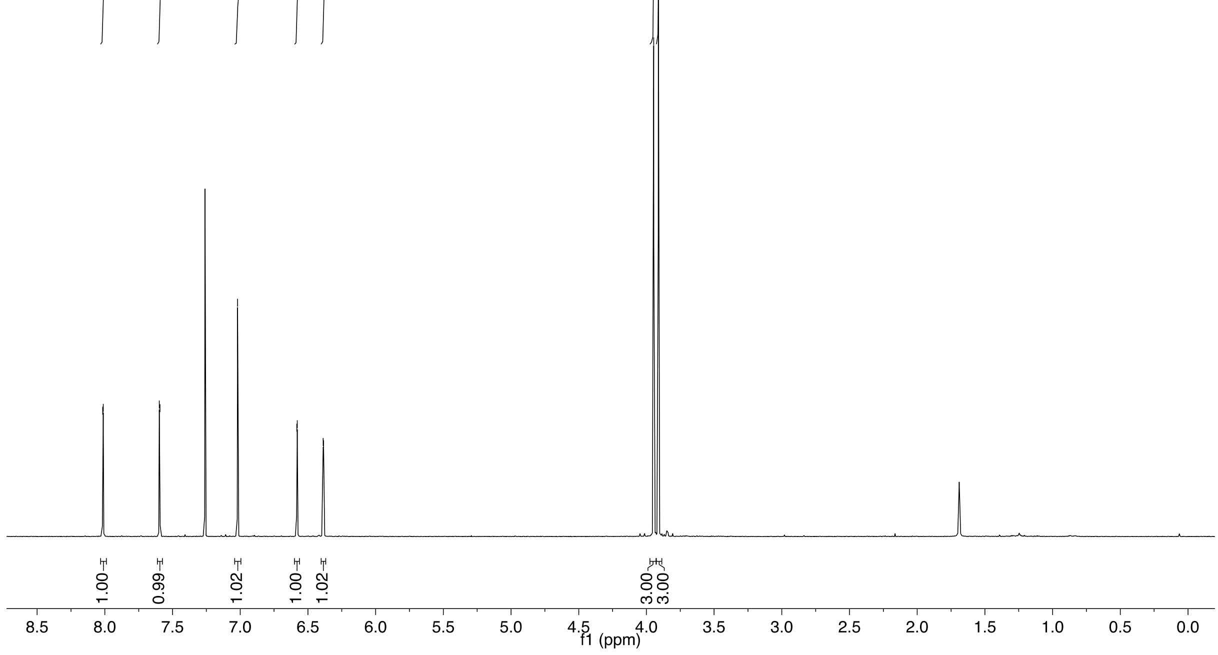
8.02
8.01
7.60
7.59
7.02
6.58
6.58
6.39
6.38
3.95
3.91
1.00
0.99
1.02
1.00
1.02
3.00
3.00
8.5 8.0 7.5 7.0 6.5 6.0 5.5 5.0 4.5 4.0 3.5 3.0 2.5 2.0 1.5 1.0 0.5 0.0
f1 (ppm)

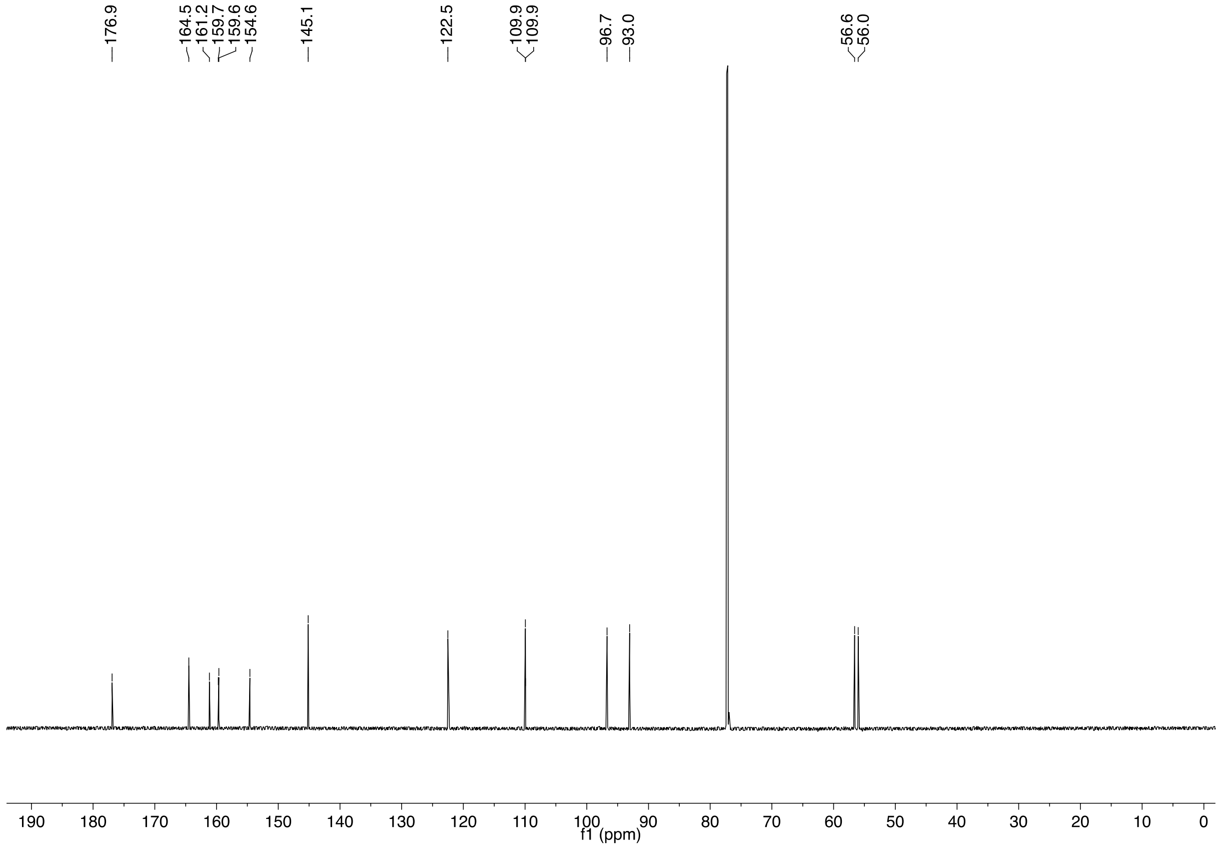
176.9
164.5
161.2
159.7
159.6
154.6
145.1
122.5
109.9
109.9
96.7
93.0
56.6
56.0
190 180 170 160 150 140 130 120 110 100 90 80 70 60 50 40 30 20 10 0
f1 (ppm)

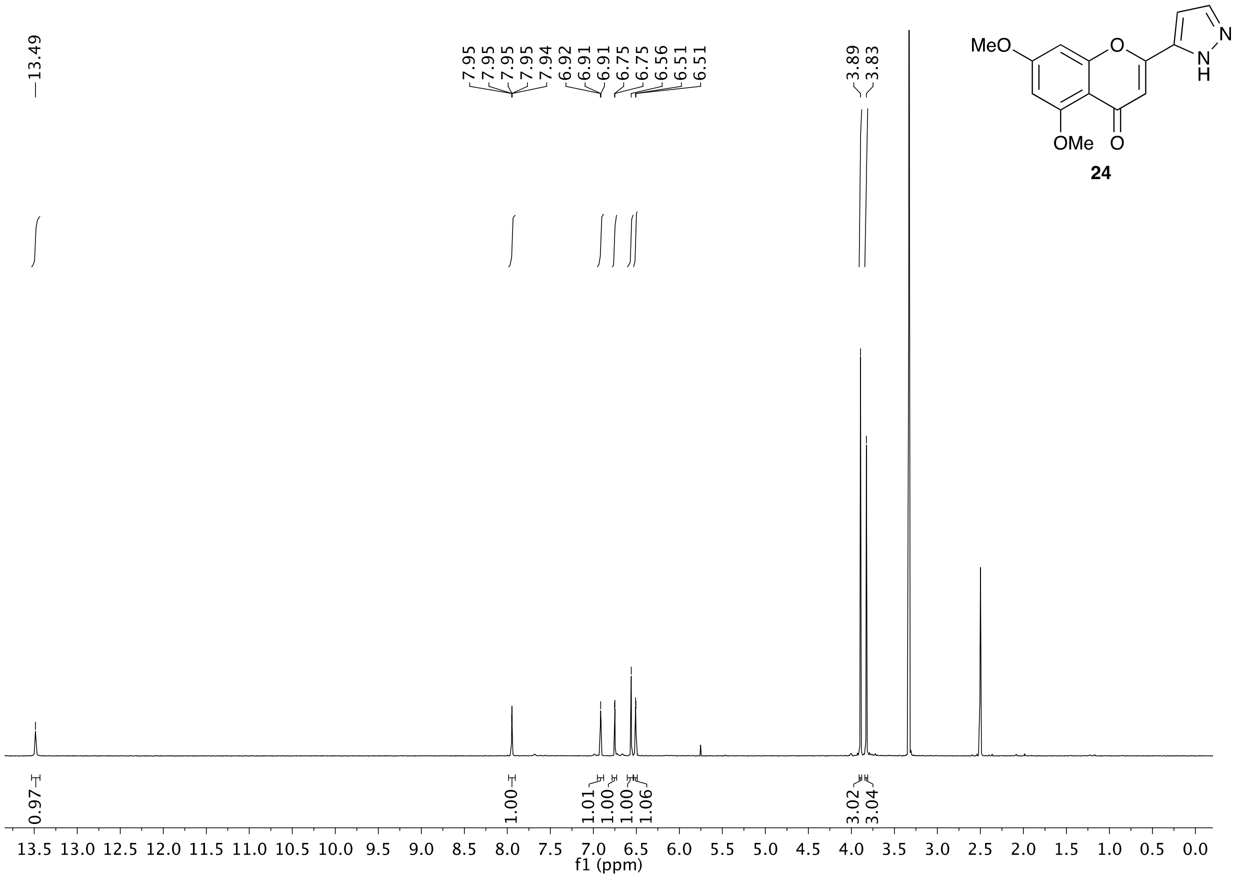
MeO
O
N
N
H
OMe
O
24
13.49
7.95
7.95
7.95
7.95
7.94
6.92
6.91
6.91
6.75
6.75
6.56
6.51
6.51
3.89
3.83
0.97
1.00
1.01
1.00
1.00
1.06
3.02
3.04
13.5 13.0 12.5 12.0 11.5 11.0 10.5 10.0 9.5 9.0 8.5 8.0 7.5 7.0 6.5 6.0 5.5 5.0 4.5 4.0 3.5 3.0 2.5 2.0 1.5 1.0 0.5 0.0
f1 (ppm)

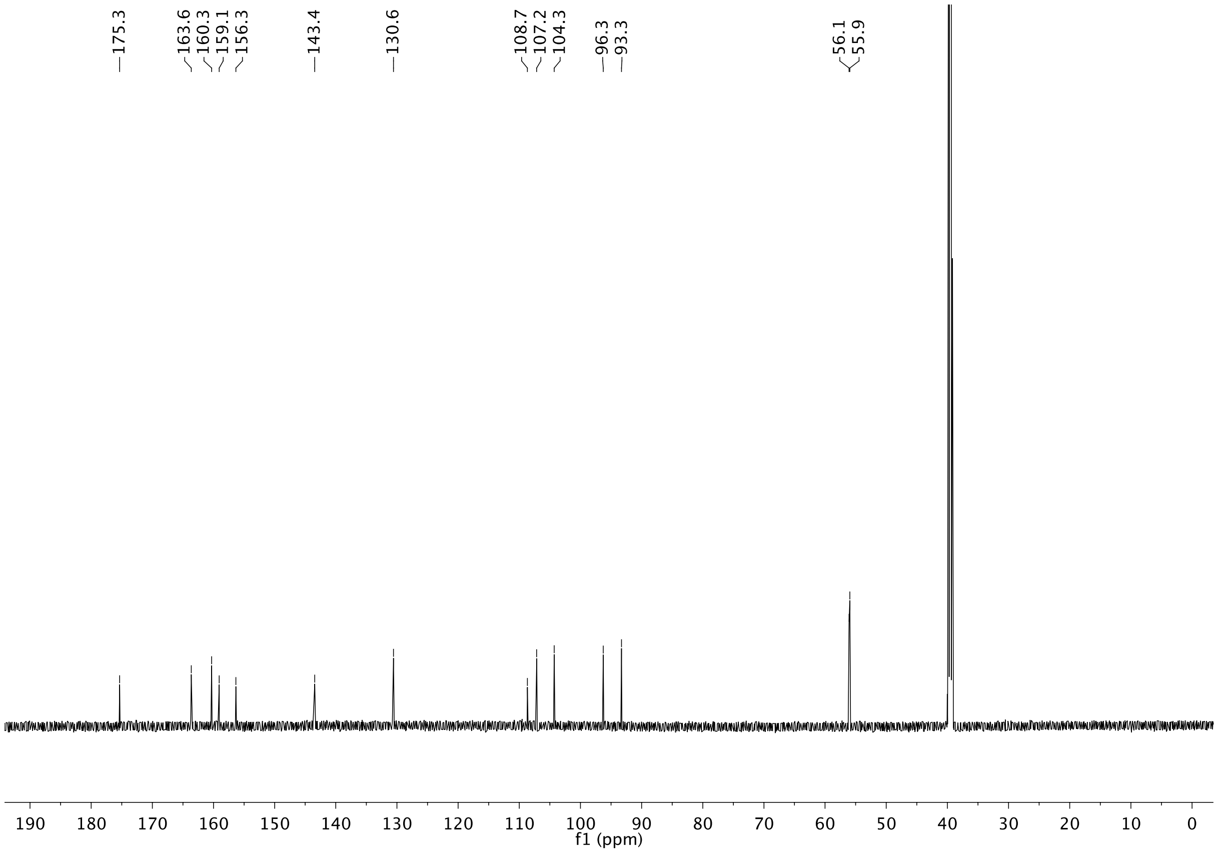
175.3
163.6
160.3
159.1
156.3
143.4
130.6
108.7
107.2
104.3
96.3
93.3
56.1
55.9
190 180 170 160 150 140 130 120 110 100 90 80 70 60 50 40 30 20 10 0
f1 (ppm)

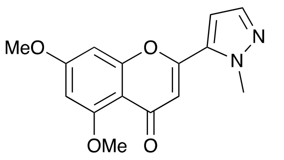
MeO
O
N
N
OMe
O

25

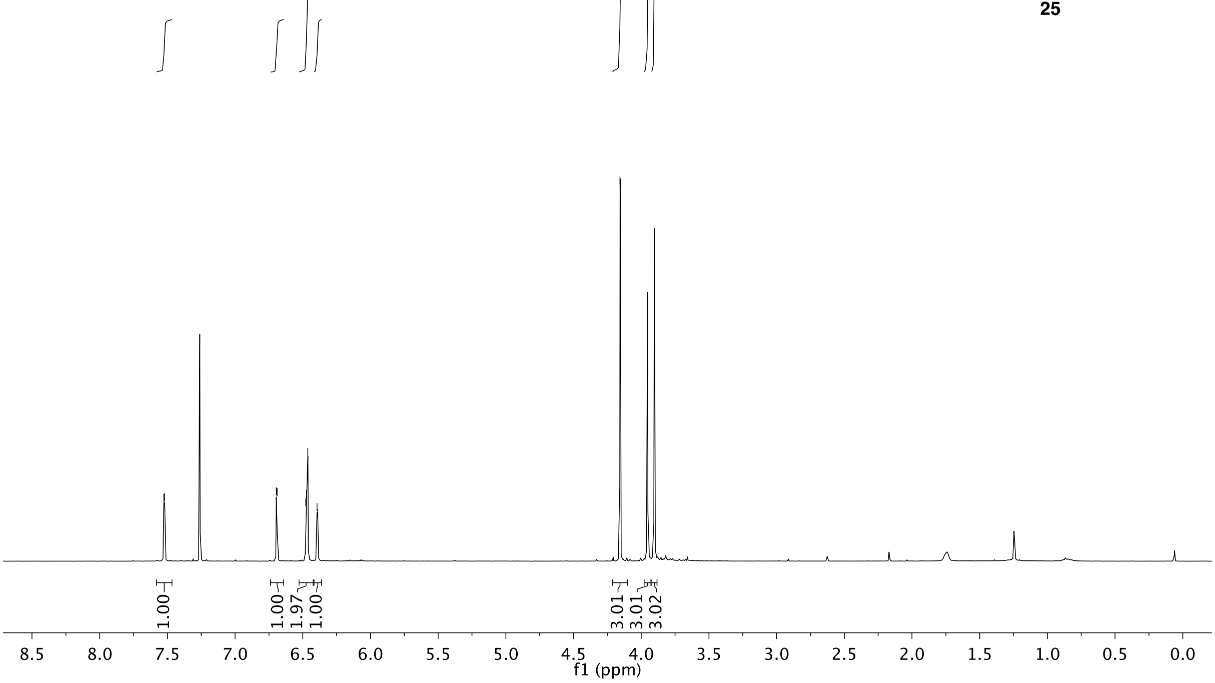
7.53
7.52
6.70
6.69
6.48
6.47
6.46
6.39
6.39
4.16
3.95
3.90
1.00
1.00
1.97
1.00
3.01
3.01
3.02
8.5 8.0 7.5 7.0 6.5 6.0 5.5 5.0 4.5 4.0 3.5 3.0 2.5 2.0 1.5 1.0 0.5 0.0
f1 (ppm)

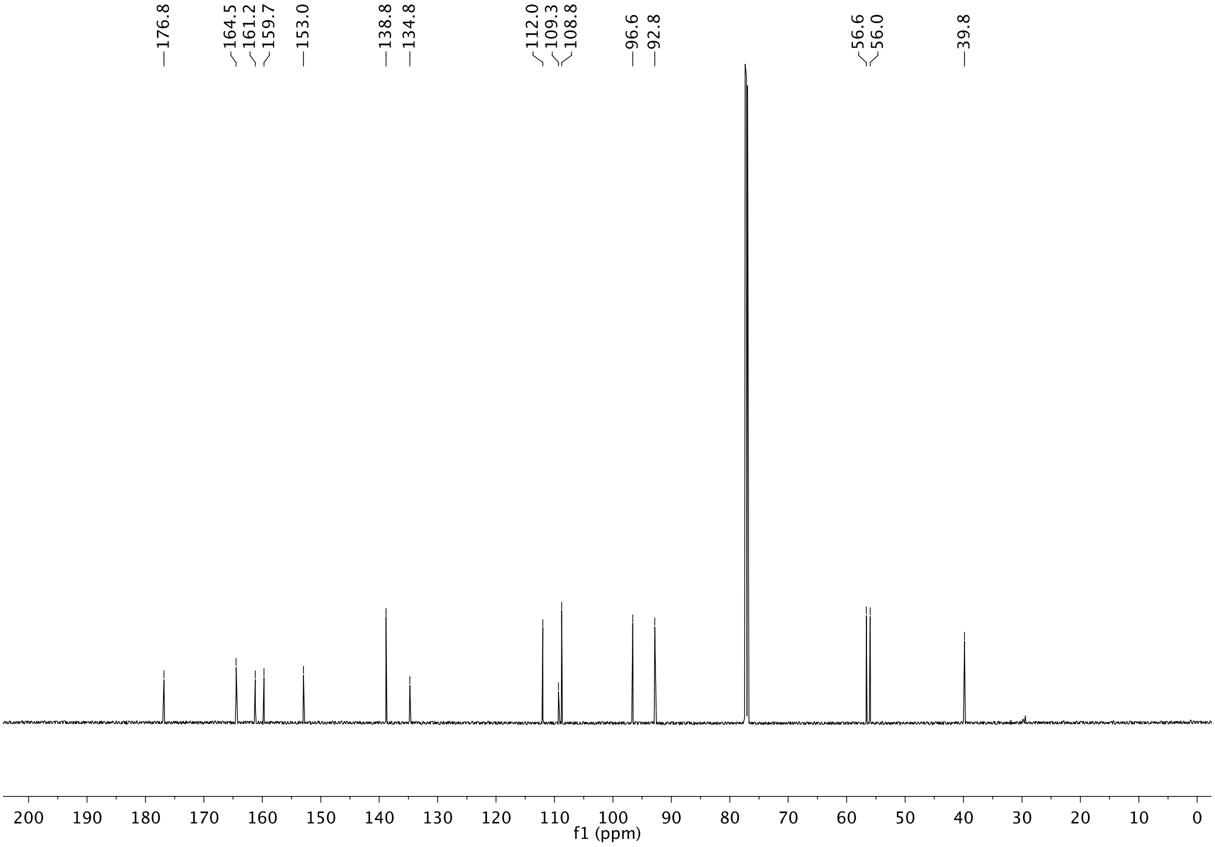
176.8
164.5
161.2
159.7
153.0
138.8
134.8
112.0
109.3
108.8
96.6
92.8
56.6
56.0
39.8
200 190 180 170 160 150 140 130 120 110 100 90 80 70 60 50 40 30 20 10 0
f1 (ppm)

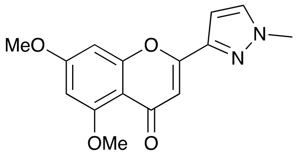
MeO
O
N
N
OMe
O

26

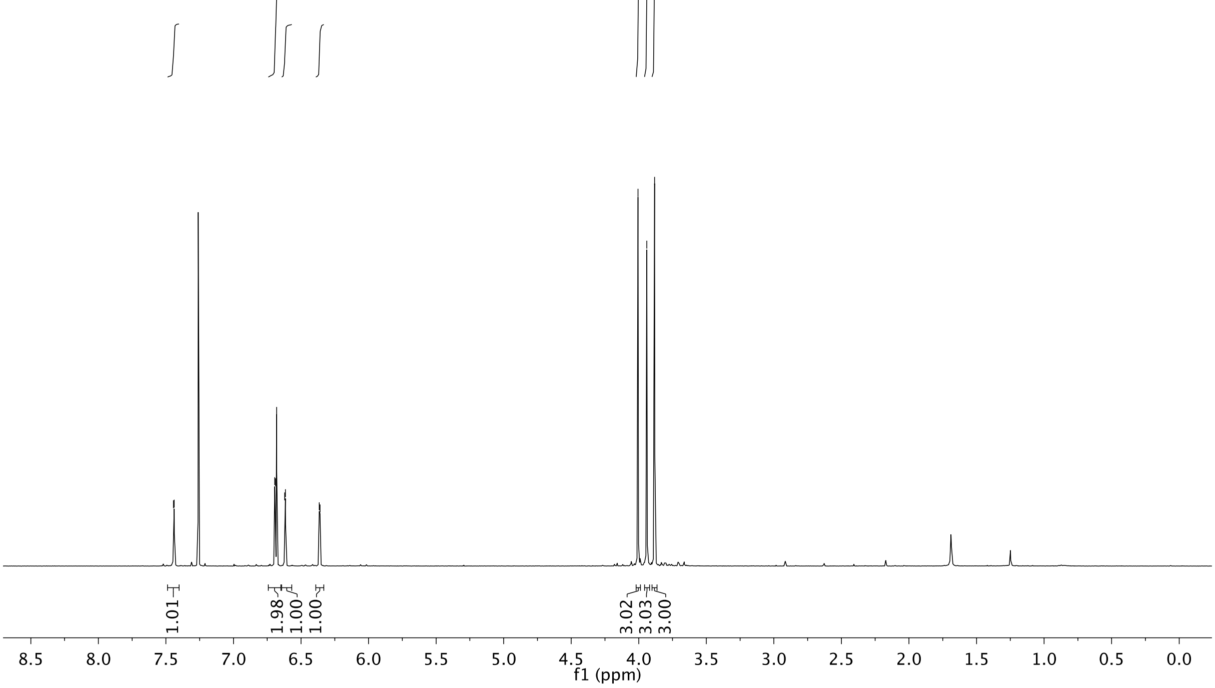
7.44
7.44
6.69
6.69
6.68
6.62
6.62
6.37
6.36
4.01
3.94
3.88
1.01
1.98
1.00
1.00
3.02
3.03
3.00
8.5 8.0 7.5 7.0 6.5 6.0 5.5 5.0 4.5 4.0 3.5 3.0 2.5 2.0 1.5 1.0 0.5 0.0
f1 (ppm)

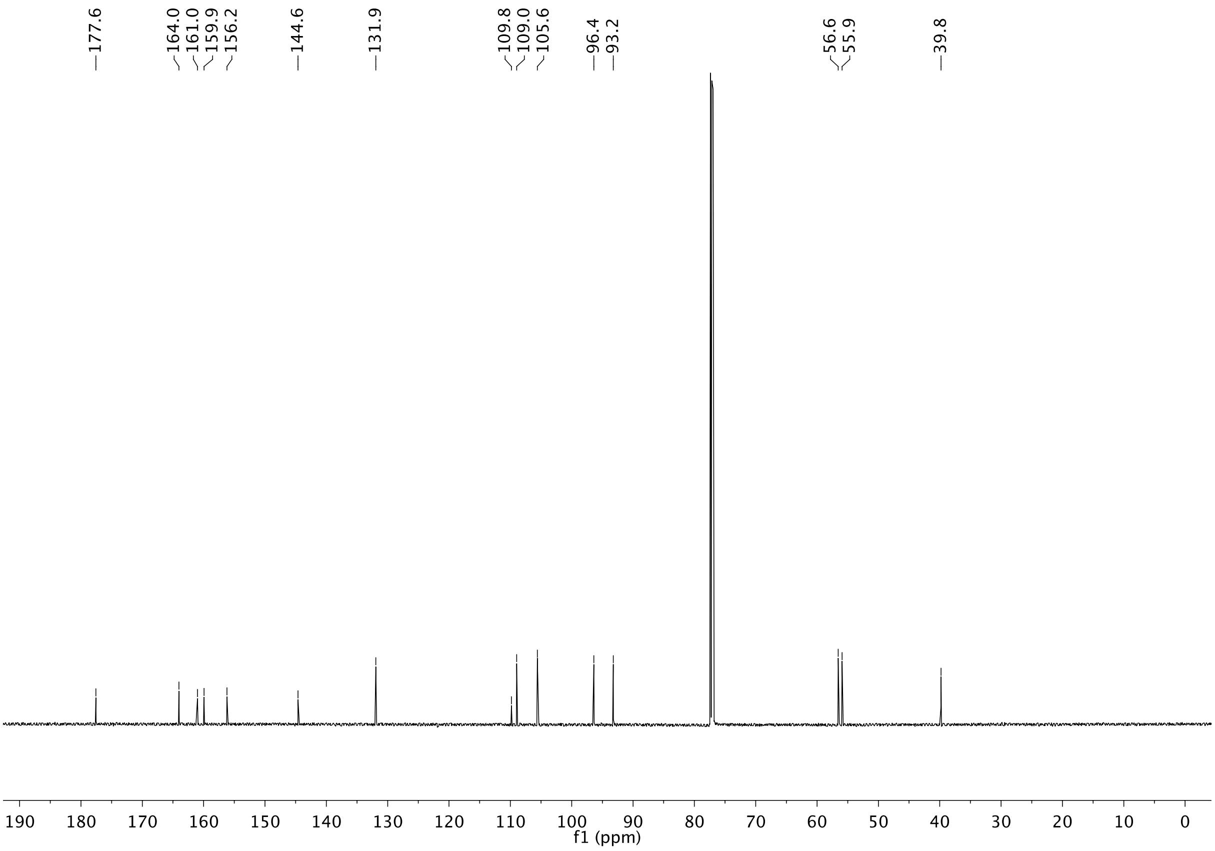
177.6
164.0
161.0
159.9
156.2
144.6
131.9
109.8
109.0
105.6
96.4
93.2
56.6
55.9
39.8
190 180 170 160 150 140 130 120 110 100 90 80 70 60 50 40 30 20 10 0
f1 (ppm)

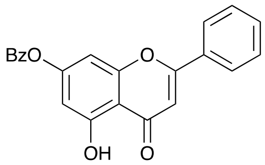
BzO
O
OH
O

27

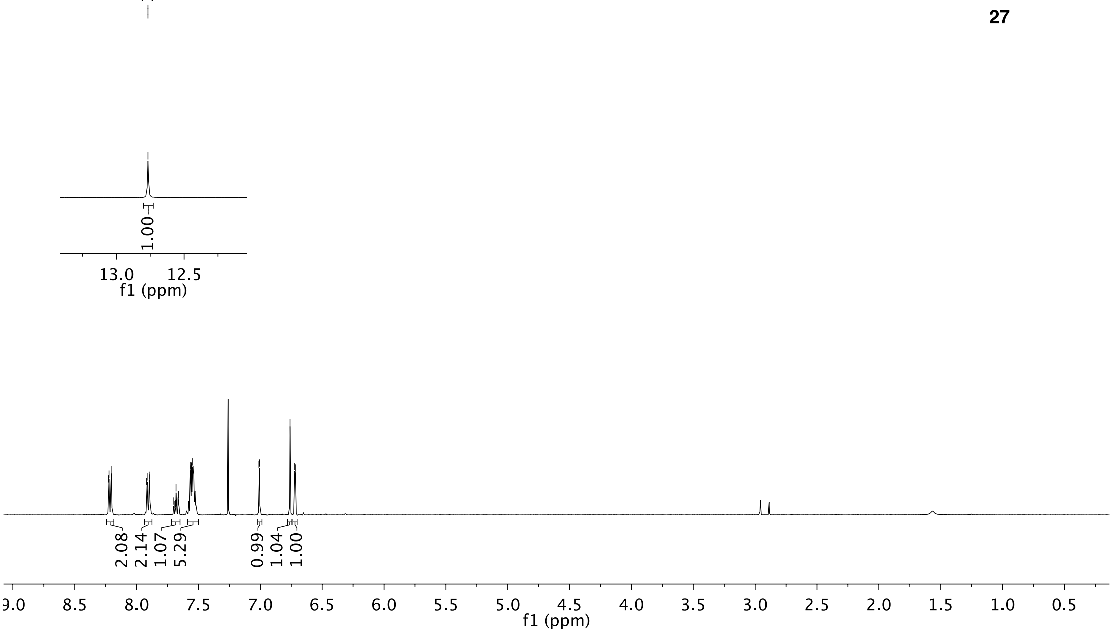
12.77
1.00
13.0
12.5
f1 (ppm)
2.08
2.14
1.07
5.29
0.99
1.04
1.00
8.5
8.0
7.5
7.0
6.5
6.0
5.5
5.0
4.5
4.0
3.5
3.0
2.5
2.0
1.5
1.0
0.5
f1 (ppm)

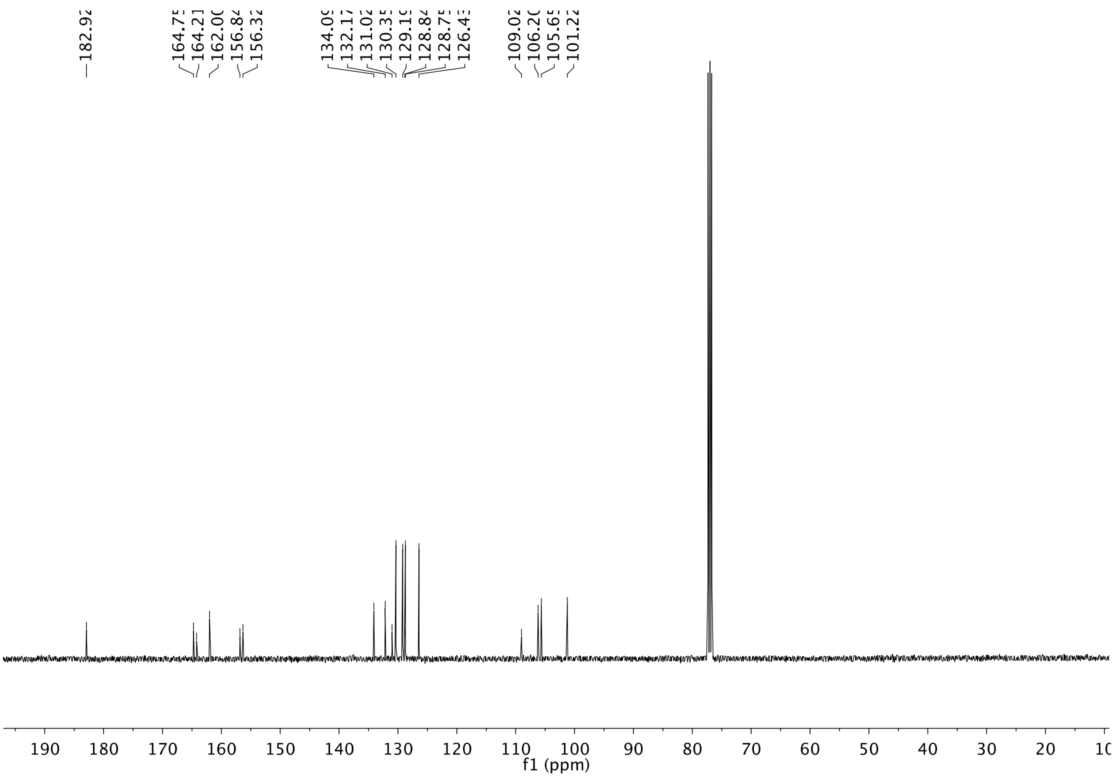
190
180
170
160
150
140
130
120
110
100
90
80
70
60
50
40
30
20
f1 (ppm)

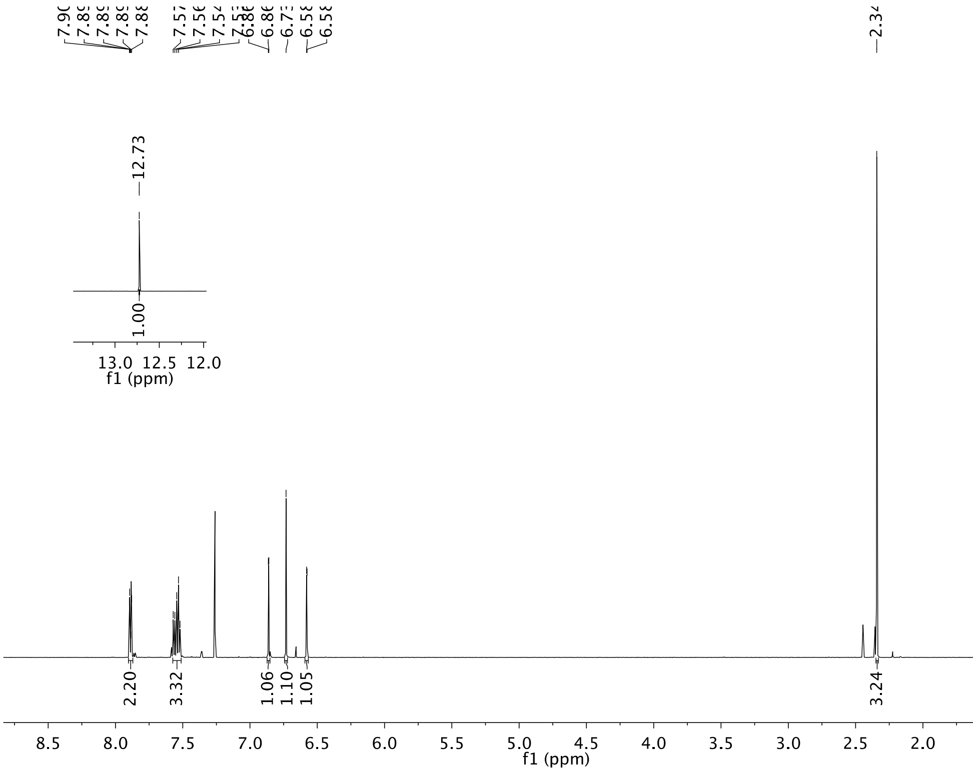

12.73
1.00
13.0 12.5 12.0
f1 (ppm)
2.34
2.20
3.32
1.06
1.10
1.05
3.24
8.5 8.0 7.5 7.0 6.5 6.0 5.5 5.0 4.5 4.0 3.5 3.0 2.5 2.0 1.5 1.0
f1 (ppm)

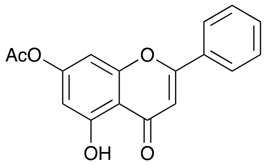

AcO
O
OH
O

28

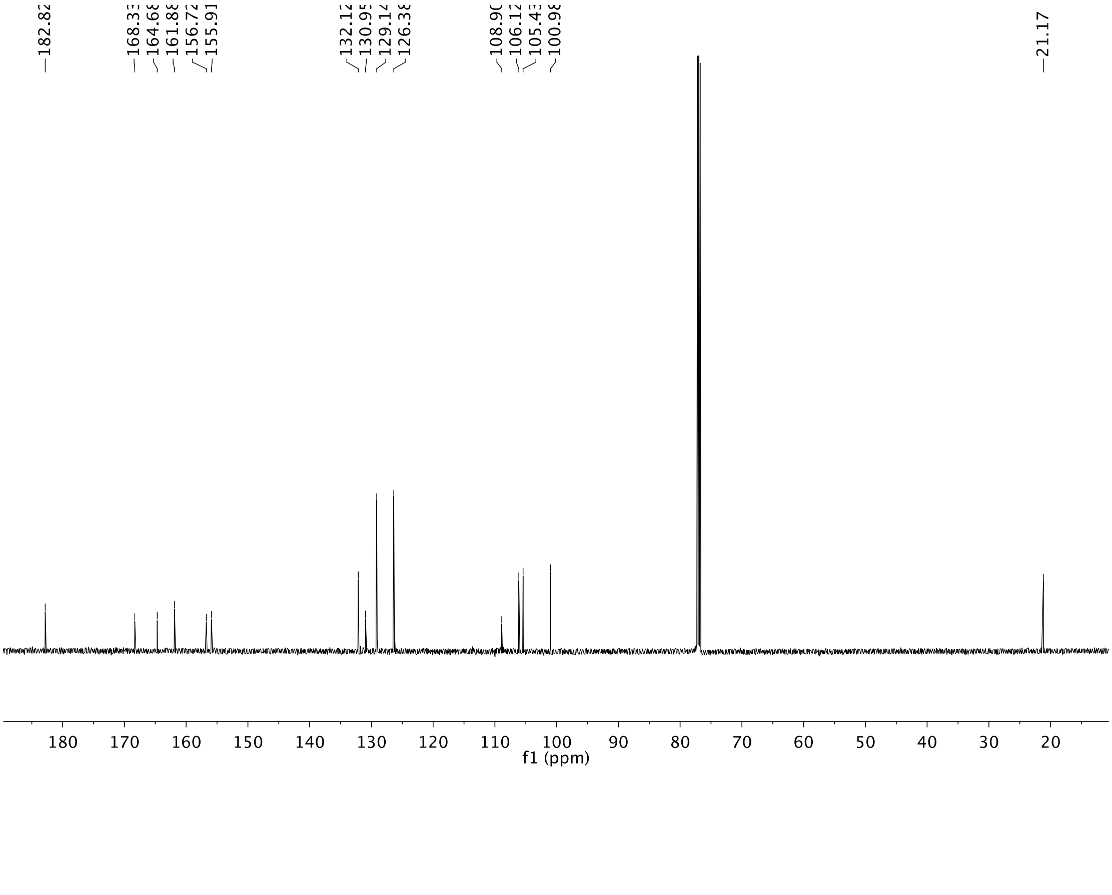

182.82
21.17
180 170 160 150 140 130 120 110 100 90 80 70 60 50 40 30 20
f1 (ppm)

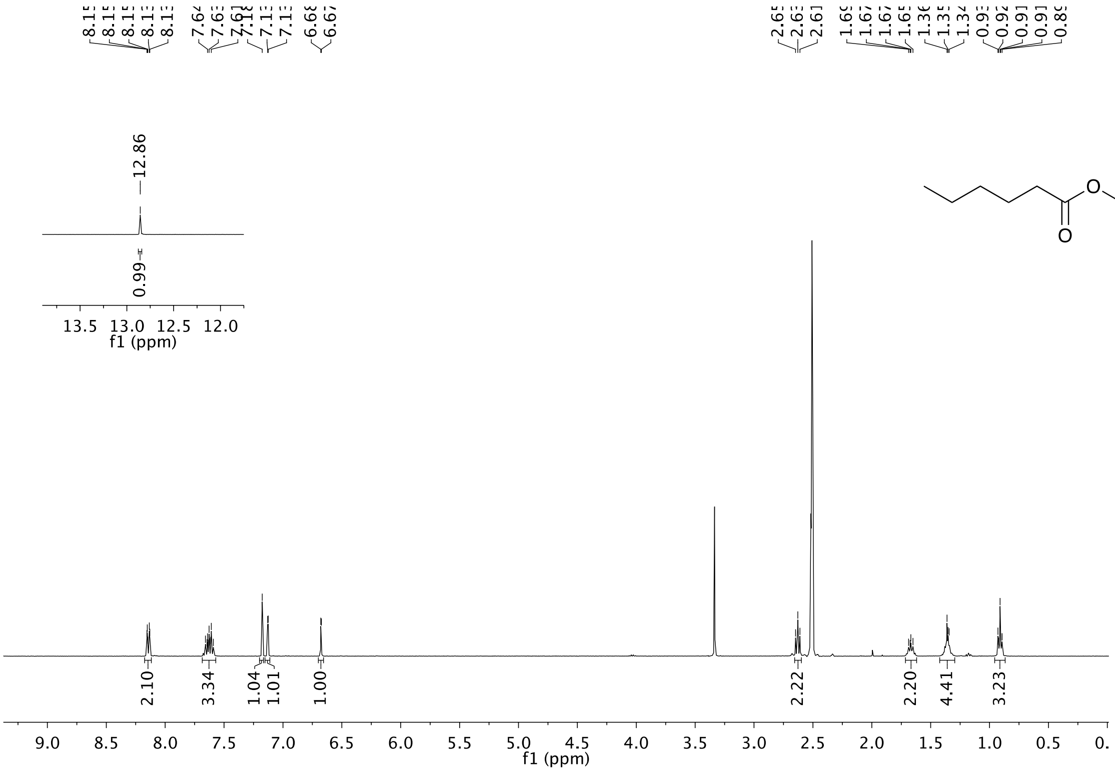

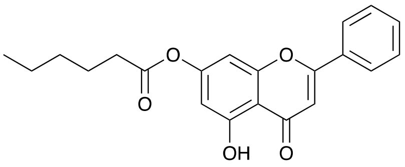

29

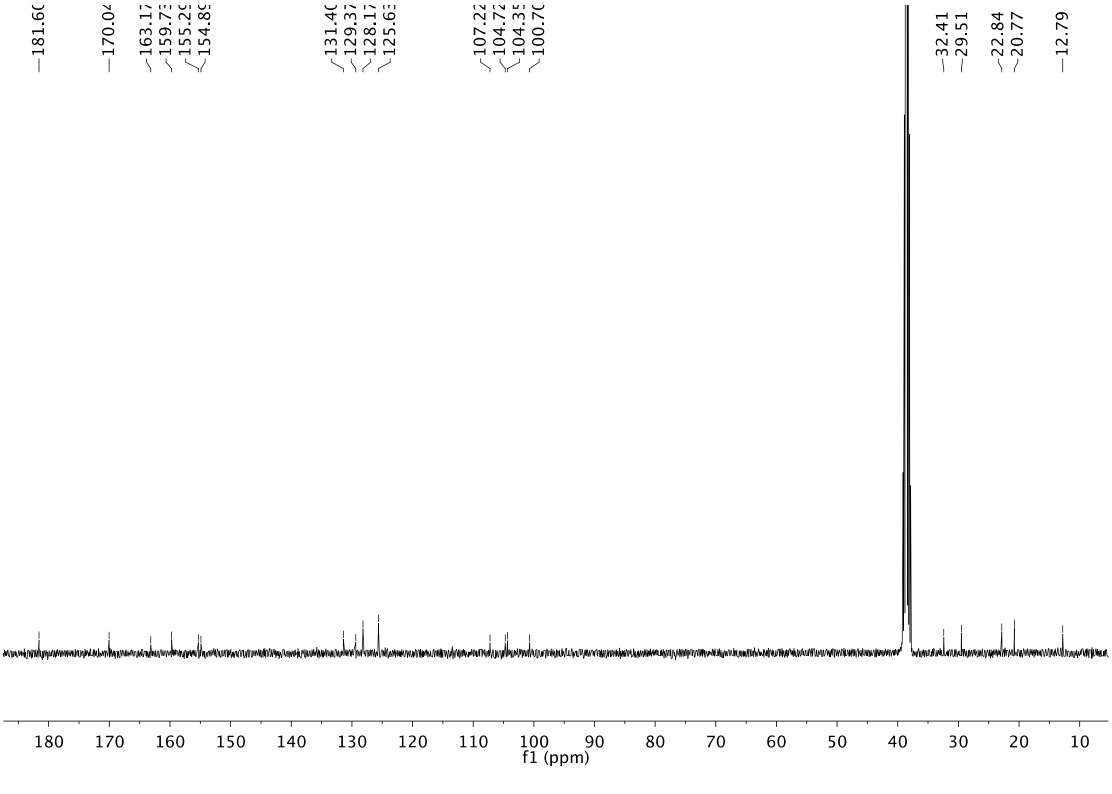

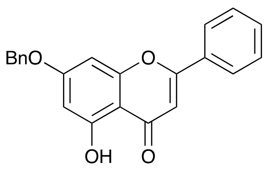
BnO
O
OH
O

30

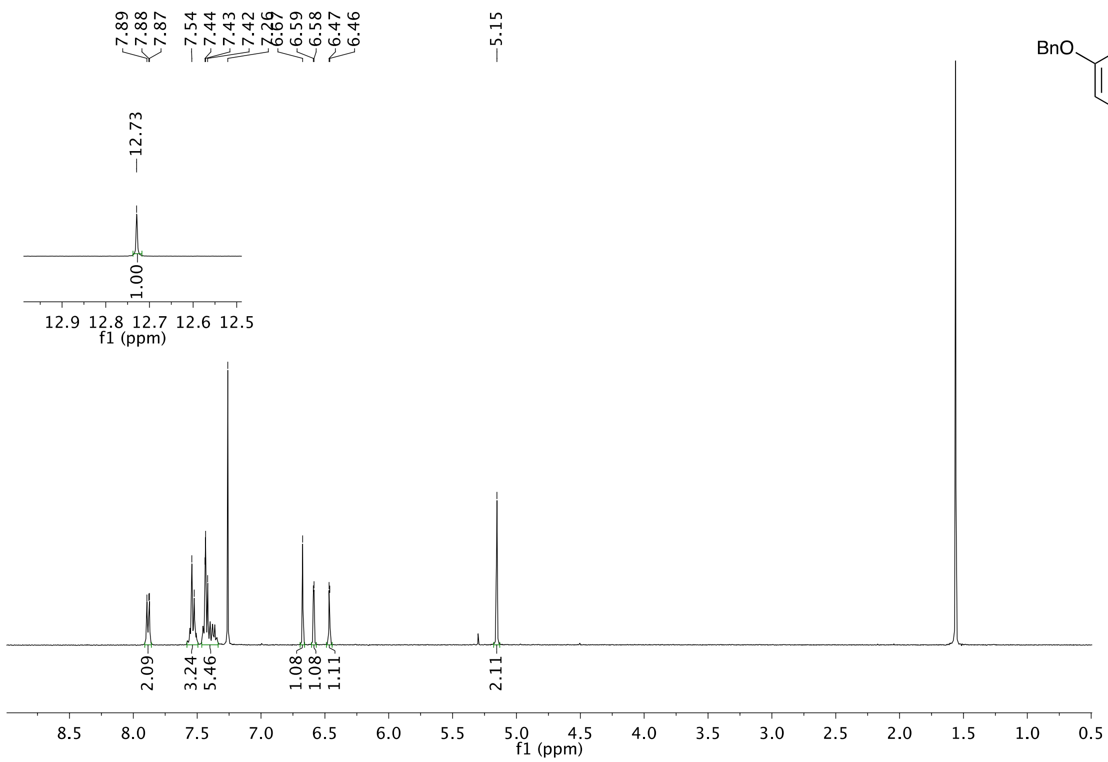
7.89
7.88
7.87
7.54
7.44
7.43
7.42
7.26
6.67
6.59
6.58
6.47
6.46
5.15
12.73
1.00
12.9 12.8 12.7 12.6 12.5
f1 (ppm)
2.09
3.24
5.46
1.08
1.08
1.11
2.11
8.5 8.0 7.5 7.0 6.5 6.0 5.5 5.0 4.5 4.0 3.5 3.0 2.5 2.0 1.5 1.0 0.5
f1 (ppm)

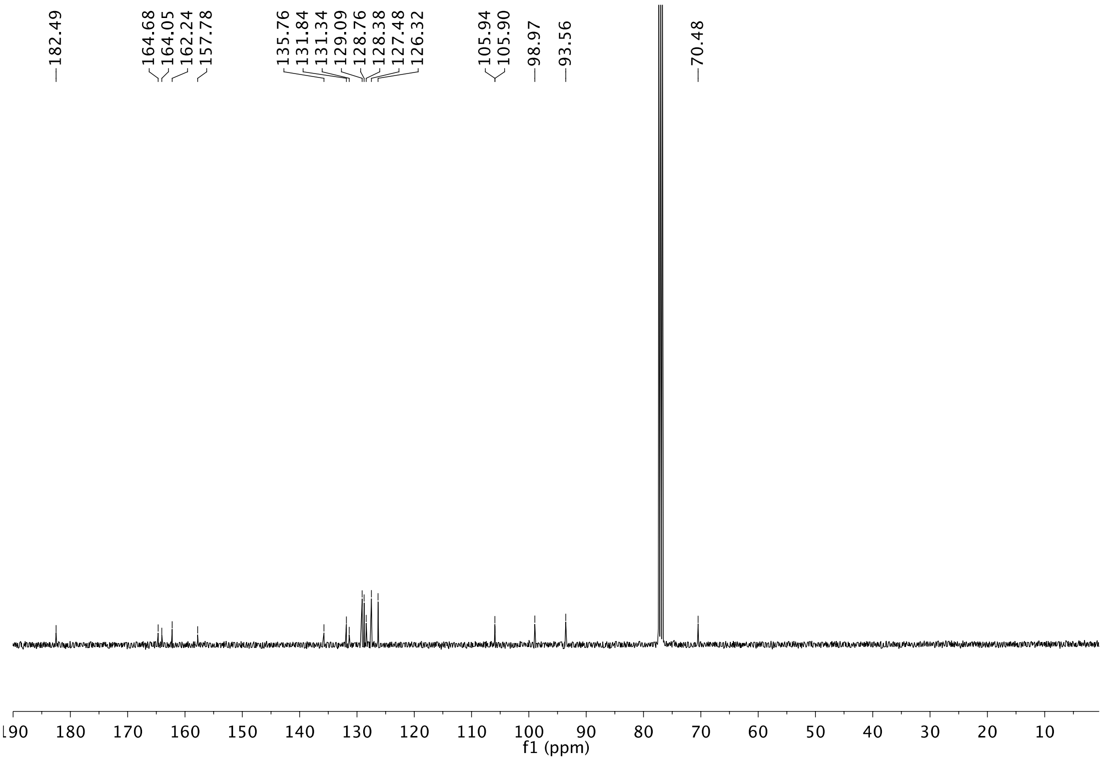
182.49
164.68
164.05
162.24
157.78
135.76
131.84
131.34
129.09
128.76
128.38
127.48
126.32
105.94
105.90
98.97
93.56
70.48
180 170 160 150 140 130 120 110 100 90 80 70 60 50 40 30 20 10
f1 (ppm)

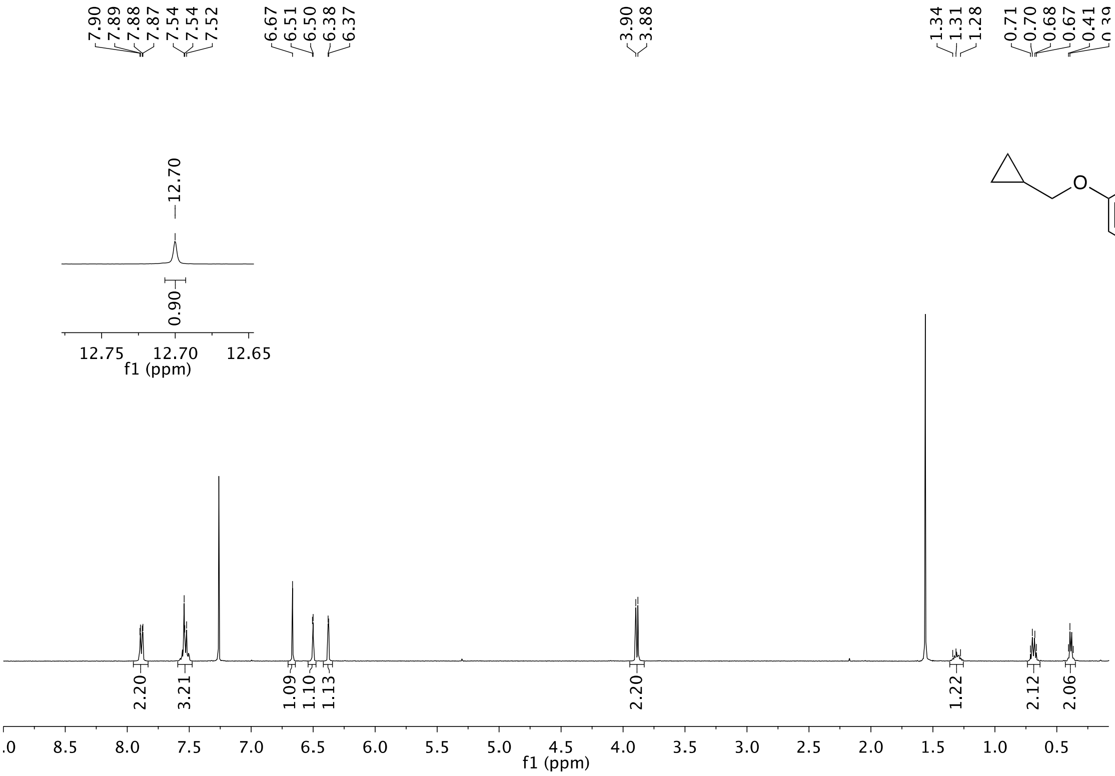
7.90 7.89 7.88 7.87 7.54 7.54 7.52
6.67 6.51 6.50 6.38 6.37
3.90 3.88
1.34 1.31 1.28
0.71 0.70 0.68 0.67 0.41
12.70
0.90
12.75 12.70 12.65
f1 (ppm)
2.20 3.21 1.09 1.10 1.13 2.20 1.22 2.12 2.06
8.5 8.0 7.5 7.0 6.5 6.0 5.5 5.0 4.5 4.0 3.5 3.0 2.5 2.0 1.5 1.0 0.5
f1 (ppm)

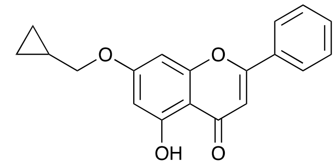

31

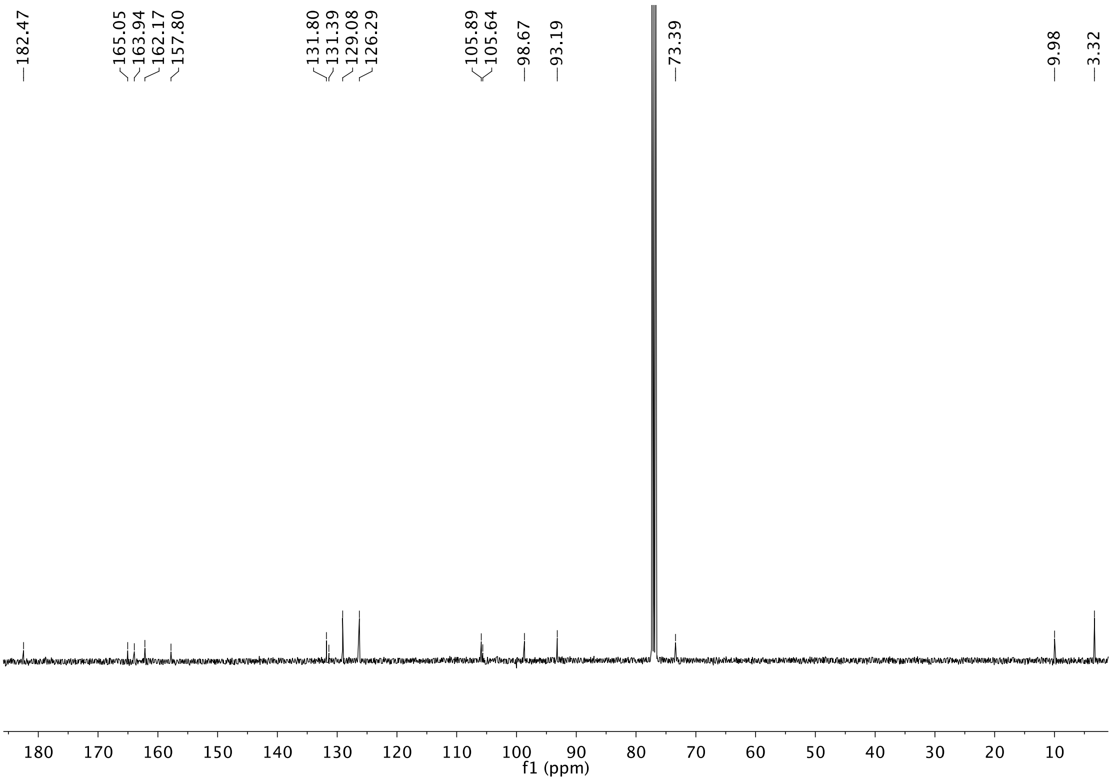
182.47
165.05 163.94 162.17 157.80
131.80 131.39 129.08 126.29
105.89 105.64
98.67
93.19
73.39
9.98
3.32
180 170 160 150 140 130 120 110 100 90 80 70 60 50 40 30 20 10
f1 (ppm)

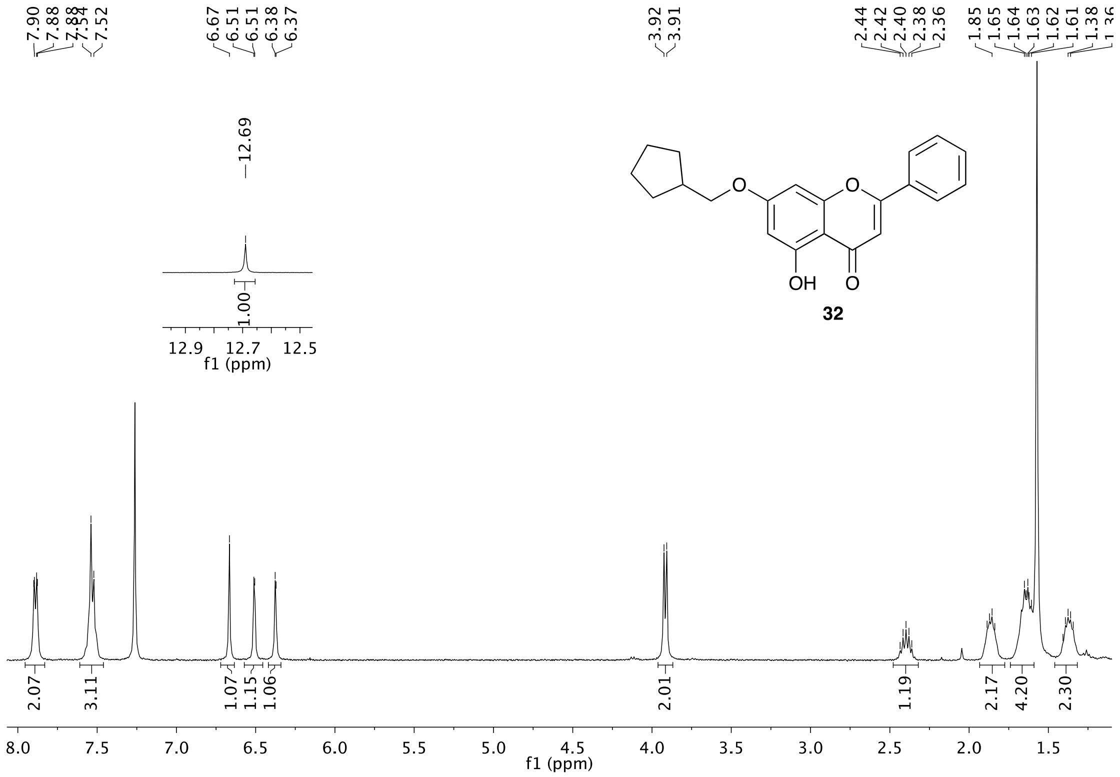
7.90
7.88
7.88
7.54
7.52
6.67
6.51
6.51
6.38
6.37
3.92
3.91
2.44
2.42
2.40
2.38
2.36
1.85
1.65
1.64
1.63
1.62
1.61
1.38
12.69
1.00
12.9 12.7 12.5
f1 (ppm)
OH
O
32
2.07
3.11
1.07
1.15
1.06
2.01
1.19
2.17
4.20
2.30
8.0 7.5 7.0 6.5 6.0 5.5 5.0 4.5 4.0 3.5 3.0 2.5 2.0 1.5
f1 (ppm)

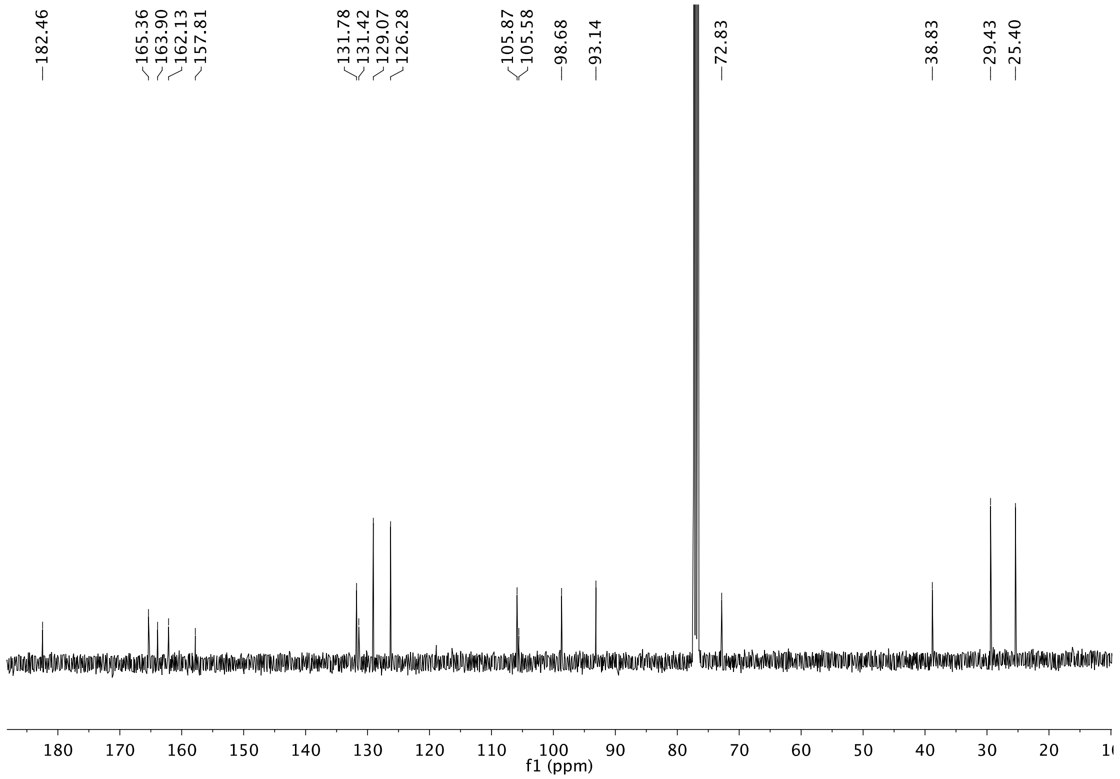
182.46
165.36
163.90
162.13
157.81
131.78
131.42
129.07
126.28
105.87
105.58
98.68
93.14
72.83
38.83
29.43
25.40
180 170 160 150 140 130 120 110 100 90 80 70 60 50 40 30 20
f1 (ppm)

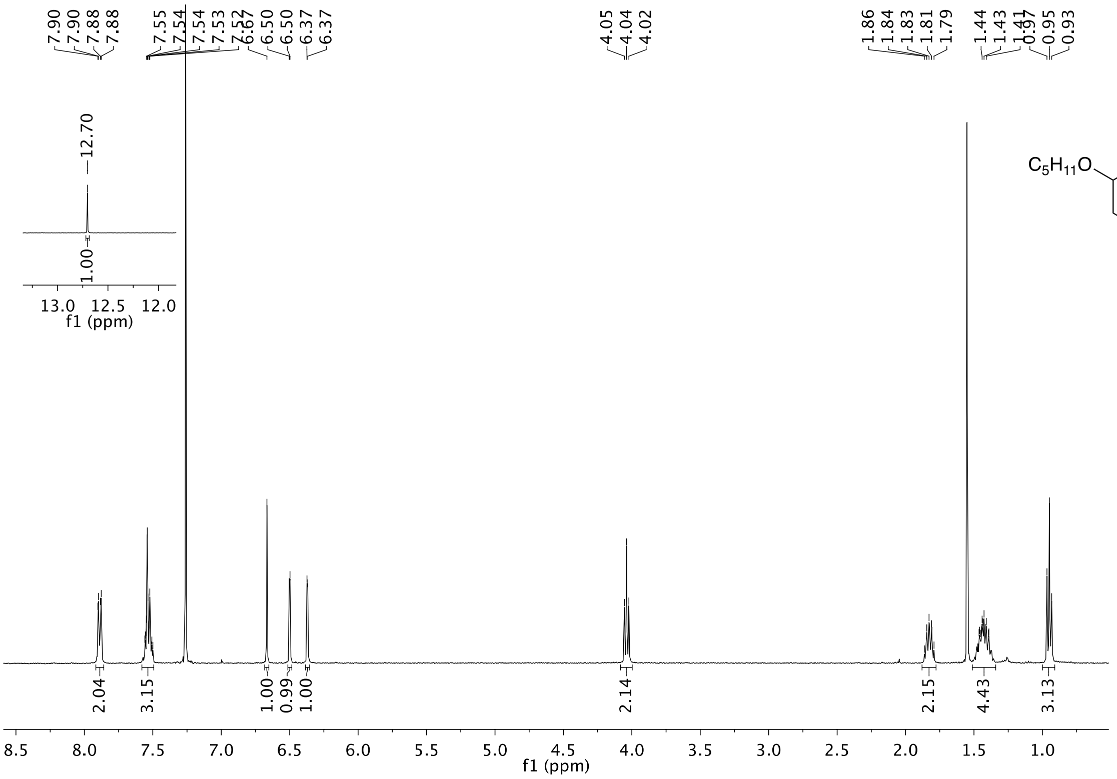

7.90 7.90 7.88 7.88 7.55 7.54 7.54 7.53 7.52 6.67 6.50 6.50 6.37 6.37
4.05 4.04 4.02
1.86 1.84 1.83 1.81 1.79 1.44 1.43 1.41 0.97 0.95 0.93
12.70
1.00
13.0 12.5 12.0
f1 (ppm)
2.04 3.15 1.00 0.99 1.00 2.14 2.15 4.43 3.13
8.5 8.0 7.5 7.0 6.5 6.0 5.5 5.0 4.5 4.0 3.5 3.0 2.5 2.0 1.5 1.0
f1 (ppm)

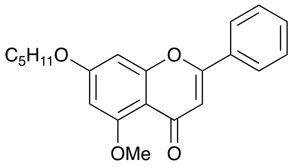

$C_5H_{11}O$
O
OMe
O

33

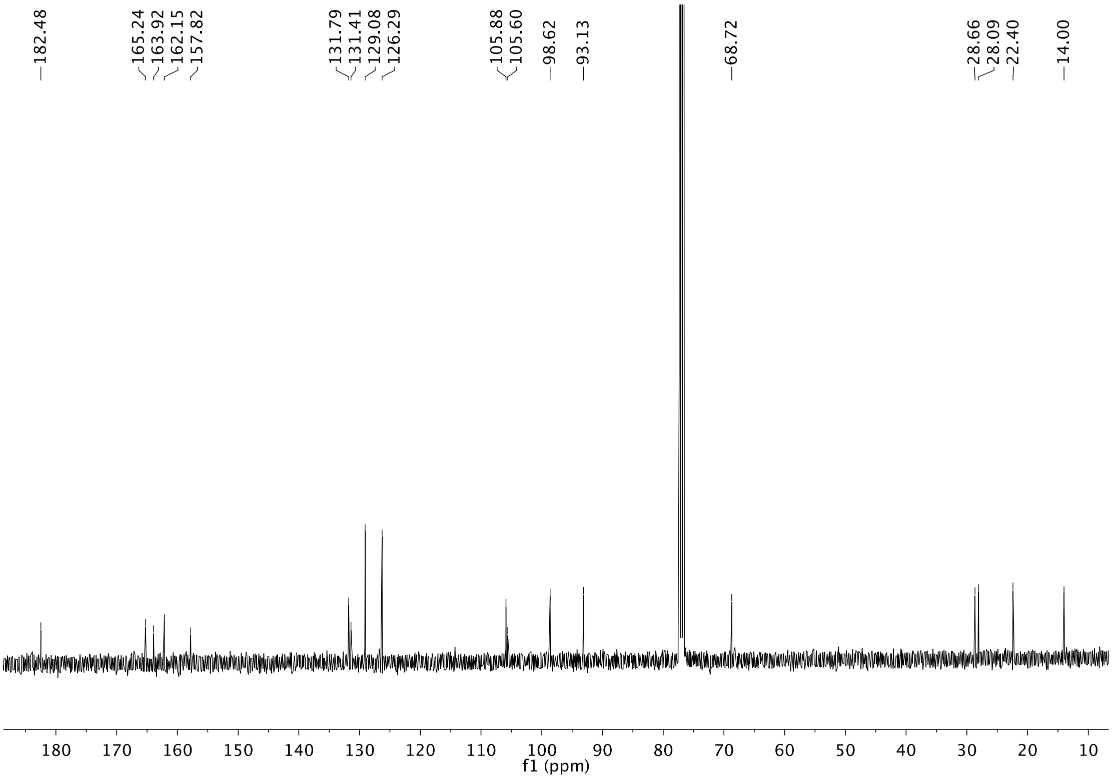

182.48
165.24 163.92 162.15 157.82
131.79 131.41 129.08 126.29
105.88 105.60 98.62 93.13
68.72
28.66 28.09 22.40 14.00
180 170 160 150 140 130 120 110 100 90 80 70 60 50 40 30 20 10
f1 (ppm)

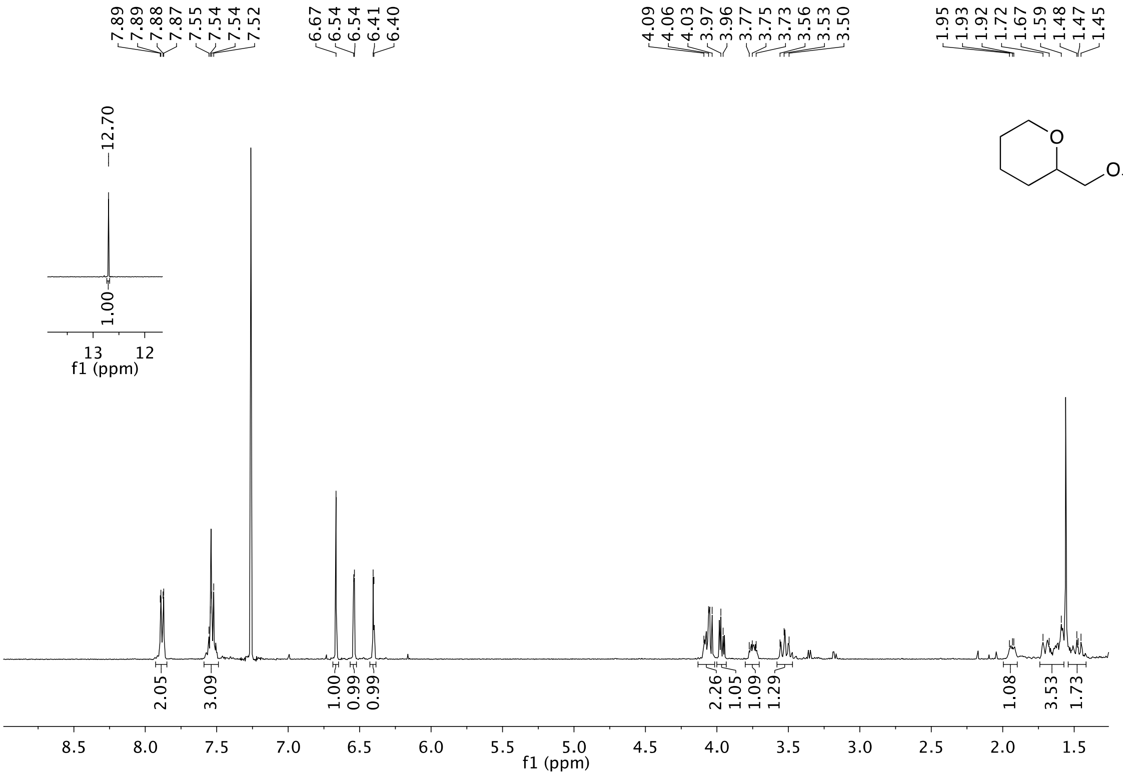
7.89
7.89
7.88
7.87
7.55
7.54
7.54
7.52
6.67
6.54
6.54
6.41
6.40
4.09
4.06
4.03
3.97
3.96
3.77
3.75
3.73
3.56
3.53
3.50
1.95
1.93
1.92
1.72
1.67
1.59
1.48
1.47
1.45
12.70
1.00
13
12
f1 (ppm)
2.05
3.09
1.00
0.99
0.99
2.26
1.05
1.09
1.29
1.08
3.53
1.73
8.5
8.0
7.5
7.0
6.5
6.0
5.5
5.0
4.5
4.0
3.5
3.0
2.5
2.0
1.5
f1 (ppm)

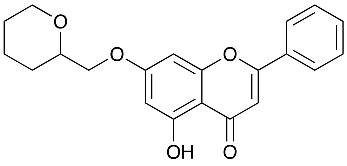
O
O
O
OH
O

34

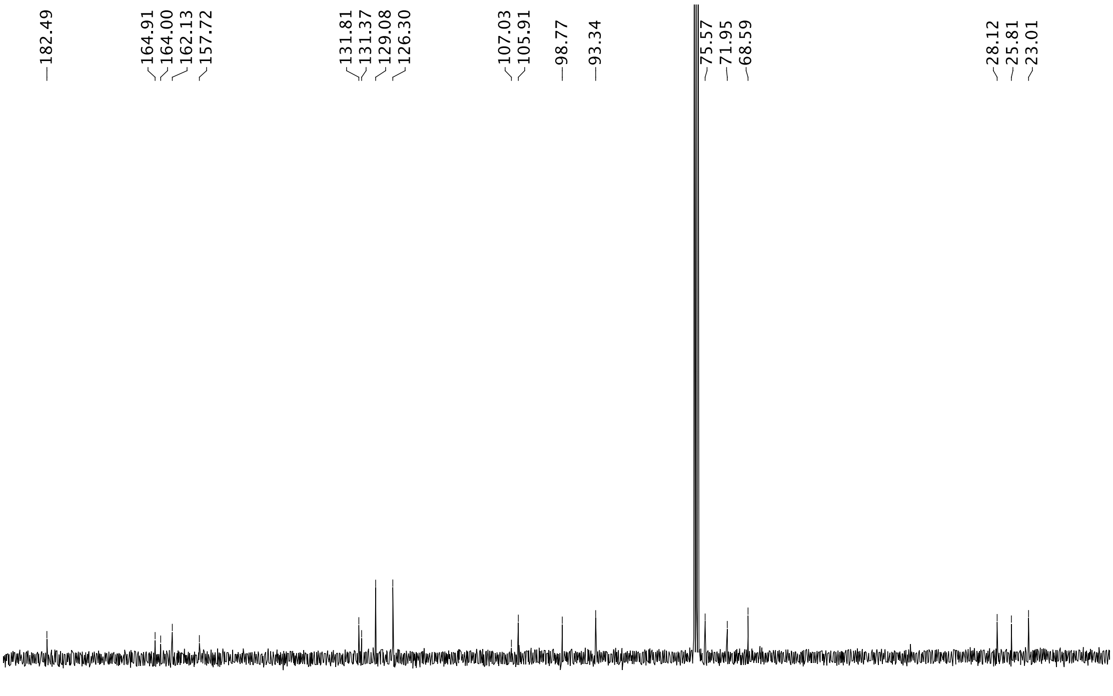
182.49
164.91
164.00
162.13
157.72
131.81
131.37
129.08
126.30
107.03
105.91
98.77
93.34
75.57
71.95
68.59
28.12
25.81
23.01
180
170
160
150
140
130
120
110
100
90
80
70
60
50
40
30
20
f1 (ppm)

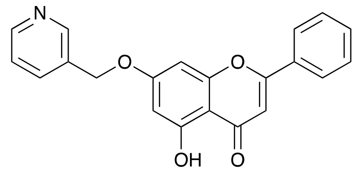
N
O
O
OH
O

35

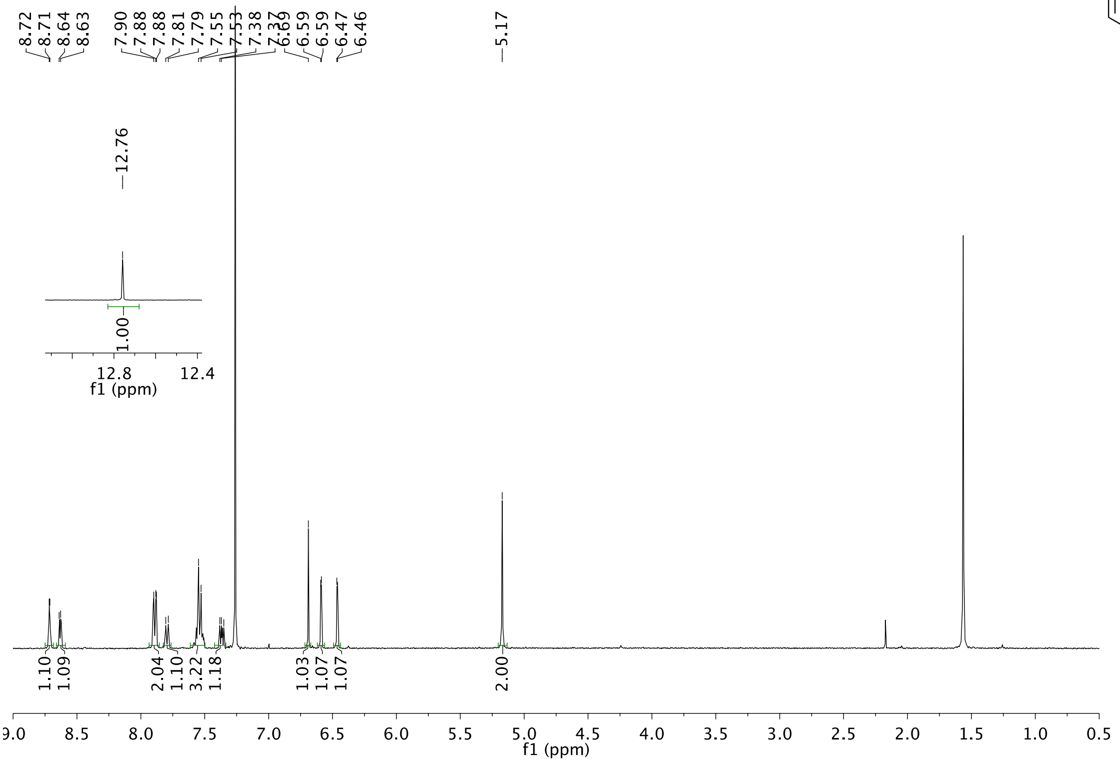
8.72
8.71
8.64
8.63
7.90
7.88
7.88
7.81
7.79
7.55
7.53
7.38
7.37
6.69
6.59
6.59
6.47
6.46
5.17
12.76
1.00
12.8
12.4
f1 (ppm)
1.10
1.09
2.04
1.10
3.22
1.18
1.03
1.07
1.07
2.00
9.0
8.5
8.0
7.5
7.0
6.5
6.0
5.5
5.0
4.5
4.0
3.5
3.0
2.5
2.0
1.5
1.0
0.5
f1 (ppm)

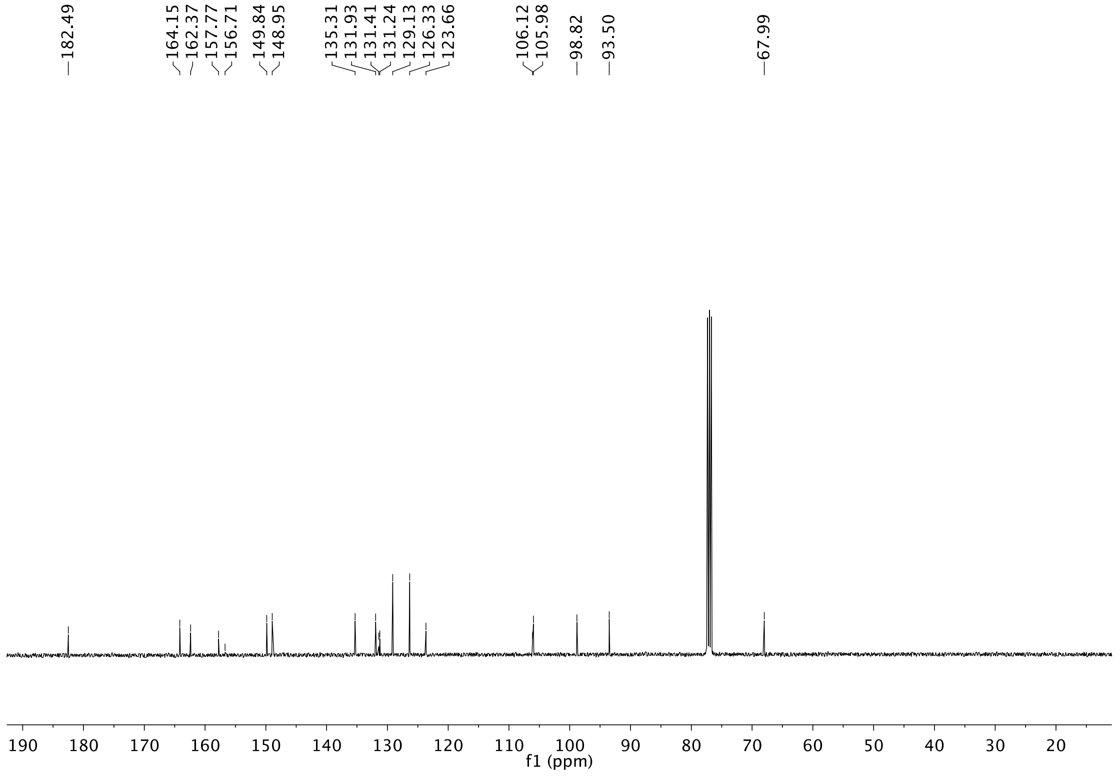
182.49
164.15
162.37
157.77
156.71
149.84
148.95
135.31
131.93
131.41
131.24
129.13
126.33
123.66
106.12
105.98
98.82
93.50
67.99
190
180
170
160
150
140
130
120
110
100
90
80
70
60
50
40
30
20
f1 (ppm)

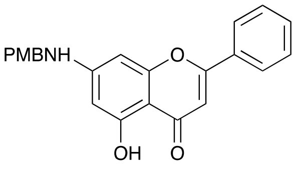
PMBNH
O
OH
O

36

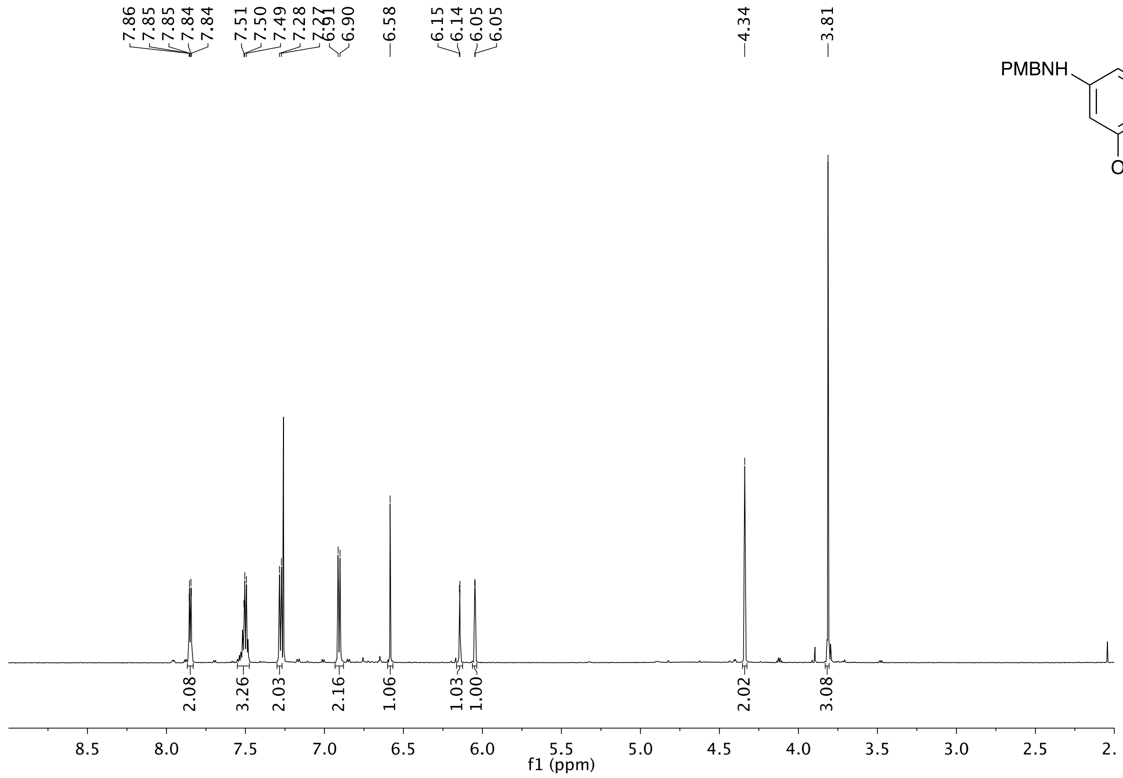
7.86
7.85
7.85
7.84
7.84
7.51
7.50
7.49
7.28
7.27
6.91
6.90
6.58
6.15
6.14
6.05
6.05
4.34
3.81
2.08
3.26
2.03
2.16
1.06
1.03
1.00
2.02
3.08
8.5
8.0
7.5
7.0
6.5
6.0
5.5
5.0
4.5
4.0
3.5
3.0
2.5
f1 (ppm)

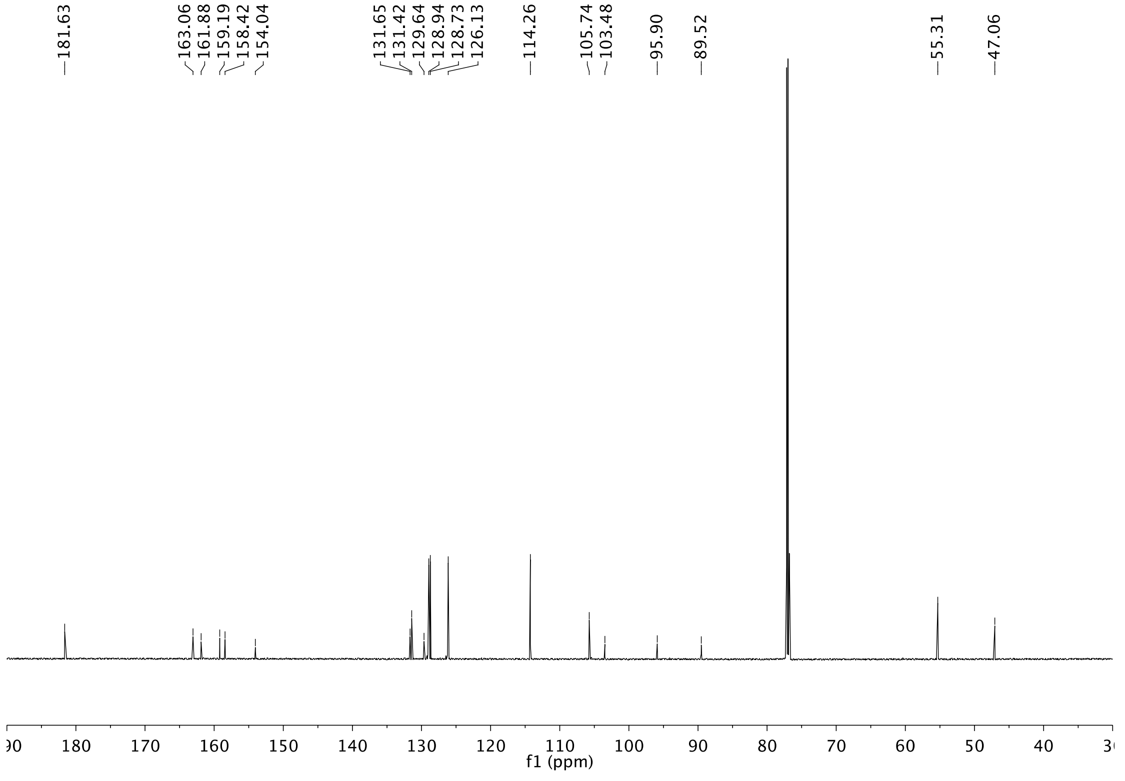
181.63
163.06
161.88
159.19
158.42
154.04
131.65
131.42
129.64
128.94
128.73
126.13
114.26
105.74
103.48
95.90
89.52
55.31
47.06
180
170
160
150
140
130
120
110
100
90
80
70
60
50
40
f1 (ppm)

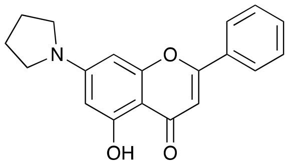
OH
O
N
O

37

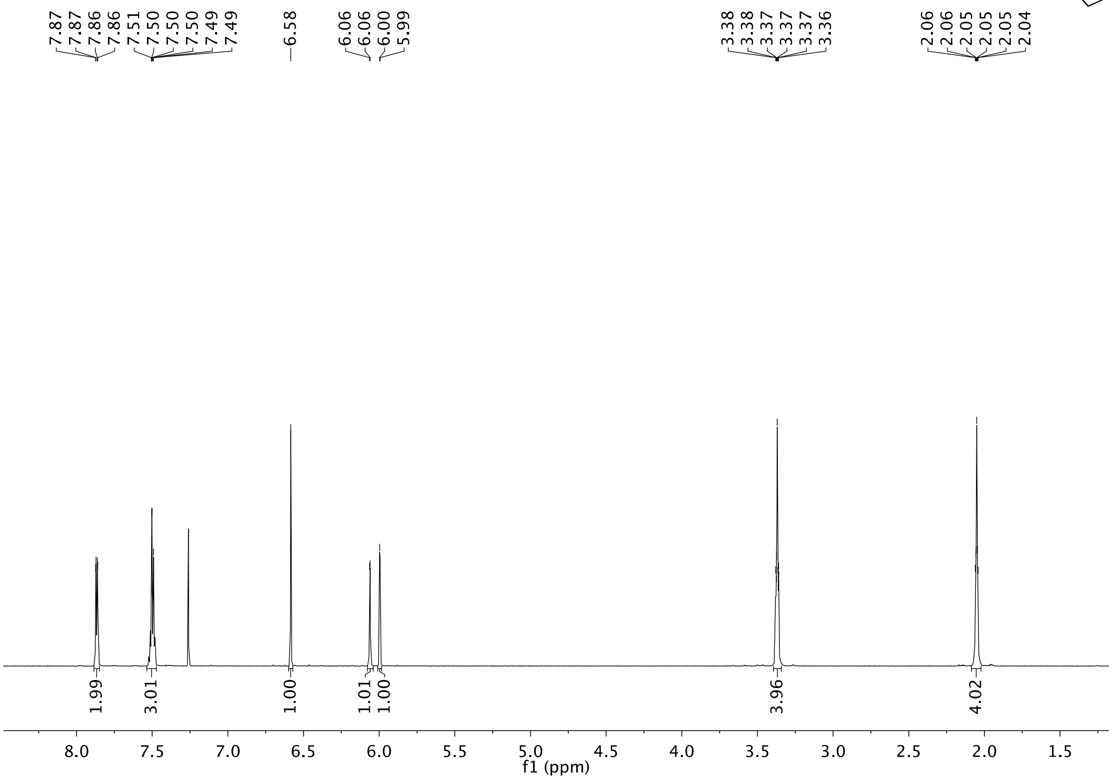
7.87
7.87
7.86
7.86
7.51
7.50
7.50
7.49
7.49
6.58
6.06
6.06
6.00
5.99
3.38
3.38
3.37
3.37
3.37
3.36
2.06
2.06
2.05
2.05
2.05
2.04
1.99
3.01
1.00
1.01
1.00
3.96
4.02
8.0
7.5
7.0
6.5
6.0
5.5
5.0
4.5
4.0
3.5
3.0
2.5
2.0
1.5
f1 (ppm)

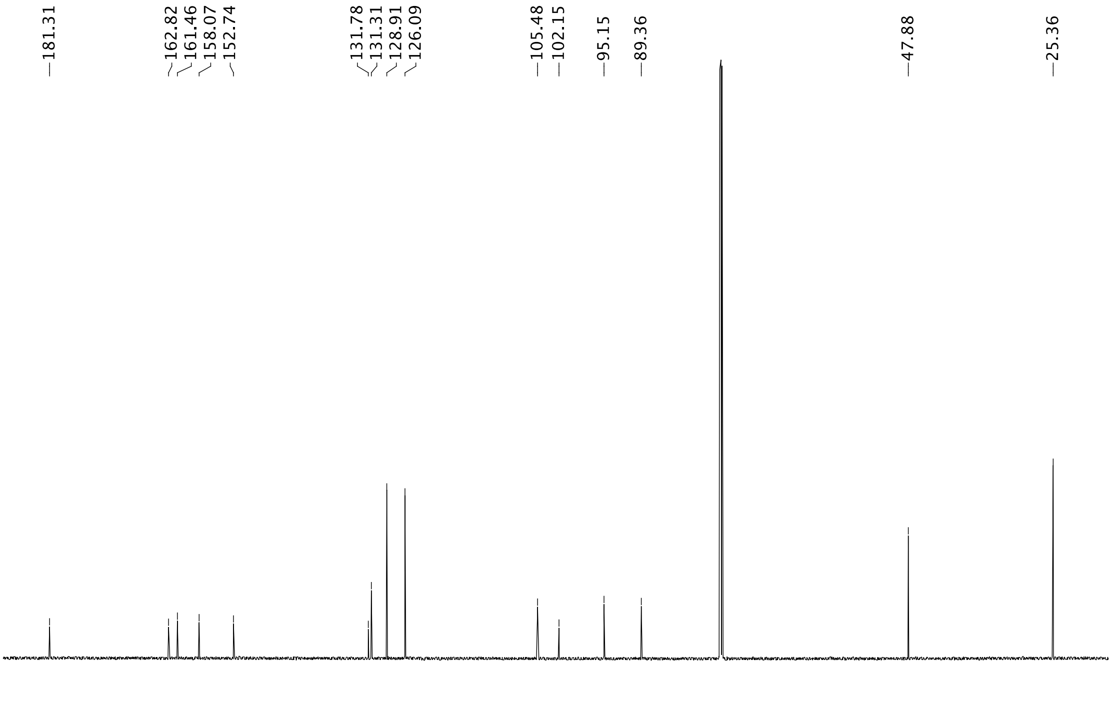
181.31
162.82
161.46
158.07
152.74
131.78
131.31
128.91
126.09
105.48
102.15
95.15
89.36
47.88
25.36
180
170
160
150
140
130
120
110
100
90
80
70
60
50
40
30
20
f1 (ppm)

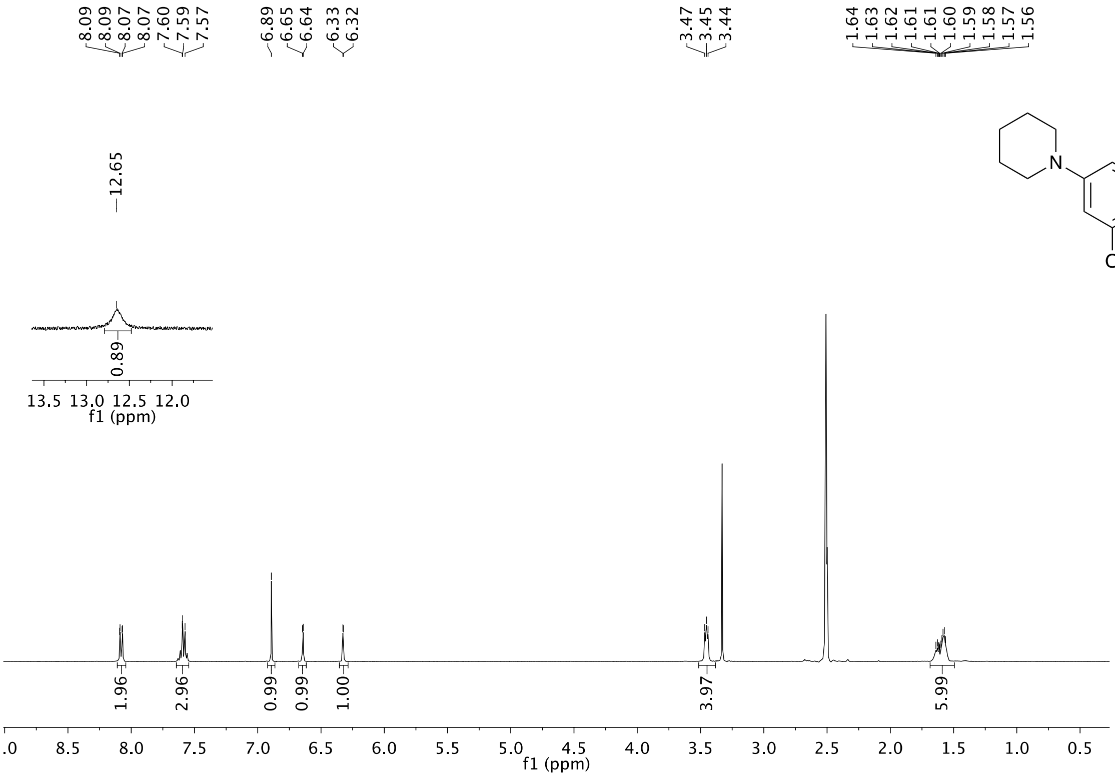
8.09 8.09 8.07 8.07 7.60 7.59 7.57
6.89 6.65 6.64 6.33 6.32
3.47 3.45 3.44
1.64 1.63 1.62 1.61 1.61 1.60 1.59 1.58 1.57 1.56
12.65
0.89
13.5 13.0 12.5 12.0
f1 (ppm)
1.96 2.96 0.99 0.99 1.00 3.97 5.99
f1 (ppm)

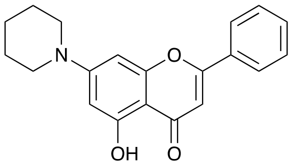

38

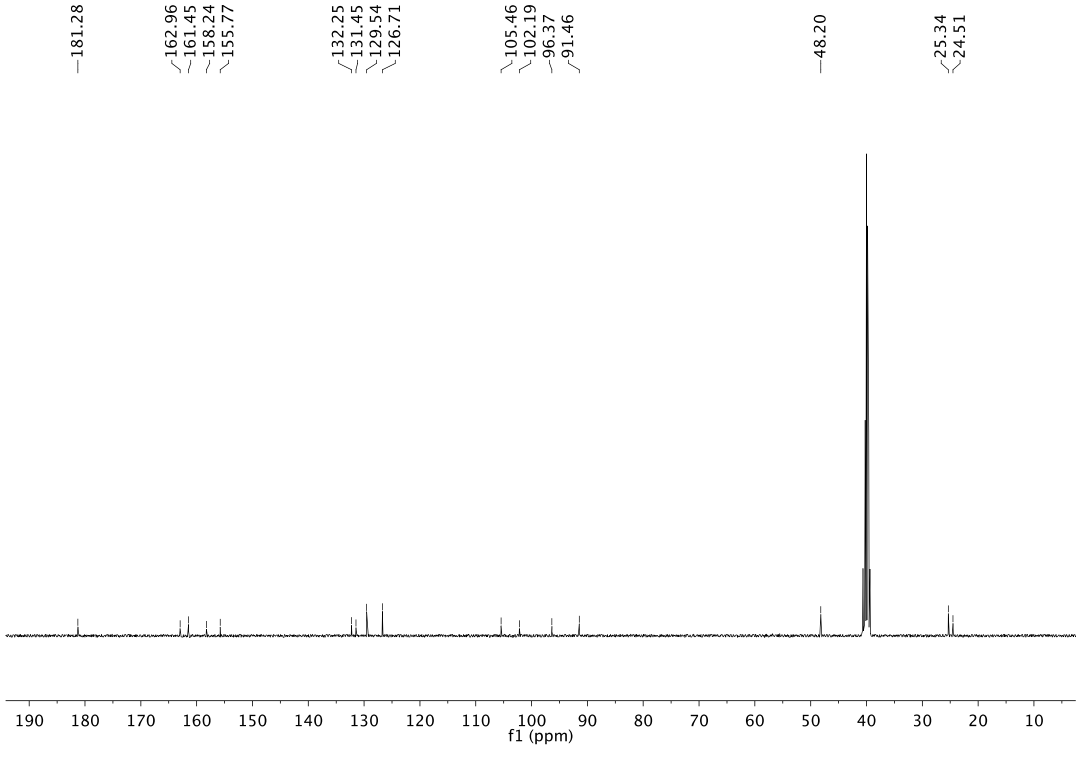
181.28
162.96 161.45 158.24 155.77
132.25 131.45 129.54 126.71
105.46 102.19 96.37 91.46
48.20
25.34 24.51
f1 (ppm)

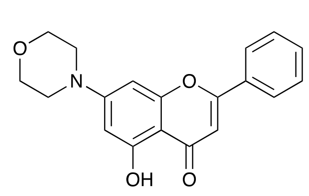
O
N
O
OH
O

39

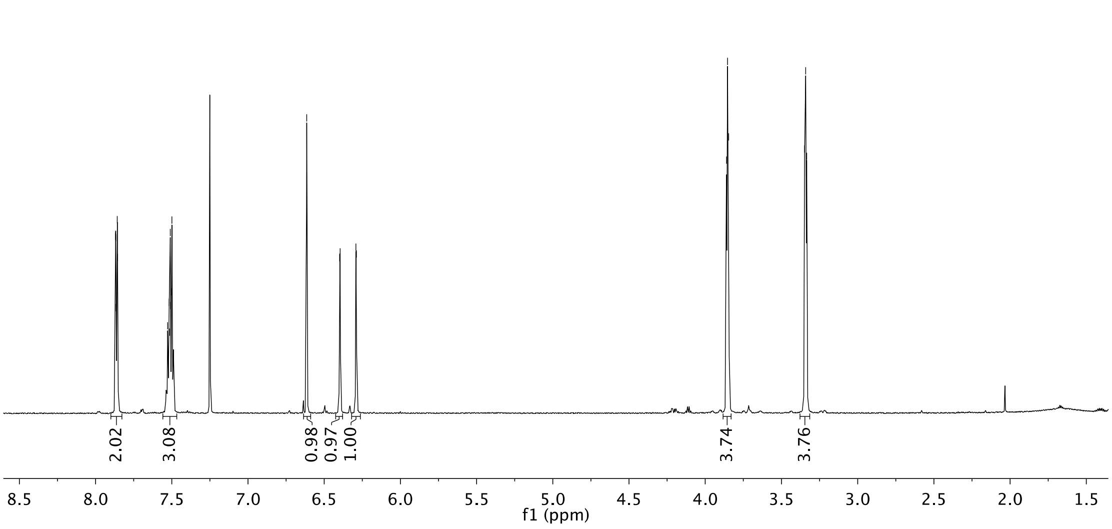
2.02
3.08
0.98
0.97
1.00
3.74
3.76
8.5 8.0 7.5 7.0 6.5 6.0 5.5 5.0 4.5 4.0 3.5 3.0 2.5 2.0 1.5
f1 (ppm)

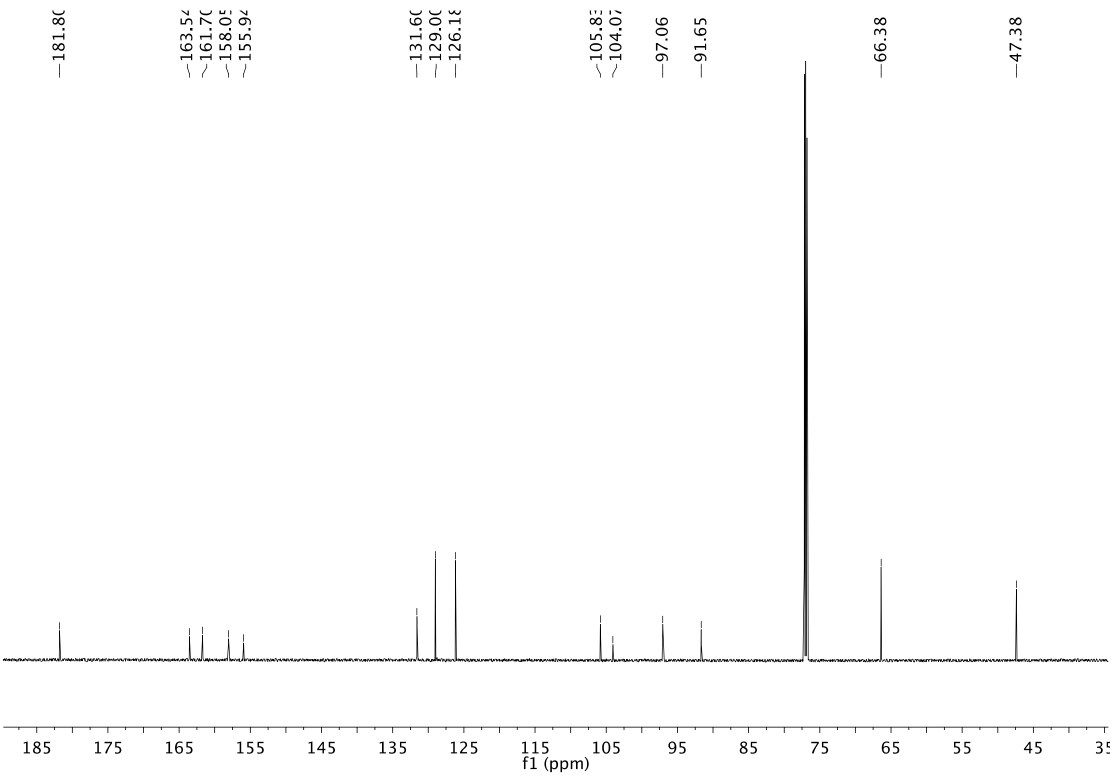
181.80
163.54
161.70
158.05
155.94
131.60
129.00
126.18
105.83
104.07
97.06
91.65
66.38
47.38
185 175 165 155 145 135 125 115 105 95 85 75 65 55 45
f1 (ppm)

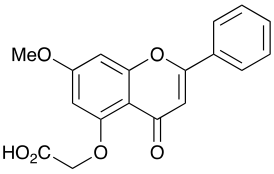
MeO
O
HO2C
O
O

40

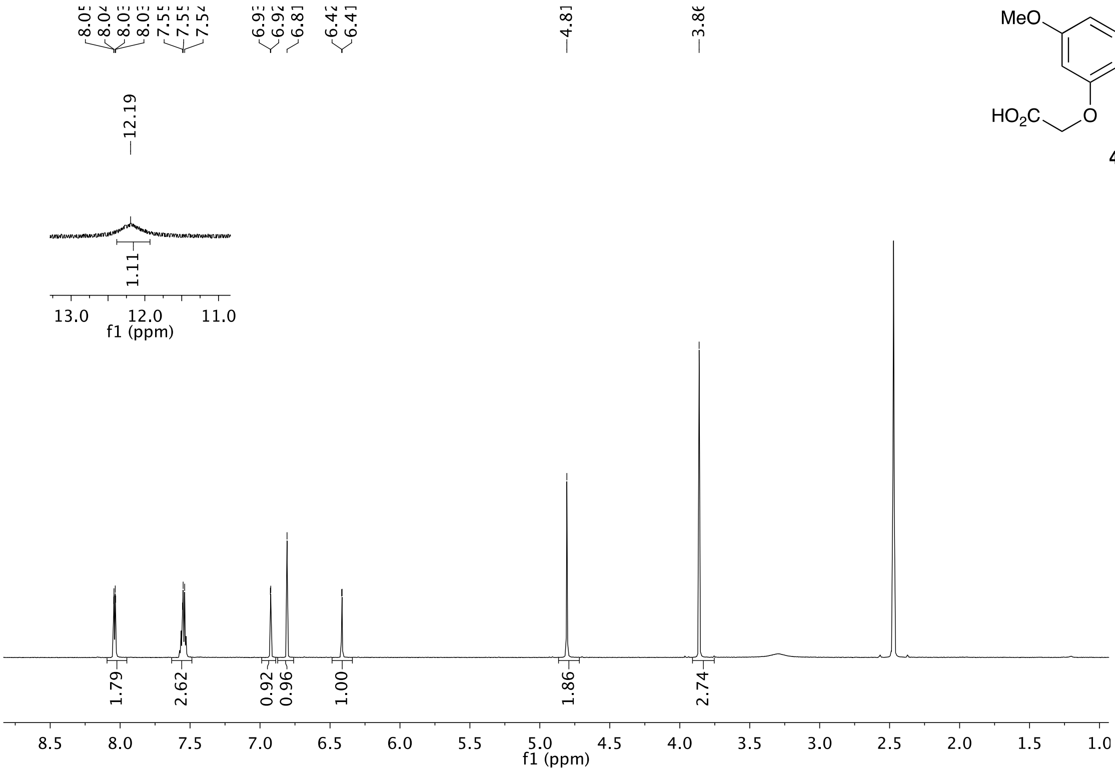
12.19
4.81
1.11
1.79
2.62
0.92
0.96
1.00
1.86
2.74
f1 (ppm)

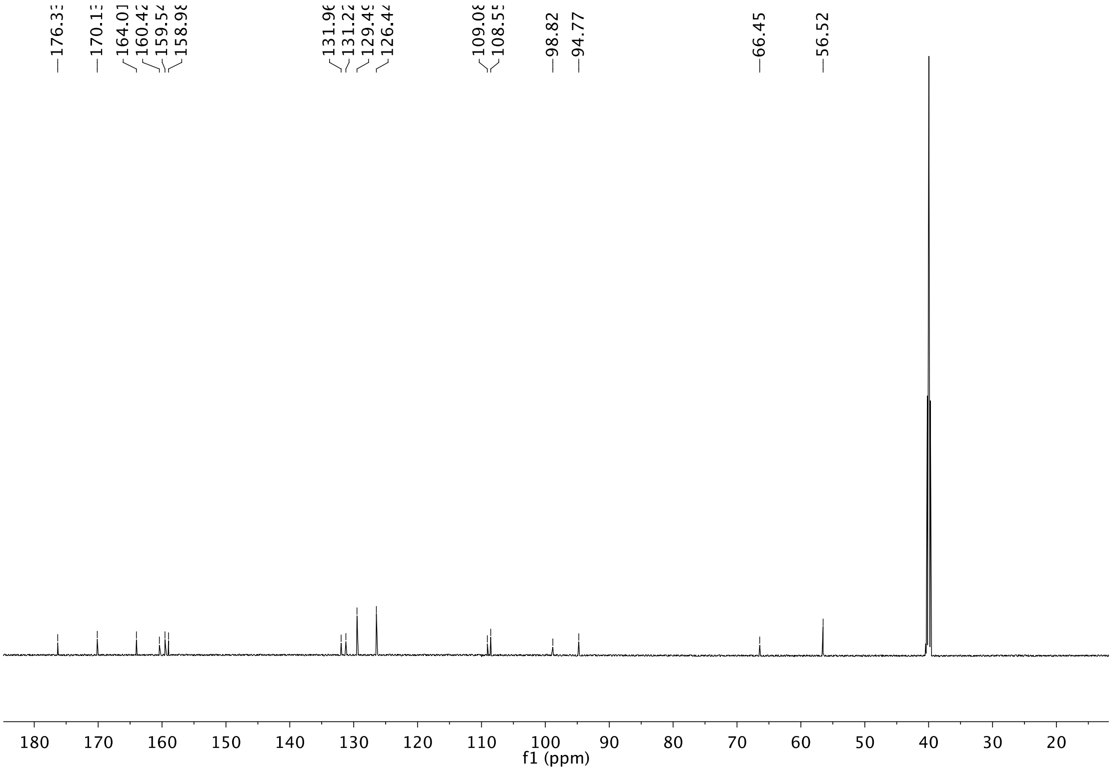
164.01
160.42
159.54
158.98
131.22
129.49
126.44
108.55
98.82
94.77
66.45
56.52
f1 (ppm)

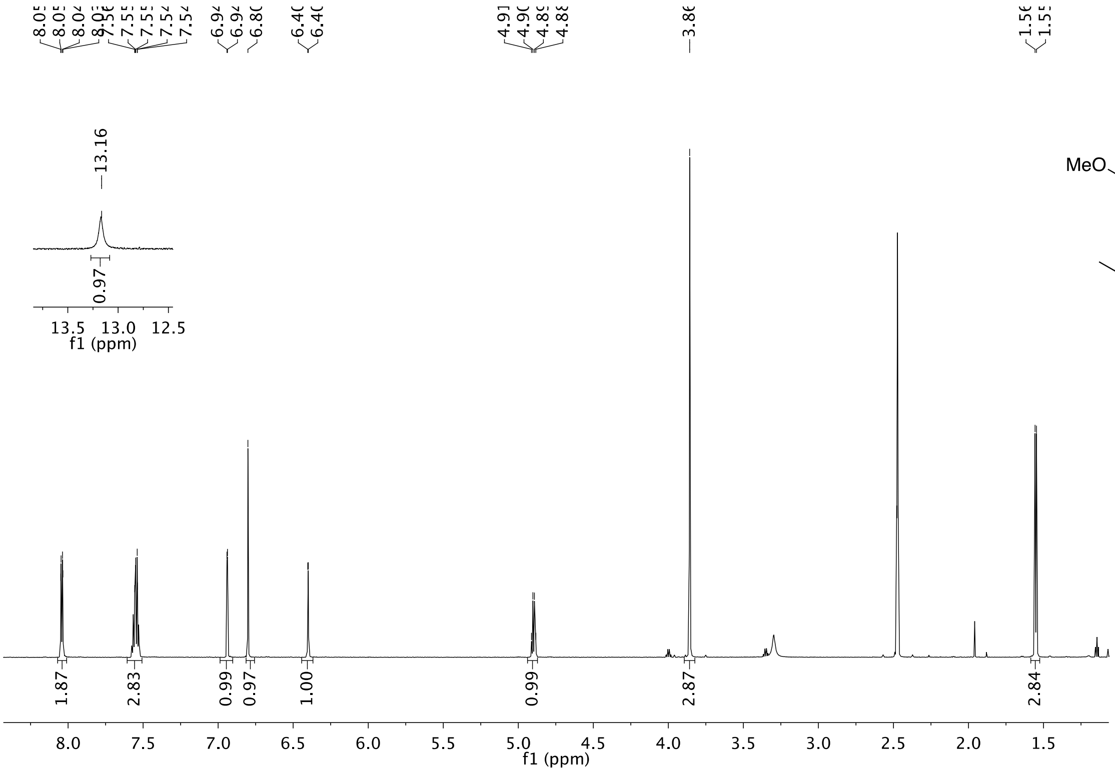
8.05
8.05
8.04
8.03
7.56
7.55
7.55
7.54
7.54
6.94
6.94
6.80
6.40
6.40
4.91
4.90
4.89
4.88
3.86
1.56
1.55
13.16
0.97
13.5 13.0 12.5
f1 (ppm)
1.87
2.83
0.99
0.97
1.00
0.99
2.87
2.84
8.0 7.5 7.0 6.5 6.0 5.5 5.0 4.5 4.0 3.5 3.0 2.5 2.0 1.5
f1 (ppm)

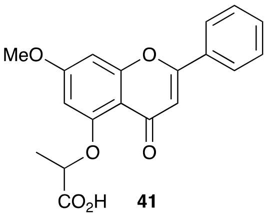
MeO
O
O
O
CO_2H

41

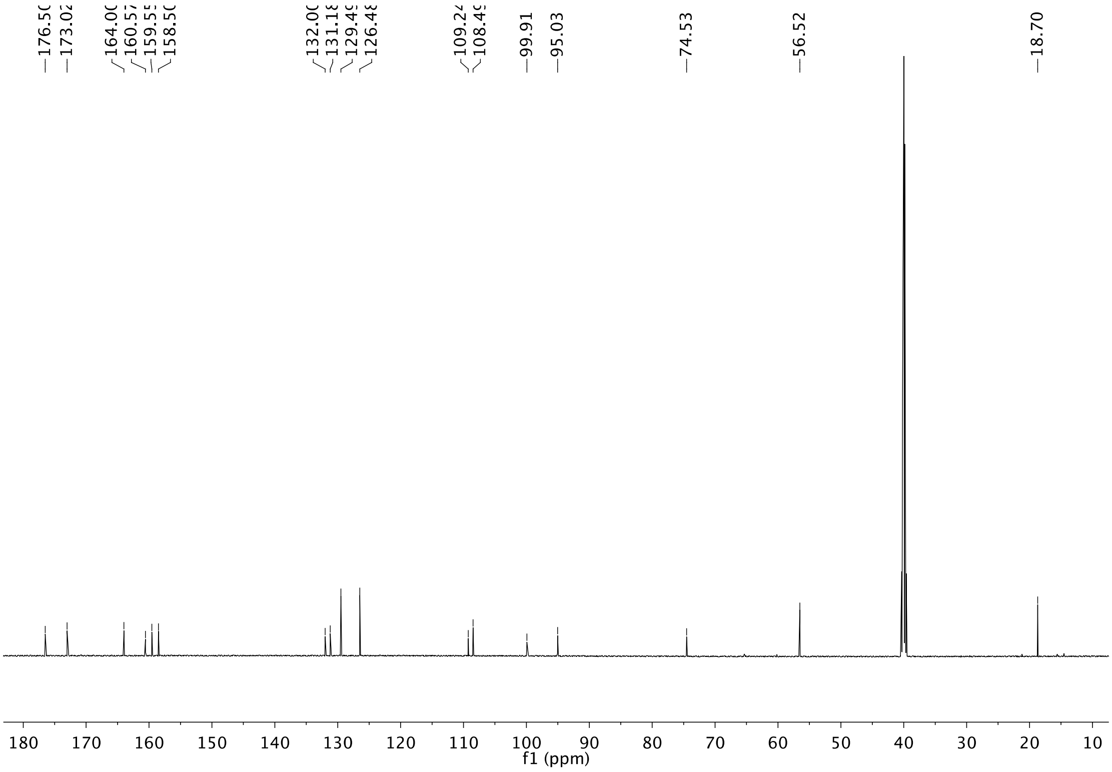
176.50
173.02
164.00
160.57
159.55
158.50
132.00
131.18
129.49
126.48
109.24
108.49
99.91
95.03
74.53
56.52
18.70
180 170 160 150 140 130 120 110 100 90 80 70 60 50 40 30 20 10
f1 (ppm)

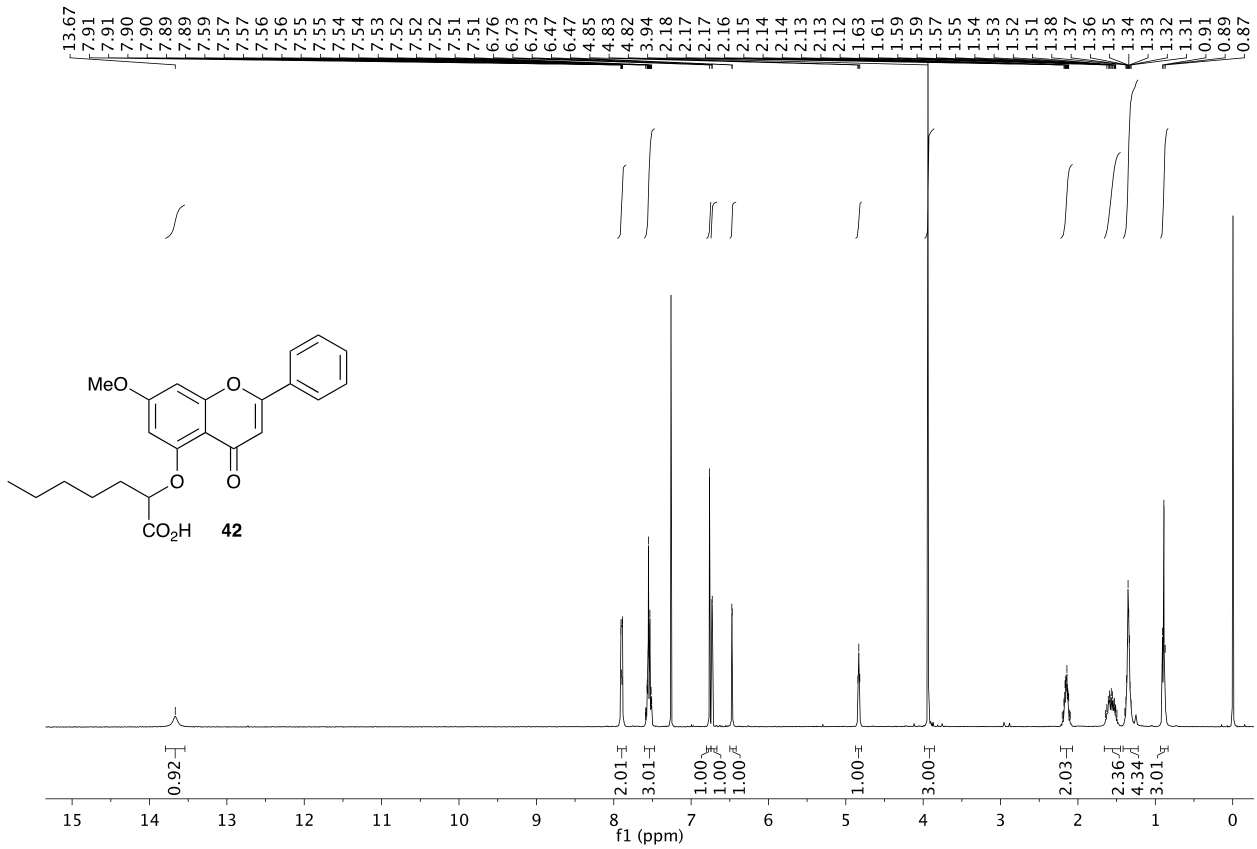

MeO
O
O
CO2H
42
13.67 7.91 7.91 7.90 7.90 7.89 7.89 7.59 7.57 7.57 7.56 7.56 7.55 7.55 7.54 7.54 7.53 7.52 7.52 7.52 7.51 7.51 6.76 6.73 6.47 6.47 4.85 4.83 4.82 3.94 2.18 2.17 2.17 2.16 2.15 2.15 2.14 2.14 2.13 2.13 2.12 1.63 1.61 1.59 1.59 1.57 1.55 1.54 1.53 1.53 1.52 1.51 1.38 1.37 1.36 1.35 1.34 1.33 1.32 1.31 0.91 0.89 0.87
0.92
2.01
3.01
1.00
1.00
1.00
1.00
3.00
2.03
2.36
4.34
3.01
15 14 13 12 11 10 9 8 7 6 5 4 3 2 1 0
f1 (ppm)

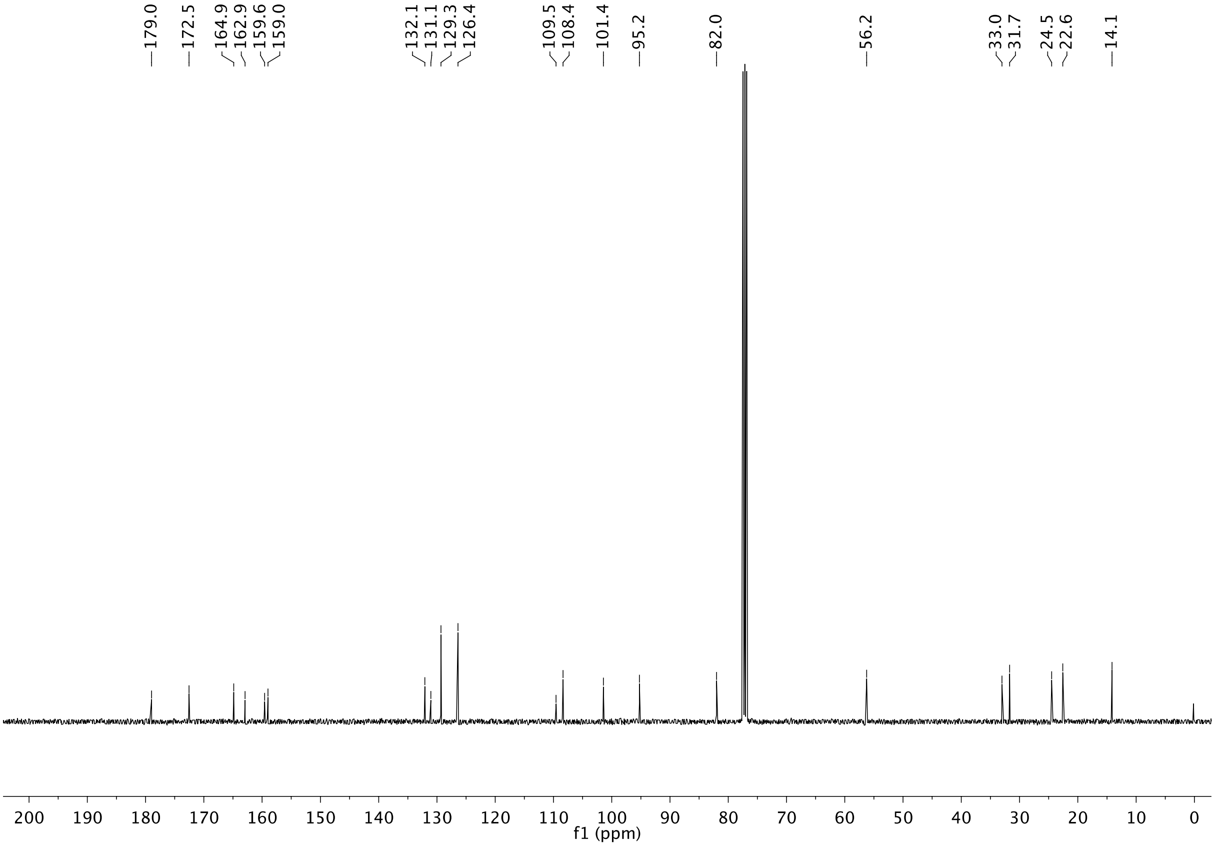

179.0 172.5 164.9 162.9 159.6 159.0 132.1 131.1 129.3 126.4 109.5 108.4 101.4 95.2 82.0 56.2 33.0 31.7 24.5 22.6 14.1
200 190 180 170 160 150 140 130 120 110 100 90 80 70 60 50 40 30 20 10 0
f1 (ppm)

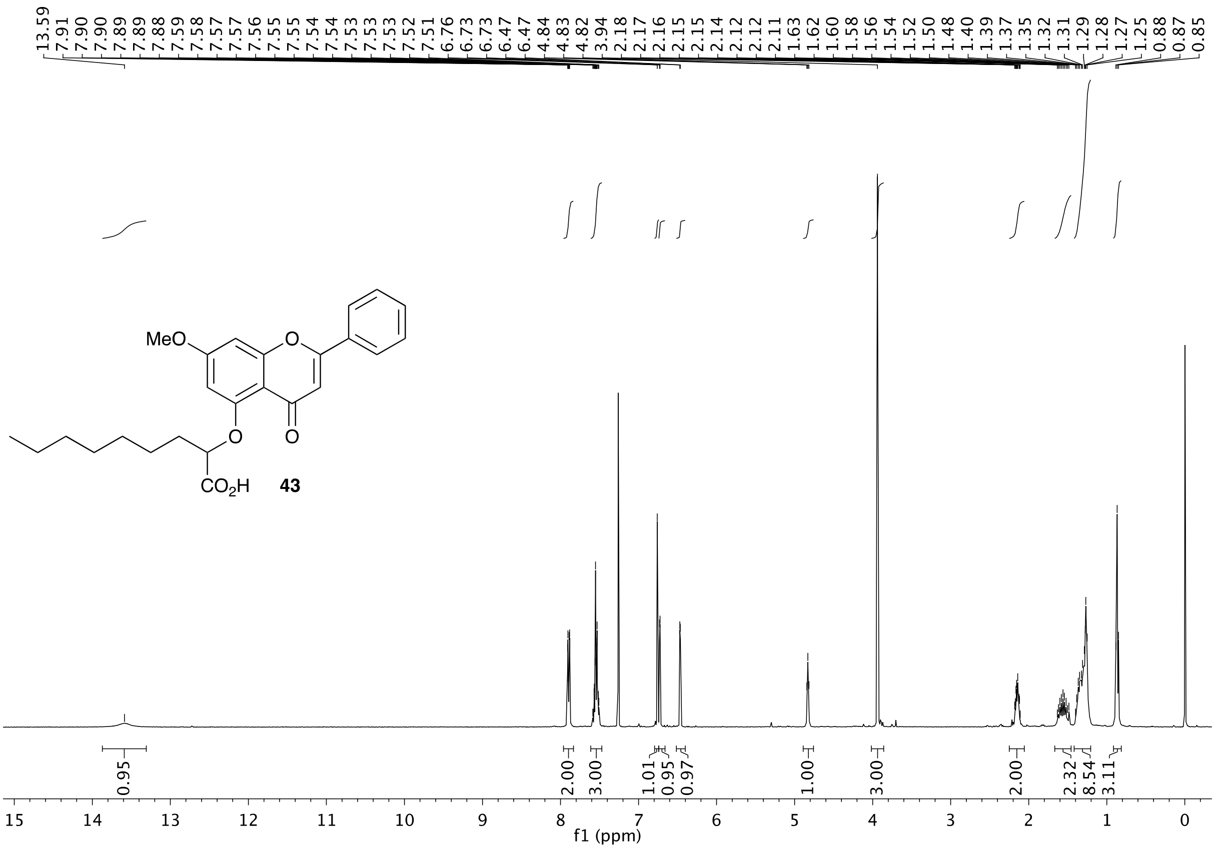
13.59 7.91 7.90 7.90 7.89 7.89 7.88 7.59 7.58 7.57 7.57 7.56 7.55 7.55 7.54 7.54 7.53 7.53 7.53 7.52 7.52 7.51 6.76 6.73 6.47 6.47 4.84 4.83 4.82 3.94 2.18 2.17 2.16 2.15 2.15 2.14 2.12 2.12 2.11 1.63 1.62 1.60 1.58 1.56 1.54 1.52 1.50 1.48 1.40 1.39 1.37 1.35 1.32 1.31 1.29 1.28 1.27 1.25 0.88 0.87 0.85
MeO
O
O
O
CO2H
43
0.95
2.00
3.00
1.01
0.95
0.97
1.00
3.00
2.00
2.32
8.54
3.11
15 14 13 12 11 10 9 8 7 6 5 4 3 2 1 0
f1 (ppm)

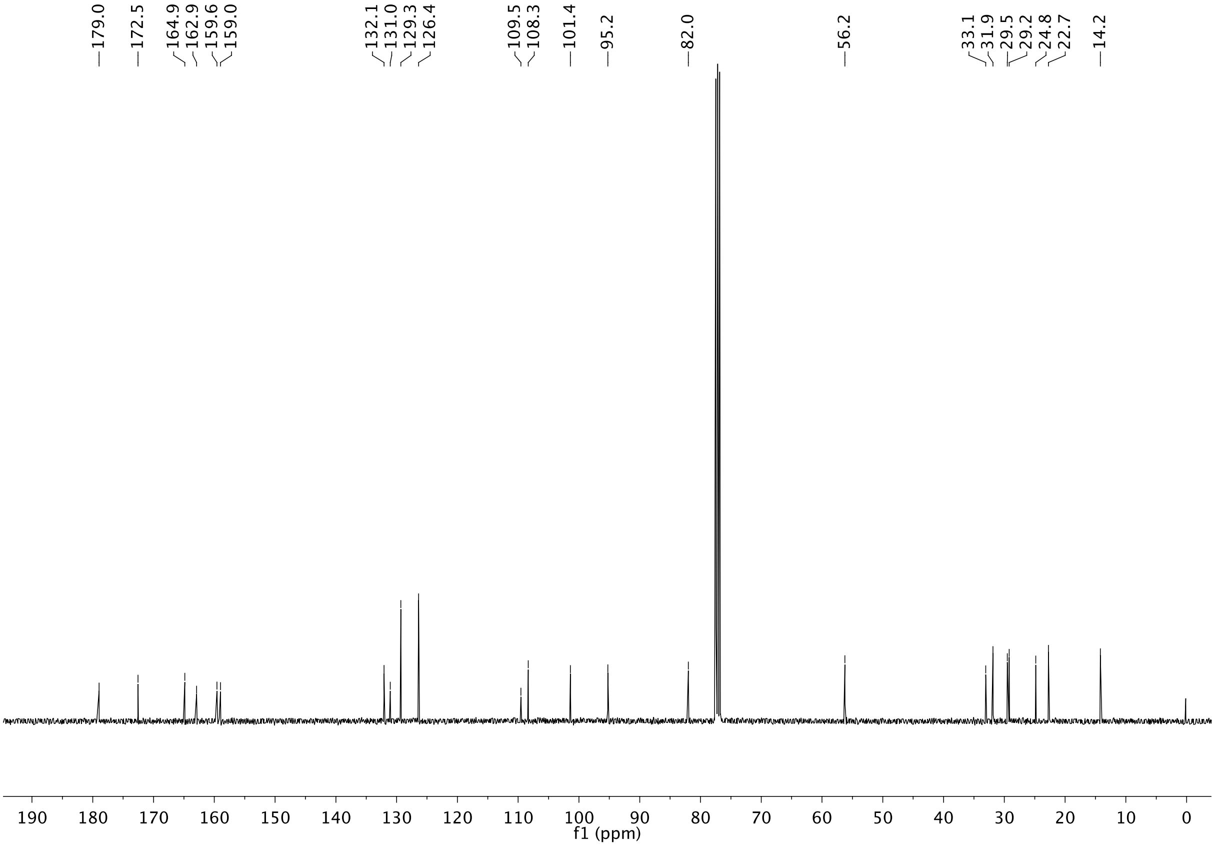
179.0
172.5
164.9 162.9 159.6 159.0
132.1 131.0 129.3 126.4
109.5 108.3
101.4
95.2
82.0
56.2
33.1 31.9 29.5 29.2 24.8 22.7
14.2
190 180 170 160 150 140 130 120 110 100 90 80 70 60 50 40 30 20 10 0
f1 (ppm)

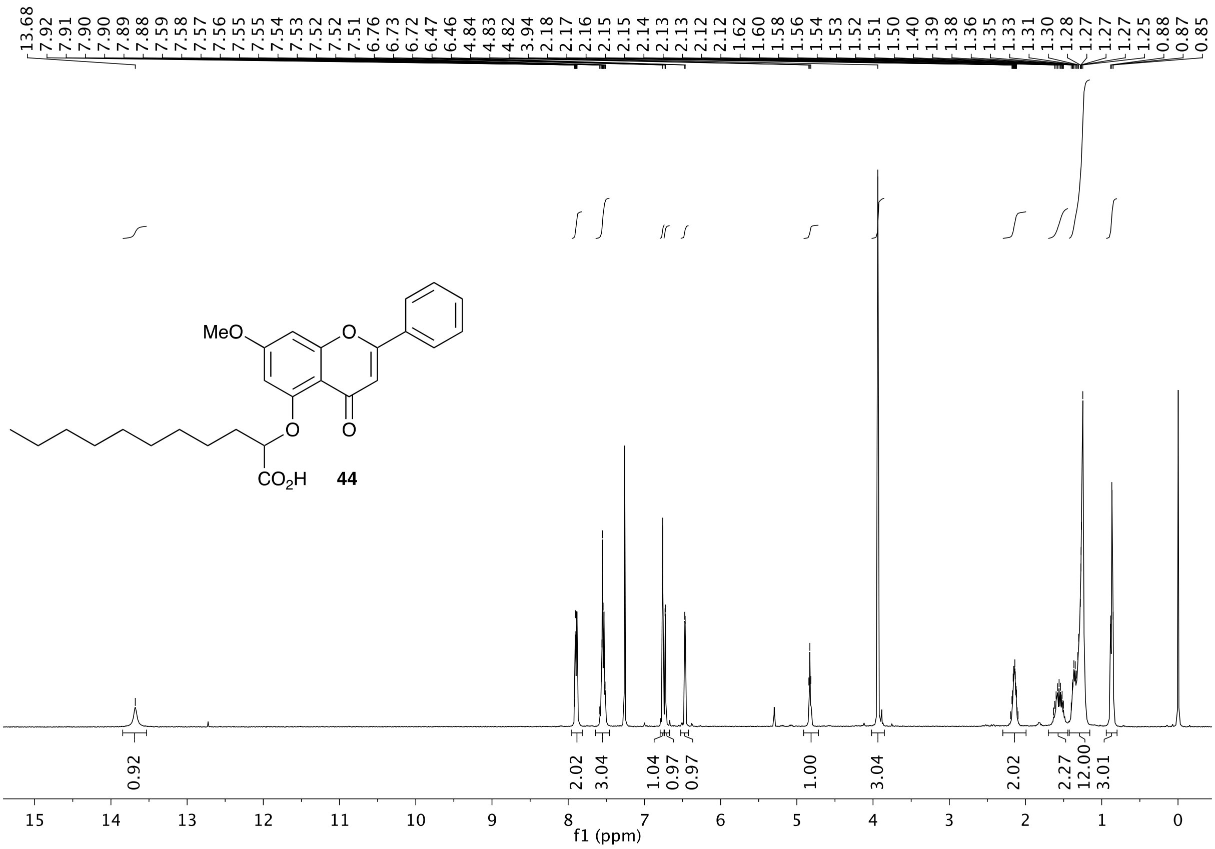
13.68 7.92 7.91 7.90 7.90 7.89 7.88 7.59 7.58 7.57 7.56 7.55 7.55 7.54 7.53 7.52 7.52 7.51 6.76 6.73 6.72 6.47 6.46 4.84 4.83 4.82 3.94 2.18 2.17 2.16 2.15 2.15 2.14 2.13 2.13 2.12 2.12 1.62 1.60 1.58 1.56 1.54 1.53 1.52 1.51 1.50 1.40 1.39 1.38 1.36 1.35 1.33 1.31 1.30 1.28 1.27 1.27 1.27 1.25 0.88 0.87 0.85
MeO
O
O
O
CO_2H
44
0.92 2.02 3.04 1.04 0.97 0.97 1.00 3.04 2.02 2.27 12.00 3.01
15 14 13 12 11 10 9 8 7 6 5 4 3 2 1 0
f1 (ppm)

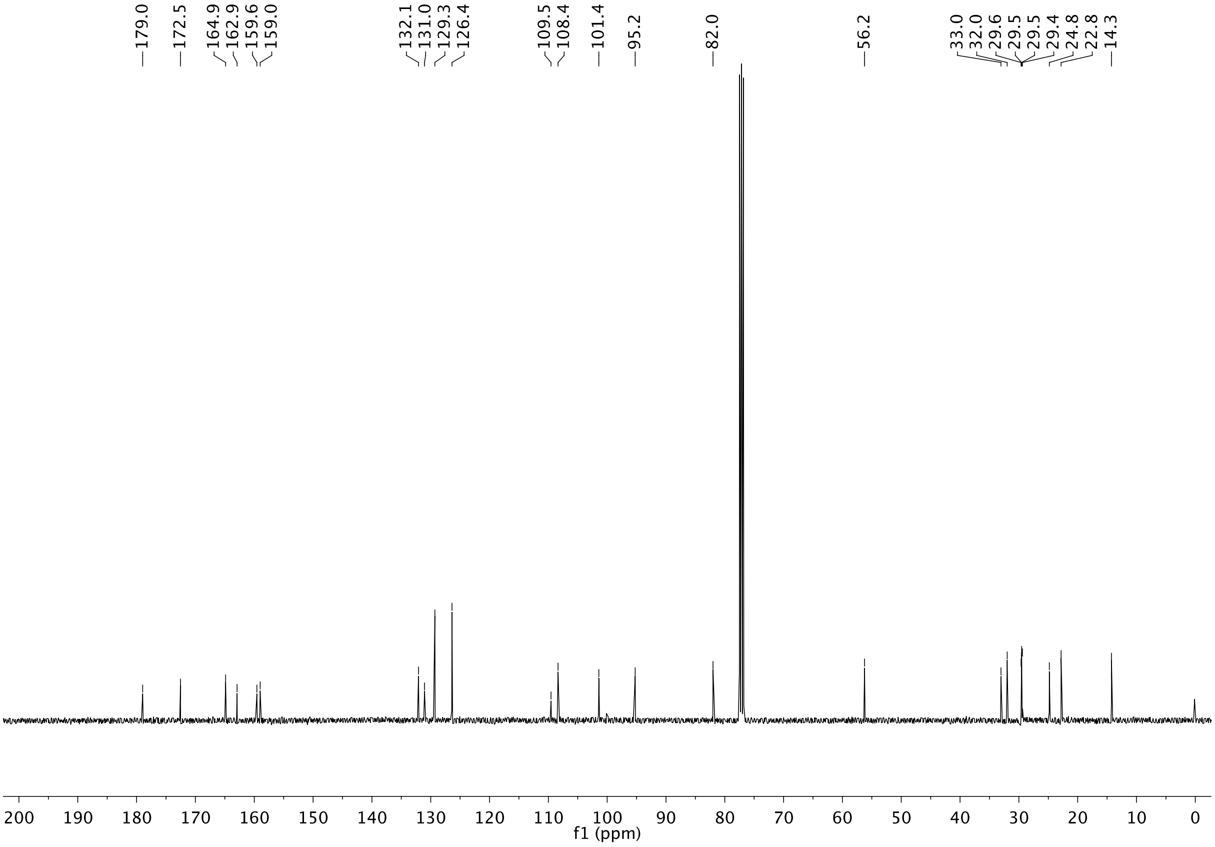
179.0 172.5 164.9 162.9 159.6 159.0 132.1 131.0 129.3 126.4 109.5 108.4 101.4 95.2 82.0 56.2 33.0 32.0 29.6 29.5 29.5 29.4 24.8 22.8 14.3
200 190 180 170 160 150 140 130 120 110 100 90 80 70 60 50 40 30 20 10 0
f1 (ppm)

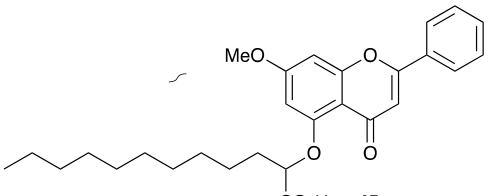
MeO
O
O
O
CO2H

45

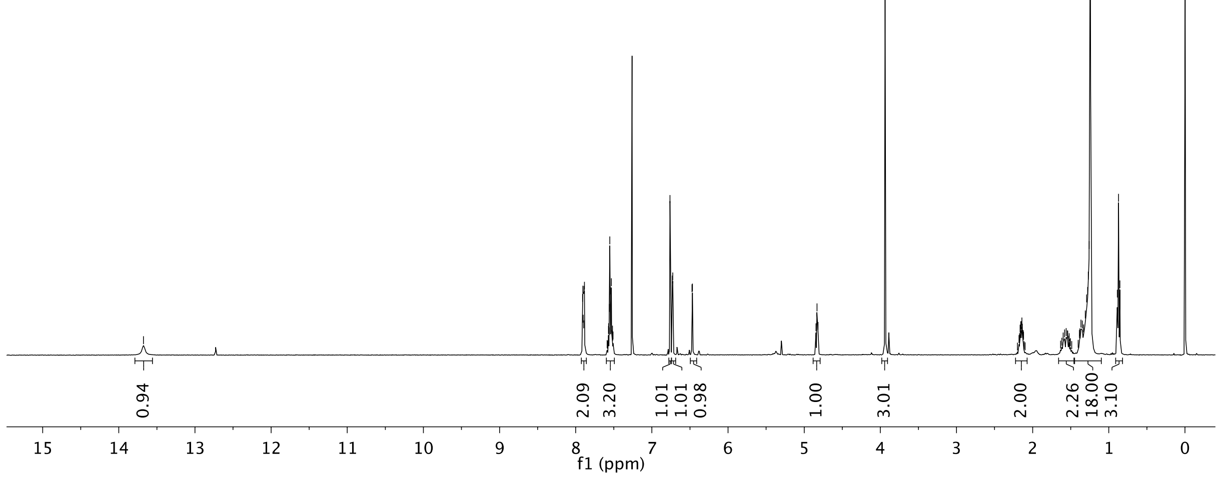
13.68 7.91 7.91 7.90 7.90 7.89 7.89 7.57 7.57 7.56 7.55 7.55 7.54 7.54 7.53 7.52 7.52 7.51 7.51 6.76 6.73 6.73 6.47 4.85 4.83 4.82 3.94 2.18 2.17 2.16 2.16 2.15 2.14 2.14 2.13 2.12 2.12 1.62 1.60 1.58 1.56 1.54 1.54 1.53 1.52 1.39 1.38 1.37 1.35 1.33 1.31 1.29 1.28 1.27 1.26 1.24 0.89 0.87 0.85
0.94
2.09
3.20
1.01
1.01
0.98
1.00
3.01
2.00
2.26
18.00
3.10
15 14 13 12 11 10 9 8 7 6 5 4 3 2 1 0
f1 (ppm)

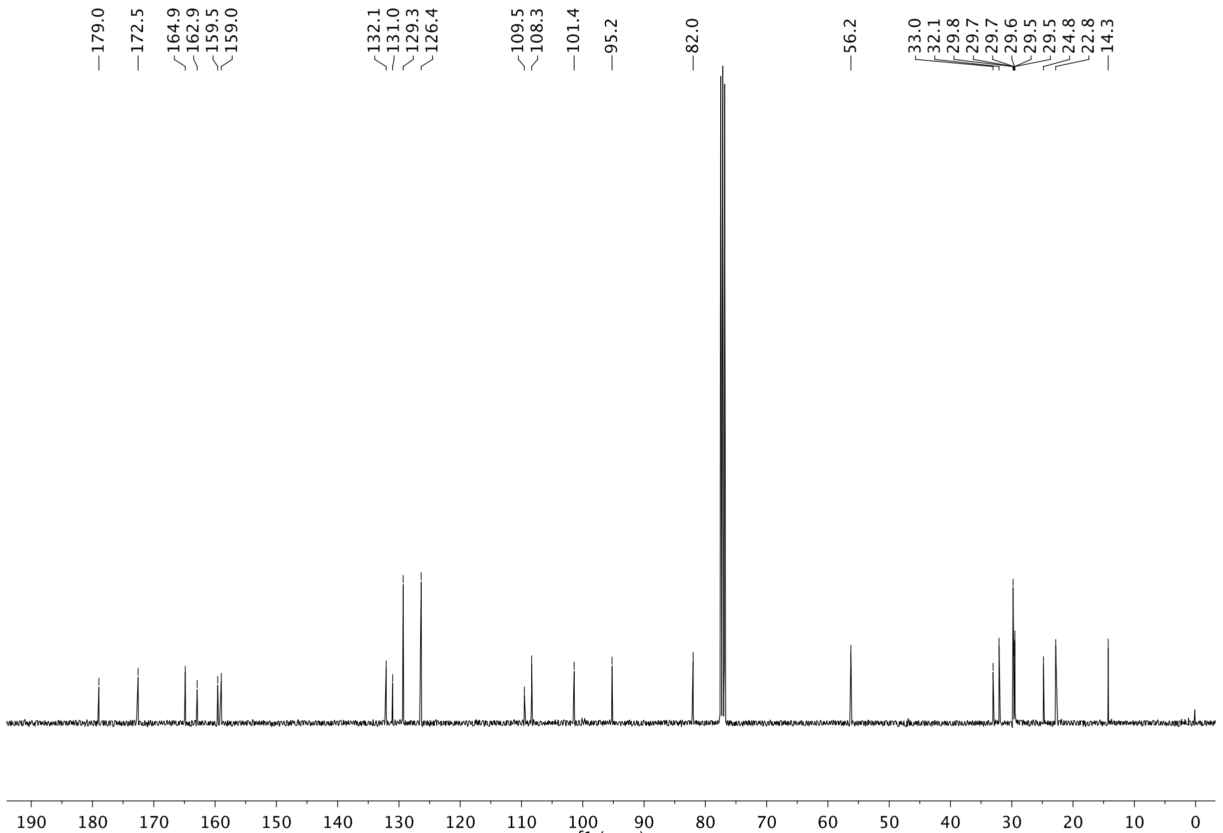
179.0
172.5
164.9 162.9 159.5 159.0
132.1 131.0 129.3 126.4
109.5 108.3
101.4
95.2
82.0
56.2
33.0 32.1 29.8 29.7 29.7 29.6 29.5 29.5 24.8 22.8
14.3
190 180 170 160 150 140 130 120 110 100 90 80 70 60 50 40 30 20 10 0
f1 (ppm)

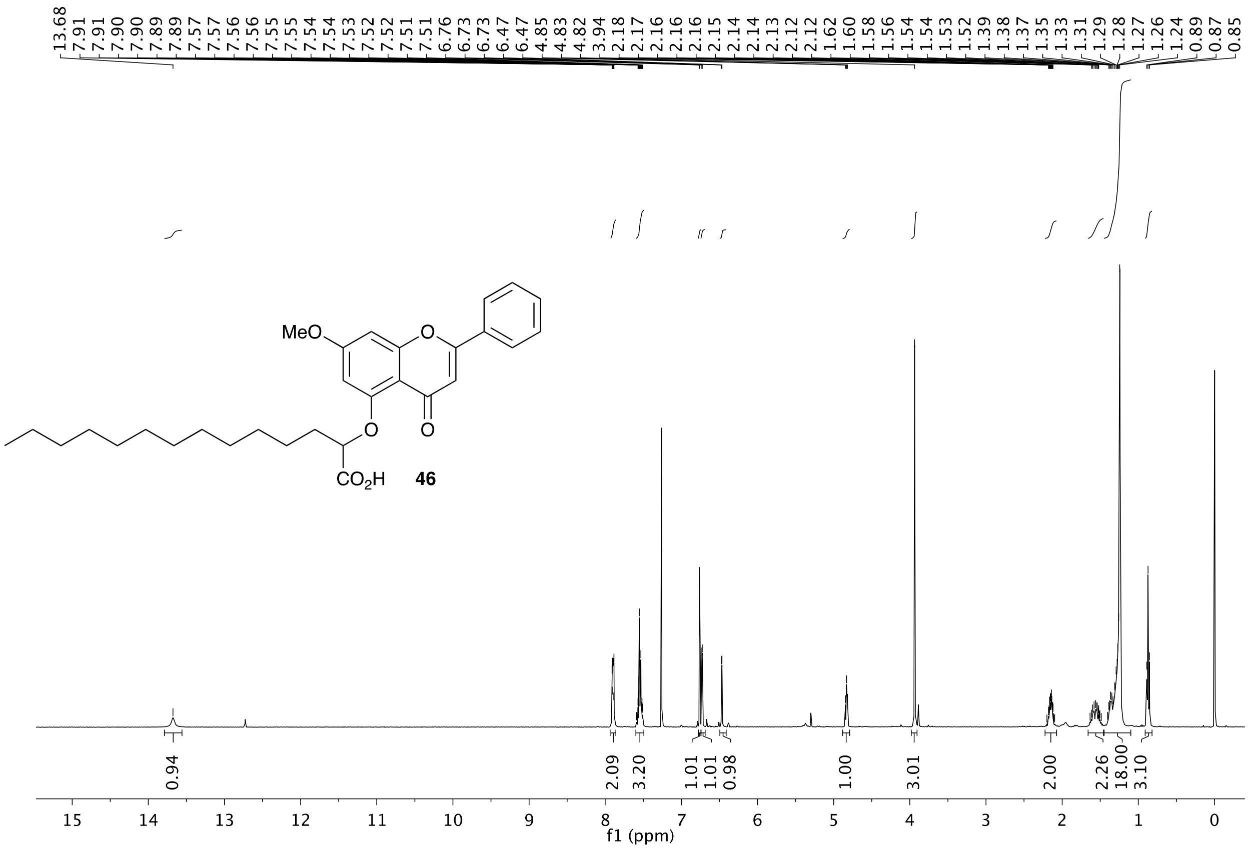
13.68 7.91 7.91 7.90 7.90 7.89 7.89 7.57 7.57 7.56 7.55 7.55 7.54 7.54 7.53 7.52 7.52 7.51 7.51 6.76 6.73 6.73 6.47 6.47 4.85 4.83 4.82 3.94 2.18 2.17 2.16 2.16 2.15 2.14 2.14 2.13 2.12 2.12 1.62 1.60 1.58 1.56 1.54 1.54 1.53 1.52 1.39 1.38 1.37 1.35 1.33 1.31 1.29 1.28 1.27 1.26 1.24 0.89 0.87 0.85
MeO
O
O
O
CO_2H
46
0.94 2.09 3.20 1.01 1.01 0.98 1.00 3.01 2.00 2.26 18.00 3.10
15 14 13 12 11 10 9 8 7 6 5 4 3 2 1 0
f1 (ppm)

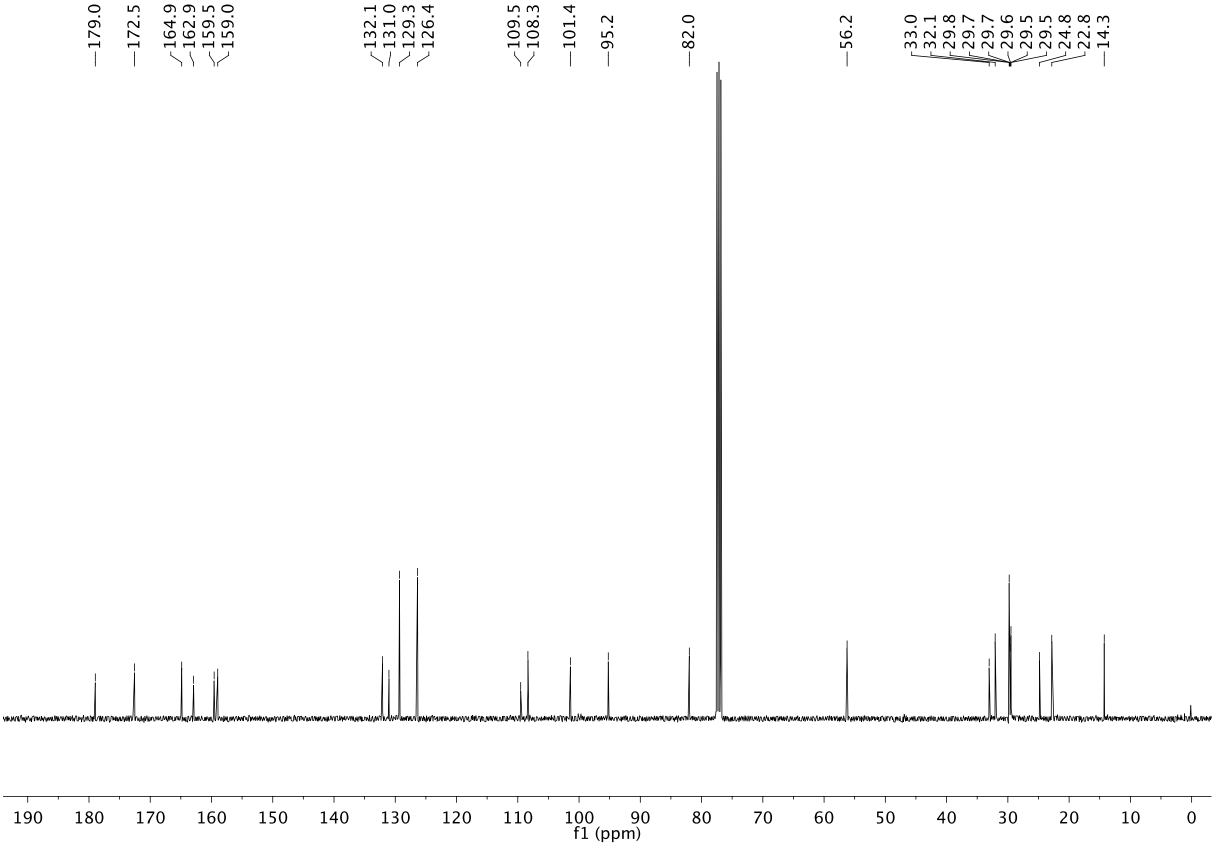
179.0 172.5 164.9 162.9 159.5 159.0 132.1 131.0 129.3 126.4 109.5 108.3 101.4 95.2 82.0 56.2 33.0 32.1 29.8 29.7 29.7 29.6 29.5 29.5 24.8 22.8 14.3
190 180 170 160 150 140 130 120 110 100 90 80 70 60 50 40 30 20 10 0
f1 (ppm)

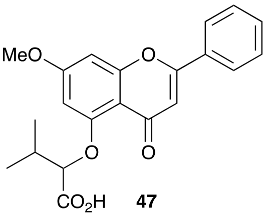

MeO
O
O
O
CO2H

47

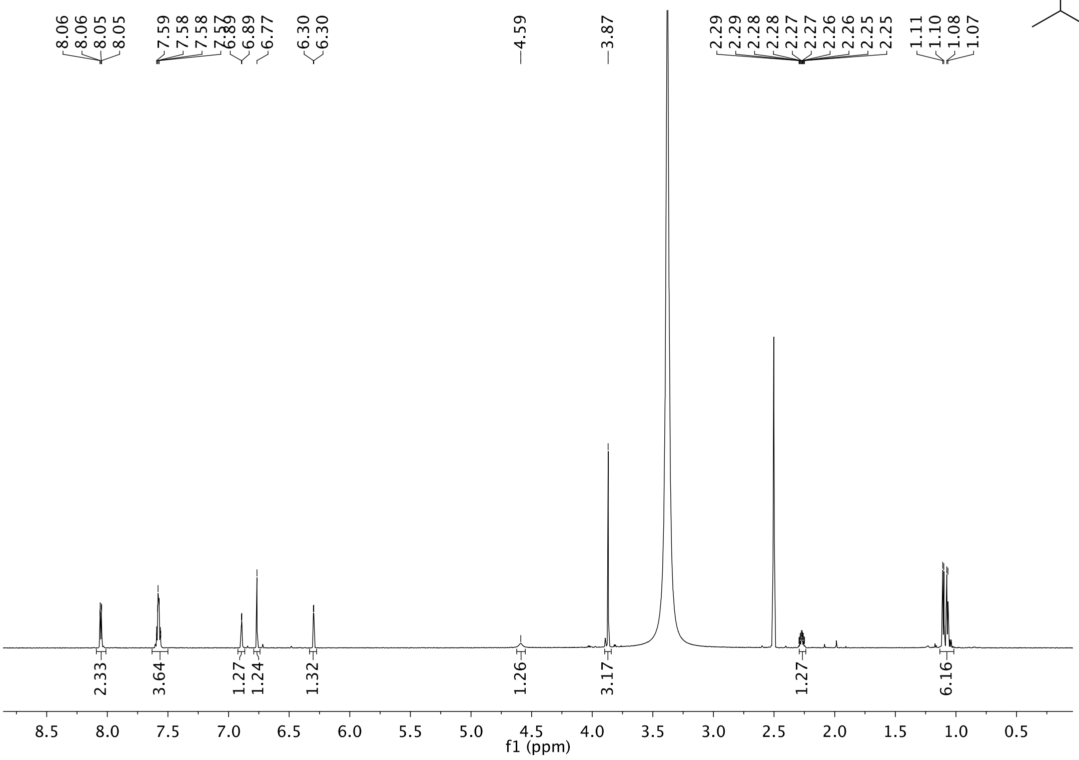

8.06
8.06
8.05
8.05
7.59
7.58
7.58
7.57
6.89
6.89
6.77
6.30
6.30
4.59
3.87
2.29
2.29
2.28
2.28
2.27
2.27
2.26
2.26
2.25
2.25
1.11
1.10
1.08
1.07
2.33
3.64
1.27
1.24
1.32
1.26
3.17
1.27
6.16
8.5 8.0 7.5 7.0 6.5 6.0 5.5 5.0 4.5 4.0 3.5 3.0 2.5 2.0 1.5 1.0 0.5
f1 (ppm)

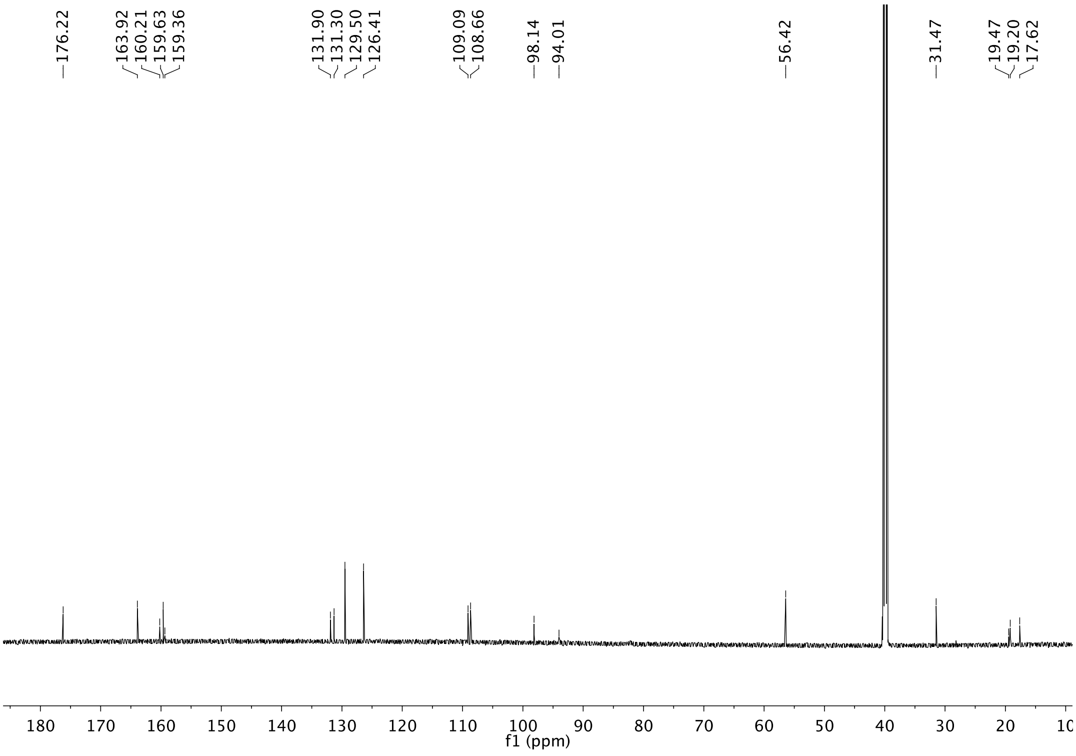

176.22
163.92
160.21
159.63
159.36
131.90
131.30
129.50
126.41
109.09
108.66
98.14
94.01
56.42
31.47
19.47
19.20
17.62
180 170 160 150 140 130 120 110 100 90 80 70 60 50 40 30 20 10
f1 (ppm)

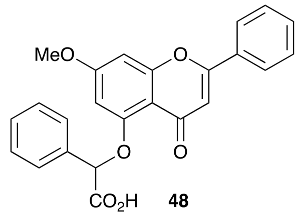
MeO
O
O
O
CO2H
48

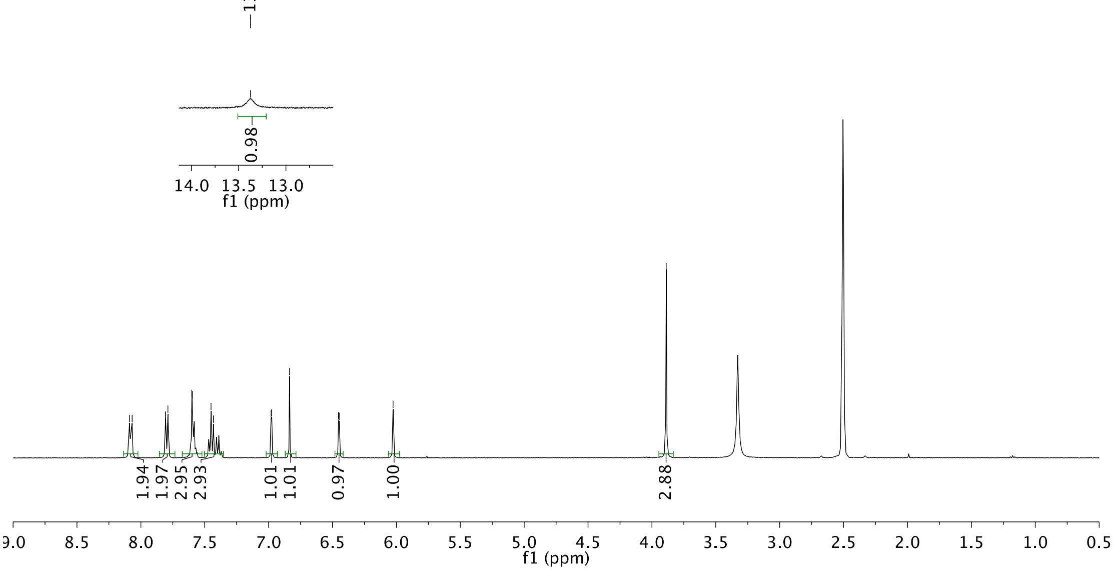
8.09
8.07
7.81
7.79
7.60
7.60
7.45
7.43
6.98
6.98
6.84
6.45
6.45
6.03
3.89
13.37
0.98
14.0 13.5 13.0
f1 (ppm)
1.94
1.97
2.95
2.93
1.01
1.01
0.97
1.00
2.88
9.0 8.5 8.0 7.5 7.0 6.5 6.0 5.5 5.0 4.5 4.0 3.5 3.0 2.5 2.0 1.5 1.0 0.5
f1 (ppm)

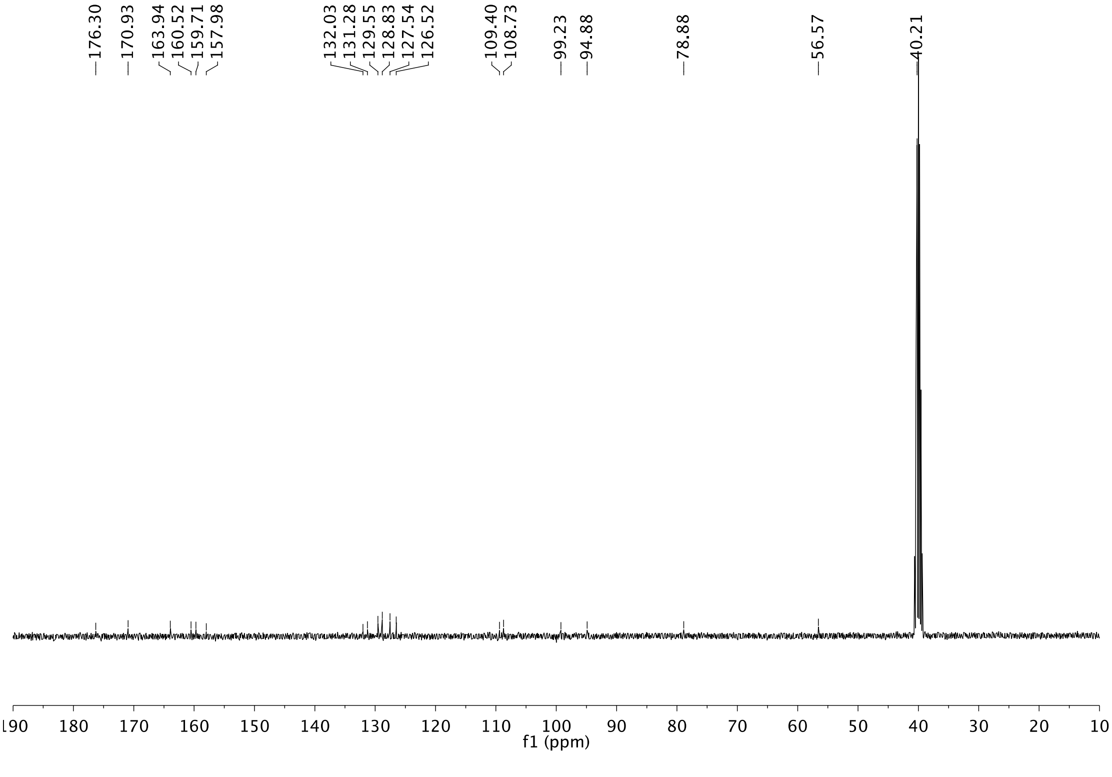
176.30
170.93
163.94
160.52
159.71
157.98
132.03
131.28
129.55
128.83
127.54
126.52
109.40
108.73
99.23
94.88
78.88
56.57
40.21
190 180 170 160 150 140 130 120 110 100 90 80 70 60 50 40 30 20 10
f1 (ppm)

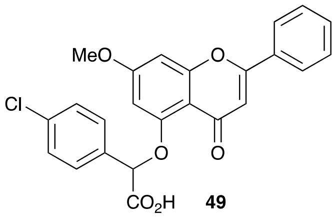
MeO
O
Cl
O
O
CO2H

49

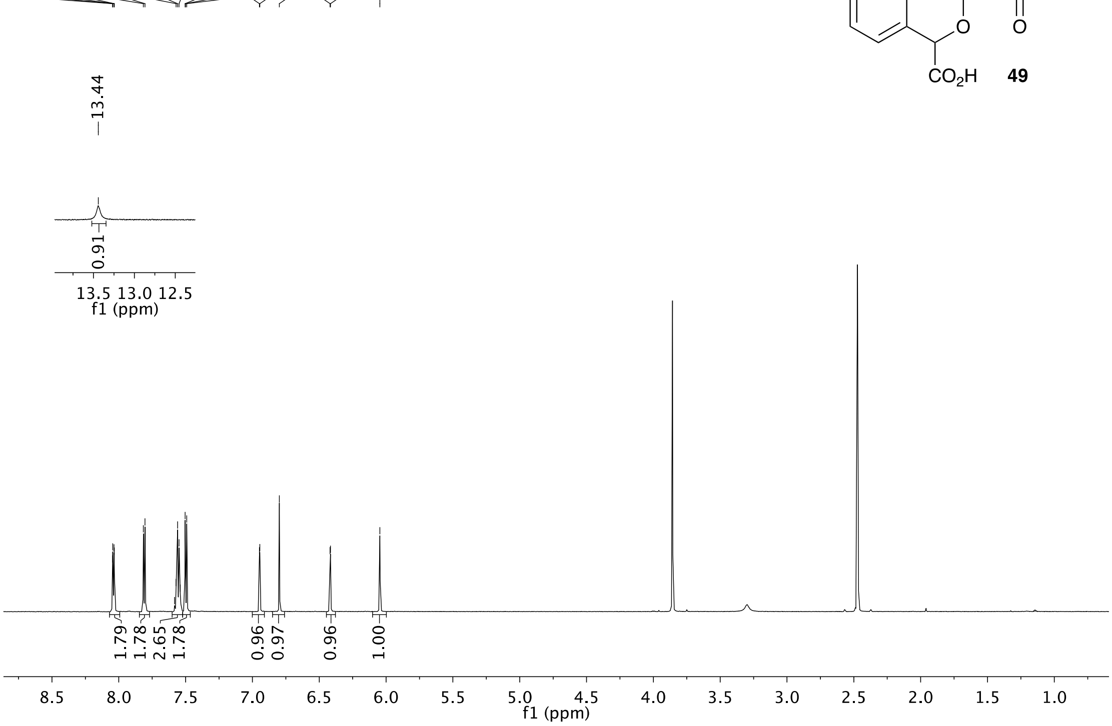
8.05
8.05
8.04
8.03
7.82
7.80
7.57
7.56
7.55
7.50
7.50
7.49
6.95
6.94
6.80
6.42
6.42
6.05
13.44
0.91
13.5 13.0 12.5
f1 (ppm)
1.79
1.78
2.65
1.78
0.96
0.97
0.96
1.00
8.5
8.0
7.5
7.0
6.5
6.0
5.5
5.0
4.5
4.0
3.5
3.0
2.5
2.0
1.5
1.0
f1 (ppm)

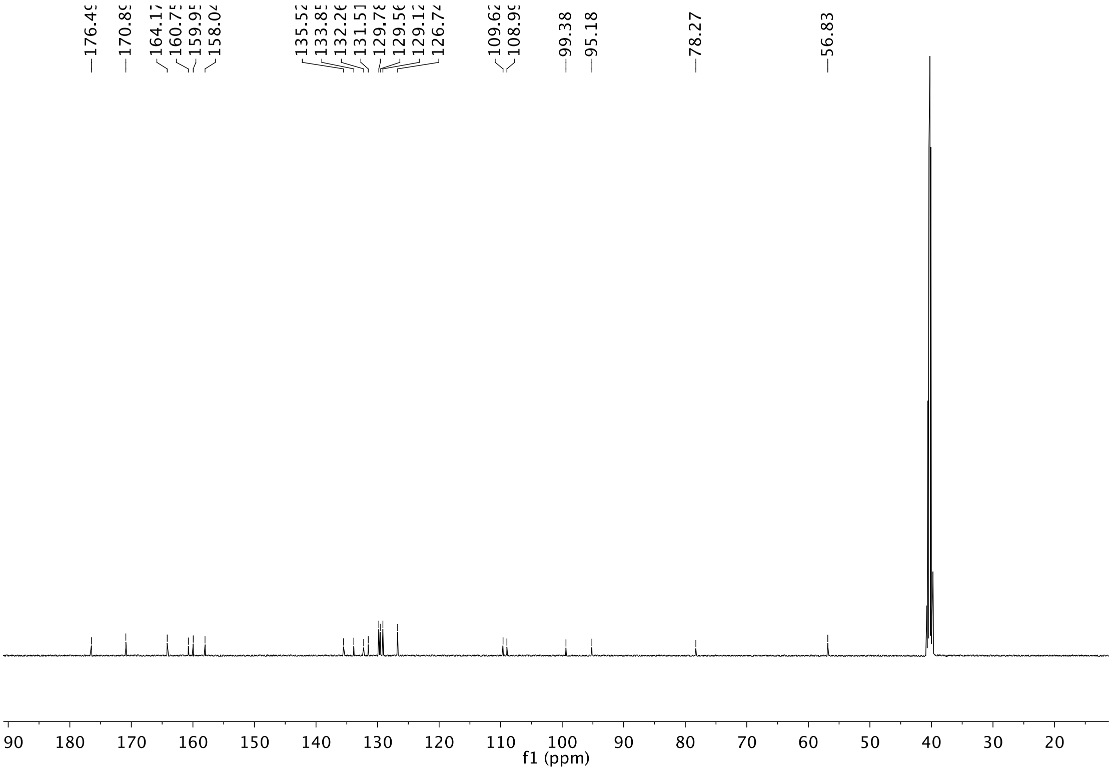
176.49
170.89
164.17
160.75
159.95
158.04
135.52
133.85
132.26
131.51
129.78
129.56
129.12
126.74
109.62
108.99
99.38
95.18
78.27
56.83
90
180
170
160
150
140
130
120
110
100
90
80
70
60
50
40
30
20
f1 (ppm)

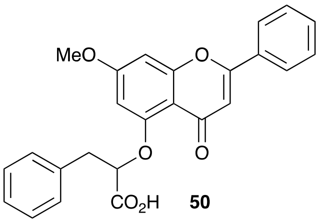
MeO
O
O
O
CO_2H

50

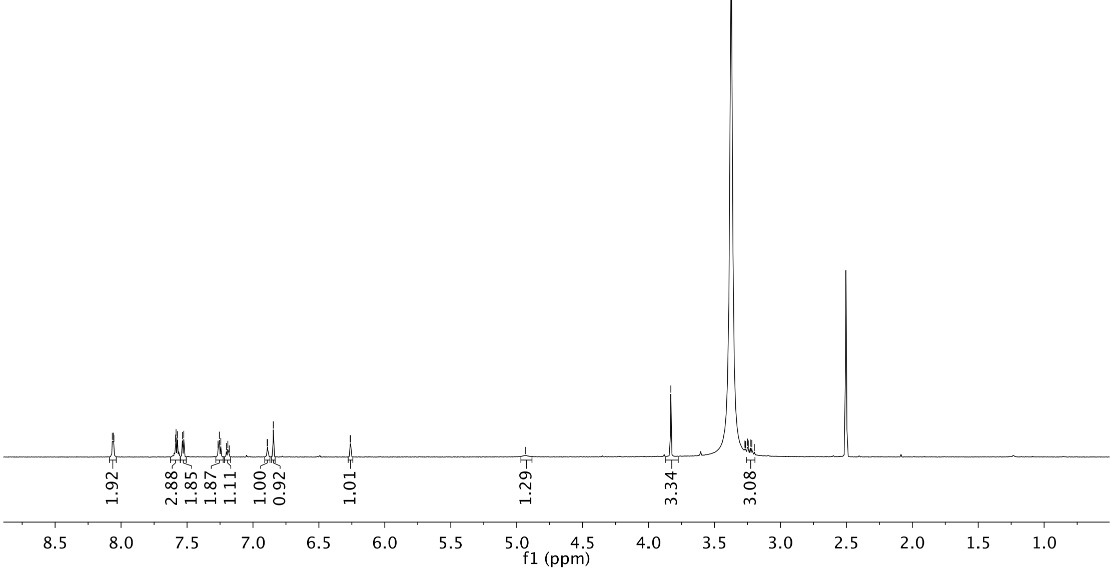
4.93
3.83
1.92
2.88
1.85
1.87
1.11
1.00
0.92
1.01
1.29
3.34
3.08
8.5
8.0
7.5
7.0
6.5
6.0
5.5
5.0
4.5
4.0
3.5
3.0
2.5
2.0
1.5
1.0
f1 (ppm)

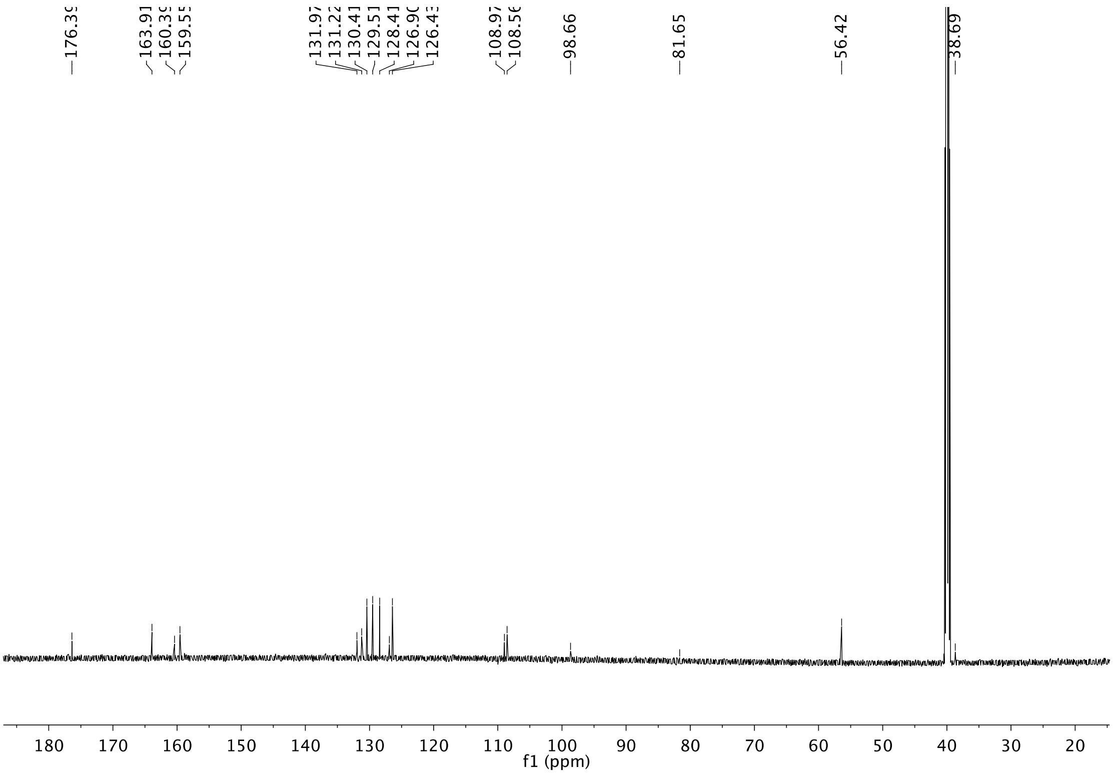
163.91
159.55
131.97
131.22
130.41
129.51
128.41
108.97
98.66
81.65
56.42
38.69
180
170
160
150
140
130
120
110
100
90
80
70
60
50
40
30
20
f1 (ppm)

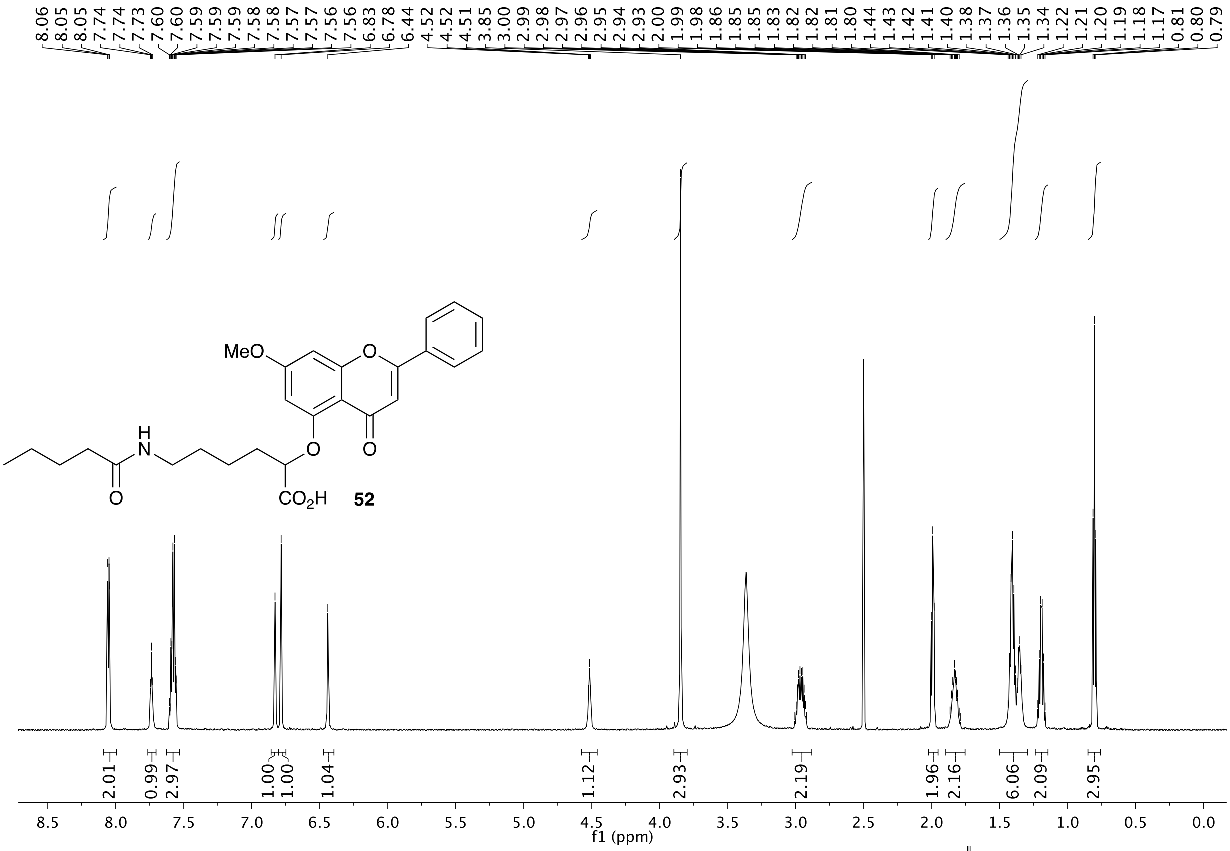
8.06 8.05 8.05 7.74 7.74 7.73 7.60 7.60 7.59 7.59 7.59 7.58 7.58 7.57 7.56 7.56 6.83 6.78 6.44 4.52 4.52 4.51 3.85 3.00 2.99 2.98 2.97 2.96 2.95 2.94 2.93 2.00 1.99 1.98 1.86 1.85 1.85 1.83 1.82 1.82 1.81 1.80 1.44 1.43 1.42 1.41 1.40 1.38 1.37 1.36 1.35 1.34 1.22 1.21 1.20 1.19 1.18 1.17 0.81 0.80 0.79
MeO
O
H
N
O
O
CO2H
O
52
2.01 0.99 2.97 1.00 1.00 1.04 1.12 2.93 2.19 1.96 2.16 6.06 2.09 2.95
f1 (ppm)

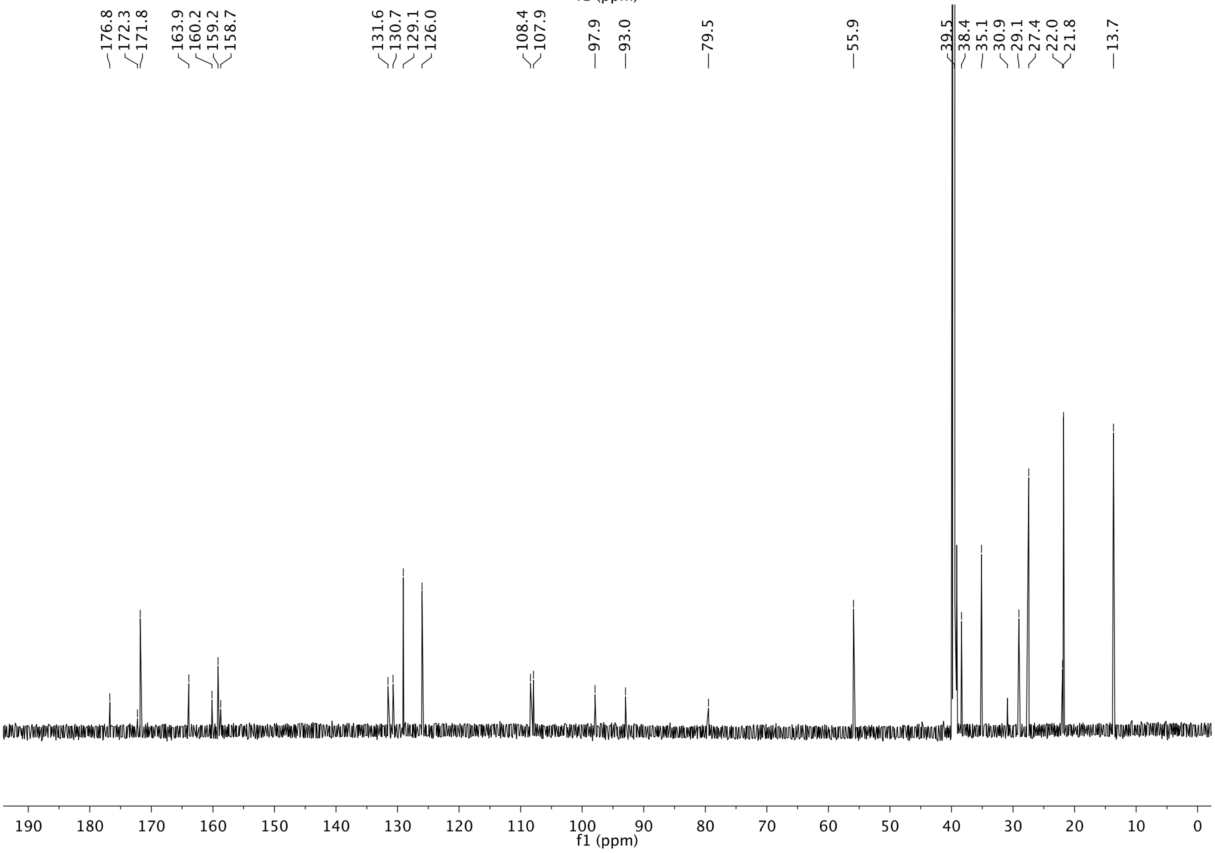
176.8 172.3 171.8 163.9 160.2 159.2 158.7 131.6 130.7 129.1 126.0 108.4 107.9 97.9 93.0 79.5 55.9 39.5 38.4 35.1 30.9 29.1 27.4 22.0 21.8 13.7
f1 (ppm)

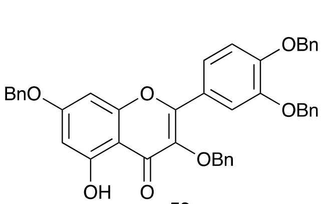

BnO
O
OBn
OBn
OBn
OH
O

53

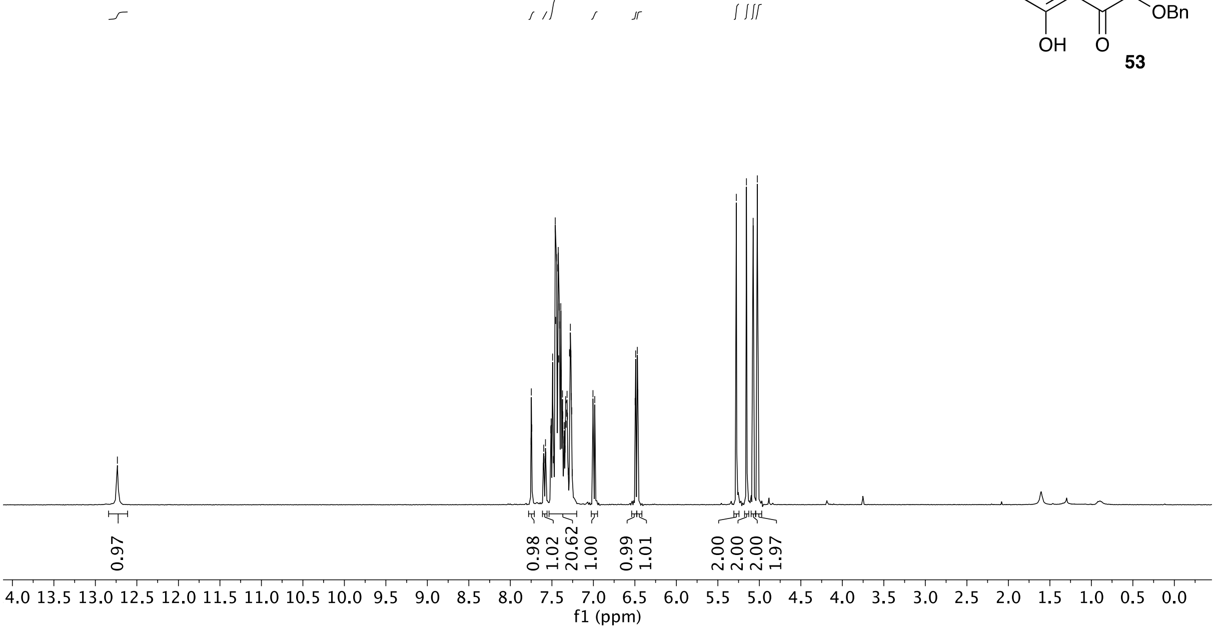

0.97
0.98
1.02
20.62
1.00
0.99
1.01
2.00
2.00
2.00
1.97
f1 (ppm)

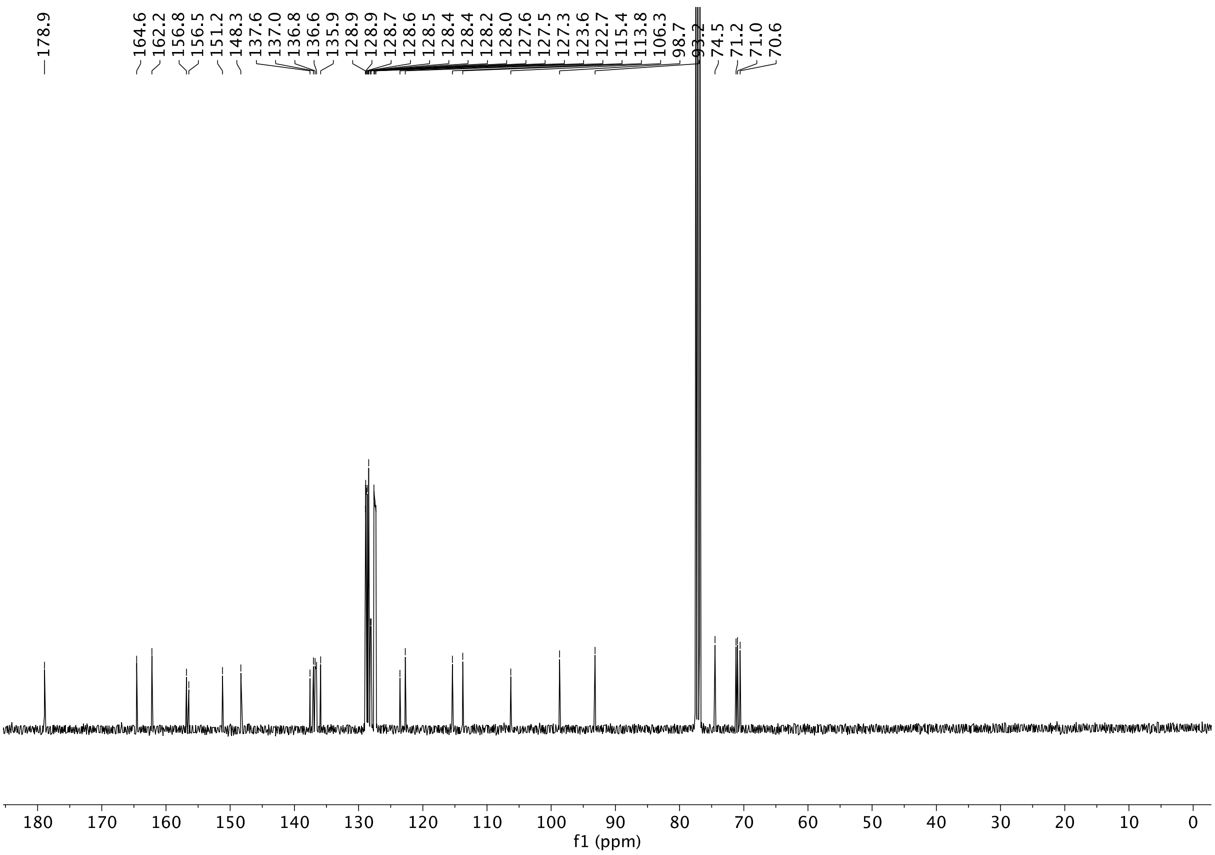

178.9
164.6
162.2
156.8
156.5
151.2
148.3
137.6
137.0
136.8
136.6
135.9
128.9
128.9
128.7
128.6
128.5
128.4
128.4
128.2
128.0
127.6
127.5
127.3
123.6
122.7
115.4
113.8
106.3
98.7
93.2
74.5
71.2
71.0
70.6
f1 (ppm)

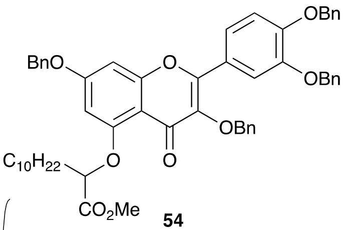
BnO
OBn
OBn
OBn
$C_{10}H_{22}$
O
O
CO_2Me

54

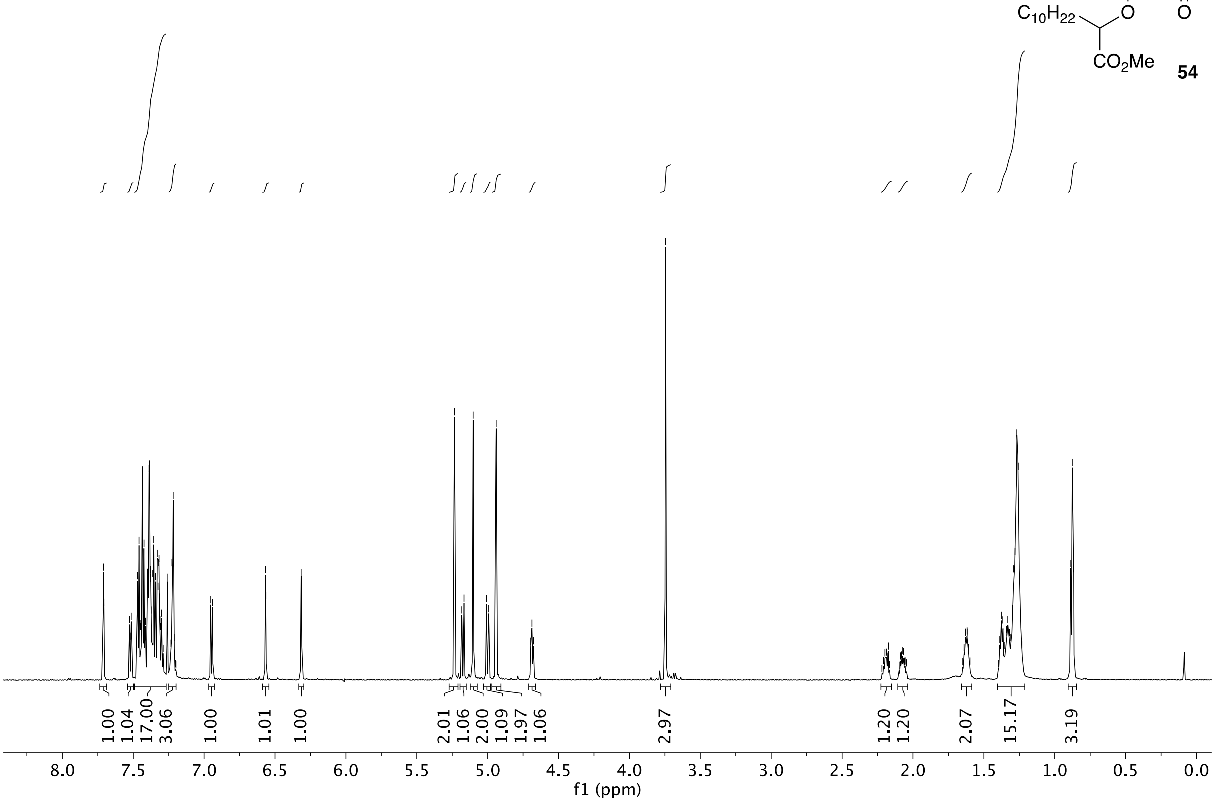
1.00
1.04
17.00
3.06
1.00
1.01
1.00
2.01
1.06
2.00
1.09
1.97
1.06
2.97
1.20
1.20
2.07
15.17
3.19
8.0 7.5 7.0 6.5 6.0 5.5 5.0 4.5 4.0 3.5 3.0 2.5 2.0 1.5 1.0 0.5 0.0
f1 (ppm)

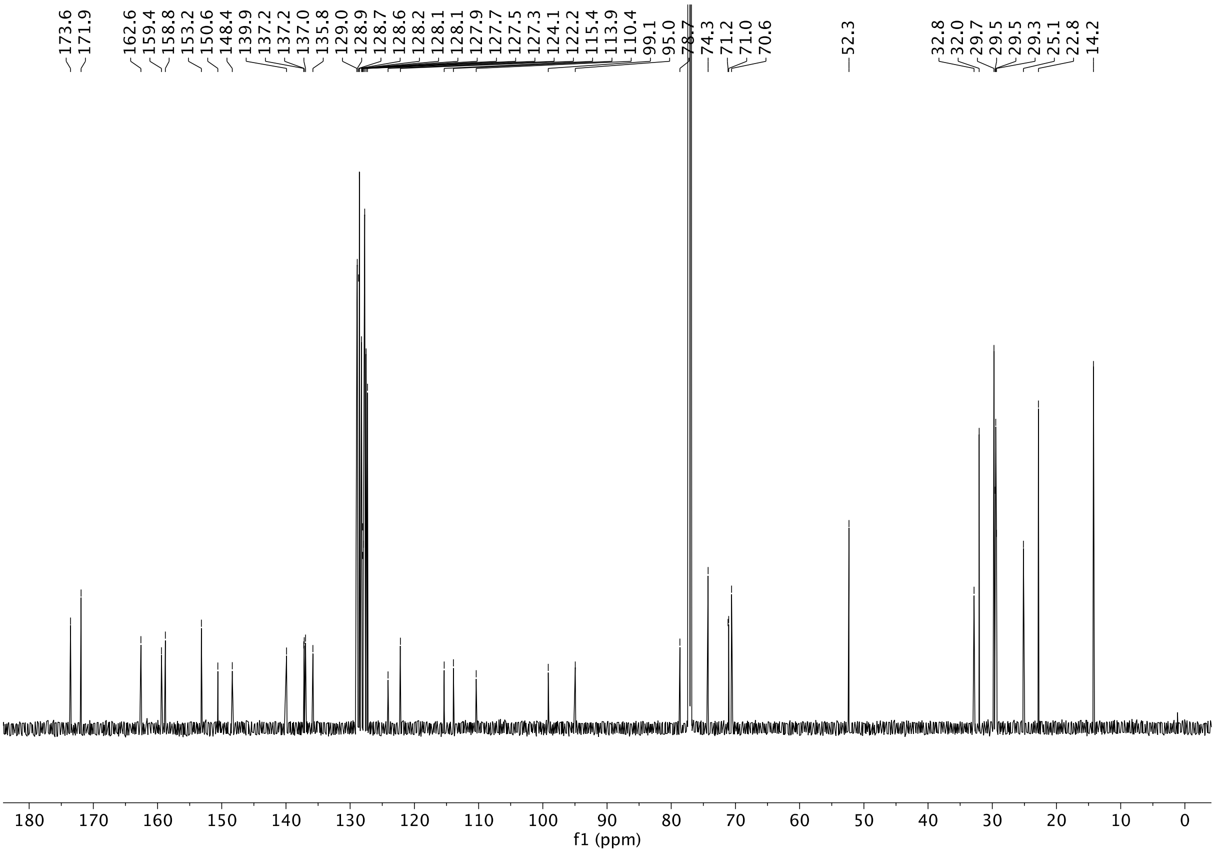
173.6
171.9
162.6
159.4
158.8
153.2
150.6
148.4
139.9
137.2
137.2
137.0
135.8
129.0
128.9
128.7
128.6
128.2
128.1
128.1
127.9
127.7
127.5
127.3
124.1
122.2
115.4
113.9
110.4
99.1
95.0
78.7
74.3
71.2
71.0
70.6
52.3
32.8
32.0
29.7
29.5
29.5
29.3
25.1
22.8
14.2
180 170 160 150 140 130 120 110 100 90 80 70 60 50 40 30 20 10 0
f1 (ppm)

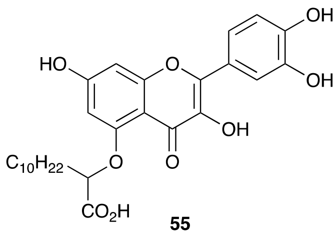
HO
OH
OH
OH
O
O
$C_{10}H_{22}$
CO_2H

55

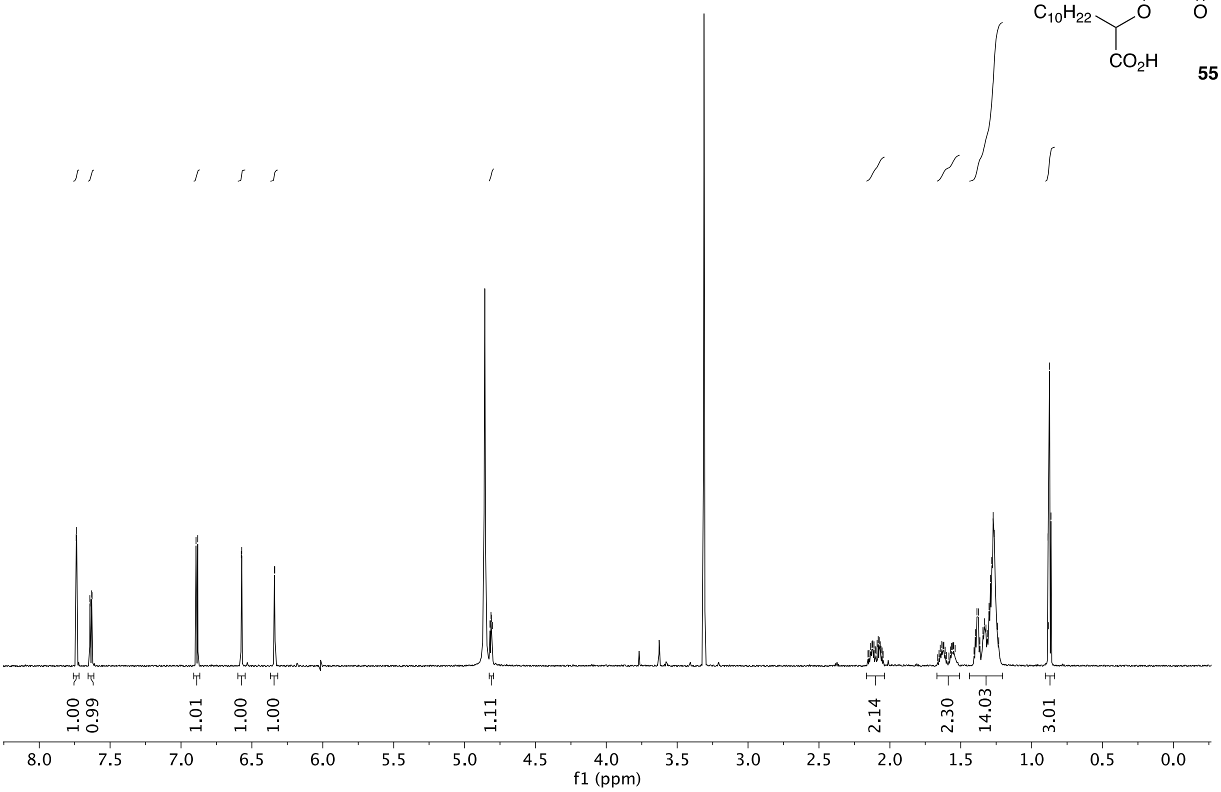
1.00
0.99
1.01
1.00
1.00
1.11
2.14
2.30
14.03
3.01
8.0 7.5 7.0 6.5 6.0 5.5 5.0 4.5 4.0 3.5 3.0 2.5 2.0 1.5 1.0 0.5 0.0
f1 (ppm)

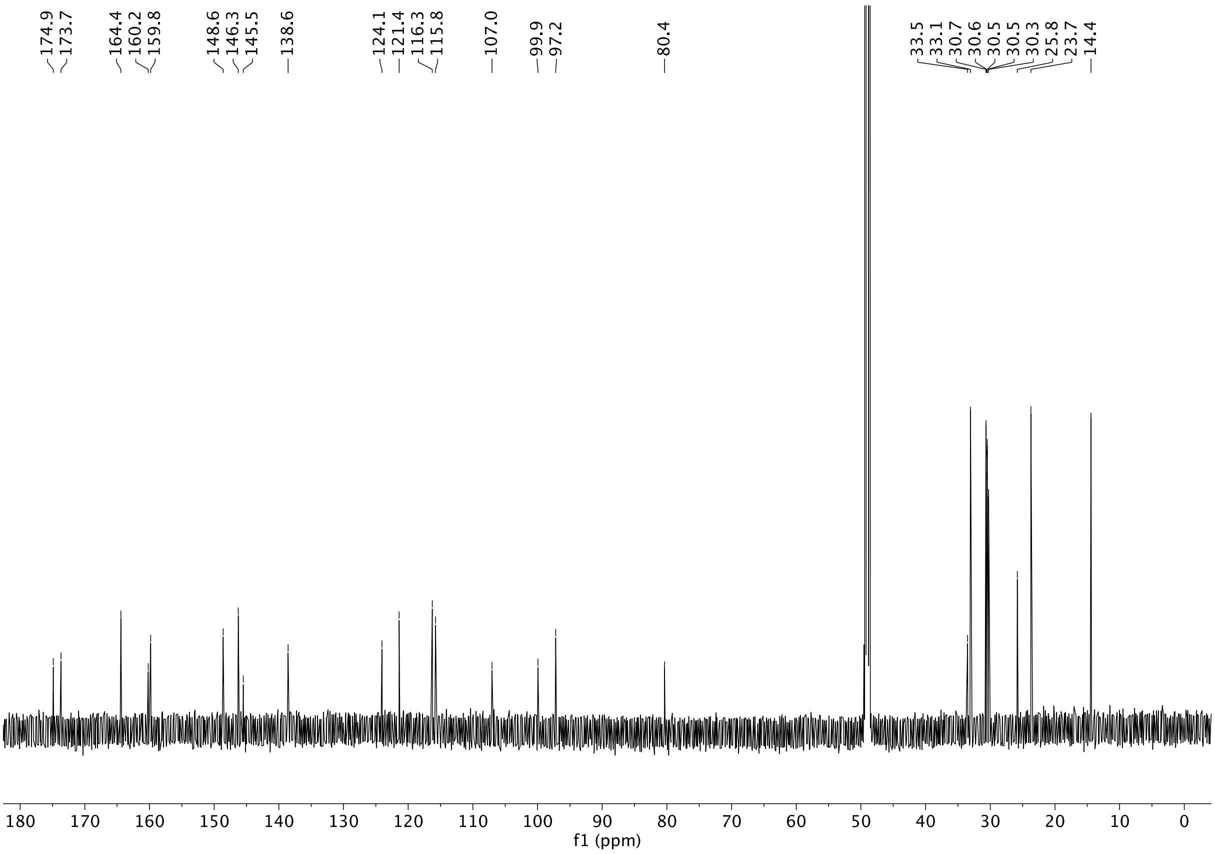
174.9
173.7
164.4
160.2
159.8
148.6
146.3
145.5
138.6
124.1
121.4
116.3
115.8
107.0
99.9
97.2
80.4
33.5
33.1
30.7
30.6
30.5
30.5
30.3
25.8
23.7
14.4
180 170 160 150 140 130 120 110 100 90 80 70 60 50 40 30 20 10 0
f1 (ppm)

References

1 Cummins, I. *et al.* Key role for a glutathione transferase in multiple-herbicide resistance in grass weeds. *Proc Natl Acad Sci U S A* **110**, 5812-5817, doi:10.1073/pnas.1221179110 (2013).

2 Cummins, I. *et al.* Cloning, characterization and regulation of a family of phi class glutathione transferases from wheat. *Plant Mol Biol* **52**, 591-603, doi:10.1023/a:1024858218804 (2003).

3 Diez, M. *et al.* Technical Report: Industrial Use of the SLS MX beam lines. *Synchr. Rad. News* **20**, 19-22 (2007).

4 Otwinowski, Z. & Minor, W. Denzo and Scalepack. *International Tables for Crystallography Volume F: Crystallography of biological macromolecules*, 226-235 (2006).

5 McCoy, A. J. *et al.* Phaser crystallographic software. *J Appl Crystallogr* **40**, 658-674, doi:10.1107/S0021889807021206 (2007).

6 Emsley, P. Tools for ligand validation in Coot. *Acta Crystallogr D Struct Biol* **73**, 203-210, doi:10.1107/S2059798317003382 (2017).

7 Murshudov, G. N. *et al.* REFMAC5 for the refinement of macromolecular crystal structures. *Acta Crystallogr D Biol Crystallogr* **67**, 355-367, doi:10.1107/S0907444911001314 (2011).

8 Uson, I. *et al.* 1.7 A structure of the stabilized REIv mutant T39K. Application of local NCS restraints. *Acta Crystallogr D Biol Crystallogr* **55**, 1158-1167, doi:10.1107/s0907444999003972 (1999).

9 Lebedev, A. A. *et al.* JLigand: a graphical tool for the CCP4 template-restraint library. *Acta Crystallogr D Biol Crystallogr* **68**, 431-440, doi:10.1107/S090744491200251X (2012).

10 Winter, G., Lobley, C. M. & Prince, S. M. Decision making in xia2. *Acta Crystallogr D Biol Crystallogr* **69**, 1260-1273, doi:10.1107/S0907444913015308 (2013).

11 Cowtan, K. The Buccaneer software for automated model building. 1. Tracing protein chains. *Acta Crystallogr D Biol Crystallogr* **62**, 1002-1011, doi:10.1107/S0907444906022116 (2006).

12 Liebeschuetz, J. W., Cole, J. C. & Korb, O. Pose prediction and virtual screening performance of GOLD scoring functions in a standardized test. *J Comput Aided Mol Des* **26**, 737-748, doi:10.1007/s10822-012-9551-4 (2012).

13 Groftehauge, M. K., Hajizadeh, N. R., Swann, M. J. & Pohl, E. Protein-ligand interactions investigated by thermal shift assays (TSA) and dual polarization interferometry (DPI). *Acta Crystallogr D Biol Crystallogr* **71**, 36-44, doi:10.1107/S1399004714016617 (2015).

14 Bruce, D., Cardew, E., Freitag-Pohl, S. & Pohl, E. How to Stabilize Protein: Stability Screens for Thermal Shift Assays and Nano Differential Scanning Fluorimetry in the Virus-X Project. *J Vis Exp*, doi:10.3791/58666 (2019).

15 Cummins, I. *et al.* Key role for a glutathione transferase in multiple-herbicide resistance in grass weeds. *P Natl Acad Sci USA* **110**, 5812-5817, doi:10.1073/pnas.1221179110
1221179110 [pii] (2013).

16 Cummins, I., Cole, D. J. & Edwards, R. A role for glutathione transferases functioning as glutathione peroxidases in resistance to multiple herbicides in black-grass. *Plant J* **18**, 285-292 (1999).
17 Dixon, D. P., Hawkins, T., Hussey, P. J. & Edwards, R. Enzyme activities and subcellular localization of members of the Arabidopsis glutathione transferase superfamily. *Journal of Experimental Botany* **60**, 1207-1218, doi:10.1093/jxb/ern365 (2009).
18 Cavaliere, C., Foglia, P., Pastorini, E., Samperi, R. & Lagana, A. Identification and mass spectrometric characterization of glycosylated flavonoids in Triticum durum plants by high-performance liquid chromatography with tandem mass spectrometry. *Rapid Commun Mass Spectrom* **19**, 3143-3158, doi:10.1002/rcm.2185 (2005).
19 Bruder, M., Haseler, P. L., Muscarella, M., Lewis, W. & Moody, C. J. Synthesis of the oxepinochromone natural products ptaeroxylin (desoxykarenin), ptaeroxylinol, and eranthin. *J Org Chem* **75**, 353-358, doi:10.1021/jo902117e (2010).
20 Kraus, G. A. & Gupta, V. Divergent Approach to Flavones and Aurones via Dihaloacrylic Acids. Unexpected Dependence on the Halogen Atom. *Org. Lett.* **12**, 5278-5280, doi:10.1021/ol1023294 (2010).
21 Schroth, W., Schmiedl, D., Jahn, U. & Spitzner, R. A SIMPLIFIED PREPARATION OF BIS(2,2-DICHLOROVINYL)KETONE. *Zeitschrift Fur Chemie* **29**, 419-420 (1989).
22 Chen, Y.-H. *et al.* Evaluation of the structure–activity relationship of flavonoids as antioxidants and toxicants of zebrafish larvae. *Food chemistry* **134**, 717-724 (2012).
23 Ribeiro, D. *et al.* Modulation of human neutrophils' oxidative burst by flavonoids. *Eur. J. Med. Chem.* **67**, 280-292, doi:10.1016/j.ejmech.2013.06.019 (2013).
24 Kudo, N., Perseghini, M. & Fu, G. C. A versatile method for Suzuki cross-coupling reactions of nitrogen heterocycles. *Angewandte Chemie-International Edition* **45**, 1282-1284, doi:10.1002/anie.200503479 (2006).
25 Augstein, J., Cairns, H., Hunter, D. & J., K. Pharmacologically active tetrazole derivatives. Germany patent (1972).
26 Daskiewicz, J. B., Bayet, C. & Barron, D. Regioselective syntheses of 6-(1,1-dimethylallyl)-chrysin and 8-(3,3-dimethylallyl) chrysin. *Tetrahedron* **58**, 3589-3595, doi:10.1016/s0040-4020(02)00309-5 (2002).
27 Zhu, Z.-Y. *et al.* Synthesis and antitumor activity evaluation of chrysin derivatives. *European Journal of Medicinal Chemistry* **75**, 297-300, doi:http://dx.doi.org/10.1016/j.ejmech.2013.12.044 (2014).
28 Comte, G. *et al.* C-isoprenylation of flavonoids enhances binding affinity toward P-glycoprotein and modulation of cancer cell chemoresistance. *J. Med. Chem.* **44**, 763-768, doi:10.1021/jm991128y (2001).
29 Echavarren, A. M. & Stille, J. K. Palladium-catalyzed coupling of aryl triflates with organostannanes. *Journal of the American Chemical Society* **109**, 5478-5486, doi:10.1021/ja00252a029 (1987).
30 Halbrook, J. *et al.* Preparation of arylmorpholines as inhibitors of DNA-dependent protein kinase and methods to potentiate cancer treatment. (2002).
